# The Global Prevalence of and Factors Associated with Parasitic Coinfection in People Living with Viruses: A Systematic Review and Meta-Analysis

**DOI:** 10.3390/pathogens14060534

**Published:** 2025-05-27

**Authors:** Yan Ge, Huaman Liu, Ningjun Ren, Abdul Qadeer, Ian Kim B. Tabios, Ian Kendrich C. Fontanilla, Lydia R. Leonardo, Banchob Sripa, Guofeng Cheng

**Affiliations:** 1 Shanghai Tenth People’s Hospital, Institute for Infectious Diseases and Vaccine Development, Tongji University School of Medicine, Shanghai 200070, China; yge@tongji.edu.cn (Y.G.); 2031104@tongji.edu.cn (H.L.); qadeerktk848@yahoo.com (A.Q.); 2 Department of Immunology and Pathogen Biology, Tongji University School of Medicine, Shanghai 200331, China; 3 School of Public Health, Southwest Medical University, Luzhou 646000, China; ningjunren@gmail.com; 4 Parasite and Vector Biology and Control Program, University of the Philippines Los Baños Zoonoses Center, Los Baños, Laguna 4031, Philippines; ibtabios2@up.edu.ph; 5 Institute of Biology, College of Science, University of the Philippines Diliman, Quezon City 1101, Philippines; icfontanilla@up.edu.ph; 6 Office of Research Coordination, University of the East, CM Recto Avenue, Manila 1008, Philippines; lydialeonardo1152@gmail.com; 7 Department of Biology, College of Arts and Sciences, University of the Philippines Manila, Padre Faura, Manila 1000, Philippines; 8 Graduate School, University of the Ease Ramon Magsaysay Memorial Medical Center, 64 Aurora Blvd., Quezon City 1100, Philippines; 9 WHO Collaborating Centre for Research and Control of Opisthorchiasis (Southeast Asian Liver Fluke Disease), Tropical Disease Research Center, Faculty of Medicine, KhonKaen University, KhonKaen 40002, Thailand; banchob@kku.ac.th; 10 Clinical Center for Brain and Spinal Cord Research, Tongji University, Shanghai 200092, China; 11 Affiliated Shanghai Blue Cross Brain Hospital, School of Medicine, Tongji University, Shanghai 200020, China

**Keywords:** helminth, protozoan, human immunodeficiency virus, hepatitis virus, dengue virus

## Abstract

Coinfection with parasites and viruses can exacerbate disease transmission, outcomes and therapy. This study searched the Web of Science, PubMed, Scopus and JSTOR databases for publications on the prevalence of parasitic coinfection in people living with viruses from 1 January 2005 to 30 April 2022, and 356 studies were included and systematically reviewed. A meta-analysis was performed to assess the global prevalence of and factors potentially associated with parasitic infection (helminths and protozoa) in virus-infected people, and the infection burden was estimated. A variety of parasites (29 families, 39 genera, and 63 species) and viruses (8 kinds) were identified. The prevalence of parasitic coinfection in (all) virus-infected people was estimated to be 21.34% (95% CI 17.58–25.10, 5593 of 29,190 participants) and 34.13% (95% CI 31.32–36.94, 21,243/76,072 participants) for helminths and protozoa, respectively. Specially, in human immunodeficiency virus (HIV)-infected people, the global prevalence was 19.96% (95% CI 16.18–23.74) for helminths and 34.18% (95% CI 31.33–37.03) for protozoa, respectively. The global prevalence of protozoa was 41.79% (95% CI 15.88–67.69) in hepatitis B virus (HBV)-infected people and 17.75% (95% CI 3.54–31.95) in DENV-infected people, respectively. The global burden of parasitic infections in HIV-infected people was 7,664,640 for helminths and 13,125,120 for protozoa, respectively, and that in HBV- and dengue virus (DENV)-infected people was 137,019,428 and 629,952, respectively. The prevalence of parasitic coinfection at the family, genus, and species levels in virus- or HIV-infected people were comprehensively estimated and further analyzed by subgroups. Among the most commonly identified parasites, the five helminth genera with the highest prevalence in HIV-infected people were *Schistosoma* (12.46%, 95% CI 5.82–19.10), *Ascaris* (7.82%, 95% CI 6.15–9.49), *Strongyloides* (5.43%, 95% CI 4.11–6.74), *Trichuris* (4·82%, 95% CI 2.48–7.17) and *Ancylostoma* (2.79%, 95% CI 1.32–4.27), whereas the top five protozoan genera were *Toxoplasma* (48.85%, 95% CI 42.01–55.69), *Plasmodium* (34.96%, 95% CI 28.11–41.82), *Cryptosporidium* (14.27%, 95% CI 11.49–17.06), *Entamoeba* (12.33%, 95% CI 10.09–14.57) and *Blastocystis* (10.61%, 95% CI 6.26–14.97). The prevalence of parasitic coinfection in virus-infected people was associated with income level. The findings provide valuable global epidemiological information for informing normative guidance, improving surveillance, and developing public healthcare strategies.

## 1. Introduction

Viral infections pose significant global public health challenges, annually causing millions of deaths worldwide. There is a sustained high occurrence of chronic viral infections such as human immunodeficiency virus/acquired immunodeficiency syndrome (HIV/AIDS) and viral hepatitis. About 39.9 million people were living with HIV by the end of 2023, and around 630,000 people died from HIV-related causes in 2023 [1]. Viral hepatitis, as the second leading infectious causes of death globally, was responsible for 1.3 million deaths in 2022 [2]. There were an estimated 304 million people living with hepatitis B virus (HBV) and hepatitis C virus (HCV) infections in 2022 [2]. Additionally, the emergence of pandemic coronavirus disease 2019 (COVID-19) has highlighted the critical need for effective measures to prevent and control viral infections [1,2,3,4,5].

Similarly, parasitic infections constitute a major global health burden, causing clinical disorders ranging from iron-deficiency anemia to growth retardation in children and other physical and mental health impairments [6,7]. Malaria alone accounted for an estimated 263 million cases and 597,000 fatalities in 2023 [8]. Coinfections with viruses and parasites establish a dynamic interplay that alters fundamental biological mechanisms, including pathogen immune evasion and dysregulation of host inflammatory homeostasis, thereby reshaping disease transmission and exacerbating clinical outcomes through synergistic pathogenesis [9,10,11]. HIV infection drives the progressive depletion of CD4+ T lymphocytes, resulting in an immunocompromised state that predisposes to opportunistic infections with accelerated progression to advanced disease states, including AIDS-defining malignancies [12,13]. Parasitic comorbidities have become a leading cause of high morbidity and mortality in individuals with HIV/AIDS [14]. Notably, HIV or dengue virus (DENV) coinfection significantly elevates malaria severity risk through mechanisms involving vascular dysfunction and altered cytokine networks, creating therapeutic challenges in differential diagnosis [15,16,17]. Helminths induce a T-helper 2 (Th2)-type immune response, which inhibits Th1-type antiviral immune defense mechanisms [11]. Therefore, deciphering the complex epidemiology of parasite–virus coinfection in a global perspective is essential for formulating precision public health interventions.

Most of the epidemiological studies on parasite–virus coinfection, however, have focused on one kind of virus within geographically constrained settings. Only a few of them have adopted a global perspective, predominantly limited to HIV coinfection with protozoa such as *Toxoplasma* and *Blastocystis* [18,19,20]. The global epidemiology of parasitic coinfection among virus-infected people and the profiles of such coinfection remain underexplored. In particular, regarding helminth and virus global syndemics, there is a critical knowledge gap despite a quarter of the world’s population being at risk of soil-transmitted helminth (STH) infections defined as neglected tropical diseases (NTDs) by World Health Organization (WHO) [21,22,23]. In this systematic review and meta-analysis, we aimed to investigate the global prevalence of parasitic coinfection in virus-infected people and to identify potential associated factors, which would inform the development of public healthcare strategies in risk regions and chart investigative priorities for subsequent studies.

## 2. Materials and Methods

### 2.1. Search Strategy and Selection Criteria

This systematic review and meta-analysis followed the Preferred Reporting Items for Systematic Reviews and Meta-Analysis (PRISMA) guidelines [24] (Table S1) and was registered with the International Prospective Register of Systematic Reviews (PROSPERO, registration number CRD42023338483). We searched the Web of Science, PubMed, Scopus, and JSTOR databases for publications from 1 January 2005 to 30 April 2022 by using terms “Parasite virus”, “Parasite virus co-infection”, “Parasite co-infection”, “Helminths virus”, “Helminths virus co-infection” or “Helminths co-infection” (Text S1). After duplication removal, the titles and abstracts were independently screened by two reviewers (AQ and HL). We included studies (original research articles, observational studies, experimental studies, or surveys) with prevalence data on parasitic coinfection in people living with viruses. Included studies were confined to the English language. Studies were excluded if they were review articles, book chapters, patents, unpublished data, conference papers, or nonhuman studies. Further full-text screening of potentially eligible articles was conducted by three reviewers (YG, HL, and AQ). Studies without relevant prevalence rates were excluded. The extracted papers were cross-checked. When any discrepancy arose, ambiguous articles were discussed with two reviewers (YG and GC) to reach consensus. In total, four reviewers conducted data extraction. The included studies were divided into helminth–virus and protozoan–virus coinfection groups for further process.

### 2.2. Data Extraction and Quality Assessment

From each eligible study, we extracted the information of first author, publication year, country, WHO region, income level, total number of virus-infected people (sample size), number of people with parasitic coinfection in virus-infected people, study design, diagnostic method, viral name, classification of parasites (including family, genus, and species), age at risk, and gender at risk.

We assessed study quality following the Grading of Recommendations Assessment, Development and Evaluation method as previously described with some modifications [20,25]. Briefly, the data were graded into high or low rank on the basis of three factors, study design, sample size, and diagnostic method. For each factor, high rank was scored one point, and low rank, zero points. The total score for each study was calculated (Text S2). Using the three-point scale, individual studies were categorized into three tiers: high- (3 points), moderate- (2 points), and low-quality (≤1 points) groups (Tables S2 and S3).

### 2.3. Statistical Analysis

We conducted a systematic review and meta-analysis to assess the global prevalence of parasitic coinfection in people living with viruses (all viruses and specific viruses with ≥5 identified articles) with 95% CIs for both overall and subgroups of income level (high, upper middle, lower middle, and low), region, country, gender, age, sample size (≤200 and >200), and publication year (<2014 and ≥2014). The prevalence rates of parasites at family, genus, and species levels in virus-infected people were also evaluated. For the mainly identified parasitic genera (with ≥30 included articles), the prevalence in HIV-infected people was further analyzed by subgroups of region, country, income level, age, and gender. To comprehensively analyze the included studies, we used both common-effect and random-effects models. When there was high heterogeneity (I^2^ > 50%, *p* < 0.1), random-effects model analysis was used for final statistics. We analyzed data by using R (version:4.1.0) “meta” (version 4.9-6). The forest plots of prevalence were generated by the package “meta”. The effect of subgroup was analyzed by using the rma function. The R function metaprop was used to calculate effect size and heterogeneity. The assessment of heterogeneity between studies was conducted using the Cochran’s Q (represented by *χ*^2^ and *p* value) and I^2^ statistics. The I^2^ statistics describe the percentage of variation between the studies due to heterogeneity. To assess publication bias, funnel plots were conducted to visually assess the symmetry of the distribution of effect sizes against their precision metrics, which were further statistically evaluated by Egger’s linear regression test [26] and Begg–Mazumdar nonparametric rank correlation test [27].

The infection burden was calculated by multiplying our estimated prevalence rate of parasites in virus (HIV, HBV, or DENV)-infected people by the global number of virus (HIV, HBV, or DENV)-infected people [28,29].

## 3. Results

### 3.1. Study Selection

**Figure 1:**
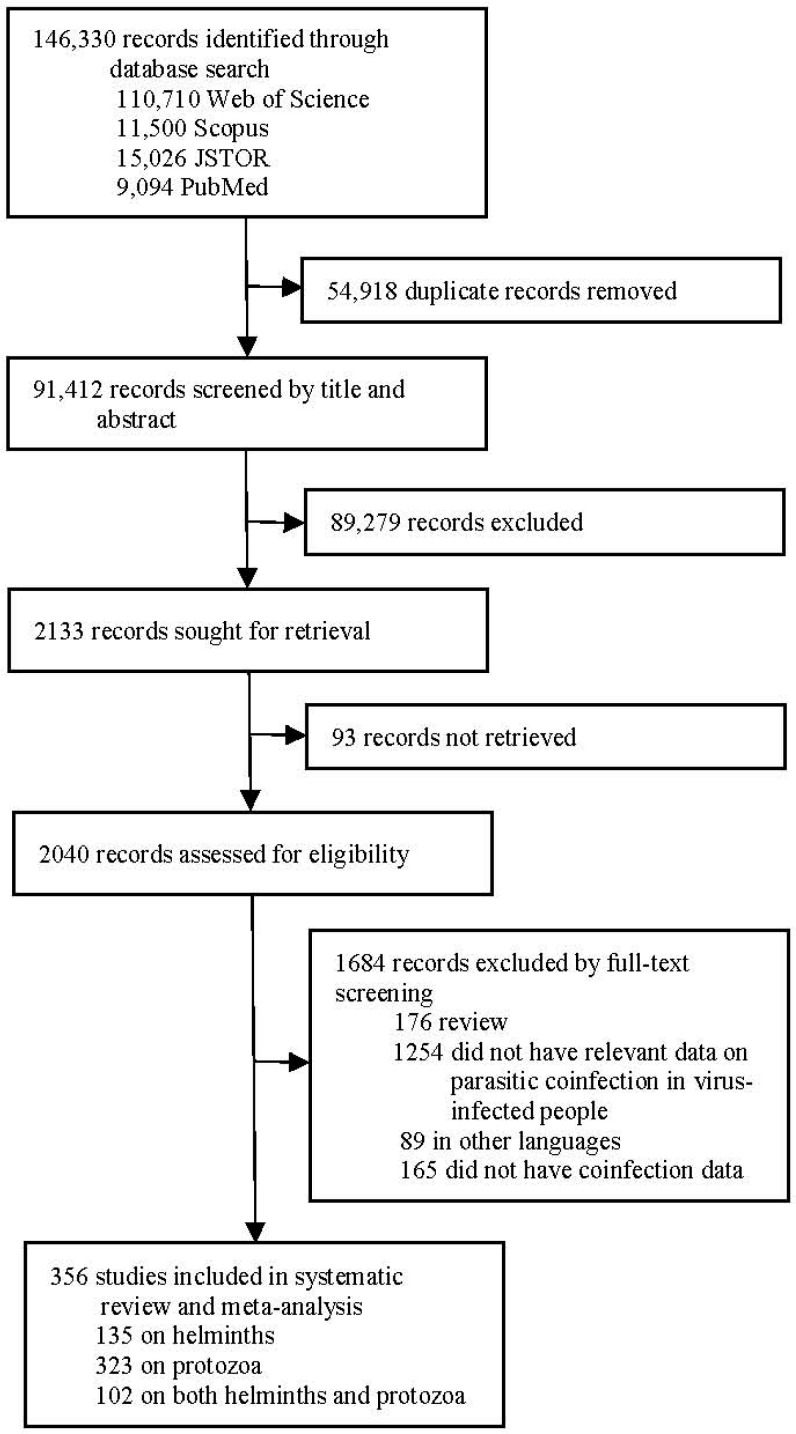
Flow diagram of study selection.

Our search identified 146,330 publications (Figure 1). After the removal of 54,918 duplicate records, 89,279 irrelevant records were excluded by title and abstract screening. Then, 2040 records were retrieved for the assessment of eligibility. After full-text review, 356 studies were included in our meta-analysis. Notably, 102 out of the 356 included articles investigated concurrent infections involving both helminths and protozoa in virus coinfection scenarios. Therefore, these articles were included in both parasite-specific analytical categories during data stratification. Consequently, 135 and 323 studies were about helminth and protozoan coinfections in virus-infected people, respectively. In the helminth–virus group, 12 out of 135 articles had multiple parasitism but without a definitive description (only included in helminth species coinfection subgroup), whereas the other 123 articles were included in all the helminth coinfection meta-analyses. In the protozoan–virus group, 15 out of 323 articles had multiple parasitism but without definitive description (only included in protozoan species coinfection subgroup), whereas the other 308 articles were included in all the protozoan coinfection meta-analyses.

### 3.2. The Global Prevalence of Parasitic Infection in People Living with Viruses

Overall, our study identified a diverse range of coinfecting parasites in people living with viruses. This included helminths from 16 families, 18 genera, and 20 species as well as protozoa from 13 families, 21 genera, and 43 species. These parasites were found in individuals infected with eight kinds of viruses, namely hepatitis B virus (HBV), hepatitis C virus (HCV), HIV, Chikungunya virus, DENV, human T-lymphotropic virus type 1 (HTLV-1), SARS-CoV-2, and influenza virus (Tables S2 and S3). We observed that the number of parasitic coinfections in people living with viruses from lower-middle-income countries was the highest, which was 1543 for helminth and 9977 for protozoan coinfections, respectively (Table 1 and Table 2). The majority of the identified articles originated from the regions of eastern and southern Africa, Asia and the Pacific, and western and central Africa (Table 1 and Table 2). Our study revealed that the estimated global prevalences of pooled helminth and protozoan coinfection in virus-infected people were 21.3% (95% CI 17.6–25.1) and 31.41% (95% CI 31.3–36.91), respectively (Table 1 and Table 2). Specially, in HIV-infected people, the estimated global prevalence of pooled helminth and protozoan coinfections was 19.96% (95% CI 16.18–23.74) and 34.18% (95% CI 31.33–37.03), respectively (Table 3 and Table 4). For HBV-infected people, the estimated global prevalence of protozoan coinfection was 41.79% (95% CI 15.88–67.69), and for DENV-infected people, it was 17.75% (95% CI 3.54–31.95) (Table 4). Notably, our study found that *Plasmodium* was the only identified parasitic genus that coinfected with DENV (Tables S2 and S3).

### 3.3. The Global Prevalence of Parasitic Coinfection at the Family, Genus, and Species Levels in Virus- or HIV-Infected People

A subgroup analysis of studies by family, genus, or species for the most commonly identified parasitic coinfection in both virus-infected people and HIV-infected people (people living with HIV, PLWH) was performed. For virus-infected people, the five most prevalent helminth families were Onchocercidae, Schistosomatidae, Opisthorchiidae, Ascarididae, and Strongylidae for helminths, while the top protozoan families were Sarcocystidae, Plasmodiidae, Trypanosomatidae, Trichomonadidae, and Blastocystidae (Figure S1A,B). Similarly, in virus-infected people, the top five most prevalent helminth genera were *Opisthorchis*, *Schistosoma*, *Ascaris*, *Strongyloides*, and *Trichuris*, and the protozoan genera were *Toxoplasma*, *Plasmodium*, *Leishmania*, *Trypanosoma*, and *Trichomonas* (Figure S2A,B).

The five most prevalent helminth genera in HIV-infected people were *Schistosoma* (12.46%, 95% CI 5.82–19.10), *Ascaris* (7.82%, 95% CI 6.15- 9.49), *Strongyloides* (5.43%, 95% CI 4.11–6.74), *Trichuris* (4.82%, 95% CI 2.48–7.17), and *Ancylostoma* (2.79%, 95% CI 1.32–4.27), followed by *Taenia* (2.19%, 95% CI 1.52–2.85), *Hymenolepis* (0.51%, 95% CI 0.37–0.64), and *Enterobius* (0.83%, 95% CI 0.55–1.10) (Table S4 and Figure S4A). The top five definitive helminth species in PLWH were *Schistosoma haematobium*, *Ascaris lumbricoides*, *Schistosoma mansoni*, *Strongyloides stercoralis*, and *Trichuris trichiura* (Figure S3A). The five most prevalent protozoan genera in HIV-infected people were *Toxoplasma* (48.85%, 95% CI 42.01–55.69), *Plasmodium* (34.96%, 95% CI 28.11–41.82), *Leishmania* (30.50%, 95% CI 21.65–39.35), *Trypanosoma* (20.32%, 95% CI 0.00–42.37), and *Trichomonas* (14.75%, 95% CI 8.75–20.75), followed by *Cryptosporidium* (14.27%, 95% CI 11.49–17.06), *Entamoeba* (12.33%, 95% CI 10.09–14.57), *Blastocystis* (10.61%, 95% CI 6.26–14.97), *Endolimax* (6.06%, 95% CI 3.03–9.09), *Giardia* (4.99%, 95% CI 4.18–5.81), *Isospora* (4.58%, 95% CI 3.37–5.80), *Cyclospora* (3.08%, 95% CI 1.89–4.28), and *Iodamoeba* (2.00%, 95% CI 1.08–2.92) (Table S4 and Figure S4B). The top ten definitive protozoan species with high prevalence rates in PLWH included *Toxoplasma gondii*, *Plasmodium falciparum*, *Trichomonas vaginalis*, *Trypanosoma cruzi*, *Cryptosporidium parvum*, *Cryptosporidium hominis*, *Entamoeba histolytica*/*dispar*, *Blastocystis hominis*, *Entamoeba coli*, and *Giardia lamblia* (Figure S3B).

### 3.4. Factors Potentially Associated with the Prevalence of Parasite Coinfection in People Living with Viruses

**Figure 2:**
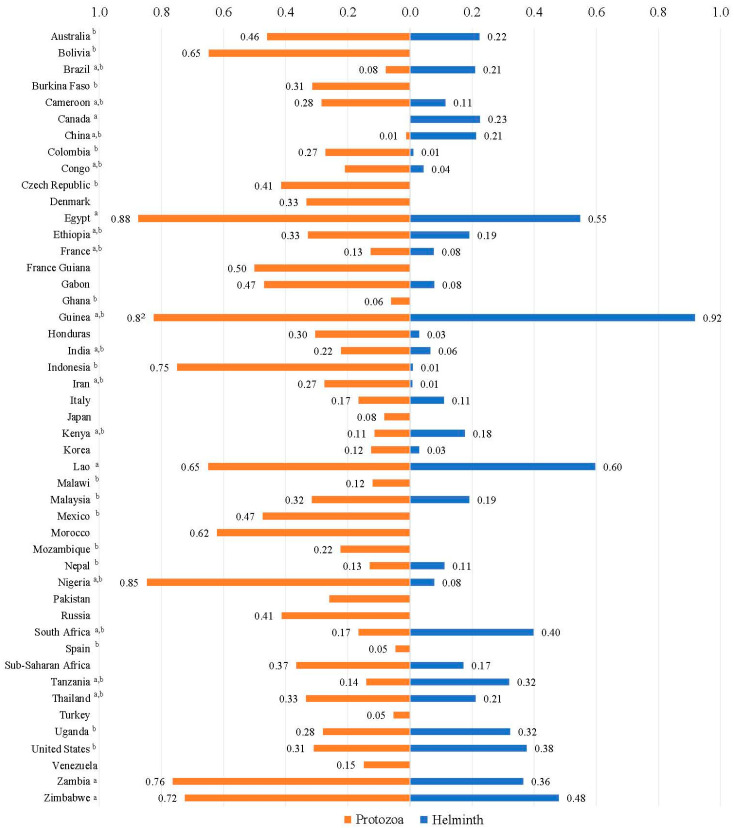
The prevalence of parasitic coinfections in virus-infected people in different countries. ^a^ labels countries with ≥2 articles on helminth coinfections in virus-infected people; ^b^ labels countries with ≥2 articles on protozoan coinfections in virus-infected people.

We further assessed several key factors potentially associated with parasite–virus coinfection. First, our subgroup analysis revealed a notable association between income level and the prevalence of parasitic coinfection in virus-infected people. Specifically, the prevalence of helminth coinfection in virus-infected people was the highest in low-income-level countries 26.78% (95% CI 17.58–35.98), compared with upper-middle (22.85%, 95% CI 12.95–32.76)-, lower-middle (19.51%, 95% CI 14.63–24.39)-, and high (16.26%, 95% CI 8.25–24.27)-income-level countries (Table 1). A similar trend was observed for protozoan infection in virus-infected people, with the highest prevalence in low-income-level countries (37.76%, 95% CI 30.25–45.27), followed by lower-middle (35.55%, 95% CI 31.76–39.34)-, high (28.09%, 95% CI 19.66–36.51)-, and upper-middle (27.66%, 95% CI 22.41–32.91)-income level countries (Table 2). Second, the prevalence of helminth coinfection in virus-infected people was notably high in the Middle East and North Africa (30.86%, 95% CI 9.20–52.51), eastern and southern Africa (27.55%, 95% CI 21.72–33.37), and western and central Africa (20.34%, 95% CI 11.37–29.30) (Table 1). These three regions also exhibited high rates of protozoan coinfection in virus-infected people, with prevalences of 39.01% (95% CI 29.57–48.45) in the Middle East and North Africa, 37.11% (95% CI 31.46–42.77) in western and central Africa, and 33.28% (95% CI 27.35–39.22) in eastern and southern Africa, respectively (Table 2). Third, among the most commonly identified countries, Tanzania, Ethiopia, Nigeria and India showed relatively high prevalence rates of helminth coinfection in virus-infected people, whereas relatively high prevalence rates of protozoan coinfection in virus-infected people were observed in Nigeria, Kenya, Thailand, Ethiopia, Burkina Faso, Cameroon, Iran, India, Brazil, and Ghana (Figure 2). Fourth, our analysis indicated that sample size significantly impacted the prevalence of parasitic coinfection in virus-infected people. Specifically, studies with sample sizes greater than 200 reported lower prevalence rates of both helminth and protozoan coinfections in virus-infected people compared with those with sample size of 200 or fewer (Table 1 and Table 2). Fifthly, age appeared to influence the prevalence of protozoan coinfection in virus-infected people but not that of helminth coinfection (Figure S5A,B). Individuals in the <35 age group (44.6%, 95% CI 30.3–58.8) exhibited a higher prevalence of protozoan coinfection in virus-infected people than those in the ≥35 age group (28.2%, 95% CI 20.4–36.1). Finally, our findings showed no significant association between the prevalence of either helminth coinfection or protozoan coinfection and factors such as gender or publication year (Figures S6A,B and S7A,B).

We also performed a subgroup analysis study of parasitic coinfection in PLWH. First, the subgroup analysis by region indicated that the prevalence rates of helminth coinfection in PLWH were significantly higher in both the regions of eastern and southern Africa (27.63%, 95% CI 21.66–33.59) and western and central Africa (20.65%, 95% CI 11.37–29.94) than in other regions. In terms of protozoan coinfection, the most commonly identified regions with the highest prevalence rates in PLWH were the Middle East and North Africa (37.41%, 95% CI 28.20–46.62), western and central Africa (36.16%, 95% CI 30.48–41.84), and eastern and southern Africa (34.26%, 95% CI 28.16–40.35) (Table 3 and Table 4). Second, in PLWH, the most commonly identified country with helminth coinfection rates surpassing the global average was Tanzania, followed by Guinea, the Lao People’s Democratic Republic, Zimbabwe, South Africa, Zambia, Canada, Thailand, and Brazil (Table 3), whereas the most commonly identified countries with protozoan coinfection rates exceeding the global average were Uganda, Kenya, Cameroon, Thailand, Ethiopia, Iran, and Nigeria, followed by Indonesia, Bolivia, Guinea, Australia, Mexico, the Czech Republic, Burkina Faso, the United States, and Malaysia (Table 4). Additionally, subgroup analyses by factors such as income level, age, gender, sample size, and publication year indicated that the patterns of parasitic coinfection prevalence in PLWH were similar to those observed in virus-infected people (Figures S6–S10).

Visual inspection of funnel plots demonstrated asymmetry in both helminth–virus and protozoan–virus coinfection studies (Figure S11A,B). The asymmetry was statistically confirmed by Egger’s linear regression test and Begg–Mazumdar rank correlation test with a significance of (*p* < 0.05) (Texts S3 and S4), suggesting that there was potential publication bias in the pooled prevalence of helminth or protozoan coinfection in people living with viruses.

### 3.5. The Global Burden of Parasitic Coinfection in Virus-Infected People

In Table 3 and Table 4, we present our calculations of the global burden of parasitic coinfections in PLWH based on our estimates of parasitic prevalence rates in PLWH and the number of PLWH reported from the WHO and GBD 2019 [28,29]. Our estimates indicated that there were approximately 7,664,640 (6,213,120–9,116,160) cases of helminth coinfection and 13,125,120 (12,030,720–14,219,520) cases of protozoan coinfection worldwide in PLWH. Among the most commonly identified regions, the highest estimated number of helminth coinfections in PLWH was found in the eastern and southern Africa region, followed by the western and central Africa region, the Asia and the Pacific region, the western and central Europe and North America region, and the Latin America and the Caribbean region, whereas the highest estimated number of protozoan coinfections in PLWH was in the eastern and southern Africa region, followed by the Asia and the Pacific region, the western and central Africa region, the Latin America and the Caribbean region, the western and central Europe and North America region, and the Middle East and North Africa region. At the country level, the top five countries with high estimated numbers of helminth coinfections in PLWH were South Africa, Zimbabwe, Tanzania, Zambia, and Uganda. For protozoan coinfections in PLWH, the leading countries included South Africa, Zambia, Zimbabwe, India, and Nigeria (Table 3 and Table 4). Additionally, we calculated the global numbers of protozoan coinfection in DENV- and HBV-infected people, which were estimated to be 629,952 (142,893–1,086,124) and 137,019,428 (52,197,877–221,840,979), respectively, using the same methodology applied in PLWH.

## 4. Discussion

To our knowledge, our study conducted the most comprehensive systematic review and meta-analysis to date on the global prevalence of parasitic coinfection at the levels of family, genus and species in people living with viruses. Our findings extended beyond previous research, which had typically focused on one parasitic genus in people coinfected with specific virus [18,19,20,30]. We provided detailed global prevalence rates of a wide array of parasites in virus-infected people and documented the coinfection profiles worldwide, which is valuable for developing effective public health strategies and highlighting future research directions.

Our study identified HIV as the predominant virus coinfecting with parasites. Notably, the majority of identified parasites in HIV-infected people in our study were intestinal parasites. Therapeutic intervention against intestinal parasites significantly reduces HIV viral load in chronically coinfected individuals [31], yet many intestinal parasitic infections remain classified as neglected tropical diseases and receive insufficient public health prioritization [22]. In our study, the helminth genera with relatively high prevalence in HIV-infected people, i.e., *Schistosoma*, *Ascaris*, *Strongyloides*, *Trichuris*, and *Ancylostoma*, are key intestinal helminths. Additionally, we observed unexpectedly high prevalence of *Opisthorchis* infection in HIV-infected people despite limited epidemiological documentation. The primary intestinal protozoa relevant to HIV infection were *Trichomonas*, *Cryptosporidium*, *Blastocystis*, *Entamoeba*, *Endolimax*, *Giardia*, *Isospora*, *Cyclospora*, *Iodamoeba*, and *Chilomastix*. Among them, *Cryptosporidium*, *Entamoeba* and *Giardia* were the most commonly identified, aligning with a prior report [32]. Our global prevalence estimates for *Cryptosporidium* and *Blastocystis* infections in HIV-infected people were 14.27% (95% CI 11.49–17.06) and 10·61% (95% CI 6.26–14.97), corroborating previous reports of 14.0% (95% CI 13.0–15.0) [30] and 9% (95% CI, 5–13%) [18], respectively, whereas the global prevalence rate of *Isospora* infection in HIV-infected people in our study, 4.58% (95% CI 3.37–5.80), exceeded that in a previous report, 2.5% (95% CI 2.1–2.9) [30], highlighting an underrecognized burden or geographic variability.

Among parasitic pathogens, *Schistosoma* rank as the second most significant global pathogen in terms of public health burden, following immediately behind *Plasmodium* that cause malaria [33]. In our study, the mainly identified *Schistosoma* species were *S. haematobium* and *S. mansoni*. *S. haematobium* infection causes urogenital schistosomiasis, whereas *S. mansoni* infection mainly results in intestinal schistosomiasis. Coinfection with HIV and *Schistosoma* creates a bidirectional pathogenic synergy. The soluble egg antigens (SEAs) secreted by *Schistosome* eggs drive a dominant Th2-skewed immune response [34] (Pearce EJ, 2004), which suppresses Th1-mediated antiviral immunity. This creates a permissive environment for HIV replication and latency establishment. Furthermore, *Schistosome* egg-induced inflammation upregulates CD4, CCR5, and CXCR4 expression on immune cells in mucosal tissues, potentially increasing HIV-1 target cells and viral entry efficiency [35]. Female genital schistosomiasis (FGS) induces chronic cervicovaginal ulceration, granuloma formation and epithelial barrier breakdown. These lesions elevate HIV shedding and increase susceptibility to HIV acquisition during sexual exposure [36]. Chronic schistosomiasis elevates Treg activity [37], which dampens effector T cell responses and impairs clearance of both schistosome eggs and HIV-infected cells. Coinfection with HIV and *Schistosome* correlates with severe pathology, and HIV-induced immunosuppression exacerbates schistosome-related organ damage [11].

In individuals with advanced HIV/AIDS, latent tissue cysts of *Toxoplasma* reactivate because of severe depletion of CD4+ T cells and consequent impairment of IL-12 and IFNγ immune surveillance [11,38,39]. The loss of these cytokines cripples macrophage and dendritic cell activation, enabling dormant bradyzoites to differentiate into rapidly proliferating tachyzoites [39]. This reactivation manifests clinically as severe toxoplasmic encephalitis, necrotizing pneumonitis, or disseminated disease, which are AIDS-defining opportunistic infections with high mortality rates if untreated [11,19,40]. Reciprocally, *T. gondii* infection exacerbates HIV pathogenesis through driven inflammation enhances HIV replication [11,39]. As the most prevalent protozoa genus in PLWH in our study, *Toxoplasma* had a global prevalence of 48.85% (95% CI 42.01–55.69) in HIV-infected people in our study. This aligns closely with Safarpour et al.’s meta-analysis (44.22%, 95% CI 37.99–50.52%) [19] but contrasts with Wang et al.’s lower estimate (35.8%, 95% CI 30.8–40.7) [20], suggesting potential geographic or methodological variability.

Emerging evidence suggests that impaired immune activation, diminished production of antimalarial antibodies, and consequent immunosuppression are mechanistically linked to elevated rates of clinical malaria episodes and heightened parasitemia in HIV-infected populations, while malaria inflammation accelerates HIV/SIV-driven immunodeficiency, collectively worsening clinical outcomes [41,42]. For *Plasmodium* coinfection in HIV-infected people, our data revealed pronounced regional disparities: 34 studies from western and central Africa and 19 from eastern and southern Africa demonstrated higher prevalence in the former (38.36%, 95% CI 29.35–47.38) than the latter (31.22%, 95% CI 19.75–42.68), consistently with known malaria endemic burdens [43]. These findings underscore the urgent need for targeted interventions in high risk zones. Additionally, neglected kinetoplastid pathogens, *Leishmania* and *Trypanosoma*, exhibited notable prevalence in HIV-infected people, warranting further investigation into their clinical synergies with HIV.

Beyond HIV coinfections, our analysis incorporated epidemiological data (≥5 studies per case) on parasitic coinfections in individuals with HBV or DENV infections. In HBV-positive cohorts, we documented helminth (*Schistosoma mansoni*, *Ascaris lumbricoides*) and protozoan (*Plasmodium* spp., *Toxoplasma gondii*, *Leishmania donovani*) copathogens. We noted coinfections of HBV with helminths (*S. mansoni* and *A. lumbricoides*) and protozoa (*Plasmodium*, *T. gondii*, and *L. donovani*). The interaction between parasites (e.g., *S. mansoni* or *Plasmodium*) and HBV in a final host remains mechanistically obscure, hindered by the complex life cycles of parasitic organisms and the absence of appropriate animal models recapitulating these multiple-pathogen interactions [44,45,46]. Furthermore, the clinical implications of HBV–parasite coinfections, particularly their effects on hepatic pathogenesis and antiviral therapy efficacy, remain poorly characterized. For DENV, transmitted primarily by the *Aedes* mosquito, *Plasmodium* emerged as the sole parasitic copathogen in our meta-analysis. Given DENV’s hyperendemicity across more than 100 tropical/subtropical regions [47] and evidence suggesting exacerbated dengue severity in *Plasmodium*-coinfected patients [44,48], systematic surveillance of these coinfections is critical. Such efforts could refine differential diagnosis protocols, optimize therapeutic strategies, and inform vector control policies, particularly in ecoregions where overlapping *Anopheles* and *Aedes* habitats create niches for concurrent malaria–dengue transmission [49].

Our analysis revealed significant socioeconomic gradients in parasitic coinfection prevalence among people living with HIV, with the highest burdens concentrated in the eastern/southern Africa, western/central Africa, and Asia/Pacific regions, which strongly correlated with national income tiers. Despite global reductions in HIV–intestinal pathogen coinfections attributed to antiretroviral therapy (ART) [50], systemic disparities in healthcare access persist across Africa, perpetuating high intestinal parasite prevalence among PLWH in these settings [51,52]. Notably, while the Asia/Pacific region exhibited lower relative coinfection rates than the global average, its substantial HIV-positive population translated to elevated absolute disease burdens. This divergence underscores the dual challenge of region-specific transmission dynamics and resource allocation: African countries face urgent needs for expanded ART coverage, whereas Asia and the Pacific require targeted surveillance to mitigate population-scale impacts despite proportionally lower infection rates.

Our meta-analysis may have some limitations. First, persistent residual heterogeneity was observed across included datasets despite rigorous stratification by geospatial and demographic covariates (income, geographic region, country, gender, and age). This unresolved variability suggests either unreported confounders in source studies or methodological inconsistencies, particularly heterogeneity in diagnostic methodologies (e.g., varied pathogen detection assays) that may have differentially influenced reported prevalence rates. Second, our analysis was restricted to publications in English, excluding potentially relevant non-English evidence. This linguistic bias risk may distort regional prevalence estimates. Third, inherent surveillance capacity gaps for NTDs likely resulted in underrepresentation of epidemiological data from resource-limited settings. The cyclical neglect of parasite monitoring in endemic areas creates critical evidence voids, leaving their true coinfection burdens underrepresented in global health datasets. Fourth, our meta-analysis estimates regarding the pooled prevalence of helminth or protozoan coinfection in people living with (all) viruses are potentially limited by publication bias, which requires cautious interpretation because of probable inflation. This bias may originate from preferential publication of investigations with positive outcomes, particularly among small-scale studies; methodological heterogeneity of diagnosis; underreporting on NTDs; or language limitations. Notwithstanding the publication bias risk in the pooled helminth–virus and protozoan–virus coinfection studies, our stratified multidimensional subgroup analyses may attenuate the magnitude of the bias. Altogether, these intersecting limitations underscore the need for standardized diagnostics, multilingual systematic reviews, and strengthened parasitic disease surveillance infrastructure to improve future meta-analytic rigor.

## 5. Conclusions

Our study highlights disproportionate coinfection impacts in low- and middle-income countries, where intersecting biological vulnerabilities and healthcare disparities necessitate urgent investments in therapeutic infrastructure and drug accessibility. Geospatial analysis revealed striking regional inequity of markedly higher burdens of parasitic coinfection in virus-infected people in Africa and the Asia/Pacific region than in other regions, which underscores the imperative for WHO-aligned surveillance frameworks integrating multiplex diagnostics and geospatial mapping to target hyperendemic areas. To mitigate the impact of these coinfections, we recommend prophylaxis and vector control in parasite-endemic areas and accelerated deployment of vaccines against viral infections. Such interventions are crucial to protect those who are uninfected. Given the global nature of these health challenges, a coordinated international response is desired to fully scale up public health interventions and enhance the capabilities for unified diagnosis, prevention, and treatment of these infections. Our study therefore serves as both an epidemiological benchmark and a call to action to address these pressing health concerns collaboratively and effectively.

## Figures and Tables

**Table 1:** Pooled prevalence of helminth coinfections in virus-infected people.

	Studies	Coinfection (n/N)	Prevalence (95% CI)	tau^2^	Heterogeneity (I^2^)
Region					
Western and central Africa	27	1306/6878	20.65% (11.37–29.94)	0.0599	0.995
Middle East and North Africa	6	222/1527	36.07% (13.30–58.84)	0.0787	0.989
Eastern and southern Africa	43	2691/11,172	27.55% (21.72–33.37)	0.0368	0.991
Asia and the Pacific	34	757/6721	13.42% (7.51–19.33)	0.0301	0.972
Latin America and the Caribbean	6	395/1566	14.57% (0.00–34.33)	0.0606	0.995
Western and central Europe and North America	6	201/1204	17.60% (8.07–27.13)	0.0133	0.951
Sub-Saharan Africa *	1	21/122	17.21% (10.51–23.91)	N/A	N/A
Gender					
N/A	63	1429/14,135	19.90% (14.50–25.29)	0.0468	0.2163
Male	31	673/7741	24.41% (17.89–30.93)	0.0328	0.1812
Female	29	602/7314	21.13% (12.77–29.49)	0.0521	0.2283
Income Level					
High	8	209/1510	16.26% (8.25–24.27)	0.0126	0.1121
Upper middle	18	365/3438	22.85% (12.95–32.76)	0.0448	0.2117
Lower middle	71	1543/17,702	19.51% (14.63–24.39)	0.0430	0.2073
Low	26	484/6540	26.78% (17.58–35.98)	0.0565	0.2377
Sample size					
≤200	66	1722/7036	23.33% (17.91–28.75)	0.0490	0.989
>200	57	3871/22,154	19.13% (13.97–24.29)	0.0391	0.994
Total	123	2652/29,190	21.30% (17.60–25.10)	-	1.000

* Definitive country data is not available (N/A).

**Table 2:** Pooled prevalence of protozoan coinfections in virus-infected people.

	Studies	Coinfection (n/N)	Prevalence (95% CI)	tau^2^	Heterogeneity (I^2^)
Region					
Western and central Africa	91	7213/23,501	37.11% (31.46–42.77)	0.0740	0.999
Middle East and North Africa	32	1613/5092	39.01% (29.57–48.45)	0.0723	0.990
Eastern and southern Africa	71	4903/19,524	33.28% (27.35–39.22)	0.0639	0.993
Asia and the Pacific	70	4752/18,583	30.94% (25.54–36.34)	0.0518	0.995
Latin America and the Caribbean	31	1715/6480	30.68% (22.73–38.62)	0.0483	0.991
Western and central Europe and North America	11	1013/2805	29.60% (18.95–40.26)	0.0306	0.974
Eastern Europe and central Asia	1	19/46	41.30% (27.08–55.53)	N/A	N/A
Sub-Saharan Africa *	1	15/41	36.59% (21.84–51.33)	N/A	N/A
Gender					
Female	69	4346/16,069	33.14% (27.58–38.70)	0.0536	0.2314
Male	74	4551/22,222	31.37% (26.24–36.50)	0.0496	0.2226
N/A	165	7165/37,781	35.78% (31.66–39.89)	0.0719	0.2682
Income Level					
High	17	1146/4304	28.09% (19.66–36.51)	0.0296	0.1720
Upper middle	56	2452/15,594	27.66% (22.41–32.91)	0.0383	0.1958
Lower middle	182	9977/44,615	35.55% (31.76–39.34)	0.0665	0.2579
Low	53	2487/11,559	37.76% (30.25–45.27)	0.0762	0.2760
Sample size					
≤200	175	11,239/58,730	37.39% (33.61–41.16)	0.0625	0.2499
>200	133	4823/17,342	29.98% (25.85–34.12)	0.0587	0.2423
Total	208	16,062/76,072	31.41% (31.30–36.91)	-	1.000

* Definitive country data is not available (N/A).

**Table 3:** Estimates of helminth infections in HIV-infected people globally.

	Estimated HIV Infections	Prevalence of Coinfections	Estimated Number of Coinfections
	Number *	Mean (95% CI)	Estimated (95% CI)
Global	38,400,000	19.96% (16.18–23.74)	7,664,640 (6,213,120–9,116,160)
Region			
Western and central Africa	5,000,000	20.65% (11.37–29.94)	1,032,500 (568,500–1,497,000)
Cameroon	500,000	11.44% (0.00–23.11)	57,200 (0–115,550)
Congo	130,000	4.37% (0.12–8.61)	5681 (156–11,193)
Gabon	47,000	7.83% (2.92–12.73)	3680 (1372–5983)
Guinea	120,000	91.80% (89.47–94.12)	110,160 (107,364–112,944)
Nigeria	1,900,000	16.70% (9.95–23.46)	317,300 (189,050–445,740)
Middle East and North Africa	180,000	0.74% (0.24–1.24)	1332 (432–2232)
Iran	53,000	0.74% (0.24- 1.24)	392 (127–657)
Eastern and southern Africa	20,600,000	27.63% (21.66–33.59)	5,691,780 (4,461,960–6,919,540)
Ethiopia	610,000	18.86% (12.56–25.16)	115,046 (76,616–153,476)
Kenya	1,400,000	17.62% (2.50–32.74)	246,680 (35,000–458,360)
South Africa	7,500,000	39.70% (24.43–54.98)	2,977,500 (1,832,250–4,123,500)
Tanzania	1,700,000	31.86% (21.58–42.14)	541,620 (366,860–716,380)
Uganda	1,400,000	32.18% (25.74–38.62)	450,520 (360,360–540,680)
Zambia	1,300,000	36.44% (13.19–59.69)	473,720 (171,470–775,970)
Zimbabwe	1,300,000	47.83% (10.69–84.98)	621,790 (138,970–1,104,740)
Asia and the Pacific	6,000,000	12.02% (6.22–17.81)	721,200 (373,200–1,068,600)
Australia	30,000	22.39% (16.63–28.15)	6717 (4989–8445)
China	551,426	4.91% (2.74–7.08)	27,075 (15,109–39,040)
India	2,400,000	6.47% (4.36–8.59)	155,280 (104,640–206,160)
Indonesia	540,000	0.94% (0.00–2.01)	5076 (0–10,854)
Lao People’s Democratic Republic	15,000	59.59% (10.45–100.00)	8939 (1,568–15,000)
Korea	29,976	2.86% (0.00–6.04)	857 (0–1798)
Malaysia	82,000	19.08% (14.94–23.21)	15,646 (12,251–19,032)
Nepal	30,000	11.14% (8.88–13.40)	3342 (2664–4020)
Thailand	520,000	21.05% (0.00–47.11)	109,460 (0–244,972)
Latin America and the Caribbean	2,530,000	14.57% (0.00–34.33)	368,621 (0–868,549)
Brazil	960,000	20.92% (0.00–49.63)	200,832 (0–476,448)
Colombia	170,000	1.04% (0.00–2.48)	1768 (0–4216)
Honduras	22,000	2.94% (0.00–6.22)	647 (0–1368)
Western and central Europe and North America	2,300,000	17.60% (8.07–27.13)	404,800 (185,610–623,990)
Canada	92,282	22.56% (18.18–26.94)	20,819 (16,777–24,861)
France	190,000	7.55% (0.00–16.92)	14,345 (0–32,148)
Italy	140,000	10.87% (5.68–16.06)	15,218 (7952–22,484)
United State	1,200,000	37.50% (29.11–45.89)	450,000 (349,320–550,680)

* Data resources from WHO Database and Global Burden of Disease 2019.

**Table 4:** Estimates of protozoan infections in HIV, HBV, and DENV-infected people globally.

	Estimated HIV Infections	Prevalence of Coinfections	Estimated Number of Coinfections
	Number *	Mean (95% CI)	Estimated (95% CI)
Global	38,400,000	34.18% (31.33–37.03)	13,125,120 (12,030,720–14,219,520)
Region			
Western and central Africa	5,000,000	36·16% (30·48–41·84)	1808,000 (1,524,000–1,524,000)
Cameroon	500,000	37.82% (23.54–52.09)	189,100 (117,700–260,450)
Congo	130,000	36.77% (0.00–73.68)	47,801 (0–95,784)
Gabon	47,000	46.96% (37.84–56.08)	22,071 (17,785–26,358)
Ghana	350,000	25.00% (3.82–46.18)	87,500 (13,370–161,630)
Guinea	120,000	54.06% (0.00–100.00)	64,872 (0–120,000)
Nigeria	1,900,000	36.00% (29.06–42.95)	684,000 (552,140–816,050)
Middle East and North Africa	180,000	37.41% (28.20–46.62)	67,338 (50,760–83,916)
Iran	53,000	36.59% (27.22–45.97)	19,393 (14,427–24,364)
Morocco	23,000	62.11% (52.35–71.86)	14,285 (12,041–16,528)
Eastern and southern Africa	20,600,000	34.26% (28.16–40.35)	7,057,560 (5,800,960–8,312,100)
Ethiopia	610,000	36.66% (26.73–46.59)	223,626 (163,053–284,199)
Kenya	1,400,000	38.08% (23.05–53.12)	533,120 (322,700–743,680)
Malawi	990,000	33.06% (7.73–58.38)	327,294 (76,527–577,962)
Mozambique	2,355,345	26.83% (12.09–41.57)	631,939 (284,761–979,116)
South Africa	7,500,000	19.77% (13.67–25.87)	1,482,750 (1,025,250–1,940,250)
Tanzania	1,700,000	17.67% (12.09–23.26)	300,390 (205,530–395,420)
Uganda	1,400,000	46.14% (14.35–77.93)	645,960 (200,900–1,091,020)
Zambia	1,300,000	76.36% (65.14–87.59)	992,680 (846,820–1,138,670)
Zimbabwe	1,300,000	72.41% (56.15–88.68)	941,330 (729,950–1,152,840)
Asia and the Pacific	6,000,000	32.09% (26.52–37.67)	1,925,400 (1,591,200–2,260,200)
Australia	30,000	45.97% (42.61–49.33)	13,791 (12,783–14,799)
China	551,426	12.82% (5.09–20.54)	70,692 (28,068–113,262)
India	2,400,000	32.02% (24.55–39.49)	768,480 (589,200–947,760)
Indonesia	540,000	58.53% (30.00–87.06)	316,062 (162,000–470,124)
Japan	45,514	8.27% (5.57–10.97)	5835 (2317–9349)
Korea	29,976	12.38% (6.08–18.68)	3711 (1823–5600)
Lao People’s Democratic Republic	15,000	64.96% (56.97–72.95)	9744 (8546–10,943)
Malaysia	82,000	38.18% (16.83–59.52)	31,308 (13,801–48,806)
Nepal	30,000	20.36% (1.89–38.83)	6108 (567–11,649)
Thailand	520,000	37.05% (20.94–53.17)	192,660 (108,888–276,484)
Latin America and the Caribbean	2,530,000	31.51% (23.44–39.57)	797,203 (593,032–1,001,121)
Bolivia	26,000	54.12% (2.30–100.00)	14,071 (598–26,000)
Brazil	960,000	31.88% (21.29–42.48)	306,048 (204,384–407,808)
Colombia	170,000	27.20% (22.35–32.05)	46,240 (37,995–54,485)
Honduras	22,000	30.39% (21.47–39.32)	6686 (4723–8650)
Mexico	360,000	44.01% (18.87–69.14)	158,436 (67,932–248,904)
Spain	160,000	14.23% (0.00–31.05)	22,768 (0–49,680)
Venezuela	98,000	14.86% (11.36–18.36)	14,563 (11,133–17,993)
Western and central Europe and North America	2,300,000	27.47% (16.64–38.29)	631,810 (382,720–880,670)
Czech Republic		41.37% (39.13–43.61)	
Denmark	6700	33.33% (23.90–42.76)	2233 (1601–2865)
France	190,000	12.60% (8.48–16.72)	23940 (16,112–31,768)
Italy	140,000	16.55% (10.50–22.60)	23,170 (14,700–31,640)
Turkey	5281	5.22% (1.15–9.28)	276 (62–490)
United State	1,200,000	38.84% (12.85–64.82)	466,080 (154,200–777,840)
Eastern Europe and central Asia			
Russia	1,137,793	41.30% (27.08–55.53)	469,909,(308,114–631,816)
	Estimated DENV infections	Prevalence of coinfections	Estimated number of coinfections
	Number *	Mean (95% CI)	Estimated (95% CI)
Global	3,394,136	17.75% (3.54–31.95)	629,952 (142,893–1,086,124)
	Estimated HBV infections	Prevalence of coinfections	Estimated number of coinfections
	Number *	Mean (95% CI)	Estimated (95% CI)
Global	326,236,734	41.79% (15.88–67.69)	137,019,428 (52,197,877–221,840,979)

* Data resources from WHO Database and Global Burden of Disease 2019.

## Supplementary Materials

The following supporting information can be downloaded at: https://www.mdpi.com/article/10.3390/pathogens14060534/s1, Figure S1A: Forest plots of the prevalence rates of helminth coinfections in virus-infected people at the family level by meta-analysis using common-effect and random-effects models; Figure S1B: Forest plots of the prevalence rates of protozoan coinfections in virus-infected people at the family level by meta-analysis using common-effect and random-effects models; Figure S2A: Forest plots of the prevalence rates of helminth coinfections in virus-infected people at the genus level by meta-analysis using common-effect and random-effects models; Figure S2B: Forest plots of the prevalence rates of protozoan coinfections in virus-infected people at the genus level by meta-analysis using common-effect and random-effects models; Figure S3A: Forest plots of the prevalence rates of helminth coinfections in virus-infected people at the species level by meta-analysis using common-effect and random-effects models; Figure S3B: Forest plots of the prevalence rates of protozoan coinfections in virus-infected people at the species level by meta-analysis using common-effect and random-effects models; Figure S4A: Forest plots of the prevalence rates of helminth coinfections in HIV-infected people at the genus level by meta-analysis using common-effect and random-effects models; Figure S4B: Forest plots of the prevalence rates of protozoan coinfections in HIV-infected people at the genus level by meta-analysis using common-effect and random-effects models; Figure S5A: Subgroup analysis of the prevalence of helminth infections in people living with viruses by age; Figure S5B: Subgroup analysis of the prevalence of protozoan infections in people living with viruses by age; Figure S6A: Subgroup analysis of the prevalence of helminth infections in people living with viruses by gender; Figure S6B: Subgroup analysis of the prevalence of protozoan infections in people living with viruses by gender; Figure S7A: Subgroup analysis of the prevalence of helminth infections in people living with viruses by publication year; Figure S7B: Subgroup analysis of the prevalence of protozoan infections in people living with viruses by publication year; Figure S8A: Subgroup analysis of the prevalence of helminth infections in people living with HIV by income level; Figure S8B: Subgroup analysis of the prevalence of protozoan infections in people living with HIV by income level; Figure S9A: Subgroup analysis of the prevalence of helminth infections in people living with HIV by age; Figure S9B: Subgroup analysis of the prevalence of protozoan infections in people living with HIV by age; Figure S10A: Subgroup analysis of the prevalence of helminth infections in people living with HIV by gender; Figure S10B: Subgroup analysis of the prevalence of protozoan infections in people living with HIV by gender; Figure S11A: Subgroup analysis of the prevalence of helminth infections in people living with HIV by sample size; Figure S11B: Subgroup analysis of the prevalence of protozoan infections in people living with HIV by sample size; Figure S12A: Subgroup analysis of the prevalence of helminth infections in people living with HIV by publication year; Figure S12B: Subgroup analysis of the prevalence of protozoan infections in people living with HIV by publication year; Figure S13A: Funnel plot assessing distribution symmetry for the pooled prevalence of helminth coinfection in people living with viruses from 135 studies; Figure S13B: Funnel plot assessing distribution symmetry for the pooled prevalence of protozoan coinfection in people living with viruses from 323 studies; Table S1: PRISMA Checklist; Table S2: Included studies on helminth coinfections in virus-infected people; Table S3: Included studies on protozoan coinfections in virus-infected people; Table S4: The prevalence rates of parasitic coinfections at genus level in HIV-infected people by meta-analysis; Text S1: Search strategy and rules for study selection; Text S2: Quality assessment of included studies; Text S3: Egger’s linear regression test and Begg–Mazumdar rank correlation test on the pooled prevalence of helminth in virus-infected people; Text S4: Egger’s linear regression test and Begg–Mazumdar rank correlation test on the pooled prevalence of protozoan in virus-infected people [53,54,55,56,57,58,59,60,61,62,63,64,65,66,67,68,69,70,71,72,73,74,75,76,77,78,79,80,81,82,83,84,85,86,87,88,89,90,91,92,93,94,95,96,97,98,99,100,101,102,103,104,105,106,107,108,109,110,111,112,113,114,115,116,117,118,119,120,121,122,123,124,125,126,127,128,129,130,131,132,133,134,135,136,137,138,139,140,141,142,143,144,145,146,147,148,149,150,151,152,153,154,155,156,157,158,159,160,161,162,163,164,165,166,167,168,169,170,171,172,173,174,175,176,177,178,179,180,181,182,183,184,185,186,187,188,189,190,191,192,193,194,195,196,197,198,199,200,201,202,203,204,205,206,207,208,209,210,211,212,213,214,215,216,217,218,219,220,221,222,223,224,225,226,227,228,229,230,231,232,233,234,235,236,237,238,239,240,241,242,243,244,245,246,247,248,249,250,251,252,253,254,255,256,257,258,259,260,261,262,263,264,265,266,267,268,269,270,271,272,273,274,275,276,277,278,279,280,281,282,283,284,285,286,287,288,289,290,291,292,293,294,295,296,297,298,299,300,301,302,303,304,305,306,307,308,309,310,311,312,313,314,315,316,317,318,319,320,321,322,323,324,325,326,327,328,329,330,331,332,333,334,335,336,337,338,339,340,341,342,343,344,345,346,347,348,349,350,351,352,353,354,355,356,357,358,359,360,361,362,363,364,365,366,367,368,369,370,371,372,373,374,375,376,377,378,379,380,381,382,383,384,385,386,387,388,389,390,391,392,393,394,395,396].
