# Supplementary material for: The Global Prevalence of and Factors Associated with Parasitic Coinfection in People Living with Viruses: A Systematic Review and Meta-Analysis
Source: Pathogens. 2025 May 27;14(6):534. doi: 10.3390/pathogens14060534 (PMC12195843; doi:10.3390/pathogens14060534)
Supplement: Supplementary file 1 [file pathogens-3593236-supplementary.pdf › pathogens-3593236-supplementary.pdf]

Figure S1A: Forest plots of the prevalence rates of helminth co-infections in virus-infected people at family level by meta-analysis using common effect and random-effects models

Study	Events	Total	Proportion	95%-CI	Weight (common)	Weight (random)
**Helminth_Family = Ascarididae**						
Abaver et al., 2011	1	85	0.01	[0.00; 0.06]	0.1%	0.3%
Abilo and Afework, 2005	6	70	0.09	[0.03; 0.18]	0.0%	0.2%
Abossie and Petros, 2015	42	97	0.43	[0.33; 0.54]	0.0%	0.2%
Adams et al., 2006	86	170	0.51	[0.43; 0.58]	0.0%	0.2%
Adamu and Petros, 2009	5	200	0.03	[0.01; 0.06]	0.1%	0.3%
Adeleke et al., 2015	24	231	0.10	[0.07; 0.15]	0.0%	0.2%
Adesij et al. 2007	9	100	0.09	[0.04; 0.16]	0.0%	0.2%
Ahmed and Abhay, 2015	5	142	0.04	[0.01; 0.08]	0.0%	0.3%
Akinbo and Omoregie, 2011	12	285	0.04	[0.02; 0.07]	0.1%	0.3%
Akinbo et al., 2011	67	463	0.14	[0.11; 0.18]	0.0%	0.3%
Akinbo et al., 2012	79	2000	0.04	[0.03; 0.05]	0.4%	0.3%
Asma et al., 2011	48	346	0.14	[0.10; 0.18]	0.0%	0.2%
Assefa et al., 2009	26	214	0.12	[0.08; 0.17]	0.0%	0.2%
Assefa et al., 2013	7	272	0.03	[0.01; 0.05]	0.1%	0.3%
Babatunde et al., 2010	6	90	0.07	[0.02; 0.14]	0.0%	0.2%
Dash et al., 2013	2	115	0.02	[0.00; 0.06]	0.1%	0.3%
Eshetu et al., 2017	15	223	0.07	[0.04; 0.11]	0.0%	0.3%
Fekadu et al., 2013	36	343	0.10	[0.07; 0.14]	0.0%	0.3%
Gaurav and Atul, 2015	1	40	0.03	[0.00; 0.13]	0.0%	0.2%
Getaneh et al., 2018	4	220	0.02	[0.00; 0.05]	0.1%	0.3%
Gebretsadik et al., 2018	3	223	0.01	[0.00; 0.04]	0.1%	0.3%
Gupta et al., 2013	1	105	0.01	[0.00; 0.05]	0.1%	0.3%
Haileeyesus et al., 2013	11	378	0.03	[0.01; 0.05]	0.1%	0.3%
Idindili et al., 2011	54	421	0.13	[0.10; 0.16]	0.0%	0.3%
Kulkarni et al., 2009	4	137	0.03	[0.01; 0.07]	0.0%	0.3%
Kawai et al., 2009	46	971	0.05	[0.03; 0.06]	0.2%	0.3%
Kipyegen et al., 2012	13	285	0.05	[0.02; 0.08]	0.1%	0.3%
Kurniawan et al., 2009	2	318	0.01	[0.00; 0.02]	0.4%	0.3%
Lar et al., 2016	16	205	0.08	[0.05; 0.12]	0.0%	0.2%
Missaye et al., 2013	7	272	0.03	[0.01; 0.05]	0.1%	0.3%
Mkhize et al., 2017	25	95	0.26	[0.18; 0.36]	0.0%	0.2%
Modjarrad et al., 2005	39	297	0.13	[0.10; 0.18]	0.0%	0.2%
Modjarrad et al., 2005	26	111	0.23	[0.16; 0.32]	0.0%	0.2%
Mulu et al., 2013	24	220	0.11	[0.07; 0.16]	0.0%	0.2%
Nsagha et al., 2016	13	300	0.04	[0.02; 0.07]	0.1%	0.3%
Ojurongbe et al., 2011	9	96	0.09	[0.04; 0.17]	0.0%	0.2%
Orji et al., 2017	9	84	0.11	[0.05; 0.19]	0.0%	0.2%
Oyedeji et al., 2015	1	52	0.02	[0.00; 0.10]	0.0%	0.2%
Paboriboune et al., 2014	1	137	0.01	[0.00; 0.04]	0.2%	0.3%
Pennap et al., 2015	27	200	0.14	[0.09; 0.19]	0.0%	0.2%
Ramakrishnan et al., 2007	6	80	0.07	[0.03; 0.16]	0.0%	0.2%
Ramana et al., 2010	13	452	0.03	[0.02; 0.05]	0.1%	0.3%
Ringo et al., 2015	13	83	0.16	[0.09; 0.25]	0.0%	0.2%
Rodrigues et al., 2008	77	582	0.13	[0.11; 0.16]	0.0%	0.3%
Roka et al., 2012	50	260	0.19	[0.15; 0.25]	0.0%	0.2%
Roka et al., 2013	63	273	0.23	[0.18; 0.29]	0.0%	0.2%
Samie et al., 2014	12	151	0.08	[0.04; 0.13]	0.0%	0.2%
Sanyaolu et al., 2011	5	65	0.08	[0.03; 0.17]	0.0%	0.2%
Sherpa et al., 2010	4	106	0.04	[0.01; 0.09]	0.0%	0.2%
Shimelis et al., 2016	22	491	0.04	[0.03; 0.07]	0.1%	0.3%
Suryawanshi et al., 2012	5	100	0.05	[0.02; 0.11]	0.0%	0.2%
Tegegne et al., 2017	15	223	0.07	[0.04; 0.11]	0.0%	0.3%
Tigist et al., 2015	11	209	0.05	[0.03; 0.09]	0.0%	0.3%
Tiwari et al., 2013	20	745	0.03	[0.02; 0.04]	0.2%	0.3%
Vouking et al., 2014	27	207	0.13	[0.09; 0.18]	0.0%	0.2%
Vyas et al., 2013	2	75	0.03	[0.00; 0.09]	0.0%	0.2%
Wanyiri et al., 2013	22	164	0.13	[0.09; 0.20]	0.0%	0.2%
Wumba et al., 2010	7	175	0.04	[0.02; 0.08]	0.0%	0.3%
Wumba et al., 2012	5	242	0.02	[0.01; 0.05]	0.1%	0.3%
Xiao et al., 2015	12	40	0.30	[0.17; 0.47]	0.0%	0.2%
Zeynudin et al., 2013	13	91	0.14	[0.08; 0.23]	0.0%	0.2%
Gebrecherkos et al., 2019	6	150	0.04	[0.01; 0.09]	0.0%	0.3%
Sannella et al., 2019	1	155	0.01	[0.00; 0.04]	0.2%	0.3%
Alemayehu etal., 2020	23	383	0.06	[0.04; 0.09]	0.1%	0.3%
Chhangte et al., 2020	6	250	0.02	[0.01; 0.05]	0.1%	0.3%
Lengongo et al., 2020	1	115	0.01	[0.00; 0.05]	0.1%	0.3%
Namaji et al., 2020	7	361	0.02	[0.01; 0.04]	0.2%	0.3%
Rabiu et al., 2020	6	197	0.03	[0.01; 0.07]	0.1%	0.3%
Bayleyegn et al., 2021	22	255	0.09	[0.05; 0.13]	0.0%	0.3%
Betancourth et al., 2021	2	102	0.02	[0.00; 0.07]	0.0%	0.3%
Feleke et al., 2022	4	223	0.02	[0.00; 0.05]	0.1%	0.3%
Ajayi et al., 2021	4	375	0.01	[0.00; 0.03]	0.3%	0.3%
Wolday et al., 2021	27	751	0.04	[0.02; 0.05]	0.2%	0.3%
Purbey., 2021	5	75	0.07	[0.02; 0.15]	0.0%	0.2%
Uzairue et al., 2021	16	170	0.09	[0.05; 0.15]	0.0%	0.2%
PrayGod et al., 2022	16	1149	0.01	[0.01; 0.02]	0.7%	0.3%
Common effect model		20138	0.03	[0.03; 0.03]	5.6%	--
Random effects model			0.08	[0.06; 0.09]	--	18.8%
Heterogeneity: $I^2 = 92\%$, $\tau^2 = 0.0056$, $p < 0.01$						
**Helminth_Family = Ancylostomatidae**						
Abaver et al., 2011	3	85	0.04	[0.01; 0.10]	0.0%	0.2%
Abossie and Petros, 2015	8	97	0.08	[0.04; 0.16]	0.0%	0.2%
Adeleke et al., 2015	3	231	0.01	[0.00; 0.04]	0.2%	0.3%
Adesij et al. 2007	2	100	0.02	[0.00; 0.07]	0.0%	0.3%
Ahmed and Abhay, 2015	2	142	0.01	[0.00; 0.05]	0.1%	0.3%
Akinbo and Omoregie, 2011	2	285	0.01	[0.00; 0.03]	0.3%	0.3%
Akinbo et al., 2011	29	463	0.06	[0.04; 0.09]	0.1%	0.3%
Akinbo et al., 2012	50	2000	0.03	[0.02; 0.03]	0.7%	0.3%
Asma et al., 2011	2	346	0.01	[0.00; 0.02]	0.5%	0.3%
Assefa et al., 2009	7	214	0.03	[0.01; 0.07]	0.1%	0.3%
Babatunde et al., 2010	7	90	0.08	[0.03; 0.15]	0.0%	0.2%
Cardoso et al., 2011	2	500	0.00	[0.00; 0.01]	1.1%	0.3%
Da Silva et al., 2005	4	100	0.04	[0.01; 0.10]	0.0%	0.2%
Dash et al., 2013	1	115	0.01	[0.00; 0.05]	0.1%	0.3%
Eshetu et al., 2017	3	223	0.01	[0.00; 0.04]	0.1%	0.3%
Fekadu et al., 2013	9	343	0.03	[0.01; 0.05]	0.1%	0.3%
Feyisayo et al., 2014	4	105	0.04	[0.01; 0.09]	0.0%	0.2%
Gaurav and Atul, 2015	2	40	0.05	[0.01; 0.17]	0.0%	0.2%
Getaneh et al., 2018	2	220	0.01	[0.00; 0.03]	0.2%	0.3%
Haileeyesus et al., 2013	2	378	0.01	[0.00; 0.02]	0.6%	0.3%
Idindili et al., 2011	65	421	0.15	[0.12; 0.19]	0.0%	0.3%
Jegede et al., 2014	4	105	0.04	[0.01; 0.09]	0.0%	0.2%
Kulkarni et al., 2009	3	137	0.02	[0.00; 0.06]	0.1%	0.3%
Kawai et al., 2009	96	971	0.10	[0.08; 0.12]	0.1%	0.3%
Kipyegen et al., 2012	2	285	0.01	[0.00; 0.03]	0.3%	0.3%
Lar et al., 2016	23	205	0.11	[0.07; 0.16]	0.0%	0.2%
Mathur et al., 2013	34	544	0.06	[0.04; 0.09]	0.1%	0.3%
Minti et al., 2017	1	27	0.04	[0.00; 0.19]	0.0%	0.2%
Modjarrad et al., 2005	23	297	0.08	[0.05; 0.11]	0.0%	0.3%
Modjarrad et al., 2005	18	111	0.16	[0.10; 0.24]	0.0%	0.2%
Morawski et al., 2017	56	202	0.28	[0.22; 0.34]	0.0%	0.2%
Mulu et al., 2013	12	220	0.05	[0.03; 0.09]	0.0%	0.3%
Nsagha et al., 2016	8	300	0.03	[0.01; 0.05]	0.1%	0.3%
Nuhu et al., 2014	6	205	0.03	[0.01; 0.06]	0.1%	0.3%
Ojurongbe et al., 2011	5	96	0.05	[0.02; 0.12]	0.0%	0.2%
Orji et al., 2017	12	84	0.14	[0.08; 0.24]	0.0%	0.2%
Paboriboune et al., 2014	19	137	0.14	[0.09; 0.21]	0.0%	0.2%
Pavie et al., 2012	2	143	0.01	[0.00; 0.05]	0.1%	0.3%
Pennap et al., 2015	22	200	0.11	[0.07; 0.16]	0.0%	0.2%
Pinlaor et al., 2005	3	78	0.04	[0.01; 0.11]	0.0%	0.2%
Ramana et al., 2010	4	452	0.01	[0.00; 0.02]	0.4%	0.3%
Rodrigues et al., 2008	68	582	0.12	[0.09; 0.15]	0.0%	0.3%
Roka et al., 2012	11	260	0.04	[0.02; 0.07]	0.1%	0.3%
Roka et al., 2013	11	273	0.04	[0.02; 0.07]	0.1%	0.3%
Samie et al., 2014	4	151	0.03	[0.01; 0.07]	0.0%	0.3%
Sanyaolu et al., 2011	3	65	0.05	[0.01; 0.13]	0.0%	0.2%
Suryawanshi et al., 2012	3	100	0.03	[0.01; 0.09]	0.0%	0.3%
Tegegne et al., 2017	3	223	0.01	[0.00; 0.04]	0.1%	0.3%
Tian et al., 2012	11	302	0.04	[0.02; 0.06]	0.1%	0.3%
Tian et al., 2013	5	79	0.06	[0.02; 0.14]	0.0%	0.2%
Tigist et al., 2015	8	209	0.04	[0.02; 0.07]	0.0%	0.3%
Tiwari et al., 2013	19	745	0.03	[0.02; 0.04]	0.3%	0.3%
Vouking et al., 2014	6	207	0.03	[0.01; 0.06]	0.1%	0.3%
Wanyiri et al., 2013	6	164	0.04	[0.01; 0.08]	0.0%	0.3%
Wumba et al., 2010	2	175	0.01	[0.00; 0.04]	0.1%	0.3%
Gebrecherkos et al., 2019	2	150	0.01	[0.00; 0.05]	0.1%	0.3%
Kaneshiro et al., 2019	22	252	0.09	[0.06; 0.13]	0.0%	0.3%
Sannella et al., 2019	2	155	0.01	[0.00; 0.05]	0.1%	0.3%
Alemayehu et al., 2020	4	383	0.01	[0.00; 0.03]	0.3%	0.3%
Namaji et al., 2020	12	361	0.03	[0.02; 0.06]	0.1%	0.3%
Bayleyegn et al., 2021	3	255	0.01	[0.00; 0.03]	0.2%	0.3%
Feleke et al., 2022	3	223	0.01	[0.00; 0.04]	0.1%	0.3%
Ajayi et al., 2021	6	375	0.02	[0.01; 0.03]	0.2%	0.3%
Wolday et al., 2021	12	751	0.02	[0.01; 0.03]	0.4%	0.3%
Purbey., 2021	7	75	0.09	[0.04; 0.18]	0.0%	0.2%
Uzairue et al., 2021	17	170	0.10	[0.06; 0.16]	0.0%	0.2%
PrayGod et al., 2022	66	1149	0.06	[0.04; 0.07]	0.2%	0.3%
Common effect model		19131	0.02	[0.02; 0.02]	8.5%	--
Random effects model			0.04	[0.03; 0.05]	--	16.8%
Heterogeneity: $I^2 = 88\%$, $\tau^2 = 0.0014$, $p < 0.01$						
**Helminth_Family = Strongylidae**						
Abaver et al., 2011	3	85	0.04	[0.01; 0.10]	0.0%	0.2%
Abilo and Afework, 2005	12	70	0.17	[0.09; 0.28]	0.0%	0.2%
Abossie and Petros, 2015	3	97	0.03	[0.01; 0.09]	0.0%	0.3%
Adeleke et al., 2015	3	231	0.01	[0.00; 0.04]	0.2%	0.3%
Ahmed and Abhay, 2015	5	142	0.04	[0.01; 0.08]	0.0%	0.3%
Akinbo and Omoregie, 2011	1	285	0.00	[0.00; 0.02]	0.7%	0.3%
Akinbo et al., 2011	14	463	0.03	[0.02; 0.05]	0.1%	0.3%
Akinbo et al., 2012	15	2000	0.01	[0.00; 0.01]	2.3%	0.3%
Assefa et al., 2009	27	214	0.13	[0.08; 0.18]	0.0%	0.2%
Assefa et al., 2013	2	272	0.01	[0.00; 0.03]	0.3%	0.3%
Babatunde et al., 2010	17	90	0.19	[0.11; 0.29]	0.0%	0.2%
Barcelos et al., 2018	1	90	0.01	[0.00; 0.06]	0.1%	0.3%
Cardoso et al., 2011	1	500	0.00	[0.00; 0.01]	2.1%	0.3%
Costiniuk et al., 2012	10	97	0.10	[0.05; 0.18]	0.0%	0.2%
Da Silva et al., 2005	12	100	0.12	[0.06; 0.20]	0.0%	0.2%
Dash et al., 2013	3	115	0.03	[0.01; 0.07]	0.0%	0.3%
Eshetu et al., 2017	8	223	0.04	[0.02; 0.07]	0.1%	0.3%
Fekadu et al., 2013	56	343	0.16	[0.13; 0.21]	0.0%	0.2%
Gaurav and Atul, 2015	3	40	0.07	[0.02; 0.20]	0.0%	0.2%
Getaneh et al., 2018	4	220	0.02	[0.00; 0.05]	0.1%	0.3%
Gebretsadik et al., 2018	1	223	0.00	[0.00; 0.02]	0.4%	0.3%
Gupta et al., 2013	1	105	0.01	[0.00; 0.05]	0.1%	0.3%
Haileeyesus et al., 2013	10	378	0.03	[0.01; 0.05]	0.1%	0.3%
Hochberg et al., 2011	33	128	0.26	[0.18; 0.34]	0.0%	0.2%
Idindili et al., 2011	57	421	0.14	[0.10; 0.17]	0.0%	0.3%
Kawai et al., 2009	13	971	0.01	[0.01; 0.02]	0.6%	0.3%
Kurniawan et al., 2009	1	318	0.00	[0.00; 0.02]	0.9%	0.3%
Lehman et al., 2013	2	201	0.01	[0.00; 0.04]	0.2%	0.3%
Mascarello et al., 2011	15	138	0.11	[0.06; 0.17]	0.0%	0.2%
Meamar et al., 2007	2	781	0.00	[0.00; 0.01]	2.6%	0.3%
Mehta et al., 2013	3	100	0.03	[0.01; 0.09]	0.0%	0.3%
Missaye et al., 2013	2	272	0.01	[0.00; 0.03]	0.3%	0.3%
Modjarrad et al., 2005	3	297	0.01	[0.00; 0.03]	0.2%	0.3%
Modjarrad et al., 2005	4	111	0.04	[0.01; 0.09]	0.0%	0.3%
Morawski et al., 2017	8	202	0.04	[0.02; 0.08]	0.0%	0.3%
Nsagha et al., 2016	2	300	0.01	[0.00; 0.02]	0.4%	0.3%
Ojurongbe et al., 2011	1	96	0.01	[0.00; 0.06]	0.1%	0.3%
Orji et al., 2017	1	84	0.01	[0.00; 0.06]	0.1%	0.3%
Paboriboune et al., 2014	28	137	0.20	[0.14; 0.28]	0.0%	0.2%
Pavie et al., 2012	2	143	0.01	[0.00; 0.05]	0.1%	0.3%
Pennap et al., 2015	26	200	0.13	[0.09; 0.18]	0.0%	0.2%
Pinlaor et al., 2005	14	78	0.18	[0.10; 0.28]	0.0%	0.2%
Plumelle et al., 2015	55	445	0.12	[0.09; 0.16]	0.0%	0.3%
Ramana et al., 2010	1	452	0.00	[0.00; 0.01]	1.7%	0.3%
Rodrigues et al., 2008	156	582	0.27	[0.23; 0.31]	0.0%	0.3%
Roka et al., 2012	23	260	0.09	[0.06; 0.13]	0.0%	0.3%
Roka et al., 2013	28	273	0.10	[0.07; 0.14]	0.0%	0.3%
Sanyaolu et al., 2011	1	65	0.02	[0.00; 0.08]	0.0%	0.3%
Shimelis et al., 2016	22	491	0.04	[0.03; 0.07]	0.1%	0.3%
Suryawanshi et al., 2012	2	100	0.02	[0.00; 0.07]	0.0%	0.3%
Tegegne et al., 2017	8	223	0.04	[0.02; 0.07]	0.1%	0.3%
Tigist et al., 2015	30	209	0.14	[0.10; 0.20]	0.0%	0.2%
Tiwari et al., 2013	2	745	0.00	[0.00; 0.01]	2.3%	0.3%
Vouking et al., 2014	7	207	0.03	[0.01; 0.07]	0.1%	0.3%
Wanyiri et al., 2013	8	164	0.05	[0.02; 0.09]	0.0%	0.3%
Zeynudin et al., 2013	6	91	0.07	[0.02; 0.14]	0.0%	0.2%
Gebrecherkos et al., 2019	16	150	0.11	[0.06; 0.17]	0.0%	0.2%
Kaneshiro et al., 2019	23	252	0.09	[0.06; 0.13]	0.0%	0.3%
Sannella et al., 2019	14	155	0.09	[0.05; 0.15]	0.0%	0.2%
Alemayehu et al., 2020	15	383	0.04	[0.02; 0.06]	0.1%	0.3%
Chhangte et al., 2020	1	250	0.00	[0.00; 0.02]	0.5%	0.3%
Lengongo et al., 2020	7	115	0.06	[0.02; 0.12]	0.0%	0.2%
McLellan et al., 2020	11	253	0.04	[0.02; 0.08]	0.1%	0.3%
Botero et al., 2021	1	192	0.01	[0.00; 0.03]	0.3%	0.3%
Feleke et al., 2022	3	223	0.01	[0.00; 0.04]	0.1%	0.3%
Talukder et al., 2021	45	201	0.22	[0.17; 0.29]	0.0%	0.2%
Purbey., 2021	1	75	0.01	[0.00; 0.07]	0.0%	0.3%
Uzairue et al., 2021	7	170	0.04	[0.02; 0.08]	0.0%	0.3%
PrayGod et al., 2022	15	1149	0.01	[0.01; 0.02]	0.7%	0.3%
Common effect model		19236	0.01	[0.01; 0.01]	18.8%	--
Random effects model			0.06	[0.04; 0.07]	--	17.3%
Heterogeneity: $I^2 = 93\%$, $\tau^2 = 0.0035$, $p < 0.01$						
**Helminth_Family = Trichuridae**						
Abaver et al., 2011	1	85	0.01	[0.00; 0.06]	0.1%	0.3%
Adeleke et al., 2015	3	231	0.01	[0.00; 0.04]	0.2%	0.3%
Adesij et al. 2007	1	100	0.01	[0.00; 0.05]	0.1%	0.3%
Ahmed and Abhay, 2015	1	142	0.01	[0.00; 0.04]	0.2%	0.3%
Akinbo et al., 2011	11	463	0.02	[0.01; 0.04]	0.2%	0.3%
Akinbo et al., 2012	8	2000	0.00	[0.00; 0.01]	4.2%	0.3%
Asma et al., 2011	22	346	0.06	[0.04; 0.09]	0.0%	0.3%
Assefa et al., 2009	8	214	0.04	[0.02; 0.07]	0.0%	0.3%
Assefa et al., 2013	2	272	0.01	[0.00; 0.03]	0.3%	0.3%
Babatunde et al., 2010	9	90	0.10	[0.05; 0.18]	0.0%	0.2%
Da Silva et al., 2005	1	100	0.01	[0.00; 0.05]	0.1%	0.3%
Eshetu et al., 2017	3	223	0.01	[0.00; 0.04]	0.1%	0.3%
Fekadu et al., 2013	3	343	0.01	[0.00; 0.03]	0.3%	0.3%
Haileeyesus et al., 2013	8	378	0.02	[0.01; 0.04]	0.2%	0.3%
Kawai et al., 2009	9	971	0.01	[0.00; 0.02]	0.9%	0.3%
Kipyegen et al., 2012	3	285	0.01	[0.00; 0.03]	0.2%	0.3%
Lar et al., 2016	8	205	0.04	[0.02; 0.08]	0.0%	0.3%
Lehman et al., 2013	1	201	0.00	[0.00; 0.03]	0.3%	0.3%
Missaye et al., 2013	2	272	0.01	[0.00; 0.03]	0.3%	0.3%
Mkhize et al., 2017	6	95	0.06	[0.02; 0.13]	0.0%	0.2%
Morawski et al., 2017	1	202	0.00	[0.00; 0.03]	0.3%	0.3%
Mulu et al., 2013	14	220	0.06	[0.04; 0.10]	0.0%	0.3%
Nsagha et al., 2016	7	300	0.02	[0.01; 0.05]	0.1%	0.3%
Orji et al., 2017	2	84	0.02	[0.00; 0.08]	0.0%	0.3%
Paboriboune et al., 2014	3	137	0.02	[0.00; 0.06]	0.1%	0.3%
Pennap et al., 2015	3	200	0.01	[0.00; 0.04]	0.1%	0.3%
Rodrigues et al., 2008	64	582	0.11	[0.09; 0.14]	0.0%	0.3%
Roka et al., 2012	134	260	0.52	[0.45; 0.58]	0.0%	0.2%
Roka et al., 2013	124	273	0.45	[0.39; 0.52]	0.0%	0.2%
Samie et al., 2014	7	151	0.05	[0.02; 0.09]	0.0%	0.3%
Sanyaolu et al., 2011	3	65	0.05	[0.01; 0.13]	0.0%	0.2%
Shimelis et al., 2016	5	491	0.01	[0.00; 0.02]	0.4%	0.3%
Tegegne et al., 2017	3	223	0.01	[0.00; 0.04]	0.1%	0.3%
Tian et al., 2012	2	302	0.01	[0.00; 0.02]	0.4%	0.3%
Tigist et al., 2015	3	209	0.01	[0.00; 0.04]	0.1%	0.3%
Tiwari et al., 2013	35	745	0.05	[0.03; 0.06]	0.1%	0.3%
Vouking et al., 2014	9	207	0.04	[0.02; 0.08]	0.0%	0.3%
Wumba et al., 2010	1	175	0.01	[0.00; 0.03]	0.3%	0.3%
Wumba et al., 2012	1	242	0.00	[0.00; 0.02]	0.5%	0.3%
Xiao et al., 2015	10	40	0.25	[0.13; 0.41]	0.0%	0.2%
Zeynudin et al., 2013	4	91	0.04	[0.01; 0.11]	0.0%	0.3%
Alemayehu et al., 2020	6	383	0.02	[0.01; 0.03]	0.2%	0.3%
Lengongo et al., 2020	1	115	0.01	[0.00; 0.05]	0.1%	0.3%
Botero et al., 2021	1	192	0.01	[0.00; 0.03]	0.3%	0.3%
Betancourth et al., 2021	1	102	0.01	[0.00; 0.05]	0.1%	0.3%
Wolday et al., 2021	11	751	0.01	[0.01; 0.03]	0.4%	0.3%
Uzairue et al., 2021	4	170	0.02	[0.01; 0.06]	0.1%	0.3%
PrayGod et al., 2022	7	1149	0.01	[0.00; 0.01]	1.6%	0.3%
Common effect model		15077	0.01	[0.01; 0.01]	13.4%	--
Random effects model			0.04	[0.02; 0.07]	--	12.1%
Heterogeneity: $I^2 = 93\%$, $\tau^2 = 0.0082$, $p < 0.01$						
**Helminth_Family = Taeniidae**						
Abaver et al., 2011	1	85	0.01	[0.00; 0.06]	0.1%	0.3%
Abossie and Petros, 2015	2	97	0.02	[0.00; 0.07]	0.0%	0.3%
Adams et al., 2006	17	170	0.10	[0.06; 0.16]	0.0%	0.2%
Akinbo et al., 2011	4	463	0.01	[0.00; 0.02]	0.5%	0.3%
Akinbo et al., 2012	4	2000	0.00	[0.00; 0.01]	8.4%	0.3%
Assefa et al., 2009	3	214	0.01	[0.00; 0.04]	0.1%	0.3%
Assefa et al., 2013	4	272	0.01	[0.00; 0.04]	0.2%	0.3%
Cardoso et al., 2011	1	500	0.00	[0.00; 0.01]	2.1%	0.3%
Da Silva et al., 2005	1	100	0.01	[0.00; 0.05]	0.1%	0.3%
Fekadu et al., 2013	14	343	0.04	[0.02; 0.07]	0.1%	0.3%
Getaneh et al., 2018	3	220	0.01	[0.00; 0.04]	0.1%	0.3%
Gebretsadik et al., 2018	2	223	0.01	[0.00; 0.03]	0.2%	0.3%
Haileeyesus et al., 2013	10	378	0.03	[0.01; 0.05]	0.1%	0.3%
Kipyegen et al., 2012	8	285	0.03	[0.01; 0.05]	0.1%	0.3%
Missaye et al., 2013	4	272	0.01	[0.00; 0.04]	0.2%	0.3%
Modjarrad et al., 2005	1	297	0.00	[0.00; 0.02]	0.7%	0.3%
Nuhu et al., 2014	2	205	0.01	[0.00; 0.03]	0.2%	0.3%
Ojurongbe et al., 2011	1	96	0.01	[0.00; 0.06]	0.1%	0.3%
Pennap et al., 2015	25	200	0.12	[0.08; 0.18]	0.0%	0.2%
Pinlaor et al., 2005	1	78	0.01	[0.00; 0.07]	0.1%	0.3%
Raytekar et al., 2012	2	127	0.02	[0.00; 0.06]	0.1%	0.3%
Shimelis et al., 2016	8	491	0.02	[0.01; 0.03]	0.3%	0.3%
Tigist et al., 2015	4	209	0.02	[0.01; 0.05]	0.1%	0.3%
Kaneshiro et al., 2019	7	252	0.03	[0.01; 0.06]	0.1%	0.3%
Alemayehu et al., 2020	12	383	0.03	[0.02; 0.05]	0.1%	0.3%
Belay et al., 2020	2	112	0.02	[0.00; 0.06]	0.1%	0.3%
Bayleyegn et al., 2021	2	255	0.01	[0.00; 0.03]	0.3%	0.3%
Feleke et al., 2022	3	223	0.01	[0.00; 0.04]	0.1%	0.3%
Chandi et al., 2021	6	80	0.07	[0.03; 0.16]	0.0%	0.2%
Wolday et al., 2021	3	751	0.00	[0.00; 0.01]	1.6%	0.3%
Common effect model		9381	0.01	[0.00; 0.01]	16.0%	--
Random effects model			0.02	[0.01; 0.02]	--	7.7%
Heterogeneity: $I^2 = 75\%$, $\tau^2 = < 0.0001$, $p < 0.01$						
**Helminth_Family = Schistosomatidae**						
Abbas et al., 2009	55	99	0.56	[0.45; 0.66]	0.0%	0.2%
Abbas et al., 2009	74	119	0.62	[0.53; 0.71]	0.0%	0.2%
Allam et al., 2014	68	141	0.48	[0.40; 0.57]	0.0%	0.2%
Assefa et al., 2009	4	214	0.02	[0.01; 0.05]	0.1%	0.3%
Assefa et al., 2013	1	272	0.00	[0.00; 0.02]	0.6%	0.3%
Colombe et al., 2018	37	79	0.47	[0.36; 0.58]	0.0%	0.2%
Costiniuk et al., 2012	7	97	0.07	[0.03; 0.14]	0.0%	0.2%
Erikstrup et al., 2008	190	198	0.96	[0.92; 0.98]	0.0%	0.2%
Eshetu et al., 2017	2	223	0.01	[0.00; 0.03]	0.2%	0.3%
Haileeyesus et al., 2013	5	378	0.01	[0.00; 0.03]	0.2%	0.3%
Hochberg et al., 2011	18	128	0.14	[0.09; 0.21]	0.0%	0.2%
Idindili et al., 2011	49	421	0.12	[0.09; 0.15]	0.0%	0.3%
Kallestrup et al., 2005	185	407	0.45	[0.41; 0.50]	0.0%	0.2%
Kawai et al., 2009	14	971	0.01	[0.01; 0.02]	0.6%	0.3%
Lar et al., 2016	21	205	0.10	[0.06; 0.15]	0.0%	0.2%
Loffredo et al., 2015	16	31	0.52	[0.33; 0.70]	0.0%	0.1%
Marti et al., 2017	60	305	0.20	[0.15; 0.25]	0.0%	0.2%
Mazigo et al., 2016	28	50	0.56	[0.41; 0.70]	0.0%	0.2%
Missaye et al., 2013	1	272	0.00	[0.00; 0.02]	0.6%	0.3%
Modjarrad et al., 2005	6	297	0.02	[0.01; 0.04]	0.1%	0.3%
Modjarrad et al., 2005	5	111	0.05	[0.01; 0.10]	0.0%	0.2%
Mtapuri et al., 2014	1	29	0.03	[0.00; 0.18]	0.0%	0.2%
Mulu et al., 2013	10	220	0.05	[0.02; 0.08]	0.0%	0.3%
Ndhlovu et al., 2007	72	156	0.46	[0.38; 0.54]	0.0%	0.2%
Pavie et al., 2012	2	143	0.01	[0.00; 0.05]	0.1%	0.3%
Roka et al., 2012	24	260	0.09	[0.06; 0.13]	0.0%	0.3%
Roka et al., 2013	20	273	0.07	[0.05; 0.11]	0.0%	0.3%
Samie et al., 2014	11	151	0.07	[0.04; 0.13]	0.0%	0.2%
Shimelis et al., 2016	3	491	0.01	[0.00; 0.02]	0.7%	0.3%
Smith et al., 2008	21	122	0.17	[0.11; 0.25]	0.0%	0.2%
Tegegne et al., 2017	2	223	0.01	[0.00; 0.03]	0.2%	0.3%
Tigist et al., 2015	1	209	0.00	[0.00; 0.03]	0.4%	0.3%
Wanyiri et al., 2013	3	164	0.02	[0.00; 0.05]	0.1%	0.3%
Wumba et al., 2010	1	175	0.01	[0.00; 0.03]	0.3%	0.3%
Gebrecherkos et al., 2019	2	150	0.01	[0.00; 0.05]	0.1%	0.3%
Alemayehu et al., 2020	3	383	0.01	[0.00; 0.02]	0.4%	0.3%
McLellan et al., 2020	47	253	0.19	[0.14; 0.24]	0.0%	0.2%
Feleke et al., 2022	2	223	0.01	[0.00; 0.03]	0.2%	0.3%
Wolday et al., 2021	34	751	0.05	[0.03; 0.06]	0.1%	0.3%
PrayGod et al., 2022	88	1149	0.08	[0.06; 0.09]	0.1%	0.3%
Common effect model		10543	0.03	[0.02; 0.03]	5.5%	--
Random effects model			0.16	[0.09; 0.24]	--	9.7%
Heterogeneity: $I^2 = 99\%$, $\tau^2 = 0.0523$, $p = 0$						
**Helminth_Family = Hymenolepididae**						
Adamu and Petros, 2009	4	200	0.02	[0.01; 0.05]	0.1%	0.3%
Adeleke et al., 2015	3	231	0.01	[0.00; 0.04]	0.2%	0.3%
Agholi et al., 2013	1	356	0.00	[0.00; 0.02]	1.1%	0.3%
Ahmed and Abhay, 2015	1	142	0.01	[0.00; 0.04]	0.2%	0.3%
Assefa et al., 2009	4	214	0.02	[0.01; 0.05]	0.1%	0.3%
Assefa et al., 2013	2	272	0.01	[0.00; 0.03]	0.3%	0.3%
Da Silva et al., 2005	1	100	0.01	[0.00; 0.05]	0.1%	0.3%
Eshetu et al., 2017	2	223	0.01	[0.00; 0.03]	0.2%	0.3%
Getaneh et al., 2018	2	220	0.01	[0.00; 0.03]	0.2%	0.3%
Gebretsadik et al., 2018	1	223	0.00	[0.00; 0.02]	0.4%	0.3%
Haileeyesus et al., 2013	6	378	0.02	[0.01; 0.03]	0.2%	0.3%
Kulkarni et al., 2009	2	137	0.01	[0.00; 0.05]	0.1%	0.3%
Lar et al., 2016	9	205	0.04	[0.02; 0.08]	0.0%	0.3%
Meamar et al., 2007	1	781	0.00	[0.00; 0.01]	5.1%	0.3%
Missaye et al., 2013	2	272	0.01	[0.00; 0.03]	0.3%	0.3%
Modjarrad et al., 2005	2	297	0.01	[0.00; 0.02]	0.4%	0.3%
Modjarrad et al., 2005	1	111	0.01	[0.00; 0.05]	0.1%	0.3%
Nsagha et al., 2016	4	300	0.01	[0.00; 0.03]	0.2%	0.3%
Ramakrishnan et al., 2007	1	80	0.01	[0.00; 0.07]	0.0%	0.3%
Ramana et al., 2010	14	452	0.03	[0.02; 0.05]	0.1%	0.3%
Rodrigues et al., 2008	1	582	0.00	[0.00; 0.01]	2.9%	0.3%
Samie et al., 2014	4	151	0.03	[0.01; 0.07]	0.0%	0.3%
Shimelis et al., 2016	1	491	0.00	[0.00; 0.01]	2.0%	0.3%
Sonali et al., 2014	2	250	0.01	[0.00; 0.03]	0.3%	0.3%
Suryawanshi et al., 2012	2	100	0.02	[0.00; 0.07]	0.0%	0.3%
Tegegne et al., 2017	2	223	0.01	[0.00; 0.03]	0.2%	0.3%
Tiwari et al., 2013	7	745	0.01	[0.00; 0.02]	0.7%	0.3%
Wanyiri et al., 2013	2	164	0.01	[0.00; 0.04]	0.1%	0.3%
Zeynudin et al., 2013	6	91	0.07	[0.02; 0.14]	0.0%	0.2%
Alemayehu et al., 2020	6	383	0.02	[0.01; 0.03]	0.2%	0.3%
Chhangte et al., 2020	1	250	0.00	[0.00; 0.02]	0.5%	0.3%
Bayleyegn et al., 2021	8	255	0.03	[0.01; 0.06]	0.1%	0.3%
Feleke et al., 2022	3	223	0.01	[0.00; 0.04]	0.1%	0.3%
Wolday et al., 2021	113	751	0.15	[0.13; 0.18]	0.0%	0.3%
Uzairue et al., 2021	7	170	0.04	[0.02; 0.08]	0.0%	0.3%
Common effect model		10023	0.01	[0.00; 0.01]	16.7%	--
Random effects model			0.02	[0.01; 0.02]	--	9.0%
Heterogeneity: $I^2 = 83\%$, $\tau^2 = 0.0004$, $p < 0.01$						
**Helminth_Family = Diphyllobothriidae**						
Adeleke et al., 2015	16	231	0.07	[0.04; 0.11]	0.0%	0.3%
Samie et al., 2014	18	151	0.12	[0.07; 0.18]	0.0%	0.2%
Common effect model		382	0.08	[0.06; 0.11]	0.0%	--
Random effects model			0.09	[0.04; 0.14]	--	0.5%
Heterogeneity: $I^2 = 61\%$, $\tau^2 = 0.0008$, $p = 0.11$						
**Helminth_Family = Fasciolidae**						
Adeleke et al., 2015	5	231	0.02	[0.01; 0.05]	0.1%	0.3%
Common effect model		231	0.02	[0.00; 0.04]	0.1%	--
Random effects model			0.02	[0.00; 0.04]	--	0.3%
Heterogeneity: not applicable						
**Helminth_Family = Oxyuridae**						
Agholi et al., 2013	3	356	0.01	[0.00; 0.02]	0.4%	0.3%
Assefa et al., 2009	2	214	0.01	[0.00; 0.03]	0.2%	0.3%
Assefa et al., 2013	1	272	0.00	[0.00; 0.02]	0.6%	0.3%
Eshetu et al., 2017	3	223	0.01	[0.00; 0.04]	0.1%	0.3%
Gebretsadik et al., 2018	1	223	0.00	[0.00; 0.02]	0.4%	0.3%
Haileeyesus et al., 2013	7	378	0.02	[0.01; 0.04]	0.2%	0.3%
Kipyegen et al., 2012	3	285	0.01	[0.00; 0.03]	0.2%	0.3%
Missaye et al., 2013	1	272	0.00	[0.00; 0.02]	0.6%	0.3%
Oyedeji et al., 2015	1	52	0.02	[0.00; 0.10]	0.0%	0.2%
Samie et al., 2014	6	151	0.04	[0.01; 0.08]	0.0%	0.3%
Tegegne et al., 2017	3	223	0.01	[0.00; 0.04]	0.1%	0.3%
Wanyiri et al., 2013	1	164	0.01	[0.00; 0.03]	0.2%	0.3%
Wumba et al., 2010	1	175	0.01	[0.00; 0.03]	0.3%	0.3%
Alemayehu et al., 2020	5	383	0.01	[0.00; 0.03]	0.2%	0.3%
Belay et al., 2020	2	112	0.02	[0.00; 0.06]	0.1%	0.3%
Feleke et al., 2022	5	223	0.02	[0.01; 0.05]	0.1%	0.3%
Common effect model		3706	0.01	[0.01; 0.01]	3.8%	--
Random effects model			0.01	[0.01; 0.01]	--	4.1%
Heterogeneity: $I^2 = 0\%$, $\tau^2 = < 0.0001$, $p = 0.45$						
**Helminth_Family = Onchocercidae**						
Costiniuk et al., 2012	4	97	0.04	[0.01; 0.10]	0.0%	0.2%
Kroidl et al., 2016	69	187	0.37	[0.30; 0.44]	0.0%	0.2%
Nielsen et al., 2007	18	34	0.53	[0.35; 0.70]	0.0%	0.1%
Talaat et al., 2015	28	80	0.35	[0.25; 0.46]	0.0%	0.2%
Talaat et al., 2008	40	632	0.06	[0.05; 0.09]	0.1%	0.3%
Common effect model		1030	0.09	[0.07; 0.10]	0.1%	--
Random effects model			0.26	[0.08; 0.44]	--	1.1%
Heterogeneity: $I^2 = 97\%$, $\tau^2 = 0.0412$, $p < 0.01$						
**Helminth_Family = Opisthorchiidae**						
Guk et al., 2005	2	105	0.02	[0.00; 0.07]	0.0%	0.2%
Paboriboune et al., 2014	65	137	0.47	[0.39; 0.56]	0.0%	0.2%
Pinlaor et al., 2005	15	78	0.19	[0.11; 0.30]	0.0%	0.2%
Saksirisampant et al., 2009	2	90	0.02	[0.00; 0.08]	0.0%	0.3%
Tian et al., 2012	1	302	0.00	[0.00; 0.02]	0.8%	0.3%
Kaneshiro et al., 2019	35	252	0.14	[0.10; 0.19]	0.0%	0.2%
Sannella et al., 2019	6	155	0.04	[0.01; 0.08]	0.0%	0.3%
Common effect model		1119	0.01	[0.01; 0.02]	0.9%	--
Random effects model			0.12	[0.00; 0.25]	--	1.7%
Heterogeneity: $I^2 = 97\%$, $\tau^2 = 0.0264$, $p < 0.01$						
**Helminth_Family = Gymnophallidae**						
Guk et al., 2005	1	105	0.01	[0.00; 0.05]	0.1%	0.3%
Common effect model		105	0.01	[0.00; 0.03]	0.1%	--
Random effects model			0.01	[0.00; 0.03]	--	0.3%
Heterogeneity: not applicable						
**Helminth_Family = Dicrocoeliidae**						
Meamar et al., 2007	1	781	0.00	[0.00; 0.01]	5.1%	0.3%
Common effect model		781	0.00	[0.00; 0.00]	5.1%	--
Random effects model			0.00	[0.00; 0.00]	--	0.3%
Heterogeneity: not applicable						
**Helminth_Family = Rhabditidae**						
Meamar et al., 2007	1	781	0.00	[0.00; 0.01]	5.1%	0.3%
Common effect model		781	0.00	[0.00; 0.00]	5.1%	--
Random effects model			0.00	[0.00; 0.00]	--	0.3%
Heterogeneity: not applicable						
**Helminth_Family = Echinostomatidae**						
Pinlaor et al., 2005	4	78	0.05	[0.01; 0.13]	0.0%	0.2%
Common effect model		78	0.05	[0.00; 0.10]	0.0%	--
Random effects model			0.05	[0.00; 0.10]	--	0.2%
Heterogeneity: not applicable						
**Common effect model**		**111742**	**0.01**	**[0.01; 0.01]**	**100.0%**	**--**
**Random effects model**			**0.06**	**[0.05; 0.07]**	**--**	**100.0%**
Heterogeneity: $I^2 = 96\%$, $\tau^2 = 0.0097$, $p = 0$						

0 0.2 0.4 0.6 0.8

Test for subgroup differences (fixed effect): $\chi^2_{15} = 905.60$, df = 15 ($p < 0.01$)

Test for subgroup differences (random effects): $\chi^2_{15} = 270.34$, df = 15 ($p < 0.01$)

Figure S1B: Forest plots of the prevalence rates of protozoan co-infections in virus-infected people at family level by meta-analysis using common effect and random-effects models

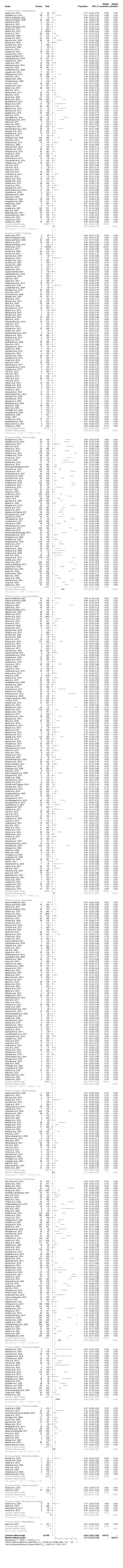

Figure S2A: Forest plots of the prevalence rates of helminth co-infections in virus-infected people at genus level by meta-analysis using common effect and random-effects models

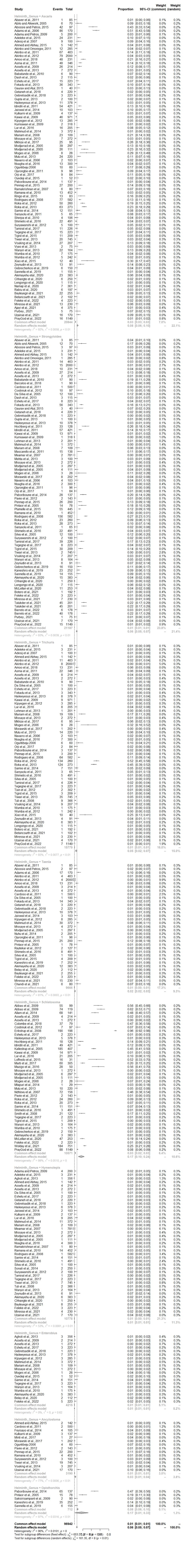

Figure S2B: Forest plots of the prevalence rates of protozoan co-infections in virus-infected people at genus level by meta-analysis using common effect and random-effects models

Study	Events	Total	Proportion	95%-CI	Weight (common)	Weight (random)
**Protozoa_Genus = Plasmodium**						
Kinimi et al., 2018	8	97	0.08	[0.04; 0.16]	0.0%	0.1%
Arundhati and Niharika, 2016	16	101	0.16	[0.09; 0.24]	0.0%	0.1%
Assir et al., 2014	22	85	0.26	[0.17; 0.37]	0.0%	0.1%
Epelboin et al., 2012	104	208	0.50	[0.43; 0.57]	0.0%	0.1%
Kolawole et al., 2017	5	81	0.06	[0.02; 0.14]	0.0%	0.1%
Magalhaes et al., 2014	44	628	0.07	[0.05; 0.09]	0.1%	0.1%
Rao et al., 2016	22	745	0.03	[0.02; 0.04]	0.3%	0.1%
Dabo et al., 2015	9	13	0.69	[0.39; 0.91]	0.0%	0.1%
Omalu et al., 2012	27	30	0.90	[0.73; 0.98]	0.0%	0.1%
Scotto and Fazio, 2018	15	41	0.37	[0.22; 0.53]	0.0%	0.1%
Anabire et al., 2019	36	191	0.19	[0.14; 0.25]	0.0%	0.1%
Adu-Gyasi et al., 2013	15	933	0.02	[0.01; 0.03]	0.6%	0.1%
Akinbo et al., 2016	32	411	0.08	[0.05; 0.11]	0.1%	0.1%
Akinbo et al., 2012	87	2000	0.04	[0.03; 0.05]	0.5%	0.1%
Ako-Nai et al., 2013	34	44	0.77	[0.62; 0.89]	0.0%	0.1%
Amuta et al., 2012	343	1080	0.32	[0.29; 0.35]	0.1%	0.1%
Bate et al., 2016	58	234	0.25	[0.19; 0.31]	0.0%	0.1%
Bharti et al., 2012	41	460	0.09	[0.06; 0.12]	0.1%	0.1%
Chalwe et al., 2009	42	55	0.76	[0.63; 0.87]	0.0%	0.1%
Chukwuanukwu et al., 2016	110	239	0.46	[0.40; 0.53]	0.0%	0.1%
Cohen et al., 2005	18	110	0.16	[0.10; 0.25]	0.0%	0.1%
Desta et al., 2005	8	49	0.16	[0.07; 0.30]	0.0%	0.1%
Franke et al., 2010	199	1066	0.19	[0.16; 0.21]	0.1%	0.1%
Frosch et al., 2017	11	138	0.08	[0.04; 0.14]	0.0%	0.1%
Gallagher et al., 2005	34	83	0.41	[0.30; 0.52]	0.0%	0.1%
Goselle et al., 2007	64	200	0.32	[0.26; 0.39]	0.0%	0.1%
Hasang et al., 2014	100	216	0.46	[0.40; 0.53]	0.0%	0.1%
Idindili et al., 2011	105	421	0.25	[0.21; 0.29]	0.0%	0.1%
Igbeneghu et al., 2012	18	38	0.47	[0.31; 0.64]	0.0%	0.1%
Iroezindu et al., 2012	24	97	0.25	[0.17; 0.35]	0.0%	0.1%
Iroezindu et al., 2012	21	63	0.33	[0.22; 0.46]	0.0%	0.1%
Izuka et al., 2017	70	102	0.69	[0.59; 0.77]	0.0%	0.1%
Johnbull et al., 2014	149	301	0.50	[0.44; 0.55]	0.0%	0.1%
Kapito et al., 2011	113	1142	0.10	[0.08; 0.12]	0.1%	0.1%
Kawai et al., 2009	177	971	0.18	[0.16; 0.21]	0.1%	0.1%
Kfutwah et al., 2009	14	80	0.17	[0.10; 0.28]	0.0%	0.1%
Kiying et al., 2010	120	160	0.75	[0.68; 0.81]	0.0%	0.1%
Manyanga et al., 2014	19	420	0.05	[0.03; 0.07]	0.1%	0.1%
Marete et al., 2014	201	245	0.82	[0.77; 0.87]	0.0%	0.1%
Mohapatra et al., 2017	23	333	0.07	[0.04; 0.10]	0.1%	0.1%
Morona et al., 2018	56	250	0.22	[0.17; 0.28]	0.0%	0.1%
Njunda et al., 2016	26	355	0.07	[0.05; 0.11]	0.1%	0.1%
Nkhoma et al., 2012	70	185	0.38	[0.31; 0.45]	0.0%	0.1%
Okogun et al., 2010	5	16	0.31	[0.11; 0.59]	0.0%	0.1%
Okonkwo et al., 2018	61	179	0.34	[0.27; 0.42]	0.0%	0.1%
Olaniran et al., 2012	83	93	0.89	[0.81; 0.95]	0.0%	0.1%
Omoti et al., 2013	254	342	0.74	[0.69; 0.79]	0.0%	0.1%
Onyenekwe et al., 2007	28	149	0.19	[0.13; 0.26]	0.0%	0.1%
Osuji et al., 2012	74	134	0.55	[0.46; 0.64]	0.0%	0.1%
Quigley et al., 2005	154	222	0.69	[0.63; 0.75]	0.0%	0.1%
Sanyaolu et al., 2013	31	65	0.48	[0.35; 0.60]	0.0%	0.1%
Saracino et al., 2012	29	220	0.13	[0.09; 0.18]	0.0%	0.1%
Tagoe and Boachie, 2012	34	220	0.15	[0.11; 0.21]	0.0%	0.1%
Tay et al., 2015	47	400	0.12	[0.09; 0.15]	0.0%	0.1%
Tegenaw et al., 2013	2	23	0.09	[0.01; 0.28]	0.0%	0.1%
Uneke et al., 2005	103	490	0.21	[0.17; 0.25]	0.0%	0.1%
Wumba et al., 2015	19	25	0.76	[0.55; 0.91]	0.0%	0.1%
Zheng et al., 2017	14	101	0.14	[0.08; 0.22]	0.0%	0.1%
Chukwuocha et al., 2019	398	398	1.00	[0.99; 1.00]	3.6%	0.1%
Amodu et al., 2020	100	140	0.71	[0.63; 0.79]	0.0%	0.1%
Anyanwu et al., 2020	142	420	0.34	[0.29; 0.39]	0.0%	0.1%
Jegede et al., 2020	386	1521	0.25	[0.23; 0.28]	0.1%	0.1%
Rabiu et al., 2020	49	197	0.25	[0.19; 0.32]	0.0%	0.1%
Sandie et al., 2021	31	190	0.16	[0.11; 0.22]	0.0%	0.1%
Stiffler et al., 2021	44	129	0.34	[0.26; 0.43]	0.0%	0.1%
Alonso et al., 2021	26	144	0.18	[0.12; 0.25]	0.0%	0.1%
Jemikalajah et al., 2021	228	300	0.76	[0.71; 0.81]	0.0%	0.1%
Thompson et al., 2012	149	2408	0.06	[0.05; 0.07]	0.5%	0.1%
Onosakponome et al., 2020	300	300	1.00	[0.99; 1.00]	2.0%	0.1%
Common effect model		23527	0.66	[0.66; 0.67]	9.0%	--
Random effects model			0.35	[0.28; 0.41]	--	9.5%
Heterogeneity: $I^2 = 100\%$, $\tau^2 = 0.0737$, $p = 0$						
**Protozoa_Genus = Toxoplasma**						
Simpore et al., 2006	12	33	0.36	[0.20; 0.55]	0.0%	0.1%
El Nahas et al., 2014	105	120	0.88	[0.80; 0.93]	0.0%	0.1%
Simpore et al., 2006	4	18	0.22	[0.06; 0.48]	0.0%	0.1%
Abdollahi et al., 2013	65	100	0.65	[0.55; 0.74]	0.0%	0.1%
Addebbous et al., 2012	59	95	0.62	[0.52; 0.72]	0.0%	0.1%
Aghaee et al., 2017	11	49	0.22	[0.12; 0.37]	0.0%	0.1%
Alavi et al., 2013	31	42	0.74	[0.58; 0.86]	0.0%	0.1%
Amuta et al., 2012	39	360	0.11	[0.08; 0.15]	0.0%	0.1%
Angal et al., 2016	84	133	0.63	[0.54; 0.71]	0.0%	0.1%
Anteneh et al., 2014	178	190	0.94	[0.89; 0.97]	0.0%	0.1%
Arefkhah et al., 2018	42	251	0.17	[0.12; 0.22]	0.0%	0.1%
Assob et al., 2011	93	133	0.70	[0.61; 0.78]	0.0%	0.1%
Ayi et al., 2016	72	125	0.58	[0.48; 0.66]	0.0%	0.1%
Bamba et al., 2014	11	16	0.69	[0.41; 0.89]	0.0%	0.1%
Beena et al., 2015	141	661	0.21	[0.18; 0.25]	0.0%	0.1%
Bessong and Mathomu 2010	29	160	0.18	[0.12; 0.25]	0.0%	0.1%
Chemoh et al., 2015	109	300	0.36	[0.31; 0.42]	0.0%	0.1%
Daryani et al., 2011	54	62	0.87	[0.76; 0.94]	0.0%	0.1%
Davarpanah et al., 2007	38	208	0.18	[0.13; 0.24]	0.0%	0.1%
Dechassa et al., 2016	109	135	0.81	[0.73; 0.87]	0.0%	0.1%
Domingos et al., 2013	92	200	0.46	[0.39; 0.53]	0.0%	0.1%
Ebrahim et al., 2018	51	246	0.21	[0.16; 0.26]	0.0%	0.1%
Fisseha et al., 2013	90	103	0.87	[0.79; 0.93]	0.0%	0.1%
Ganiem et al., 2013	21	64	0.33	[0.22; 0.46]	0.0%	0.1%
Hari et al., 2007	25	307	0.08	[0.05; 0.12]	0.0%	0.1%
Hung et al., 2005	56	550	0.10	[0.08; 0.13]	0.1%	0.1%
Kodym et al., 2015	502	1235	0.41	[0.38; 0.43]	0.1%	0.1%
Lago et al., 2009	121	168	0.72	[0.65; 0.79]	0.0%	0.1%
Li et al., 2010	58	183	0.32	[0.25; 0.39]	0.0%	0.1%
Machala et al., 2009	268	626	0.43	[0.39; 0.47]	0.0%	0.1%
Malla et al., 2005	14	100	0.14	[0.08; 0.22]	0.0%	0.1%
Mengistu et al., 2014	38	43	0.88	[0.75; 0.96]	0.0%	0.1%
Mohraz et al., 2011	100	201	0.50	[0.43; 0.57]	0.0%	0.1%
Nazari et al., 2018	167	385	0.43	[0.38; 0.48]	0.0%	0.1%
Ngobeni and Samie, 2017	38	100	0.38	[0.28; 0.48]	0.0%	0.1%
Nissapatorn et al., 2007	329	693	0.47	[0.44; 0.51]	0.0%	0.1%
Ogoina et al., 2013	43	111	0.39	[0.30; 0.48]	0.0%	0.1%
Oshinaike et al., 2010	71	83	0.86	[0.76; 0.92]	0.0%	0.1%
Osunkalu et al., 2011	206	380	0.54	[0.49; 0.59]	0.0%	0.1%
Ouermi et al., 2009	44	138	0.32	[0.24; 0.40]	0.0%	0.1%
Pappoe et al., 2017	293	394	0.74	[0.70; 0.79]	0.0%	0.1%
Rahimi et al., 2015	79	82	0.96	[0.90; 0.99]	0.0%	0.1%
Reema and Dharmendra, 2017	52	100	0.52	[0.42; 0.62]	0.0%	0.1%
Rezanezhad et al., 2017	19	90	0.21	[0.13; 0.31]	0.0%	0.1%
Rostami et al., 2014	19	94	0.20	[0.13; 0.30]	0.0%	0.1%
Shafieenia et al., 2018	142	379	0.37	[0.33; 0.43]	0.0%	0.1%
Shafiei et al., 2011	49	121	0.40	[0.32; 0.50]	0.0%	0.1%
Shen et al., 2016	25	259	0.10	[0.06; 0.14]	0.0%	0.1%
Simpore et al., 2006	59	207	0.29	[0.22; 0.35]	0.0%	0.1%
Sitoe et al., 2010	18	58	0.31	[0.20; 0.45]	0.0%	0.1%
Tsegaye et al., 2014	150	170	0.88	[0.82; 0.93]	0.0%	0.1%
Xavier et al., 2013	200	250	0.80	[0.74; 0.85]	0.0%	0.1%
Zeleke and Melsew, 2017	255	270	0.94	[0.91; 0.97]	0.1%	0.1%
Bavand et al., 2019	66	149	0.44	[0.36; 0.53]	0.0%	0.1%
Bokharaei et al., 2020	95	108	0.88	[0.80; 0.93]	0.0%	0.1%
Dutta et al., 2020	292	400	0.73	[0.68; 0.77]	0.0%	0.1%
Hoshina et al., 2020	33	399	0.08	[0.06; 0.11]	0.1%	0.1%
Moro et al., 2020	81	332	0.24	[0.20; 0.29]	0.0%	0.1%
Zakari et al., 2020	54	160	0.34	[0.26; 0.42]	0.0%	0.1%
Steinberg et al., 2021	173	215	0.80	[0.75; 0.86]	0.0%	0.1%
Fang et al., 2021	164	325	0.50	[0.45; 0.56]	0.0%	0.1%
Sajedi et al., 2021	73	121	0.60	[0.51; 0.69]	0.0%	0.1%
Common effect model		13790	0.44	[0.44; 0.45]	1.0%	--
Random effects model			0.49	[0.42; 0.56]	--	8.6%
Heterogeneity: $I^2 = 99\%$, $\tau^2 = 0.0708$, $p = 0$						
**Protozoa_Genus = Cryptosporidium**						
Yu et al. 2011	14	340	0.04	[0.02; 0.07]	0.1%	0.1%
Abilo and Afework, 2005	20	70	0.29	[0.18; 0.41]	0.0%	0.1%
Adamu and Petros, 2009	16	200	0.08	[0.05; 0.13]	0.0%	0.1%
Adamu et al., 2014	139	520	0.27	[0.23; 0.31]	0.0%	0.1%
Adesij et al. 2007	79	100	0.79	[0.70; 0.87]	0.0%	0.1%
Agholi et al., 2013	34	356	0.10	[0.07; 0.13]	0.0%	0.1%
Ahmed and Abhay, 2015	6	142	0.04	[0.02; 0.09]	0.0%	0.1%
Ajjampur et al., 2007	48	534	0.09	[0.07; 0.12]	0.1%	0.1%
Akinbo et al., 2010	36	400	0.09	[0.06; 0.12]	0.1%	0.1%
Akinbo et al., 2012	74	2000	0.04	[0.03; 0.05]	0.6%	0.1%
Alberto et al., 2011	38	131	0.29	[0.21; 0.38]	0.0%	0.1%
Amoo et al., 2018	52	231	0.23	[0.17; 0.28]	0.0%	0.1%
Asma et al., 2011	43	346	0.12	[0.09; 0.16]	0.0%	0.1%
Assefa et al., 2009	43	214	0.20	[0.15; 0.26]	0.0%	0.1%
Assefa et al., 2013	2	272	0.01	[0.00; 0.03]	0.3%	0.1%
Babatunde et al., 2010	29	90	0.32	[0.23; 0.43]	0.0%	0.1%
Barcelos et al., 2018	1	90	0.01	[0.00; 0.06]	0.1%	0.1%
Cardoso et al., 2011	1	500	0.00	[0.00; 0.01]	1.9%	0.1%
da Silva et al., 2005	4	100	0.04	[0.01; 0.10]	0.0%	0.1%
Daryani et al., 2009	6	64	0.09	[0.04; 0.19]	0.0%	0.1%
Dash et al., 2013	14	115	0.12	[0.07; 0.20]	0.0%	0.1%
Djieyep et al., 2014	127	305	0.42	[0.36; 0.47]	0.0%	0.1%
Echoru et al., 2015	4	111	0.04	[0.01; 0.09]	0.0%	0.1%
Erhabor et al., 2011	3	105	0.03	[0.01; 0.08]	0.0%	0.1%
Eshetu et al., 2017	7	223	0.03	[0.01; 0.06]	0.1%	0.1%
Gabriela et al., 2005	59	397	0.15	[0.12; 0.19]	0.0%	0.1%
Gaurav and Atul, 2015	6	40	0.15	[0.06; 0.30]	0.0%	0.1%
Gebretsadik et al., 2018	3	223	0.01	[0.00; 0.04]	0.2%	0.1%
Getaneh et al., 2018	19	220	0.09	[0.05; 0.13]	0.0%	0.1%
Ghafari et al., 2018	27	250	0.11	[0.07; 0.15]	0.0%	0.1%
Gholami et al., 2016	4	53	0.08	[0.02; 0.18]	0.0%	0.1%
Girma et al., 2014	92	268	0.34	[0.29; 0.40]	0.0%	0.1%
Guk et al., 2005	7	105	0.07	[0.03; 0.13]	0.0%	0.1%
Gupta et al., 2013	4	100	0.04	[0.01; 0.10]	0.0%	0.1%
Haileeyesus et al., 2013	32	378	0.08	[0.06; 0.12]	0.1%	0.1%
Huibers et al., 2018	4	35	0.11	[0.03; 0.27]	0.0%	0.1%
Hung et al., 2007	5	1376	0.00	[0.00; 0.01]	3.9%	0.1%
Hussain et al., 2016	25	127	0.20	[0.13; 0.28]	0.0%	0.1%
Iqbal et al., 2015	43	346	0.12	[0.09; 0.16]	0.0%	0.1%
Jaiswal et al., 2014	3	103	0.03	[0.01; 0.08]	0.0%	0.1%
Jayalakshmi et al., 2008	12	89	0.13	[0.07; 0.22]	0.0%	0.1%
Javid et al., 2005	84	200	0.42	[0.35; 0.49]	0.0%	0.1%
Kammili et al., 2008	12	175	0.07	[0.04; 0.12]	0.0%	0.1%
Kiros et al., 2015	23	399	0.06	[0.04; 0.09]	0.1%	0.1%
Kucerova et al., 2011	19	46	0.41	[0.27; 0.57]	0.0%	0.1%
Kulkarni et al., 2009	16	137	0.12	[0.07; 0.18]	0.0%	0.1%
Kurniawan et al., 2013	30	36	0.83	[0.67; 0.94]	0.0%	0.1%
Kurniawan et al., 2009	32	318	0.10	[0.07; 0.14]	0.0%	0.1%
Langoni and Souza, 2007	11	105	0.10	[0.05; 0.18]	0.0%	0.1%
Lar et al., 2016	17	205	0.08	[0.05; 0.13]	0.0%	0.1%
Lehman et al., 2013	13	201	0.06	[0.03; 0.11]	0.0%	0.1%
Maikai et al., 2012	1	61	0.02	[0.00; 0.09]	0.0%	0.1%
Mariam et al., 2008	8	109	0.07	[0.03; 0.14]	0.0%	0.1%
Masarat et al., 2012	45	45	1.00	[0.92; 1.00]	0.0%	0.1%
Mathur et al., 2013	135	544	0.25	[0.21; 0.29]	0.0%	0.1%
Meamar et al., 2007	7	781	0.01	[0.00; 0.02]	0.9%	0.1%
Mehta et al., 2013	2	100	0.02	[0.00; 0.07]	0.0%	0.1%
Mekonnen et al., 2014	92	268	0.34	[0.29; 0.40]	0.0%	0.1%
Missaye et al., 2013	2	272	0.01	[0.00; 0.03]	0.3%	0.1%
Mitra et al., 2016	57	194	0.29	[0.23; 0.36]	0.0%	0.1%
Mohan et al., 2014	2	150	0.01	[0.00; 0.05]	0.1%	0.1%
Morawski et al., 2017	1	202	0.00	[0.00; 0.03]	0.3%	0.1%
Mtapuri et al., 2014	5	29	0.17	[0.06; 0.36]	0.0%	0.1%
Ngum et al., 2015	46	320	0.14	[0.11; 0.19]	0.0%	0.1%
Nkenfou et al., 2013	8	42	0.19	[0.09; 0.34]	0.0%	0.1%
Nkenfou et al., 2017	5	68	0.07	[0.02; 0.16]	0.0%	0.1%
Nsagha et al., 2016	132	300	0.44	[0.38; 0.50]	0.0%	0.1%
Nuhu et al., 2014	19	205	0.09	[0.06; 0.14]	0.0%	0.1%
Oguntibeju 2006	6	60	0.10	[0.04; 0.21]	0.0%	0.1%
Ojuromi et al., 2016	4	90	0.04	[0.01; 0.11]	0.0%	0.1%
Olopade et al., 2017	10	226	0.04	[0.02; 0.08]	0.1%	0.1%
Oyedeji et al., 2015	10	52	0.19	[0.10; 0.33]	0.0%	0.1%
Paboriboune et al., 2014	9	137	0.07	[0.03; 0.12]	0.0%	0.1%
Patel et al., 2011	20	100	0.20	[0.13; 0.29]	0.0%	0.1%
Pavie et al., 2012	9	143	0.06	[0.03; 0.12]	0.0%	0.1%
Pennap et al., 2015	9	200	0.04	[0.02; 0.08]	0.1%	0.1%
Pinlaor et al., 2005	9	78	0.12	[0.05; 0.21]	0.0%	0.1%
Ramakrishnan et al., 2007	23	80	0.29	[0.19; 0.40]	0.0%	0.1%
Ramana et al., 2010	48	452	0.11	[0.08; 0.14]	0.1%	0.1%
Rashmi and Ravi, 2013	15	90	0.17	[0.10; 0.26]	0.0%	0.1%
Raytekar et al., 2012	9	127	0.07	[0.03; 0.13]	0.0%	0.1%
Rodrigues et al., 2008	39	582	0.07	[0.05; 0.09]	0.1%	0.1%
Roka et al., 2012	24	260	0.09	[0.06; 0.13]	0.0%	0.1%
Roka et al., 2013	8	273	0.03	[0.01; 0.06]	0.1%	0.1%
Saksirisampant et al., 2009	31	90	0.34	[0.25; 0.45]	0.0%	0.1%
Samie et al., 2014	40	151	0.26	[0.20; 0.34]	0.0%	0.1%
Sanyaolu et al., 2011	5	65	0.08	[0.03; 0.17]	0.0%	0.1%
Sarfati et al., 2006	6	154	0.04	[0.01; 0.08]	0.0%	0.1%
Sherchan et al., 2012	4	146	0.03	[0.01; 0.07]	0.1%	0.1%
Sherpa et al., 2010	8	106	0.08	[0.03; 0.14]	0.0%	0.1%
Shimelis and Tadesse, 2014	32	250	0.13	[0.09; 0.18]	0.0%	0.1%
Shimelis et al., 2016	65	491	0.13	[0.10; 0.17]	0.0%	0.1%
Silva et al., 2005	4	100	0.04	[0.01; 0.10]	0.0%	0.1%
Sintayehu et al., 2013	10	343	0.03	[0.01; 0.05]	0.1%	0.1%
Sonali et al., 2014	52	250	0.21	[0.16; 0.26]	0.0%	0.1%
Srisuphanunt et al., 2008	23	143	0.16	[0.10; 0.23]	0.0%	0.1%
Stark et al., 2007	14	628	0.02	[0.01; 0.04]	0.3%	0.1%
Stensvold et al., 2011	1	96	0.01	[0.00; 0.06]	0.1%	0.1%
Sucilathangam et al., 2012	48	103	0.47	[0.37; 0.57]	0.0%	0.1%
Suryawanshi et al., 2012	15	100	0.15	[0.09; 0.24]	0.0%	0.1%
Taherkhani et al., 2007	20	75	0.27	[0.17; 0.38]	0.0%	0.1%
Tegegne et al., 2017	7	223	0.03	[0.01; 0.06]	0.1%	0.1%
Tellevik et al., 2015	8	33	0.24	[0.11; 0.42]	0.0%	0.1%
Tian et al., 2012	25	302	0.08	[0.05; 0.12]	0.0%	0.1%
Tian et al., 2013	8	79	0.10	[0.04; 0.19]	0.0%	0.1%
Tiwari et al. 2013	23	745	0.03	[0.02; 0.05]	0.3%	0.1%
Tuli et al., 2008	146	366	0.40	[0.35; 0.45]	0.0%	0.1%
Tumwine et al., 2005	67	91	0.74	[0.63; 0.82]	0.0%	0.1%
Uysal et al., 2017	3	115	0.03	[0.01; 0.07]	0.0%	0.1%
Vouking et al., 2014	15	207	0.07	[0.04; 0.12]	0.0%	0.1%
Vyas et al., 2013	11	75	0.15	[0.08; 0.25]	0.0%	0.1%
Wang et al., 2013	11	683	0.02	[0.01; 0.03]	0.5%	0.1%
Wanyiri et al., 2013	17	164	0.10	[0.06; 0.16]	0.0%	0.1%
Wanyiri et al., 2014	56	164	0.34	[0.27; 0.42]	0.0%	0.1%
Wumba et al., 2010	17	175	0.10	[0.06; 0.15]	0.0%	0.1%
Yosefi et al., 2012	8	60	0.13	[0.06; 0.25]	0.0%	0.1%
Zeynudin et al., 2013	8	91	0.09	[0.04; 0.17]	0.0%	0.1%
Gebrecherkos et al., 2019	10	150	0.07	[0.03; 0.12]	0.0%	0.1%
Sannella et al., 2019	104	155	0.67	[0.59; 0.74]	0.0%	0.1%
Abange et al., 2020	7	119	0.06	[0.02; 0.12]	0.0%	0.1%
Ayele et al., 2020	15	180	0.08	[0.05; 0.13]	0.0%	0.1%
Belay et al., 2020	9	112	0.08	[0.04; 0.15]	0.0%	0.1%
Chhangte et al., 2020	1	250	0.00	[0.00; 0.02]	0.5%	0.1%
Lengongo et al., 2020	1	115	0.01	[0.00; 0.05]	0.1%	0.1%
Namaji et al., 2020	89	361	0.25	[0.20; 0.29]	0.0%	0.1%
Purbey., 2021	2	75	0.03	[0.00; 0.09]	0.0%	0.1%
Sepahvand et al., 2022	7	26	0.27	[0.12; 0.48]	0.0%	0.1%
Uzairue et al., 2021	25	170	0.15	[0.10; 0.21]	0.0%	0.1%
Yulfi et al., 2021	13	54	0.24	[0.13; 0.38]	0.0%	0.1%
Weinreich et al., 2021	86	967	0.09	[0.07; 0.11]	0.1%	0.1%
Ajayi et al., 2021	19	375	0.05	[0.03; 0.08]	0.1%	0.1%
Bayleyegn et al., 2021	2	255	0.01	[0.00; 0.03]	0.3%	0.1%
Betancourth et al., 2021	4	102	0.04	[0.01; 0.10]	0.0%	0.1%
Botero et al., 2021	1	192	0.01	[0.00; 0.03]	0.3%	0.1%
Chandi et al., 2021	5	80	0.06	[0.02; 0.14]	0.0%	0.1%
Dankwa et al., 2021	26	418	0.06	[0.04; 0.09]	0.1%	0.1%
Feleke et al., 2022	4	223	0.02	[0.00; 0.05]	0.1%	0.1%
Wumba et al., 2012	13	121	0.11	[0.06; 0.18]	0.0%	0.1%
Common effect model		31495	0.03	[0.03; 0.03]	14.4%	--
Random effects model			0.14	[0.11; 0.17]	--	19.4%
Heterogeneity: $I^2 = 98\%$, $\tau^2 = 0.0267$, $p = 0$						
**Protozoa_Genus = Entamoeba**						
Abaver et al., 2011	9	85	0.11	[0.05; 0.19]	0.0%	0.1%
Abdollahi et al., 2015	28	91	0.31	[0.22; 0.41]	0.0%	0.1%
Abilo and Afework, 2005	3	70	0.04	[0.01; 0.12]	0.0%	0.1%
Adamu and Petros, 2009	26	200	0.13	[0.09; 0.18]	0.0%	0.1%
Adesij et al. 2007	6	100	0.06	[0.02; 0.13]	0.0%	0.1%
Agholi et al., 2013	7	356	0.02	[0.01; 0.04]	0.2%	0.1%
Ahmed and Abhay, 2015	7	142	0.05	[0.02; 0.10]	0.0%	0.1%
Akinbo et al., 2011	5	463	0.01	[0.00; 0.03]	0.4%	0.1%
Akinbo et al., 2012	6	2000	0.00	[0.00; 0.01]	7.0%	0.1%
Asma et al., 2011	58	346	0.17	[0.13; 0.21]	0.0%	0.1%
Assefa et al., 2009	57	214	0.27	[0.21; 0.33]	0.0%	0.1%
Assefa et al., 2013	43	272	0.16	[0.12; 0.21]	0.0%	0.1%
Babatunde et al., 2010	31	90	0.34	[0.25; 0.45]	0.0%	0.1%
Barcelos et al., 2018	14	90	0.16	[0.09; 0.25]	0.0%	0.1%
Berenji et al., 2010	3	31	0.10	[0.02; 0.26]	0.0%	0.1%
Cardoso et al., 2011	4	500	0.01	[0.00; 0.02]	0.6%	0.1%
Daryani et al., 2009	2	64	0.03	[0.00; 0.11]	0.0%	0.1%
Dash et al., 2013	9	115	0.08	[0.04; 0.14]	0.0%	0.1%
Eshetu et al., 2017	19	223	0.09	[0.05; 0.13]	0.0%	0.1%
Feyisayo et al., 2014	7	105	0.07	[0.03; 0.13]	0.0%	0.1%
Gaurav and Atul, 2015	2	40	0.05	[0.01; 0.17]	0.0%	0.1%
Gebretsadik et al., 2018	16	223	0.07	[0.04; 0.11]	0.0%	0.1%
Getaneh et al., 2018	8	220	0.04	[0.02; 0.07]	0.1%	0.1%
Gupta et al., 2013	11	100	0.11	[0.06; 0.19]	0.0%	0.1%
Haileeyesus et al., 2013	16	378	0.04	[0.02; 0.07]	0.1%	0.1%
Hung et al., 2008	66	991	0.07	[0.05; 0.08]	0.2%	0.1%
Jaiswal et al., 2014	11	103	0.11	[0.05; 0.18]	0.0%	0.1%
Jayalakshmi et al., 2008	3	89	0.03	[0.01; 0.10]	0.0%	0.1%
Javid et al., 2005	35	200	0.17	[0.13; 0.23]	0.0%	0.1%
Jegede et al., 2014	7	105	0.07	[0.03; 0.13]	0.0%	0.1%
Kipyegen et al., 2012	97	285	0.34	[0.29; 0.40]	0.0%	0.1%
Kiros et al., 2015	77	399	0.19	[0.16; 0.24]	0.0%	0.1%
Kulkarni et al., 2009	9	137	0.07	[0.03; 0.12]	0.0%	0.1%
Kurniawan et al., 2009	3	318	0.01	[0.00; 0.03]	0.3%	0.1%
Lar et al., 2016	33	205	0.16	[0.11; 0.22]	0.0%	0.1%
Lehman et al., 2013	6	201	0.03	[0.01; 0.06]	0.1%	0.1%
Mahmud et al., 2014	147	372	0.40	[0.35; 0.45]	0.0%	0.1%
Mariam et al., 2008	21	109	0.19	[0.12; 0.28]	0.0%	0.1%
Mathur et al., 2013	49	544	0.09	[0.07; 0.12]	0.1%	0.1%
Minti et al., 2017	1	27	0.04	[0.00; 0.19]	0.0%	0.1%
Missaye et al., 2013	43	272	0.16	[0.12; 0.21]	0.0%	0.1%
Mitra et al., 2016	38	194	0.20	[0.14; 0.26]	0.0%	0.1%
Moges et al., 2006	1	28	0.04	[0.00; 0.18]	0.0%	0.1%
Moran et al., 2009	31	100	0.31	[0.22; 0.41]	0.0%	0.1%
Moran et al., 2005	90	203	0.44	[0.37; 0.51]	0.0%	0.1%
Morawski et al., 2017	3	202	0.01	[0.00; 0.04]	0.1%	0.1%
Mtapuri et al., 2014	9	29	0.31	[0.15; 0.51]	0.0%	0.1%
Navarro et al., 2006	10	103	0.10	[0.05; 0.17]	0.0%	0.1%
Ngum et al., 2015	79	320	0.25	[0.20; 0.30]	0.0%	0.1%
Nkenfou et al., 2013	17	42	0.40	[0.26; 0.57]	0.0%	0.1%
Nkenfou et al., 2017	20	68	0.29	[0.19; 0.42]	0.0%	0.1%
Nsagha et al., 2016	22	300	0.07	[0.05; 0.11]	0.0%	0.1%
Nuhu et al., 2014	2	205	0.01	[0.00; 0.03]	0.2%	0.1%
Oguntibeju 2006	10	60	0.17	[0.08; 0.29]	0.0%	0.1%
Ojurongbe et al., 2011	56	96	0.58	[0.48; 0.68]	0.0%	0.1%
Paboriboune et al., 2014	23	137	0.17	[0.11; 0.24]	0.0%	0.1%
Patel et al., 2011	2	100	0.02	[0.00; 0.07]	0.0%	0.1%
Pavie et al., 2012	4	143	0.03	[0.01; 0.07]	0.1%	0.1%
Pennap et al., 2015	20	200	0.10	[0.06; 0.15]	0.0%	0.1%
Ramakrishnan et al., 2007	30	80	0.38	[0.27; 0.49]	0.0%	0.1%
Ramana et al., 2010	70	452	0.15	[0.12; 0.19]	0.0%	0.1%
Raytekar et al., 2012	13	127	0.10	[0.06; 0.17]	0.0%	0.1%
Ringo et al., 2015	4	83	0.05	[0.01; 0.12]	0.0%	0.1%
Rodrigues et al., 2008	107	582	0.18	[0.15; 0.22]	0.0%	0.1%
Roka et al., 2012	154	260	0.59	[0.53; 0.65]	0.0%	0.1%
Roka et al., 2013	135	273	0.49	[0.43; 0.56]	0.0%	0.1%
Samie et al., 2014	40	151	0.26	[0.20; 0.34]	0.0%	0.1%
Sanyaolu et al., 2011	11	65	0.17	[0.09; 0.28]	0.0%	0.1%
Sarfati et al., 2006	7	154	0.05	[0.02; 0.09]	0.0%	0.1%
Sherchan et al., 2012	8	146	0.05	[0.02; 0.11]	0.0%	0.1%
Shimelis et al., 2016	50	491	0.10	[0.08; 0.13]	0.1%	0.1%
Sintayehu et al., 2013	45	343	0.13	[0.10; 0.17]	0.0%	0.1%
Sonali et al., 2014	10	250	0.04	[0.02; 0.07]	0.1%	0.1%
Stark et al., 2007	47	628	0.07	[0.06; 0.10]	0.1%	0.1%
Stensvold et al., 2011	10	96	0.10	[0.05; 0.18]	0.0%	0.1%
Suryawanshi et al., 2012	9	100	0.09	[0.04; 0.16]	0.0%	0.1%
Tamirat et al., 2017	23	226	0.10	[0.07; 0.15]	0.0%	0.1%
Tegegne et al., 2017	19	223	0.09	[0.05; 0.13]	0.0%	0.1%
Tian et al., 2012	5	302	0.02	[0.01; 0.04]	0.2%	0.1%
Tian et al., 2013	3	79	0.04	[0.01; 0.11]	0.0%	0.1%
Tigist et al., 2015	7	209	0.03	[0.01; 0.07]	0.1%	0.1%
Tiwari et al. 2013	19	745	0.03	[0.02; 0.04]	0.3%	0.1%
Tuli et al., 2008	11	366	0.03	[0.02; 0.05]	0.1%	0.1%
Vouking et al., 2014	46	207	0.22	[0.17; 0.29]	0.0%	0.1%
Vyas et al., 2013	4	75	0.05	[0.01; 0.13]	0.0%	0.1%
Wanyiri et al., 2013	15	164	0.09	[0.05; 0.15]	0.0%	0.1%
Wumba et al., 2010	9	175	0.05	[0.02; 0.10]	0.0%	0.1%
Wumba et al., 2012	23	242	0.10	[0.06; 0.14]	0.0%	0.1%
Yosefi et al., 2012	7	60	0.12	[0.05; 0.23]	0.0%	0.1%
Zeynudin et al., 2013	9	91	0.10	[0.05; 0.18]	0.0%	0.1%
Gebrecherkos et al., 2019	31	150	0.21	[0.14; 0.28]	0.0%	0.1%
Sannella et al., 2019	2	155	0.01	[0.00; 0.05]	0.1%	0.1%
Abange et al., 2020	4	119	0.03	[0.01; 0.08]	0.0%	0.1%
Alemayehu et al., 2020	7	383	0.02	[0.01; 0.04]	0.2%	0.1%
Belay et al., 2020	8	112	0.07	[0.03; 0.14]	0.0%	0.1%
Chhangte et al., 2020	32	250	0.13	[0.09; 0.18]	0.0%	0.1%
Lengongo et al., 2020	17	115	0.15	[0.09; 0.23]	0.0%	0.1%
Namaji et al., 2020	2	361	0.01	[0.00; 0.02]	0.6%	0.1%
Purbey., 2021	4	75	0.05	[0.01; 0.13]	0.0%	0.1%
Ajayi et al., 2021	64	375	0.17	[0.13; 0.21]	0.0%	0.1%
Bayleyegn et al., 2021	25	255	0.10	[0.06; 0.14]	0.0%	0.1%
Betancourth et al., 2021	5	102	0.05	[0.02; 0.11]	0.0%	0.1%
Botero et al., 2021	25	192	0.13	[0.09; 0.19]	0.0%	0.1%
Chandi et al., 2021	5	80	0.06	[0.02; 0.14]	0.0%	0.1%
Feleke et al., 2022	12	223	0.05	[0.03; 0.09]	0.0%	0.1%
Miressa et al., 2021	23	230	0.10	[0.06; 0.15]	0.0%	0.1%
Barreto et al., 2022	12	178	0.07	[0.04; 0.11]	0.0%	0.1%
Wolday et al., 2021	126	751	0.17	[0.14; 0.20]	0.1%	0.1%
Common effect model		24956	0.02	[0.02; 0.02]	13.0%	--
Random effects model			0.12	[0.10; 0.15]	--	15.2%
Heterogeneity: $I^2 = 97\%$, $\tau^2 = 0.0129$, $p = 0$						
**Protozoa_Genus = Giardia**						
Abaver et al., 2011	3	85	0.04	[0.01; 0.10]	0.0%	0.1%
Abilo and Afework, 2005	6	70	0.09	[0.03; 0.18]	0.0%	0.1%
Adamu and Petros, 2009	32	200	0.16	[0.11; 0.22]	0.0%	0.1%
Agholi et al., 2013	23	356	0.06	[0.04; 0.10]	0.1%	0.1%
Ahmed and Abhay, 2015	5	142	0.04	[0.01; 0.08]	0.0%	0.1%
Akinbo et al., 2012	2	2000	0.00	[0.00; 0.00]	18.1%	0.1%
Asma et al., 2011	11	346	0.03	[0.02; 0.06]	0.1%	0.1%
Assefa et al., 2009	24	214	0.11	[0.07; 0.16]	0.0%	0.1%
Assefa et al., 2013	20	272	0.07	[0.05; 0.11]	0.0%	0.1%
Babatunde et al., 2010	16	90	0.18	[0.11; 0.27]	0.0%	0.1%
Barcelos et al., 2018	1	90	0.01	[0.00; 0.06]	0.1%	0.1%
Berenji et al., 2010	7	31	0.23	[0.10; 0.41]	0.0%	0.1%
Cardoso et al., 2011	5	500	0.01	[0.00; 0.02]	0.5%	0.1%
da Silva et al., 2005	1	100	0.01	[0.00; 0.05]	0.1%	0.1%
Daryani et al., 2009	2	64	0.03	[0.00; 0.11]	0.0%	0.1%
Dash et al., 2013	1	115	0.01	[0.00; 0.05]	0.1%	0.1%
Eshetu et al., 2017	4	223	0.02	[0.00; 0.05]	0.1%	0.1%
Gaurav and Atul, 2015	2	40	0.05	[0.01; 0.17]	0.0%	0.1%
Gebretsadik et al., 2018	3	223	0.01	[0.00; 0.04]	0.2%	0.1%
Getaneh et al., 2018	4	220	0.02	[0.00; 0.05]	0.1%	0.1%
Guk et al., 2005	1	105	0.01	[0.00; 0.05]	0.1%	0.1%
Gupta et al., 2013	8	100	0.08	[0.04; 0.15]	0.0%	0.1%
Haileeyesus et al., 2013	15	378	0.04	[0.02; 0.06]	0.1%	0.1%
Huibers et al., 2018	9	35	0.26	[0.12; 0.43]	0.0%	0.1%
Jaiswal et al., 2014	7	103	0.07	[0.03; 0.14]	0.0%	0.1%
Jayalakshmi et al., 2008	3	89	0.03	[0.01; 0.10]	0.0%	0.1%
Javid et al., 2005	45	200	0.22	[0.17; 0.29]	0.0%	0.1%
Kipyegen et al., 2012	25	285	0.09	[0.06; 0.13]	0.0%	0.1%
Kiros et al., 2015	17	399	0.04	[0.03; 0.07]	0.1%	0.1%
Kurniawan et al., 2009	16	318	0.05	[0.03; 0.08]	0.1%	0.1%
Mahmud et al., 2014	47	372	0.13	[0.09; 0.16]	0.0%	0.1%
Mariam et al., 2008	11	109	0.10	[0.05; 0.17]	0.0%	0.1%
Meamar et al., 2007	33	781	0.04	[0.03; 0.06]	0.2%	0.1%
Mehta et al., 2013	5	100	0.05	[0.02; 0.11]	0.0%	0.1%
Missaye et al., 2013	20	272	0.07	[0.05; 0.11]	0.0%	0.1%
Mitra et al., 2016	20	194	0.10	[0.06; 0.15]	0.0%	0.1%
Moges et al., 2006	2	28	0.07	[0.01; 0.24]	0.0%	0.1%
Moran et al., 2005	4	203	0.02	[0.01; 0.05]	0.1%	0.1%
Morawski et al., 2017	12	202	0.06	[0.03; 0.10]	0.0%	0.1%
Mtapuri et al., 2014	2	29	0.07	[0.01; 0.23]	0.0%	0.1%
Navarro et al., 2006	4	103	0.04	[0.01; 0.10]	0.0%	0.1%
Nkenfou et al., 2013	1	42	0.02	[0.00; 0.13]	0.0%	0.1%
Nkenfou et al., 2017	2	68	0.03	[0.00; 0.10]	0.0%	0.1%
Nsagha et al., 2016	10	300	0.03	[0.02; 0.06]	0.1%	0.1%
Nuhu et al., 2014	13	205	0.06	[0.03; 0.11]	0.0%	0.1%
Oguntibeju 2006	5	60	0.08	[0.03; 0.18]	0.0%	0.1%
Paboriboune et al., 2014	12	137	0.09	[0.05; 0.15]	0.0%	0.1%
Patel et al., 2011	2	100	0.02	[0.00; 0.07]	0.0%	0.1%
Pavie et al., 2012	7	143	0.05	[0.02; 0.10]	0.0%	0.1%
Pennap et al., 2015	12	200	0.06	[0.03; 0.10]	0.0%	0.1%
Pinlaor et al., 2005	1	78	0.01	[0.00; 0.07]	0.0%	0.1%
Ramakrishnan et al., 2007	3	80	0.04	[0.01; 0.11]	0.0%	0.1%
Ramana et al., 2010	34	452	0.08	[0.05; 0.10]	0.1%	0.1%
Raytekar et al., 2012	3	127	0.02	[0.00; 0.07]	0.1%	0.1%
Ringo et al., 2015	8	83	0.10	[0.04; 0.18]	0.0%	0.1%
Rodrigues et al., 2008	39	582	0.07	[0.05; 0.09]	0.1%	0.1%
Roka et al., 2012	32	260	0.12	[0.09; 0.17]	0.0%	0.1%
Roka et al., 2013	14	273	0.05	[0.03; 0.08]	0.1%	0.1%
Samie et al., 2014	18	151	0.12	[0.07; 0.18]	0.0%	0.1%
Sanyaolu et al., 2011	6	65	0.09	[0.03; 0.19]	0.0%	0.1%
Sarfati et al., 2006	1	154	0.01	[0.00; 0.04]	0.2%	0.1%
Sherchan et al., 2012	14	146	0.10	[0.05; 0.16]	0.0%	0.1%
Sherpa et al., 2010	3	106	0.03	[0.01; 0.08]	0.0%	0.1%
Shimelis et al., 2016	39	491	0.08	[0.06; 0.11]	0.1%	0.1%
Silva et al., 2005	1	100	0.01	[0.00; 0.05]	0.1%	0.1%
Sintayehu et al., 2013	21	343	0.06	[0.04; 0.09]	0.1%	0.1%
Sonali et al., 2014	4	250	0.02	[0.00; 0.04]	0.2%	0.1%
Stark et al., 2007	28	628	0.04	[0.03; 0.06]	0.2%	0.1%
Stensvold et al., 2011	2	96	0.02	[0.00; 0.07]	0.0%	0.1%
Suryawanshi et al., 2012	6	100	0.06	[0.02; 0.13]	0.0%	0.1%
Tamirat et al., 2017	8	226	0.04	[0.02; 0.07]	0.1%	0.1%
Tegegne et al., 2017	4	223	0.02	[0.00; 0.05]	0.1%	0.1%
Tian et al., 2012	4	302	0.01	[0.00; 0.03]	0.2%	0.1%
Tian et al., 2013	1	79	0.01	[0.00; 0.07]	0.0%	0.1%
Tigist et al., 2015	31	209	0.15	[0.10; 0.20]	0.0%	0.1%
Tiwari et al. 2013	26	745	0.03	[0.02; 0.05]	0.2%	0.1%
Tuli et al., 2008	37	366	0.10	[0.07; 0.14]	0.0%	0.1%
Vyas et al., 2013	3	75	0.04	[0.01; 0.11]	0.0%	0.1%
Wanyiri et al., 2013	13	164	0.08	[0.04; 0.13]	0.0%	0.1%
Wumba et al., 2010	3	175	0.02	[0.00; 0.05]	0.1%	0.1%
Wumba et al., 2012	2	242	0.01	[0.00; 0.03]	0.3%	0.1%
Yosefi et al., 2012	3	60	0.05	[0.01; 0.14]	0.0%	0.1%
Zeynudin et al., 2013	8	91	0.09	[0.04; 0.17]	0.0%	0.1%
Gebrecherkos et al., 2019	13	150	0.09	[0.05; 0.14]	0.0%	0.1%
Masoumi et al., 2019	1	102	0.01	[0.00; 0.05]	0.1%	0.1%
Sannella et al., 2019	3	155	0.02	[0.00; 0.06]	0.1%	0.1%
Abange et al., 2020	5	119	0.04	[0.01; 0.10]	0.0%	0.1%
Alemayehu et al., 2020	8	383	0.02	[0.01; 0.04]	0.2%	0.1%
Ayele et al., 2020	16	180	0.09	[0.05; 0.14]	0.0%	0.1%
Belay et al., 2020	4	112	0.04	[0.01; 0.09]	0.0%	0.1%
Chhangte et al., 2020	7	250	0.03	[0.01; 0.06]	0.1%	0.1%
Lengongo et al., 2020	2	115	0.02	[0.00; 0.06]	0.1%	0.1%
Namaji et al., 2020	1	361	0.00	[0.00; 0.02]	1.0%	0.1%
Purbey., 2021	1	75	0.01	[0.00; 0.07]	0.0%	0.1%
Bayleyegn et al., 2021	3	255	0.01	[0.00; 0.03]	0.2%	0.1%
Betancourth et al., 2021	2	102	0.02	[0.00; 0.07]	0.0%	0.1%
Feleke et al., 2022	4	223	0.02	[0.00; 0.05]	0.1%	0.1%
Miressa et al., 2021	50	230	0.22	[0.17; 0.28]	0.0%	0.1%
Barreto et al., 2022	5	178	0.03	[0.01; 0.06]	0.1%	0.1%
Wolday et al., 2021	27	751	0.04	[0.02; 0.05]	0.2%	0.1%
Common effect model		22304	0.01	[0.01; 0.01]	25.8%	--
Random effects model			0.05	[0.04; 0.06]	--	14.2%
Heterogeneity: $I^2 = 90\%$, $\tau^2 = 0.0013$, $p < 0.01$						
**Protozoa_Genus = Isospora**						
Abilo and Afework, 2005	1	70	0.01	[0.00; 0.08]	0.0%	0.1%
Adamu and Petros, 2009	10	200	0.05	[0.02; 0.09]	0.0%	0.1%
Agholi et al., 2013	2	356	0.01	[0.00; 0.02]	0.6%	0.1%
Ahmed and Abhay, 2015	22	142	0.15	[0.10; 0.23]	0.0%	0.1%
Akinbo et al., 2011	16	463	0.03	[0.02; 0.06]	0.2%	0.1%
Akinbo et al., 2012	21	2000	0.01	[0.01; 0.02]	2.1%	0.1%
Archana et al., 2017	17	104	0.16	[0.10; 0.25]	0.0%	0.1%
Asma et al., 2011	35	346	0.10	[0.07; 0.14]	0.0%	0.1%
Assefa et al., 2009	26	214	0.12	[0.08; 0.17]	0.0%	0.1%
Assefa et al., 2013	1	272	0.00	[0.00; 0.02]	0.6%	0.1%
Babatunde et al., 2010	10	90	0.11	[0.05; 0.19]	0.0%	0.1%
Cardoso et al., 2011	6	500	0.01	[0.00; 0.03]	0.4%	0.1%
da Silva et al., 2005	7	100	0.07	[0.03; 0.14]	0.0%	0.1%
Dash et al., 2013	19	115	0.17	[0.10; 0.25]	0.0%	0.1%
Djieyep et al., 2014	95	305	0.31	[0.26; 0.37]	0.0%	0.1%
Echoru et al., 2015	2	111	0.02	[0.00; 0.06]	0.1%	0.1%
Gaurav and Atul, 2015	4	40	0.10	[0.03; 0.24]	0.0%	0.1%
Gebretsadik et al., 2018	1	223	0.00	[0.00; 0.02]	0.4%	0.1%
Getaneh et al., 2018	3	220	0.01	[0.00; 0.04]	0.2%	0.1%
Girma et al., 2014	4	268	0.01	[0.00; 0.04]	0.2%	0.1%
Guk et al., 2005	5	105	0.05	[0.02; 0.11]	0.0%	0.1%
Gupta et al., 2013	10	100	0.10	[0.05; 0.18]	0.0%	0.1%
Haileeyesus et al., 2013	6	378	0.02	[0.01; 0.03]	0.3%	0.1%
Jaiswal et al., 2014	3	103	0.03	[0.01; 0.08]	0.0%	0.1%
Jayalakshmi et al., 2008	4	89	0.04	[0.01; 0.11]	0.0%	0.1%
Kammili et al., 2008	28	175	0.16	[0.11; 0.22]	0.0%	0.1%
Kiros et al., 2015	5	399	0.01	[0.00; 0.03]	0.3%	0.1%
Kulkarni et al., 2009	11	137	0.08	[0.04; 0.14]	0.0%	0.1%
Kurniawan et al., 2009	2	318	0.01	[0.00; 0.02]	0.5%	0.1%
Lehman et al., 2013	1	201	0.00	[0.00; 0.03]	0.3%	0.1%
Mariam et al., 2008	2	109	0.02	[0.00; 0.06]	0.1%	0.1%
Mathur et al., 2013	42	544	0.08	[0.06; 0.10]	0.1%	0.1%
Meamar et al., 2007	2	781	0.00	[0.00; 0.01]	2.8%	0.1%
Mehta et al., 2013	18	100	0.18	[0.11; 0.27]	0.0%	0.1%
Mekonnen et al., 2014	4	268	0.01	[0.00; 0.04]	0.2%	0.1%
Missaye et al., 2013	1	272	0.00	[0.00; 0.02]	0.6%	0.1%
Mitra et al., 2016	28	194	0.14	[0.10; 0.20]	0.0%	0.1%
Mohan et al., 2014	11	150	0.07	[0.04; 0.13]	0.0%	0.1%
Moran et al., 2005	1	203	0.00	[0.00; 0.03]	0.3%	0.1%
Navarro et al., 2006	2	103	0.02	[0.00; 0.07]	0.0%	0.1%
Nsagha et al., 2016	13	300	0.04	[0.02; 0.07]	0.1%	0.1%
Oguntibeju 2006	2	60	0.03	[0.00; 0.12]	0.0%	0.1%
Patel et al., 2011	3	100	0.03	[0.01; 0.09]	0.0%	0.1%
Pavie et al., 2012	1	143	0.01	[0.00; 0.04]	0.2%	0.1%
Pennap et al., 2015	7	200	0.04	[0.01; 0.07]	0.1%	0.1%
Pinlaor et al., 2005	1	78	0.01	[0.00; 0.07]	0.0%	0.1%
Ramakrishnan et al., 2007	1	80	0.01	[0.00; 0.07]	0.1%	0.1%
Ramana et al., 2010	46	452	0.10	[0.08; 0.13]	0.1%	0.1%
Rodrigues et al., 2008	24	582	0.04	[0.03; 0.06]	0.2%	0.1%
Saksirisampant et al., 2009	1	90	0.01	[0.00; 0.06]	0.1%	0.1%
Sanyaolu et al., 2011	2	65	0.03	[0.00; 0.11]	0.0%	0.1%
Sarfati et al., 2006	3	154	0.02	[0.00; 0.06]	0.1%	0.1%
Sherchan et al., 2012	3	146	0.02	[0.00; 0.06]	0.1%	0.1%
Shimelis et al., 2016	11	491	0.02	[0.01; 0.04]	0.2%	0.1%
Silva et al., 2005	7	100	0.07	[0.03; 0.14]	0.0%	0.1%
Sintayehu et al., 2013	2	343	0.01	[0.00; 0.02]	0.5%	0.1%
Sonali et al., 2014	2	250	0.01	[0.00; 0.03]	0.3%	0.1%
Stensvold et al., 2011	1	96	0.01	[0.00; 0.06]	0.1%	0.1%
Suryawanshi et al., 2012	13	100	0.13	[0.07; 0.21]	0.0%	0.1%
Tuli et al., 2008	2	366	0.01	[0.00; 0.02]	0.6%	0.1%
Vouking et al., 2014	12	207	0.06	[0.03; 0.10]	0.0%	0.1%
Vyas et al., 2013	9	75	0.12	[0.06; 0.22]	0.0%	0.1%
Wanyiri et al., 2013	18	164	0.11	[0.07; 0.17]	0.0%	0.1%
Wumba et al., 2010	3	175	0.02	[0.00; 0.05]	0.1%	0.1%
Zeynudin et al., 2013	6	91	0.07	[0.02; 0.14]	0.0%	0.1%
Gebrecherkos et al., 2019	3	150	0.02	[0.00; 0.06]	0.1%	0.1%
Sannella et al., 2019	4	155	0.03	[0.01; 0.06]	0.1%	0.1%
Lengongo et al., 2020	1	115	0.01	[0.00; 0.05]	0.1%	0.1%
Ajayi et al., 2021	5	375	0.01	[0.00; 0.03]	0.3%	0.1%
Feleke et al., 2022	2	223	0.01	[0.00; 0.03]	0.2%	0.1%
Common effect model		16794	0.01	[0.01; 0.01]	14.0%	--
Random effects model			0.05	[0.03; 0.06]	--	10.0%
Heterogeneity: $I^2 = 88\%$, $\tau^2 = 0.0024$, $p < 0.01$						
**Protozoa_Genus = Blastocystis**						
Adamu and Petros, 2009	3	200	0.01	[0.00; 0.04]	0.1%	0.1%
Agholi et al., 2013	14	356	0.04	[0.02; 0.07]	0.1%	0.1%
Barcelos et al., 2018	2	90	0.02	[0.00; 0.08]	0.0%	0.1%
Berenji et al., 2010	7	31	0.23	[0.10; 0.41]	0.0%	0.1%
Feyisayo et al., 2014	1	105	0.01	[0.00; 0.05]	0.1%	0.1%
Gupta et al., 2013	1	100	0.01	[0.00; 0.05]	0.1%	0.1%
Haileeyesus et al., 2013	24	378	0.06	[0.04; 0.09]	0.1%	0.1%
Jegede et al., 2014	1	105	0.01	[0.00; 0.05]	0.1%	0.1%
Kurniawan et al., 2009	234	318	0.74	[0.68; 0.78]	0.0%	0.1%
Mariam et al., 2008	2	109	0.02	[0.00; 0.06]	0.1%	0.1%
Meamar et al., 2007	48	781	0.06	[0.05; 0.08]	0.2%	0.1%
Mohan et al., 2014	1	150	0.01	[0.00; 0.04]	0.2%	0.1%
Moran et al., 2005	4	203	0.02	[0.01; 0.05]	0.1%	0.1%
Mtapuri et al., 2014	1	29	0.03	[0.00; 0.18]	0.0%	0.1%
Nkenfou et al., 2017	30	68	0.44	[0.32; 0.57]	0.0%	0.1%
Nsagha et al., 2016	75	300	0.25	[0.20; 0.30]	0.0%	0.1%
Oguntibeju 2006	2	60	0.03	[0.00; 0.12]	0.0%	0.1%
Paboriboune et al., 2014	36	137	0.26	[0.19; 0.34]	0.0%	0.1%
Pennap et al., 2015	11	200	0.06	[0.03; 0.10]	0.0%	0.1%
Pinlaor et al., 2005	1	78	0.01	[0.00; 0.07]	0.0%	0.1%
Ramana et al., 2010	139	452	0.31	[0.27; 0.35]	0.0%	0.1%
Roka et al., 2012	28	260	0.11	[0.07; 0.15]	0.0%	0.1%
Roka et al., 2013	16	273	0.06	[0.03; 0.09]	0.1%	0.1%
Saksirisampant et al., 2009	2	90	0.02	[0.00; 0.08]	0.0%	0.1%
Samie et al., 2014	3	151	0.02	[0.00; 0.06]	0.1%	0.1%
Sherchan et al., 2012	9	146	0.06	[0.03; 0.11]	0.0%	0.1%
Stark et al., 2007	111	628	0.18	[0.15; 0.21]	0.0%	0.1%
Stensvold et al., 2011	22	96	0.23	[0.15; 0.33]	0.0%	0.1%
Tamirat et al., 2017	14	226	0.06	[0.03; 0.10]	0.0%	0.1%
Tian et al., 2012	49	302	0.16	[0.12; 0.21]	0.0%	0.1%
Tian et al., 2013	11	79	0.14	[0.07; 0.24]	0.0%	0.1%
Tiwari et al. 2013	2	745	0.00	[0.00; 0.01]	2.5%	0.1%
Yosefi et al., 2012	2	60	0.03	[0.00; 0.12]	0.0%	0.1%
Masoumi et al., 2019	2	102	0.02	[0.00; 0.07]	0.0%	0.1%
Abange et al., 2020	3	119	0.03	[0.01; 0.07]	0.0%	0.1%
Chhangte et al., 2020	17	250	0.07	[0.04; 0.11]	0.0%	0.1%
Lengongo et al., 2020	26	115	0.23	[0.15; 0.31]	0.0%	0.1%
Yulfi et al., 2021	5	54	0.09	[0.03; 0.20]	0.0%	0.1%
Betancourth et al., 2021	11	102	0.11	[0.06; 0.18]	0.0%	0.1%
Botero et al., 2021	23	192	0.12	[0.08; 0.17]	0.0%	0.1%
Common effect model		8240	0.02	[0.02; 0.02]	4.3%	--
Random effects model			0.11	[0.06; 0.15]	--	5.6%
Heterogeneity: $I^2 = 98\%$, $\tau^2 = 0.0191$, $p < 0.01$						
**Protozoa_Genus = Cyclospora**						
Agholi et al., 2013	1	356	0.00	[0.00; 0.02]	1.0%	0.1%
Ahmed and Abhay, 2015	3	142	0.02	[0.00; 0.06]	0.1%	0.1%
Asma et al., 2011	17	346	0.05	[0.03; 0.08]	0.1%	0.1%
Babatunde et al., 2010	16	90	0.18	[0.11; 0.27]	0.0%	0.1%
Dash et al., 2013	1	115	0.01	[0.00; 0.05]	0.1%	0.1%
Eshetu et al., 2017	6	223	0.03	[0.01; 0.06]	0.1%	0.1%
Getaneh et al., 2018	13	220	0.06	[0.03; 0.10]	0.0%	0.1%
Jaiswal et al., 2014	2	103	0.02	[0.00; 0.07]	0.0%	0.1%
Kammili et al., 2008	2	175	0.01	[0.00; 0.04]	0.1%	0.1%
Kulkarni et al., 2009	1	137	0.01	[0.00; 0.04]	0.1%	0.1%
Kurniawan et al., 2009	21	318	0.07	[0.04; 0.10]	0.1%	0.1%
Lehman et al., 2013	1	201	0.00	[0.00; 0.03]	0.3%	0.1%
Mathur et al., 2013	12	544	0.02	[0.01; 0.04]	0.3%	0.1%
Mitra et al., 2016	2	194	0.01	[0.00; 0.04]	0.2%	0.1%
Mohan et al., 2014	1	150	0.01	[0.00; 0.04]	0.2%	0.1%
Mtapuri et al., 2014	1	29	0.03	[0.00; 0.18]	0.0%	0.1%
Nkenfou et al., 2017	2	68	0.03	[0.00; 0.10]	0.0%	0.1%
Nsagha et al., 2016	11	300	0.04	[0.02; 0.06]	0.1%	0.1%
Paboriboune et al., 2014	3	137	0.02	[0.00; 0.06]	0.1%	0.1%
Pennap et al., 2015	15	200	0.07	[0.04; 0.12]	0.0%	0.1%
Pinlaor et al., 2005	1	78	0.01	[0.00; 0.07]	0.0%	0.1%
Ramana et al., 2010	27	452	0.06	[0.04; 0.09]	0.1%	0.1%
Saksirisampant et al., 2009	1	90	0.01	[0.00; 0.06]	0.1%	0.1%
Sherchan et al., 2012	6	146	0.04	[0.02; 0.09]	0.0%	0.1%
Sherpa et al., 2010	4	106	0.04	[0.01; 0.09]	0.0%	0.1%
Shimelis et al., 2016	2	491	0.00	[0.00; 0.01]	1.1%	0.1%
Sucilathangam et al., 2012	6	103	0.06	[0.02; 0.12]	0.0%	0.1%
Tegegne et al., 2017	6	223	0.03	[0.01; 0.06]	0.1%	0.1%
Tiwari et al. 2013	14	745	0.02	[0.01; 0.03]	0.4%	0.1%
Tuli et al., 2008	88	366	0.24	[0.20; 0.29]	0.0%	0.1%
Uysal et al., 2017	3	115	0.03	[0.01; 0.07]	0.0%	0.1%
Wanyiri et al., 2013	5	164	0.03	[0.01; 0.07]	0.1%	0.1%
Masoumi et al., 2019	1	102	0.01	[0.00; 0.05]	0.1%	0.1%
Belay et al., 2020	1	112	0.01	[0.00; 0.05]	0.1%	0.1%
Chhangte et al., 2020	1	250	0.00	[0.00; 0.02]	0.5%	0.1%
Namaji et al., 2020	7	361	0.02	[0.01; 0.04]	0.2%	0.1%
Ajayi et al., 2021	2	375	0.01	[0.00; 0.02]	0.6%	0.1%
Common effect model		8327	0.01	[0.01; 0.01]	6.4%	--
Random effects model			0.03	[0.02; 0.04]	--	5.3%
Heterogeneity: $I^2 = 85\%$, $\tau^2 = 0.0012$, $p < 0.01$						
**Protozoa_Genus = Leishmania**						
Almeida et al., 2012	5	10	0.50	[0.19; 0.81]	0.0%	0.1%
Carranza et al., 2009	26	163	0.16	[0.11; 0.22]	0.0%	0.1%
Cavalcanti et al., 2012	5	10	0.50	[0.19; 0.81]	0.0%	0.1%
Colomba et al., 2009	24	145	0.17	[0.11; 0.24]	0.0%	0.1%
Cota et al., 2013	41	113	0.36	[0.27; 0.46]	0.0%	0.1%
Garcia et al., 2006	28	92	0.30	[0.21; 0.41]	0.0%	0.1%
Júnior et al., 2015	7	15	0.47	[0.21; 0.73]	0.0%	0.1%
Oliveira et al., 2011	13	169	0.08	[0.04; 0.13]	0.0%	0.1%
Pandey et al., 2018	176	305	0.58	[0.52; 0.63]	0.0%	0.1%
Rezaei et al., 2018	19	251	0.08	[0.05; 0.12]	0.0%	0.1%
Shafiei et al., 2014	9	49	0.18	[0.09; 0.32]	0.0%	0.1%
Abdullahi et al., 2020	28	58	0.48	[0.35; 0.62]	0.0%	0.1%
Barbosa et al., 2020	65	113	0.58	[0.48; 0.67]	0.0%	0.1%
Barbosa et al., 2020	110	309	0.36	[0.30; 0.41]	0.0%	0.1%
Charoensakulchai et al., 2020	110	526	0.21	[0.18; 0.25]	0.0%	0.1%
Cunha et al., 2020	36	240	0.15	[0.11; 0.20]	0.0%	0.1%
Common effect model		2568	0.21	[0.20; 0.23]	0.2%	--
Random effects model			0.30	[0.22; 0.39]	--	2.1%
Heterogeneity: $I^2 = 96\%$, $\tau^2 = 0.0287$, $p < 0.01$						
**Protozoa_Genus = Trypanosoma**						
Almeida et al., 2010	9	716	0.01	[0.01; 0.02]	0.6%	0.1%
Llenas et al., 2012	4	155	0.03	[0.01; 0.06]	0.1%	0.1%
Rodrigues et al., 2011	2	19	0.11	[0.01; 0.33]	0.0%	0.1%
Rodrigues et al., 2005	18	28	0.64	[0.44; 0.81]	0.0%	0.1%
McAtee et al., 2021	32	116	0.28	[0.20; 0.37]	0.0%	0.1%
Common effect model		1034	0.02	[0.01; 0.03]	0.7%	--
Random effects model			0.20	[0.00; 0.42]	--	0.7%
Heterogeneity: $I^2 = 96\%$, $\tau^2 = 0.0604$, $p < 0.01$						
**Protozoa_Genus = Trichomonas**						
Assefa et al., 2009	2	214	0.01	[0.00; 0.03]	0.2%	0.1%
Davis et al., 2016	16	44	0.36	[0.22; 0.52]	0.0%	0.1%
De Lemos and Garcia-Zapata, 2010	23	125	0.18	[0.12; 0.26]	0.0%	0.1%
Gupta et al., 2013	2	100	0.02	[0.00; 0.07]	0.0%	0.1%
Kissinger et al., 2008	11	60	0.18	[0.10; 0.30]	0.0%	0.1%
Lemos et al., 2009	23	125	0.18	[0.12; 0.26]	0.0%	0.1%
Masese et al., 2011	31	147	0.21	[0.15; 0.29]	0.0%	0.1%
Mtapuri et al., 2014	1	29	0.03	[0.00; 0.18]	0.0%	0.1%
Nangammbi et al., 2013	32	155	0.21	[0.15; 0.28]	0.0%	0.1%
Nwadioha et al., 2012	84	350	0.24	[0.20; 0.29]	0.0%	0.1%
Nweze and Mouneke, 2011	355	970	0.37	[0.34; 0.40]	0.0%	0.1%
Price et al., 2018	76	359	0.21	[0.17; 0.26]	0.0%	0.1%
Stark et al., 2007	2	628	0.00	[0.00; 0.01]	1.8%	0.1%
Abange et al., 2020	1	119	0.01	[0.00; 0.05]	0.1%	0.1%
Medina et al., 2020	86	427	0.20	[0.16; 0.24]	0.0%	0.1%
Betancourth et al., 2021	1	102	0.01	[0.00; 0.05]	0.1%	0.1%
Common effect model		3954	0.02	[0.01; 0.02]	2.4%	--
Random effects model			0.15	[0.09; 0.21]	--	2.2%
Heterogeneity: $I^2 = 98\%$, $\tau^2 = 0.0141$, $p < 0.01$						
**Protozoa_Genus = Iodamoeba**						
Babatunde et al., 2010	7	90	0.08	[0.03; 0.15]	0.0%	0.1%
Barcelos et al., 2018	1	90	0.01	[0.00; 0.06]	0.1%	0.1%
Gupta et al., 2013	1	100	0.01	[0.00; 0.05]	0.1%	0.1%
Moran et al., 2005	3	203	0.01	[0.00; 0.04]	0.1%	0.1%
Mtapuri et al., 2014	1	29	0.03	[0.00; 0.18]	0.0%	0.1%
Ngum et al., 2015	7	320	0.02	[0.01; 0.04]	0.2%	0.1%
Nkenfou et al., 2017	3	68	0.04	[0.01; 0.12]	0.0%	0.1%
Patel et al., 2011	1	100	0.01	[0.00; 0.05]	0.1%	0.1%
Rodrigues et al., 2008	37	582	0.06	[0.05; 0.09]	0.1%	0.1%
Roka et al., 2012	1	260	0.00	[0.00; 0.02]	0.5%	0.1%
Roka et al., 2013	16	273	0.06	[0.03; 0.09]	0.1%	0.1%
Sanyaolu et al., 2011	2	65	0.03	[0.00; 0.11]	0.0%	0.1%
Stark et al., 2007	8	628	0.01	[0.01; 0.02]	0.5%	0.1%
Wumba et al., 2012	1	242	0.00	[0.00; 0.02]	0.4%	0.1%
Abange et al., 2020	2	119	0.02	[0.00; 0.06]	0.1%	0.1%
Lengongo et al., 2020	1	115	0.01	[0.00; 0.05]	0.1%	0.1%
Betancourth et al., 2021	1	102	0.01	[0.00; 0.05]	0.1%	0.1%
Barreto et al., 2022	2	178	0.01	[0.00; 0.04]	0.1%	0.1%
Common effect model		3564	0.01	[0.01; 0.02]	2.6%	--
Random effects model			0.02	[0.01; 0.03]	--	2.6%
Heterogeneity: $I^2 = 69\%$, $\tau^2 = 0.0002$, $p < 0.01$						
**Protozoa_Genus = Endolimax**						
Barcelos et al., 2018	15	90	0.17	[0.10; 0.26]	0.0%	0.1%
Cardoso et al., 2011	3	500	0.01	[0.00; 0.02]	0.8%	0.1%
Gupta et al., 2013	2	100	0.02	[0.00; 0.07]	0.0%	0.1%
Moran et al., 2005	13	203	0.06	[0.03; 0.11]	0.0%	0.1%
Mtapuri et al., 2014	1	29	0.03	[0.00; 0.18]	0.0%	0.1%
Rodrigues et al., 2008	38	582	0.07	[0.05; 0.09]	0.1%	0.1%
Roka et al., 2012	13	260	0.05	[0.03; 0.08]	0.1%	0.1%
Roka et al., 2013	58	273	0.21	[0.17; 0.27]	0.0%	0.1%
Stark et al., 2007	62	628	0.10	[0.08; 0.12]	0.1%	0.1%
Tamirat et al., 2017	1	226	0.00	[0.00; 0.02]	0.4%	0.1%
Wumba et al., 2012	4	242	0.02	[0.00; 0.04]	0.2%	0.1%
Yosefi et al., 2012	5	60	0.08	[0.03; 0.18]	0.0%	0.1%
Abange et al., 2020	2	119	0.02	[0.00; 0.06]	0.1%	0.1%
Betancourth et al., 2021	7	102	0.07	[0.03; 0.14]	0.0%	0.1%
Barreto et al., 2022	18	178	0.10	[0.06; 0.16]	0.0%	0.1%
Common effect model		3592	0.02	[0.02; 0.02]	1.8%	--
Random effects model			0.06	[0.03; 0.09]	--	2.1%
Heterogeneity: $I^2 = 93\%$, $\tau^2 = 0.0028$, $p < 0.01$						
**Protozoa_Genus = Cystoisospora**						
Barcelos et al., 2018	1	90	0.01	[0.00; 0.06]	0.1%	0.1%
Ngum et al., 2015	6	320	0.02	[0.01; 0.04]	0.2%	0.1%
Paboriboune et al., 2014	6	137	0.04	[0.02; 0.09]	0.0%	0.1%
Gebrecherkos et al., 2019	2	150	0.01	[0.00; 0.05]	0.1%	0.1%
Chhangte et al., 2020	3	250	0.01	[0.00; 0.03]	0.2%	0.1%
Namaji et al., 2020	30	361	0.08	[0.06; 0.12]	0.1%	0.1%
Betancourth et al., 2021	1	102	0.01	[0.00; 0.05]	0.1%	0.1%
Botero et al., 2021	1	192	0.01	[0.00; 0.03]	0.3%	0.1%
Common effect model		1602	0.01	[0.01; 0.02]	1.0%	--
Random effects model			0.02	[0.01; 0.04]	--	1.1%
Heterogeneity: $I^2 = 76\%$, $\tau^2 = 0.0004$, $p < 0.01$						
**Protozoa_Genus = Chilomastix**						
Berenji et al., 2010	7	31	0.23	[0.10; 0.41]	0.0%	0.1%
Daryani et al., 2009	1	64	0.02	[0.00; 0.08]	0.0%	0.1%
Gupta et al., 2013	1	100	0.01	[0.00; 0.05]	0.1%	0.1%
Rodrigues et al., 2008	3	582	0.01	[0.00; 0.01]	1.1%	0.1%
Roka et al., 2012	11	260	0.04	[0.02; 0.07]	0.1%	0.1%
Roka et al., 2013	3	273	0.01	[0.00; 0.03]	0.2%	0.1%
Stark et al., 2007	3	628	0.00	[0.00; 0.01]	1.3%	0.1%
Lengongo et al., 2020	5	115	0.04	[0.01; 0.10]	0.0%	0.1%
Betancourth et al., 2021	1	102	0.01	[0.00; 0.05]	0.1%	0.1%
Barreto et al., 2022	1	178	0.01	[0.00; 0.03]	0.2%	0.1%
Common effect model		2333	0.01	[0.00; 0.01]	3.2%	--
Random effects model			0.01	[0.00; 0.01]	--	1.4%
Heterogeneity: $I^2 = 59\%$, $\tau^2 = 0$, $p < 0.01$						
**Common effect model**		**168080**	**0.08**	**[0.08; 0.08]**	**100.0%**	**--**
**Random effects model**			**0.16**	**[0.14; 0.17]**	**--**	**100.0%**
Heterogeneity: $I^2 = 100\%$, $\tau^2 = 0.0407$, $p = 0$						
Test for subgroup differences (fixed effect): $\chi^2_{14} = 325328.29$, df = 14 ($p = 0$)						
Test for subgroup differences (random effects): $\chi^2_{14} = 655.23$, df = 14 ($p < 0.01$)						

0.2 0.4 0.6 0.8 1

Figure S3A: Forest plots of the prevalence rates of helminth co-infections in virus-infected people at species level by meta-analysis using common effect and random-effects models

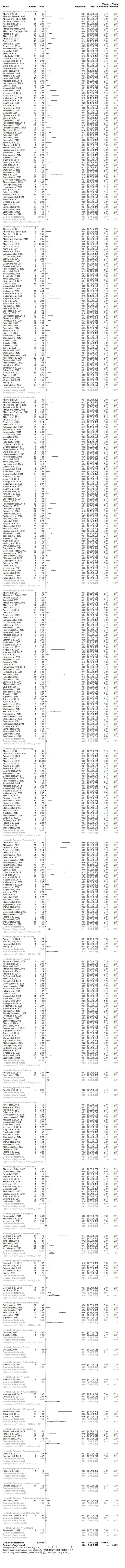

Figure S3B: Forest plots of the prevalence rates of protozoan co-infections in virus-infected people at species level by meta-analysis using common effect and random-effects models

Study	Events	Total	Proportion	95%-CI	Weight (common)	Weight (random)
**Protozoa_Species = E. histolytica**						
Abaver et al., 2011	6	85	0.07	[0.03; 0.15]	0.0%	0.1%
Abdollahi et al., 2015	28	91	0.31	[0.22; 0.41]	0.0%	0.1%
Abilo and Afework, 2005	3	70	0.04	[0.01; 0.12]	0.0%	0.1%
Adesij et al. 2007	6	100	0.06	[0.02; 0.13]	0.0%	0.1%
Akinbo et al., 2011	5	463	0.01	[0.00; 0.03]	0.2%	0.1%
Akinbo et al., 2012	6	2000	0.00	[0.00; 0.01]	3.5%	0.1%
Babatunde et al., 2010	19	90	0.21	[0.13; 0.31]	0.0%	0.1%
Daryani et al., 2009	1	64	0.02	[0.00; 0.08]	0.0%	0.1%
Feyisayo et al., 2014	6	105	0.06	[0.02; 0.12]	0.0%	0.1%
Gaurav and Atul, 2015	2	40	0.05	[0.01; 0.17]	0.0%	0.1%
Gebretsadik et al., 2018	16	223	0.07	[0.04; 0.11]	0.0%	0.1%
Gupta et al., 2013	4	100	0.04	[0.01; 0.10]	0.0%	0.1%
Hung et al., 2008	66	991	0.07	[0.05; 0.08]	0.1%	0.1%
Jayalakshmi et al., 2008	1	89	0.01	[0.00; 0.06]	0.0%	0.1%
Javid et al., 2005	35	200	0.17	[0.13; 0.23]	0.0%	0.1%
Jegede et al., 2014	6	105	0.06	[0.02; 0.12]	0.0%	0.1%
Lar et al., 2016	18	205	0.09	[0.05; 0.14]	0.0%	0.1%
Mariam et al., 2008	21	109	0.19	[0.12; 0.28]	0.0%	0.1%
Mathur et al., 2013	49	544	0.09	[0.07; 0.12]	0.0%	0.1%
Moges et al., 2006	1	28	0.04	[0.00; 0.18]	0.0%	0.1%
Moran et al., 2009	10	100	0.10	[0.05; 0.18]	0.0%	0.1%
Moran et al., 2005	40	203	0.20	[0.14; 0.26]	0.0%	0.1%
Morawski et al., 2017	3	202	0.01	[0.00; 0.04]	0.1%	0.1%
Mtapuri et al., 2014	3	29	0.10	[0.02; 0.27]	0.0%	0.1%
Ngum et al., 2015	36	320	0.11	[0.08; 0.15]	0.0%	0.1%
Nkenfou et al., 2013	8	42	0.19	[0.09; 0.34]	0.0%	0.1%
Nsagha et al., 2016	22	300	0.07	[0.05; 0.11]	0.0%	0.1%
Nuhu et al., 2014	2	205	0.01	[0.00; 0.03]	0.1%	0.1%
Oguntibeju 2006	4	60	0.07	[0.02; 0.16]	0.0%	0.1%
Ojurongbe et al., 2011	4	96	0.04	[0.01; 0.10]	0.0%	0.1%
Patel et al., 2011	2	100	0.02	[0.00; 0.07]	0.0%	0.1%
Pennap et al., 2015	20	200	0.10	[0.06; 0.15]	0.0%	0.1%
Ramakrishnan et al., 2007	14	80	0.17	[0.10; 0.28]	0.0%	0.1%
Ramana et al., 2010	42	452	0.09	[0.07; 0.12]	0.0%	0.1%
Raytekar et al., 2012	13	127	0.10	[0.06; 0.17]	0.0%	0.1%
Ringo et al., 2015	4	83	0.05	[0.01; 0.12]	0.0%	0.1%
Sherchan et al., 2012	8	146	0.05	[0.02; 0.11]	0.0%	0.1%
Shimelis et al., 2016	50	491	0.10	[0.08; 0.13]	0.0%	0.1%
Sintayehu et al., 2013	45	343	0.13	[0.10; 0.17]	0.0%	0.1%
Sonali et al., 2014	10	250	0.04	[0.02; 0.07]	0.0%	0.1%
Suryawanshi et al., 2012	9	100	0.09	[0.04; 0.16]	0.0%	0.1%
Tigist et al., 2015	7	209	0.03	[0.01; 0.07]	0.0%	0.1%
Tiwari et al. 2013	19	745	0.03	[0.02; 0.04]	0.2%	0.1%
Vouking et al., 2014	19	207	0.09	[0.06; 0.14]	0.0%	0.1%
Wumba et al., 2012	3	242	0.01	[0.00; 0.04]	0.1%	0.1%
Yosefi et al., 2012	2	60	0.03	[0.00; 0.12]	0.0%	0.1%
Zeynudin et al., 2013	9	91	0.10	[0.05; 0.18]	0.0%	0.1%
Abange et al., 2020	1	119	0.01	[0.00; 0.05]	0.1%	0.1%
Namaji et al., 2020	2	361	0.01	[0.00; 0.02]	0.3%	0.1%
Purbey., 2021	3	75	0.04	[0.01; 0.11]	0.0%	0.1%
Wolday et al., 2021	17	751	0.02	[0.01; 0.04]	0.2%	0.1%
Ajayi et al., 2021	64	375	0.17	[0.13; 0.21]	0.0%	0.1%
Bayleyegn et al., 2021	25	255	0.10	[0.06; 0.14]	0.0%	0.1%
Chandi et al., 2021	5	80	0.06	[0.02; 0.14]	0.0%	0.1%
Feleke et al., 2022	12	223	0.05	[0.03; 0.09]	0.0%	0.1%
Miressa et al., 2021	23	230	0.10	[0.06; 0.15]	0.0%	0.1%
Common effect model		13654	0.01	[0.01; 0.02]	5.4%	--
Random effects model			0.07	[0.06; 0.09]	--	6.9%
Heterogeneity: $I^2 = 93\%$, $\tau^2 = 0.0024$, $p < 0.01$						
**Protozoa_Species = G. lamblia**						
Abaver et al., 2011	3	85	0.04	[0.01; 0.10]	0.0%	0.1%
Abilo and Afework, 2005	6	70	0.09	[0.03; 0.18]	0.0%	0.1%
Adamu and Petros, 2009	32	200	0.16	[0.11; 0.22]	0.0%	0.1%
Agholi et al., 2013	23	356	0.06	[0.04; 0.10]	0.0%	0.1%
Assefa et al., 2009	24	214	0.11	[0.07; 0.16]	0.0%	0.1%
Assefa et al., 2013	20	272	0.07	[0.05; 0.11]	0.0%	0.1%
Babatunde et al., 2010	16	90	0.18	[0.11; 0.27]	0.0%	0.1%
Barcelos et al., 2018	1	90	0.01	[0.00; 0.06]	0.0%	0.1%
Berenji et al., 2010	7	31	0.23	[0.10; 0.41]	0.0%	0.1%
Cardoso et al., 2011	5	500	0.01	[0.00; 0.02]	0.3%	0.1%
da Silva et al., 2005	1	100	0.01	[0.00; 0.05]	0.0%	0.1%
Daryani et al., 2009	2	64	0.03	[0.00; 0.11]	0.0%	0.1%
Dash et al., 2013	1	115	0.01	[0.00; 0.05]	0.0%	0.1%
Eshetu et al., 2017	4	223	0.02	[0.00; 0.05]	0.1%	0.1%
Gaurav and Atul, 2015	2	40	0.05	[0.01; 0.17]	0.0%	0.1%
Gebretsadik et al., 2018	3	223	0.01	[0.00; 0.04]	0.1%	0.1%
Getaneh et al., 2018	4	220	0.02	[0.00; 0.05]	0.1%	0.1%
Guk et al., 2005	1	105	0.01	[0.00; 0.05]	0.0%	0.1%
Gupta et al., 2013	8	100	0.08	[0.04; 0.15]	0.0%	0.1%
Haileeyesus et al., 2013	15	378	0.04	[0.02; 0.06]	0.1%	0.1%
Huibers et al., 2018	9	35	0.26	[0.12; 0.43]	0.0%	0.1%
Javid et al., 2005	45	200	0.22	[0.17; 0.29]	0.0%	0.1%
Kipyegen et al., 2012	25	285	0.09	[0.06; 0.13]	0.0%	0.1%
Kiros et al., 2015	17	399	0.04	[0.03; 0.07]	0.1%	0.1%
Mahmud et al., 2014	47	372	0.13	[0.09; 0.16]	0.0%	0.1%
Mariam et al., 2008	11	109	0.10	[0.05; 0.17]	0.0%	0.1%
Meamar et al., 2007	33	781	0.04	[0.03; 0.06]	0.1%	0.1%
Mehta et al., 2013	5	100	0.05	[0.02; 0.11]	0.0%	0.1%
Missaye et al., 2013	20	272	0.07	[0.05; 0.11]	0.0%	0.1%
Moges et al., 2006	2	28	0.07	[0.01; 0.24]	0.0%	0.1%
Moran et al., 2005	4	203	0.02	[0.01; 0.05]	0.1%	0.1%
Nkenfou et al., 2013	1	42	0.02	[0.00; 0.13]	0.0%	0.1%
Nkenfou et al., 2017	2	68	0.03	[0.00; 0.10]	0.0%	0.1%
Nsagha et al., 2016	10	300	0.03	[0.02; 0.06]	0.1%	0.1%
Nuhu et al., 2014	13	205	0.06	[0.03; 0.11]	0.0%	0.1%
Patel et al., 2011	2	100	0.02	[0.00; 0.07]	0.0%	0.1%
Pennap et al., 2015	12	200	0.06	[0.03; 0.10]	0.0%	0.1%
Pinlaor et al., 2005	1	78	0.01	[0.00; 0.07]	0.0%	0.1%
Ramakrishnan et al., 2007	3	80	0.04	[0.01; 0.11]	0.0%	0.1%
Ramana et al., 2010	34	452	0.08	[0.05; 0.10]	0.0%	0.1%
Raytekar et al., 2012	3	127	0.02	[0.00; 0.07]	0.0%	0.1%
Ringo et al., 2015	8	83	0.10	[0.04; 0.18]	0.0%	0.1%
Samie et al., 2014	18	151	0.12	[0.07; 0.18]	0.0%	0.1%
Sherchan et al., 2012	14	146	0.10	[0.05; 0.16]	0.0%	0.1%
Sherpa et al., 2010	3	106	0.03	[0.01; 0.08]	0.0%	0.1%
Shimelis et al., 2016	39	491	0.08	[0.06; 0.11]	0.0%	0.1%
Silva et al., 2005	1	100	0.01	[0.00; 0.05]	0.0%	0.1%
Suryawanshi et al., 2012	6	100	0.06	[0.02; 0.13]	0.0%	0.1%
Tamirat et al., 2017	8	226	0.04	[0.02; 0.07]	0.0%	0.1%
Tegegne et al., 2017	4	223	0.02	[0.00; 0.05]	0.1%	0.1%
Tigist et al., 2015	31	209	0.15	[0.10; 0.20]	0.0%	0.1%
Tiwari et al., 2013	26	745	0.03	[0.02; 0.05]	0.1%	0.1%
Vyas et al., 2013	3	75	0.04	[0.01; 0.11]	0.0%	0.1%
Wanyiri et al., 2013	13	164	0.08	[0.04; 0.13]	0.0%	0.1%
Wumba et al., 2012	2	242	0.01	[0.00; 0.03]	0.1%	0.1%
Zeynudin et al., 2013	8	91	0.09	[0.04; 0.17]	0.0%	0.1%
Gebrecherkos et al., 2019	6	150	0.04	[0.01; 0.09]	0.0%	0.1%
Masoumi et al., 2019	1	102	0.01	[0.00; 0.05]	0.0%	0.1%
Abange et al., 2020	5	119	0.04	[0.01; 0.10]	0.0%	0.1%
Alemayehu et al., 2020	8	383	0.02	[0.01; 0.04]	0.1%	0.1%
Ayele et al., 2020	16	180	0.09	[0.05; 0.14]	0.0%	0.1%
Belay et al., 2020	4	112	0.04	[0.01; 0.09]	0.0%	0.1%
Chhangte et al., 2020	7	250	0.03	[0.01; 0.06]	0.0%	0.1%
Namaji et al., 2020	1	361	0.00	[0.00; 0.02]	0.5%	0.1%
Wolday et al., 2021	27	751	0.04	[0.02; 0.05]	0.1%	0.1%
Bayleyegn et al., 2021	3	255	0.01	[0.00; 0.03]	0.1%	0.1%
Feleke et al., 2022	4	223	0.02	[0.00; 0.05]	0.1%	0.1%
Miressa et al., 2021	50	230	0.22	[0.17; 0.28]	0.0%	0.1%
Common effect model		14180	0.02	[0.02; 0.03]	2.9%	--
Random effects model			0.05	[0.04; 0.06]	--	8.4%
Heterogeneity: $I^2 = 87\%$, $\tau^2 = 0.0017$, $p < 0.01$						
**Protozoa_Species = E. coli**						
Abaver et al., 2011	3	85	0.04	[0.01; 0.10]	0.0%	0.1%
Assefa et al., 2009	4	214	0.02	[0.01; 0.05]	0.1%	0.1%
Babatunde et al., 2010	12	90	0.13	[0.07; 0.22]	0.0%	0.1%
Barcelos et al., 2018	6	90	0.07	[0.02; 0.14]	0.0%	0.1%
Berenji et al., 2010	3	31	0.10	[0.02; 0.26]	0.0%	0.1%
Cardoso et al., 2011	4	500	0.01	[0.00; 0.02]	0.3%	0.1%
Daryani et al., 2009	1	64	0.02	[0.00; 0.08]	0.0%	0.1%
Feyisayo et al., 2014	1	105	0.01	[0.00; 0.05]	0.0%	0.1%
Gupta et al., 2013	7	100	0.07	[0.03; 0.14]	0.0%	0.1%
Jayalakshmi et al., 2008	2	89	0.02	[0.00; 0.08]	0.0%	0.1%
Jegede et al., 2014	1	105	0.01	[0.00; 0.05]	0.0%	0.1%
Kipyegen et al., 2012	9	285	0.03	[0.01; 0.06]	0.1%	0.1%
Kurniawan et al., 2009	2	318	0.01	[0.00; 0.02]	0.2%	0.1%
Lar et al., 2016	15	205	0.07	[0.04; 0.12]	0.0%	0.1%
Lehman et al., 2013	3	201	0.01	[0.00; 0.04]	0.1%	0.1%
Minti et al., 2017	1	27	0.04	[0.00; 0.19]	0.0%	0.1%
Moran et al., 2005	15	203	0.07	[0.04; 0.12]	0.0%	0.1%
Mtapuri et al., 2014	6	29	0.21	[0.08; 0.40]	0.0%	0.1%
Ngum et al., 2015	43	320	0.13	[0.10; 0.18]	0.0%	0.1%
Nkenfou et al., 2013	9	42	0.21	[0.10; 0.37]	0.0%	0.1%
Nkenfou et al., 2017	10	68	0.15	[0.07; 0.25]	0.0%	0.1%
Oguntibeju 2006	6	60	0.10	[0.04; 0.21]	0.0%	0.1%
Paboriboune et al., 2014	1	137	0.01	[0.00; 0.04]	0.1%	0.1%
Ramakrishnan et al., 2007	16	80	0.20	[0.12; 0.30]	0.0%	0.1%
Ramana et al., 2010	28	452	0.06	[0.04; 0.09]	0.0%	0.1%
Rodrigues et al., 2008	90	582	0.15	[0.13; 0.19]	0.0%	0.1%
Roka et al., 2012	41	260	0.16	[0.12; 0.21]	0.0%	0.1%
Roka et al., 2013	33	273	0.12	[0.08; 0.17]	0.0%	0.1%
Sanyaolu et al., 2011	5	65	0.08	[0.03; 0.17]	0.0%	0.1%
Stark et al., 2007	18	628	0.03	[0.02; 0.04]	0.1%	0.1%
Tamirat et al., 2017	3	226	0.01	[0.00; 0.04]	0.1%	0.1%
Vouking et al., 2014	27	207	0.13	[0.09; 0.18]	0.0%	0.1%
Wumba et al., 2012	20	242	0.08	[0.05; 0.12]	0.0%	0.1%
Yosefi et al., 2012	5	60	0.08	[0.03; 0.18]	0.0%	0.1%
Abange et al., 2020	3	119	0.03	[0.01; 0.07]	0.0%	0.1%
Chhangte et al., 2020	8	250	0.03	[0.01; 0.06]	0.0%	0.1%
Lengongo et al., 2020	15	115	0.13	[0.07; 0.21]	0.0%	0.1%
Barreto et al., 2022	11	178	0.06	[0.03; 0.11]	0.0%	0.1%
Betancourth et al., 2021	2	102	0.02	[0.00; 0.07]	0.0%	0.1%
Common effect model		7207	0.03	[0.02; 0.03]	1.5%	--
Random effects model			0.06	[0.05; 0.08]	--	4.8%
Heterogeneity: $I^2 = 89\%$, $\tau^2 = 0.0023$, $p < 0.01$						
**Protozoa_Species = Toxoplasma spp.**						
Abdollahi et al., 2013	65	100	0.65	[0.55; 0.74]	0.0%	0.1%
Alavi et al., 2013	31	42	0.74	[0.58; 0.86]	0.0%	0.1%
Bokharaei et al., 2020	95	108	0.88	[0.80; 0.93]	0.0%	0.1%
Dutta et al., 2020	292	400	0.73	[0.68; 0.77]	0.0%	0.1%
Common effect model		650	0.76	[0.73; 0.79]	0.0%	--
Random effects model			0.75	[0.66; 0.85]	--	0.5%
Heterogeneity: $I^2 = 86\%$, $\tau^2 = 0.0082$, $p < 0.01$						
**Protozoa_Species = C. parvum**						
Abilo and Afework, 2005	20	70	0.29	[0.18; 0.41]	0.0%	0.1%
Adamu et al., 2014	92	520	0.18	[0.15; 0.21]	0.0%	0.1%
Adesij et al. 2007	79	100	0.79	[0.70; 0.87]	0.0%	0.1%
Akinbo et al., 2010	16	400	0.04	[0.02; 0.06]	0.1%	0.1%
Cardoso et al., 2011	1	500	0.00	[0.00; 0.01]	1.0%	0.1%
Dash et al., 2013	14	115	0.12	[0.07; 0.20]	0.0%	0.1%
Djieyep et al., 2014	127	305	0.42	[0.36; 0.47]	0.0%	0.1%
Eshetu et al., 2017	7	223	0.03	[0.01; 0.06]	0.0%	0.1%
Gaurav and Atul, 2015	6	40	0.15	[0.06; 0.30]	0.0%	0.1%
Ghafari et al., 2018	19	250	0.08	[0.05; 0.12]	0.0%	0.1%
Guk et al., 2005	7	105	0.07	[0.03; 0.13]	0.0%	0.1%
Gupta et al., 2013	4	100	0.04	[0.01; 0.10]	0.0%	0.1%
Iqbal et al., 2015	27	346	0.08	[0.05; 0.11]	0.0%	0.1%
Jaiswal et al., 2014	3	103	0.03	[0.01; 0.08]	0.0%	0.1%
Kammili et al., 2008	3	175	0.02	[0.00; 0.05]	0.1%	0.1%
Kucerova et al., 2011	19	46	0.41	[0.27; 0.57]	0.0%	0.1%
Kulkarni et al., 2009	16	137	0.12	[0.07; 0.18]	0.0%	0.1%
Kurniawan et al., 2013	1	36	0.03	[0.00; 0.15]	0.0%	0.1%
Lar et al., 2016	17	205	0.08	[0.05; 0.13]	0.0%	0.1%
Mathur et al., 2013	135	544	0.25	[0.21; 0.29]	0.0%	0.1%
Mehta et al., 2013	2	100	0.02	[0.00; 0.07]	0.0%	0.1%
Mtapuri et al., 2014	5	29	0.17	[0.06; 0.36]	0.0%	0.1%
Ngum et al., 2015	46	320	0.14	[0.11; 0.19]	0.0%	0.1%
Nkenfou et al., 2013	8	42	0.19	[0.09; 0.34]	0.0%	0.1%
Nkenfou et al., 2017	5	68	0.07	[0.02; 0.16]	0.0%	0.1%
Nsagha et al., 2016	132	300	0.44	[0.38; 0.50]	0.0%	0.1%
Nuhu et al., 2014	19	205	0.09	[0.06; 0.14]	0.0%	0.1%
Oguntibeju 2006	6	60	0.10	[0.04; 0.21]	0.0%	0.1%
Ojuromi et al., 2016	1	90	0.01	[0.00; 0.06]	0.0%	0.1%
Patel et al., 2011	20	100	0.20	[0.13; 0.29]	0.0%	0.1%
Ramakrishnan et al., 2007	23	80	0.29	[0.19; 0.40]	0.0%	0.1%
Raytekar et al., 2012	9	127	0.07	[0.03; 0.13]	0.0%	0.1%
Sarfati et al., 2006	6	154	0.04	[0.01; 0.08]	0.0%	0.1%
Sherchan et al., 2012	4	146	0.03	[0.01; 0.07]	0.0%	0.1%
Sucilathangam et al., 2012	48	103	0.47	[0.37; 0.57]	0.0%	0.1%
Suryawanshi et al., 2012	15	100	0.15	[0.09; 0.24]	0.0%	0.1%
Tegegne et al., 2017	7	223	0.03	[0.01; 0.06]	0.0%	0.1%
Tiwari et al. 2013	23	745	0.03	[0.02; 0.05]	0.1%	0.1%
Wang et al., 2013	2	683	0.00	[0.00; 0.01]	1.1%	0.1%
Yosefi et al., 2012	8	60	0.13	[0.06; 0.25]	0.0%	0.1%
Zeynudin et al., 2013	8	91	0.09	[0.04; 0.17]	0.0%	0.1%
Gebrecherkos et al., 2019	10	150	0.07	[0.03; 0.12]	0.0%	0.1%
Sannella et al., 2019	5	155	0.03	[0.01; 0.07]	0.0%	0.1%
Ayele et al., 2020	15	180	0.08	[0.05; 0.13]	0.0%	0.1%
Belay et al., 2020	9	112	0.08	[0.04; 0.15]	0.0%	0.1%
Namaji et al., 2020	89	361	0.25	[0.20; 0.29]	0.0%	0.1%
Betancourth et al., 2021	4	102	0.04	[0.01; 0.10]	0.0%	0.1%
Chandi et al., 2021	5	80	0.06	[0.02; 0.14]	0.0%	0.1%
Common effect model		9286	0.02	[0.02; 0.02]	2.8%	--
Random effects model			0.13	[0.09; 0.18]	--	5.8%
Heterogeneity: $I^2 = 97\%$, $\tau^2 = 0.0214$, $p < 0.01$						
**Protozoa_Species = I. belli**						
Abilo and Afework, 2005	1	70	0.01	[0.00; 0.08]	0.0%	0.1%
Adamu and Petros, 2009	10	200	0.05	[0.02; 0.09]	0.0%	0.1%
Agholi et al., 2013	2	356	0.01	[0.00; 0.02]	0.3%	0.1%
Ahmed and Abhay, 2015	22	142	0.15	[0.10; 0.23]	0.0%	0.1%
Akinbo et al., 2011	16	463	0.03	[0.02; 0.06]	0.1%	0.1%
Akinbo et al., 2012	21	2000	0.01	[0.01; 0.02]	1.1%	0.1%
Archana et al., 2017	17	104	0.16	[0.10; 0.25]	0.0%	0.1%
Asma et al., 2011	35	346	0.10	[0.07; 0.14]	0.0%	0.1%
Assefa et al., 2009	26	214	0.12	[0.08; 0.17]	0.0%	0.1%
Assefa et al., 2013	1	272	0.00	[0.00; 0.02]	0.3%	0.1%
Cardoso et al., 2011	6	500	0.01	[0.00; 0.03]	0.2%	0.1%
da Silva et al., 2005	7	100	0.07	[0.03; 0.14]	0.0%	0.1%
Dash et al., 2013	19	115	0.17	[0.10; 0.25]	0.0%	0.1%
Djieyep et al., 2014	95	305	0.31	[0.26; 0.37]	0.0%	0.1%
Echoru et al., 2015	2	111	0.02	[0.00; 0.06]	0.0%	0.1%
Gaurav and Atul 2015	4	40	0.10	[0.03; 0.24]	0.0%	0.1%
Gebretsadik et al., 2018	1	223	0.00	[0.00; 0.02]	0.2%	0.1%
Getaneh et al., 2018	3	220	0.01	[0.00; 0.04]	0.1%	0.1%
Girma et al., 2014	4	268	0.01	[0.00; 0.04]	0.1%	0.1%
Guk et al., 2005	5	105	0.05	[0.02; 0.11]	0.0%	0.1%
Gupta et al., 2013	10	100	0.10	[0.05; 0.18]	0.0%	0.1%
Haileeyesus et al., 2013	6	378	0.02	[0.01; 0.03]	0.1%	0.1%
Jayalakshmi et al., 2008	4	89	0.04	[0.01; 0.11]	0.0%	0.1%
Kiros et al., 2015	5	399	0.01	[0.00; 0.03]	0.2%	0.1%
Kulkarni et al., 2009	11	137	0.08	[0.04; 0.14]	0.0%	0.1%
Kurniawan et al., 2009	2	318	0.01	[0.00; 0.02]	0.2%	0.1%
Lehman et al., 2013	1	201	0.00	[0.00; 0.03]	0.2%	0.1%
Mariam et al., 2008	2	109	0.02	[0.00; 0.06]	0.0%	0.1%
Mathur et al., 2013	42	544	0.08	[0.06; 0.10]	0.0%	0.1%
Meamar et al., 2007	2	781	0.00	[0.00; 0.01]	1.4%	0.1%
Mekonnen et al., 2014	4	268	0.01	[0.00; 0.04]	0.1%	0.1%
Missaye et al., 2013	1	272	0.00	[0.00; 0.02]	0.3%	0.1%
Mohan et al., 2014	11	150	0.07	[0.04; 0.13]	0.0%	0.1%
Moran et al., 2005	1	203	0.00	[0.00; 0.03]	0.2%	0.1%
Navarro et al., 2006	2	103	0.02	[0.00; 0.07]	0.0%	0.1%
Nsagha et al., 2016	13	300	0.04	[0.02; 0.07]	0.1%	0.1%
Oguntibeju 2006	2	60	0.03	[0.00; 0.12]	0.0%	0.1%
Patel et al., 2011	3	100	0.03	[0.01; 0.09]	0.0%	0.1%
Pavie et al., 2012	1	143	0.01	[0.00; 0.04]	0.1%	0.1%
Pennap et al., 2015	7	200	0.04	[0.01; 0.07]	0.0%	0.1%
Pinlaor et al., 2005	1	78	0.01	[0.00; 0.07]	0.0%	0.1%
Ramakrishnan et al., 2007	1	80	0.01	[0.00; 0.07]	0.0%	0.1%
Rodrigues et al., 2008	24	582	0.04	[0.03; 0.06]	0.1%	0.1%
Saksirisampant et al., 2009	1	90	0.01	[0.00; 0.06]	0.0%	0.1%
Sanyaolu et al., 2011	2	65	0.03	[0.00; 0.11]	0.0%	0.1%
Sarfati et al., 2006	3	154	0.02	[0.00; 0.06]	0.0%	0.1%
Sherchan et al., 2012	3	146	0.02	[0.00; 0.06]	0.0%	0.1%
Shimelis et al., 2016	11	491	0.02	[0.01; 0.04]	0.1%	0.1%
Silva et al., 2005	7	100	0.07	[0.03; 0.14]	0.0%	0.1%
Sintayehu et al., 2013	2	343	0.01	[0.00; 0.02]	0.3%	0.1%
Sonali et al., 2014	2	250	0.01	[0.00; 0.03]	0.1%	0.1%
Suryawanshi et al., 2012	13	100	0.13	[0.07; 0.21]	0.0%	0.1%
Tuli et al., 2008	2	366	0.01	[0.00; 0.02]	0.3%	0.1%
Vouking et al., 2014	12	207	0.06	[0.03; 0.10]	0.0%	0.1%
Vyas et al., 2013	9	75	0.12	[0.06; 0.22]	0.0%	0.1%
Wanyiri et al., 2013	18	164	0.11	[0.07; 0.17]	0.0%	0.1%
Wumba et al., 2010	3	175	0.02	[0.00; 0.05]	0.1%	0.1%
Zeynudin et al., 2013	6	91	0.07	[0.02; 0.14]	0.0%	0.1%
Gebrecherkos et al., 2019	3	150	0.02	[0.00; 0.06]	0.0%	0.1%
Lengongo et al., 2020	1	115	0.01	[0.00; 0.05]	0.0%	0.1%
Feleke et al., 2022	2	223	0.01	[0.00; 0.03]	0.1%	0.1%
Common effect model		15054	0.01	[0.01; 0.01]	6.8%	--
Random effects model			0.04	[0.03; 0.05]	--	7.6%
Heterogeneity: $I^2 = 87\%$, $\tau^2 = 0.0021$, $p < 0.01$						
**Protozoa_Species = Cryptosporidium spp.**						
Adamu and Petros, 2009	16	200	0.08	[0.05; 0.13]	0.0%	0.1%
Agholi et al., 2013	34	356	0.10	[0.07; 0.13]	0.0%	0.1%
Ahmed and Abhay, 2015	6	142	0.04	[0.02; 0.09]	0.0%	0.1%
Ajjampur et al., 2007	48	534	0.09	[0.07; 0.12]	0.0%	0.1%
Akinbo et al., 2012	74	2000	0.04	[0.03; 0.05]	0.3%	0.1%
Alberto et al., 2011	38	131	0.29	[0.21; 0.38]	0.0%	0.1%
Amoo et al., 2018	52	231	0.23	[0.17; 0.28]	0.0%	0.1%
Asma et al., 2011	43	346	0.12	[0.09; 0.16]	0.0%	0.1%
Assefa et al., 2009	43	214	0.20	[0.15; 0.26]	0.0%	0.1%
Assefa et al., 2013	2	272	0.01	[0.00; 0.03]	0.2%	0.1%
Babatunde et al., 2010	29	90	0.32	[0.23; 0.43]	0.0%	0.1%
Barcelos et al., 2018	1	90	0.01	[0.00; 0.06]	0.0%	0.1%
da Silva et al., 2005	4	100	0.04	[0.01; 0.10]	0.0%	0.1%
Daryani et al., 2009	6	64	0.09	[0.04; 0.19]	0.0%	0.1%
Echoru et al., 2015	4	111	0.04	[0.01; 0.09]	0.0%	0.1%
Erhabor et al., 2011	3	105	0.03	[0.01; 0.08]	0.0%	0.1%
Gabriela et al., 2005	59	397	0.15	[0.12; 0.19]	0.0%	0.1%
Gebretsadik et al., 2018	3	223	0.01	[0.00; 0.04]	0.1%	0.1%
Getaneh et al., 2018	19	220	0.09	[0.05; 0.13]	0.0%	0.1%
Gholami et al., 2016	4	53	0.08	[0.02; 0.18]	0.0%	0.1%
Girma et al., 2014	92	268	0.34	[0.29; 0.40]	0.0%	0.1%
Haileeyesus et al., 2013	32	378	0.08	[0.06; 0.12]	0.0%	0.1%
Huibers et al., 2018	4	35	0.11	[0.03; 0.27]	0.0%	0.1%
Hung et al., 2007	1	1376	0.00	[0.00; 0.00]	7.2%	0.1%
Hussain et al., 2016	25	127	0.20	[0.13; 0.28]	0.0%	0.1%
Iqbal et al., 2015	11	346	0.03	[0.02; 0.06]	0.1%	0.1%
Jayalakshmi et al., 2008	12	89	0.13	[0.07; 0.22]	0.0%	0.1%
Javid et al., 2005	84	200	0.42	[0.35; 0.49]	0.0%	0.1%
Kiros et al., 2015	23	399	0.06	[0.04; 0.09]	0.0%	0.1%
Kurniawan et al., 2009	32	318	0.10	[0.07; 0.14]	0.0%	0.1%
Langoni and Souza, 2007	11	105	0.10	[0.05; 0.18]	0.0%	0.1%
Lehman et al., 2013	13	201	0.06	[0.03; 0.11]	0.0%	0.1%
Mariam et al., 2008	8	109	0.07	[0.03; 0.14]	0.0%	0.1%
Masarat et al., 2012	45	45	1.00	[0.92; 1.00]	0.0%	0.1%
Meamar et al., 2007	7	781	0.01	[0.00; 0.02]	0.5%	0.1%
Mekonnen et al., 2014	92	268	0.34	[0.29; 0.40]	0.0%	0.1%
Missaye et al., 2013	2	272	0.01	[0.00; 0.03]	0.2%	0.1%
Mitra et al., 2016	57	194	0.29	[0.23; 0.36]	0.0%	0.1%
Mohan et al., 2014	2	150	0.01	[0.00; 0.05]	0.1%	0.1%
Ojurongbe et al., 2011	52	96	0.54	[0.44; 0.64]	0.0%	0.1%
Olopade et al., 2017	10	226	0.04	[0.02; 0.08]	0.0%	0.1%
Oyedeji et al., 2015	10	52	0.19	[0.10; 0.33]	0.0%	0.1%
Paboriboune et al., 2014	9	137	0.07	[0.03; 0.12]	0.0%	0.1%
Pavie et al., 2012	9	143	0.06	[0.03; 0.12]	0.0%	0.1%
Pennap et al., 2015	9	200	0.04	[0.02; 0.08]	0.0%	0.1%
Pinlaor et al., 2005	9	78	0.12	[0.05; 0.21]	0.0%	0.1%
Ramana et al., 2010	48	452	0.11	[0.08; 0.14]	0.0%	0.1%
Rashmi and Ravi, 2013	15	90	0.17	[0.10; 0.26]	0.0%	0.1%
Rodrigues et al., 2008	39	582	0.07	[0.05; 0.09]	0.1%	0.1%
Roka et al., 2012	24	260	0.09	[0.06; 0.13]	0.0%	0.1%
Roka et al., 2013	8	273	0.03	[0.01; 0.06]	0.1%	0.1%
Samie et al., 2014	40	151	0.26	[0.20; 0.34]	0.0%	0.1%
Sanyaolu et al., 2011	5	65	0.08	[0.03; 0.17]	0.0%	0.1%
Sherpa et al., 2010	8	106	0.08	[0.03; 0.14]	0.0%	0.1%
Shimelis and Tadesse, 2014	32	250	0.13	[0.09; 0.18]	0.0%	0.1%
Shimelis et al., 2016	65	491	0.13	[0.10; 0.17]	0.0%	0.1%
Silva et al., 2005	4	100	0.04	[0.01; 0.10]	0.0%	0.1%
Sintayehu et al., 2013	10	343	0.03	[0.01; 0.05]	0.1%	0.1%
Sonali et al., 2014	52	250	0.21	[0.16; 0.26]	0.0%	0.1%
Srisuphanunt et al., 2008	23	143	0.16	[0.10; 0.23]	0.0%	0.1%
Stark et al., 2007	14	628	0.02	[0.01; 0.04]	0.2%	0.1%
Stensvold et al., 2011	1	96	0.01	[0.00; 0.06]	0.0%	0.1%
Taherkhani et al., 2007	20	75	0.27	[0.17; 0.38]	0.0%	0.1%
Tellevik et al., 2015	8	33	0.24	[0.11; 0.42]	0.0%	0.1%
Tian et al., 2012	25	302	0.08	[0.05; 0.12]	0.0%	0.1%
Tian et al., 2013	8	79	0.10	[0.04; 0.19]	0.0%	0.1%
Tuli et al., 2008	146	366	0.40	[0.35; 0.45]	0.0%	0.1%
Tumwine et al., 2005	67	91	0.74	[0.63; 0.82]	0.0%	0.1%
Uysal et al., 2017	3	115	0.03	[0.01; 0.07]	0.0%	0.1%
Vouking et al., 2014	15	207	0.07	[0.04; 0.12]	0.0%	0.1%
Vyas et al., 2013	11	75	0.15	[0.08; 0.25]	0.0%	0.1%
Wanyiri et al., 2013	17	164	0.10	[0.06; 0.16]	0.0%	0.1%
Wanyiri et al., 2014	56	164	0.34	[0.27; 0.42]	0.0%	0.1%
Wumba et al., 2010	17	175	0.10	[0.06; 0.15]	0.0%	0.1%
Yu et al. 2011	14	340	0.04	[0.02; 0.07]	0.0%	0.1%
Sannella et al., 2019	14	155	0.09	[0.05; 0.15]	0.0%	0.1%
Abange et al., 2020	7	119	0.06	[0.02; 0.12]	0.0%	0.1%
Chhangte et al., 2020	1	250	0.00	[0.00; 0.02]	0.2%	0.1%
Lengongo et al., 2020	1	115	0.01	[0.00; 0.05]	0.0%	0.1%
Purbey., 2021	2	75	0.03	[0.00; 0.09]	0.0%	0.1%
Sepahvand et al., 2022	7	26	0.27	[0.12; 0.48]	0.0%	0.1%
Uzairue et al., 2021	25	170	0.15	[0.10; 0.21]	0.0%	0.1%
Yulfi et al., 2021	13	54	0.24	[0.13; 0.38]	0.0%	0.1%
Weinreich et al., 2021	86	967	0.09	[0.07; 0.11]	0.1%	0.1%
Ajayi et al., 2021	19	375	0.05	[0.03; 0.08]	0.0%	0.1%
Bayleyegn et al., 2021	2	255	0.01	[0.00; 0.03]	0.1%	0.1%
Botero et al., 2021	1	192	0.01	[0.00; 0.03]	0.1%	0.1%
Dankwa et al., 2021	26	418	0.06	[0.04; 0.09]	0.0%	0.1%
Feleke et al., 2022	4	223	0.02	[0.00; 0.05]	0.1%	0.1%
Wumba et al., 2012	13	121	0.11	[0.06; 0.18]	0.0%	0.1%
Common effect model		22453	0.02	[0.01; 0.02]	10.5%	--
Random effects model			0.13	[0.10; 0.16]	--	11.0%
Heterogeneity: $I^2 = 99\%$, $\tau^2 = 0.0235$, $p = 0$						
**Protozoa_Species = E. histolytica/dispar**						
Adamu and Petros, 2009	26	200	0.13	[0.09; 0.18]	0.0%	0.1%
Agholi et al., 2013	7	356	0.02	[0.01; 0.04]	0.1%	0.1%
Ahmed and Abhay, 2015	7	142	0.05	[0.02; 0.10]	0.0%	0.1%
Asma et al., 2011	58	346	0.17	[0.13; 0.21]	0.0%	0.1%
Assefa et al., 2009	53	214	0.25	[0.19; 0.31]	0.0%	0.1%
Barcelos et al., 2018	1	90	0.01	[0.00; 0.06]	0.0%	0.1%
Dash et al., 2013	9	115	0.08	[0.04; 0.14]	0.0%	0.1%
Eshetu et al., 2017	19	223	0.09	[0.05; 0.13]	0.0%	0.1%
Getaneh et al., 2018	8	220	0.04	[0.02; 0.07]	0.0%	0.1%
Haileeyesus et al., 2013	16	378	0.04	[0.02; 0.07]	0.1%	0.1%
Kipyegen et al., 2012	88	285	0.31	[0.26; 0.37]	0.0%	0.1%
Kiros et al., 2015	77	399	0.19	[0.16; 0.24]	0.0%	0.1%
Kulkarni et al., 2009	9	137	0.07	[0.03; 0.12]	0.0%	0.1%
Kurniawan et al., 2009	1	318	0.00	[0.00; 0.02]	0.4%	0.1%
Lehman et al., 2013	3	201	0.01	[0.00; 0.04]	0.1%	0.1%
Mahmud et al., 2014	147	372	0.40	[0.35; 0.45]	0.0%	0.1%
Missaye et al., 2013	43	272	0.16	[0.12; 0.21]	0.0%	0.1%
Moran et al., 2009	14	100	0.14	[0.08; 0.22]	0.0%	0.1%
Navarro et al., 2006	10	103	0.10	[0.05; 0.17]	0.0%	0.1%
Paboriboune et al., 2014	17	137	0.12	[0.07; 0.19]	0.0%	0.1%
Pavie et al., 2012	4	143	0.03	[0.01; 0.07]	0.0%	0.1%
Rodrigues et al., 2008	17	582	0.03	[0.02; 0.05]	0.1%	0.1%
Roka et al., 2012	112	260	0.43	[0.37; 0.49]	0.0%	0.1%
Sanyaolu et al., 2011	6	65	0.09	[0.03; 0.19]	0.0%	0.1%
Sarfati et al., 2006	7	154	0.05	[0.02; 0.09]	0.0%	0.1%
Stark et al., 2007	20	628	0.03	[0.02; 0.05]	0.1%	0.1%
Tamirat et al., 2017	20	226	0.09	[0.05; 0.13]	0.0%	0.1%
Tegegne et al., 2017	19	223	0.09	[0.05; 0.13]	0.0%	0.1%
Vyas et al., 2013	4	75	0.05	[0.01; 0.13]	0.0%	0.1%
Wanyiri et al., 2013	15	164	0.09	[0.05; 0.15]	0.0%	0.1%
Wumba et al., 2010	9	175	0.05	[0.02; 0.10]	0.0%	0.1%
Gebrecherkos et al., 2019	31	150	0.21	[0.14; 0.28]	0.0%	0.1%
Alemayehu et al., 2020	7	383	0.02	[0.01; 0.04]	0.1%	0.1%
Belay et al., 2020	8	112	0.07	[0.03; 0.14]	0.0%	0.1%
Chhangte et al., 2020	24	250	0.10	[0.06; 0.14]	0.0%	0.1%
Lengongo et al., 2020	1	115	0.01	[0.00; 0.05]	0.0%	0.1%
Barreto et al., 2022	1	178	0.01	[0.00; 0.03]	0.1%	0.1%
Betancourth et al., 2021	2	102	0.02	[0.00; 0.07]	0.0%	0.1%
Botero et al., 2021	25	192	0.13	[0.09; 0.19]	0.0%	0.1%
Common effect model		8785	0.03	[0.03; 0.03]	1.5%	--
Random effects model			0.10	[0.07; 0.13]	--	4.8%
Heterogeneity: $I^2 = 96\%$, $\tau^2 = 0.0095$, $p < 0.01$						
**Protozoa_Species = B. hominis**						
Adamu and Petros, 2009	3	200	0.01	[0.00; 0.04]	0.1%	0.1%
Agholi et al., 2013	14	356	0.04	[0.02; 0.07]	0.1%	0.1%
Barcelos et al., 2018	2	90	0.02	[0.00; 0.08]	0.0%	0.1%
Berenji et al., 2010	7	31	0.23	[0.10; 0.41]	0.0%	0.1%
Feyisayo et al., 2014	1	105	0.01	[0.00; 0.05]	0.0%	0.1%
Gupta et al., 2013	1	100	0.01	[0.00; 0.05]	0.0%	0.1%
Jegede et al., 2014	1	105	0.01	[0.00; 0.05]	0.0%	0.1%
Kurniawan et al., 2009	234	318	0.74	[0.68; 0.78]	0.0%	0.1%
Meamar et al., 2007	48	781	0.06	[0.05; 0.08]	0.1%	0.1%
Mohan et al., 2014	1	150	0.01	[0.00; 0.04]	0.1%	0.1%
Moran et al., 2005	4	203	0.02	[0.01; 0.05]	0.1%	0.1%
Mtapuri et al., 2014	1	29	0.03	[0.00; 0.18]	0.0%	0.1%
Nkenfou et al., 2017	30	68	0.44	[0.32; 0.57]	0.0%	0.1%
Nsagha et al., 2016	75	300	0.25	[0.20; 0.30]	0.0%	0.1%
Oguntibeju 2006	2	60	0.03	[0.00; 0.12]	0.0%	0.1%
Pennap et al., 2015	11	200	0.06	[0.03; 0.10]	0.0%	0.1%
Pinlaor et al., 2005	1	78	0.01	[0.00; 0.07]	0.0%	0.1%
Ramana et al., 2010	139	452	0.31	[0.27; 0.35]	0.0%	0.1%
Roka et al., 2012	28	260	0.11	[0.07; 0.15]	0.0%	0.1%
Roka et al., 2013	16	273	0.06	[0.03; 0.09]	0.0%	0.1%
Samie et al., 2014	3	151	0.02	[0.00; 0.06]	0.0%	0.1%
Sherchan et al., 2012	9	146	0.06	[0.03; 0.11]	0.0%	0.1%
Stark et al., 2007	111	628	0.18	[0.15; 0.21]	0.0%	0.1%
Tamirat et al., 2017	14	226	0.06	[0.03; 0.10]	0.0%	0.1%
Tian et al., 2012	49	302	0.16	[0.12; 0.21]	0.0%	0.1%
Tian et al., 2013	11	79	0.14	[0.07; 0.24]	0.0%	0.1%
Tiwari et al. 2013	2	745	0.00	[0.00; 0.01]	1.3%	0.1%
Yosefi et al., 2012	2	60	0.03	[0.00; 0.12]	0.0%	0.1%
Masoumi et al., 2019	2	102	0.02	[0.00; 0.07]	0.0%	0.1%
Abange et al., 2020	3	119	0.03	[0.01; 0.07]	0.0%	0.1%
Chhangte et al., 2020	17	250	0.07	[0.04; 0.11]	0.0%	0.1%
Yulfi et al., 2021	5	54	0.09	[0.03; 0.20]	0.0%	0.1%
Betancourth et al., 2021	11	102	0.11	[0.06; 0.18]	0.0%	0.1%
Common effect model		7123	0.02	[0.02; 0.02]	2.1%	--
Random effects model			0.10	[0.05; 0.15]	--	4.1%
Heterogeneity: $I^2 = 98\%$, $\tau^2 = 0.0213$, $p < 0.01$						
**Protozoa_Species = C. hominis**						
Adamu et al., 2014	25	520	0.05	[0.03; 0.07]	0.1%	0.1%
Akinbo et al., 2010	17	400	0.04	[0.02; 0.07]	0.1%	0.1%
Ghafari et al., 2018	7	250	0.03	[0.01; 0.06]	0.0%	0.1%
Hung et al., 2007	2	1376	0.00	[0.00; 0.01]	4.3%	0.1%
Iqbal et al., 2015	2	346	0.01	[0.00; 0.02]	0.3%	0.1%
Kammili et al., 2008	9	175	0.05	[0.02; 0.10]	0.0%	0.1%
Kurniawan et al., 2013	25	36	0.69	[0.52; 0.84]	0.0%	0.1%
Maikai et al., 2012	1	61	0.02	[0.00; 0.09]	0.0%	0.1%
Ojuromi et al., 2016	3	90	0.03	[0.01; 0.09]	0.0%	0.1%
Saksirisampant et al., 2009	27	90	0.30	[0.21; 0.41]	0.0%	0.1%
Wang et al., 2013	3	683	0.00	[0.00; 0.01]	0.8%	0.1%
Sannella et al., 2019	41	155	0.26	[0.20; 0.34]	0.0%	0.1%
Common effect model		4182	0.00	[0.00; 0.01]	5.6%	--
Random effects model			0.12	[0.01; 0.22]	--	1.5%
Heterogeneity: $I^2 = 95\%$, $\tau^2 = 0.0345$, $p < 0.01$						
**Protozoa_Species = C. viatorum**						
Adamu et al., 2014	10	520	0.02	[0.01; 0.04]	0.1%	0.1%
Common effect model		520	0.02	[0.01; 0.03]	0.1%	--
Random effects model			0.02	[0.01; 0.03]	--	0.1%
Heterogeneity: not applicable						
**Protozoa_Species = C. felis**						
Adamu et al., 2014	5	520	0.01	[0.00; 0.02]	0.3%	0.1%
Akinbo et al., 2010	2	400	0.00	[0.00; 0.02]	0.4%	0.1%
Hung et al., 2007	1	1376	0.00	[0.00; 0.00]	7.2%	0.1%
Iqbal et al., 2015	1	346	0.00	[0.00; 0.02]	0.5%	0.1%
Kurniawan et al., 2013	3	36	0.08	[0.02; 0.22]	0.0%	0.1%
Sannella et al., 2019	7	155	0.05	[0.02; 0.09]	0.0%	0.1%
Common effect model		2833	0.00	[0.00; 0.00]	8.3%	--
Random effects model			0.00	[0.00; 0.01]	--	0.7%
Heterogeneity: $I^2 = 69\%$, $\tau^2 = < 0.0001$, $p < 0.01$						
**Protozoa_Species = C. meleagridis**						
Adamu et al., 2014	3	520	0.01	[0.00; 0.02]	0.4%	0.1%
Ghafari et al., 2018	1	250	0.00	[0.00; 0.02]	0.2%	0.1%
Hung et al., 2007	1	1376	0.00	[0.00; 0.00]	7.2%	0.1%
Iqbal et al., 2015	2	346	0.01	[0.00; 0.02]	0.3%	0.1%
Kurniawan et al., 2013	4	36	0.11	[0.03; 0.26]	0.0%	0.1%
Ojuromi et al., 2016	1	90	0.01	[0.00; 0.06]	0.0%	0.1%
Saksirisampant et al., 2009	4	90	0.04	[0.01; 0.11]	0.0%	0.1%
Wang et al., 2013	5	683	0.01	[0.00; 0.02]	0.5%	0.1%
Sannella et al., 2019	19	155	0.12	[0.08; 0.18]	0.0%	0.1%
Common effect model		3546	0.00	[0.00; 0.00]	8.7%	--
Random effects model			0.02	[0.00; 0.04]	--	1.1%
Heterogeneity: $I^2 = 79\%$, $\tau^2 = 0.0008$, $p < 0.01$						
**Protozoa_Species = C. canis**						
Adamu et al., 2014	2	520	0.00	[0.00; 0.01]	0.6%	0.1%
Akinbo et al., 2010	1	400	0.00	[0.00; 0.01]	0.6%	0.1%
Sannella et al., 2019	12	155	0.08	[0.04; 0.13]	0.0%	0.1%
Common effect model		1075	0.00	[0.00; 0.01]	1.2%	--
Random effects model			0.02	[0.00; 0.07]	--	0.4%
Heterogeneity: $I^2 = 83\%$, $\tau^2 = 0.0013$, $p < 0.01$						
**Protozoa_Species = C. xiaoi**						
Adamu et al., 2014	2	520	0.00	[0.00; 0.01]	0.6%	0.1%
Common effect model		520	0.00	[0.00; 0.01]	0.6%	--
Random effects model			0.00	[0.00; 0.01]	--	0.1%
Heterogeneity: not applicable						
**Protozoa_Species = T. gondii**						
Addebbous et al., 2012	59	95	0.62	[0.52; 0.72]	0.0%	0.1%
Aghaee et al., 2017	11	49	0.22	[0.12; 0.37]	0.0%	0.1%
Amuta et al., 2012	39	360	0.11	[0.08; 0.15]	0.0%	0.1%
Angal et al., 2016	84	133	0.63	[0.54; 0.71]	0.0%	0.1%
Anteneh et al., 2014	178	190	0.94	[0.89; 0.97]	0.0%	0.1%
Arefkhah et al., 2018	42	251	0.17	[0.12; 0.22]	0.0%	0.1%
Assob et al., 2011	93	133	0.70	[0.61; 0.78]	0.0%	0.1%
Ayi et al., 2016	72	125	0.58	[0.48; 0.66]	0.0%	0.1%
Bamba et al., 2014	11	16	0.69	[0.41; 0.89]	0.0%	0.1%
Beena et al., 2015	141	661	0.21	[0.18; 0.25]	0.0%	0.1%
Bessong and Mathomu 2010	29	160	0.18	[0.12; 0.25]	0.0%	0.1%
Chemoh et al., 2015	109	300	0.36	[0.31; 0.42]	0.0%	0.1%
Daryani et al., 2011	54	62	0.87	[0.76; 0.94]	0.0%	0.1%
Davarpanah et al., 2007	38	208	0.18	[0.13; 0.24]	0.0%	0.1%
Dechassa et al., 2016	109	135	0.81	[0.73; 0.87]	0.0%	0.1%
Domingos et al., 2013	92	200	0.46	[0.39; 0.53]	0.0%	0.1%
Ebrahim et al., 2018	51	246	0.21	[0.16; 0.26]	0.0%	0.1%
El Nahas et al., 2014	105	120	0.88	[0.80; 0.93]	0.0%	0.1%
Fisseha et al., 2013	90	103	0.87	[0.79; 0.93]	0.0%	0.1%
Ganiem et al., 2013	21	64	0.33	[0.22; 0.46]	0.0%	0.1%
Hari et al., 2007	25	307	0.08	[0.05; 0.12]	0.0%	0.1%
Hung et al., 2005	56	550	0.10	[0.08; 0.13]	0.0%	0.1%
Kodym et al., 2015	502	1235	0.41	[0.38; 0.43]	0.0%	0.1%
Lago et al., 2009	121	168	0.72	[0.65; 0.79]	0.0%	0.1%
Li et al., 2010	58	183	0.32	[0.25; 0.39]	0.0%	0.1%
Machala et al., 2009	268	626	0.43	[0.39; 0.47]	0.0%	0.1%
Malla et al., 2005	14	100	0.14	[0.08; 0.22]	0.0%	0.1%
Mengistu et al., 2014	38	43	0.88	[0.75; 0.96]	0.0%	0.1%
Mohraz et al., 2011	100	201	0.50	[0.43; 0.57]	0.0%	0.1%
Nazari et al., 2018	167	385	0.43	[0.38; 0.48]	0.0%	0.1%
Ngobeni and Samie, 2017	38	100	0.38	[0.28; 0.48]	0.0%	0.1%
Nissapatorn et al., 2007	329	693	0.47	[0.44; 0.51]	0.0%	0.1%
Ogoina et al., 2013	43	111	0.39	[0.30; 0.48]	0.0%	0.1%
Oshinaike et al., 2010	71	83	0.86	[0.76; 0.92]	0.0%	0.1%
Osunkalu et al., 2011	206	380	0.54	[0.49; 0.59]	0.0%	0.1%
Ouermi et al., 2009	44	138	0.32	[0.24; 0.40]	0.0%	0.1%
Pappoe et al., 2017	293	394	0.74	[0.70; 0.79]	0.0%	0.1%
Rahimi et al., 2015	79	82	0.96	[0.90; 0.99]	0.0%	0.1%
Reema and Dharmendra, 2017	52	100	0.52	[0.42; 0.62]	0.0%	0.1%
Rezanezhad et al., 2017	19	90	0.21	[0.13; 0.31]	0.0%	0.1%
Rostami et al., 2014	19	94	0.20	[0.13; 0.30]	0.0%	0.1%
Shafieenia et al., 2018	142	379	0.37	[0.33; 0.43]	0.0%	0.1%
Shafiei et al., 2011	49	121	0.40	[0.32; 0.50]	0.0%	0.1%
Shen et al., 2016	25	259	0.10	[0.06; 0.14]	0.0%	0.1%
Simpore et al., 2006	59	207	0.29	[0.22; 0.35]	0.0%	0.1%
Simpore et al., 2006	12	33	0.36	[0.20; 0.55]	0.0%	0.1%
Simpore et al., 2006	4	18	0.22	[0.06; 0.48]	0.0%	0.1%
Sitoe et al., 2010	18	58	0.31	[0.20; 0.45]	0.0%	0.1%
Tsegaye et al., 2014	150	170	0.88	[0.82; 0.93]	0.0%	0.1%
Xavier et al., 2013	200	250	0.80	[0.74; 0.85]	0.0%	0.1%
Zeleke and Melsew, 2017	255	270	0.94	[0.91; 0.97]	0.0%	0.1%
Bavand et al., 2019	66	149	0.44	[0.36; 0.53]	0.0%	0.1%
Hoshina et al., 2020	33	399	0.08	[0.06; 0.11]	0.0%	0.1%
Moro et al., 2020	81	332	0.24	[0.20; 0.29]	0.0%	0.1%
Zakari et al., 2020	54	160	0.34	[0.26; 0.42]	0.0%	0.1%
Steinberg et al., 2021	173	215	0.80	[0.75; 0.86]	0.0%	0.1%
Fang et al., 2021	164	325	0.50	[0.45; 0.56]	0.0%	0.1%
Sajedi et al., 2021	73	121	0.60	[0.51; 0.69]	0.0%	0.1%
Common effect model		13140	0.43	[0.42; 0.43]	0.5%	--
Random effects model			0.47	[0.40; 0.54]	--	7.0%
Heterogeneity: $I^2 = 99\%$, $\tau^2 = 0.0703$, $p = 0$						
**Protozoa_Species = Plasmodium spp.**						
Adu-Gyasi et al., 2013	15	933	0.02	[0.01; 0.03]	0.3%	0.1%
Amuta et al., 2012	343	1080	0.32	[0.29; 0.35]	0.0%	0.1%
Cohen et al., 2005	18	110	0.16	[0.10; 0.25]	0.0%	0.1%
Dabo et al., 2015	9	13	0.69	[0.39; 0.91]	0.0%	0.1%
Epelboin et al., 2012	104	208	0.50	[0.43; 0.57]	0.0%	0.1%
Franke et al., 2010	199	1066	0.19	[0.16; 0.21]	0.0%	0.1%
Frosch et al., 2017	11	138	0.08	[0.04; 0.14]	0.0%	0.1%
Gallagher et al., 2005	34	83	0.41	[0.30; 0.52]	0.0%	0.1%
Goselle et al., 2007	64	200	0.32	[0.26; 0.39]	0.0%	0.1%
Idindili et al., 2011	105	421	0.25	[0.21; 0.29]	0.0%	0.1%
Igbeneghu et al., 2012	18	38	0.47	[0.31; 0.64]	0.0%	0.1%
Iroezindu et al., 2012	24	97	0.25	[0.17; 0.35]	0.0%	0.1%
Iroezindu et al., 2012	21	63	0.33	[0.22; 0.46]	0.0%	0.1%
Izuka et al., 2017	70	102	0.69	[0.59; 0.77]	0.0%	0.1%
Johnbull et al., 2014	149	301	0.50	[0.44; 0.55]	0.0%	0.1%
Kapito et al., 2011	43	1142	0.04	[0.03; 0.05]	0.2%	0.1%
Kapito et al., 2011	4	1142	0.00	[0.00; 0.01]	1.7%	0.1%
Kawai et al., 2009	177	971	0.18	[0.16; 0.21]	0.0%	0.1%
Kolawole et al., 2017	5	81	0.06	[0.02; 0.14]	0.0%	0.1%
Manyanga et al., 2014	19	420	0.05	[0.03; 0.07]	0.1%	0.1%
Marete et al., 2014	201	245	0.82	[0.77; 0.87]	0.0%	0.1%
Njunda et al., 2016	26	355	0.07	[0.05; 0.11]	0.0%	0.1%
Okogun et al., 2010	5	16	0.31	[0.11; 0.59]	0.0%	0.1%
Quigley et al., 2005	154	222	0.69	[0.63; 0.75]	0.0%	0.1%
Rao et al., 2016	22	745	0.03	[0.02; 0.04]	0.1%	0.1%
Saracino et al., 2012	29	220	0.13	[0.09; 0.18]	0.0%	0.1%
Scotto and Fazio, 2018	15	41	0.37	[0.22; 0.53]	0.0%	0.1%
Tay et al., 2015	47	400	0.12	[0.09; 0.15]	0.0%	0.1%
Tegenaw et al., 2013	2	23	0.09	[0.01; 0.28]	0.0%	0.1%
Thompson et al., 2012	149	2408	0.06	[0.05; 0.07]	0.2%	0.1%
Uneke et al., 2005	103	490	0.21	[0.17; 0.25]	0.0%	0.1%
Wumba et al., 2015	19	25	0.76	[0.55; 0.91]	0.0%	0.1%
Zheng et al., 2017	14	101	0.14	[0.08; 0.22]	0.0%	0.1%
Chukwuocha et al., 2019	398	398	1.00	[0.99; 1.00]	1.8%	0.1%
Amodu et al., 2020	100	140	0.71	[0.63; 0.79]	0.0%	0.1%
Anyanwu et al., 2020	142	420	0.34	[0.29; 0.39]	0.0%	0.1%
Jegede et al., 2020	383	1521	0.25	[0.23; 0.27]	0.0%	0.1%
Jegede et al., 2020	3	1521	0.00	[0.00; 0.01]	3.8%	0.1%
Rabiu et al., 2020	49	197	0.25	[0.19; 0.32]	0.0%	0.1%
Jemikalajah et al., 2021	228	300	0.76	[0.71; 0.81]	0.0%	0.1%
Common effect model		18397	0.23	[0.23; 0.23]	8.5%	--
Random effects model			0.31	[0.23; 0.40]	--	4.8%
Heterogeneity: $I^2 = 100\%$, $\tau^2 = 0.0704$, $p = 0$						
**Protozoa_Species = C. cayetanensis**						
Agholi et al., 2013	1	356	0.00	[0.00; 0.02]	0.5%	0.1%
Ahmed and Abhay, 2015	3	142	0.02	[0.00; 0.06]	0.0%	0.1%
Dash et al., 2013	1	115	0.01	[0.00; 0.05]	0.1%	0.1%
Kurniawan et al., 2009	21	318	0.07	[0.04; 0.10]	0.0%	0.1%
Lehman et al., 2013	1	201	0.00	[0.00; 0.03]	0.2%	0.1%
Mtapuri et al., 2014	1	29	0.03	[0.00; 0.18]	0.0%	0.1%
Nkenfou et al., 2017	2	68	0.03	[0.00; 0.10]	0.0%	0.1%
Nsagha et al., 2016	11	300	0.04	[0.02; 0.06]	0.0%	0.1%
Paboriboune et al., 2014	3	137	0.02	[0.00; 0.06]	0.0%	0.1%
Pinlaor et al., 2005	1	78	0.01	[0.00; 0.07]	0.0%	0.1%
Saksirisampant et al., 2009	1	90	0.01	[0.00; 0.06]	0.0%	0.1%
Sherchan et al., 2012	6	146	0.04	[0.02; 0.09]	0.0%	0.1%
Sherpa et al., 2010	4	106	0.04	[0.01; 0.09]	0.0%	0.1%
Sucilathangam et al., 2012	6	103	0.06	[0.02; 0.12]	0.0%	0.1%
Tiwari et al. 2013	14	745	0.02	[0.01; 0.03]	0.2%	0.1%
Uysal et al., 2017	3	115	0.03	[0.01; 0.07]	0.0%	0.1%
Wanyiri et al., 2013	5	164	0.03	[0.01; 0.07]	0.0%	0.1%
Masoumi et al., 2019	1	102	0.01	[0.00; 0.05]	0.0%	0.1%
Belay et al., 2020	1	112	0.01	[0.00; 0.05]	0.0%	0.1%
Common effect model		3427	0.01	[0.01; 0.01]	1.3%	--
Random effects model			0.02	[0.01; 0.03]	--	2.4%
Heterogeneity: $I^2 = 63\%$, $\tau^2 = 0.0001$, $p < 0.01$						
**Protozoa_Species = G. intestinalis**						
Ahmed and Abhay, 2015	5	142	0.04	[0.01; 0.08]	0.0%	0.1%
Akinbo et al., 2012	2	2000	0.00	[0.00; 0.00]	9.1%	0.1%
Morawski et al., 2017	12	202	0.06	[0.03; 0.10]	0.0%	0.1%
Oguntibeju 2006	5	60	0.08	[0.03; 0.18]	0.0%	0.1%
Paboriboune et al., 2014	12	137	0.09	[0.05; 0.15]	0.0%	0.1%
Sanyaolu et al., 2011	6	65	0.09	[0.03; 0.19]	0.0%	0.1%
Sarfati et al., 2006	1	154	0.01	[0.00; 0.04]	0.1%	0.1%
Sintayehu et al., 2013	21	343	0.06	[0.04; 0.09]	0.0%	0.1%
Sonali et al., 2014	4	250	0.02	[0.00; 0.04]	0.1%	0.1%
Stark et al., 2007	28	628	0.04	[0.03; 0.06]	0.1%	0.1%
Stensvold et al., 2011	2	96	0.02	[0.00; 0.07]	0.0%	0.1%
Tian et al., 2012	4	302	0.01	[0.00; 0.03]	0.1%	0.1%
Tian et al., 2013	1	79	0.01	[0.00; 0.07]	0.0%	0.1%
Wumba et al., 2010	3	175	0.02	[0.00; 0.05]	0.1%	0.1%
Yosefi et al., 2012	3	60	0.05	[0.01; 0.14]	0.0%	0.1%
Betancourth et al., 2021	2	102	0.02	[0.00; 0.07]	0.0%	0.1%
Common effect model		4795	0.00	[0.00; 0.00]	9.7%	--
Random effects model			0.03	[0.02; 0.04]	--	2.0%
Heterogeneity: $I^2 = 86\%$, $\tau^2 = 0.0004$, $p < 0.01$						
**Protozoa_Species = P. falciparum**						
Akinbo et al., 2016	32	411	0.08	[0.05; 0.11]	0.0%	0.1%
Akinbo et al., 2012	87	2000	0.04	[0.03; 0.05]	0.3%	0.1%
Ako-Nai et al., 2013	34	44	0.77	[0.62; 0.89]	0.0%	0.1%
Arundhati and Niharika, 2016	16	101	0.16	[0.09; 0.24]	0.0%	0.1%
Assir et al., 2014	22	85	0.26	[0.17; 0.37]	0.0%	0.1%
Bate et al., 2016	58	234	0.25	[0.19; 0.31]	0.0%	0.1%
Bharti et al., 2012	18	460	0.04	[0.02; 0.06]	0.1%	0.1%
Chalwe et al., 2009	42	55	0.76	[0.63; 0.87]	0.0%	0.1%
Chukwuanukwu et al., 2016	110	239	0.46	[0.40; 0.53]	0.0%	0.1%
Desta et al., 2005	3	49	0.06	[0.01; 0.17]	0.0%	0.1%
Hasang et al., 2014	100	216	0.46	[0.40; 0.53]	0.0%	0.1%
Kapito et al., 2011	70	1142	0.06	[0.05; 0.08]	0.1%	0.1%
Kfutwah et al., 2009	14	80	0.17	[0.10; 0.28]	0.0%	0.1%
Kinimi et al., 2018	8	97	0.08	[0.04; 0.16]	0.0%	0.1%
Kiying et al., 2010	120	160	0.75	[0.68; 0.81]	0.0%	0.1%
Mohapatra et al., 2017	19	333	0.06	[0.03; 0.09]	0.0%	0.1%
Morona et al., 2018	56	250	0.22	[0.17; 0.28]	0.0%	0.1%
Nkhoma et al., 2012	70	185	0.38	[0.31; 0.45]	0.0%	0.1%
Okonkwo et al., 2018	61	179	0.34	[0.27; 0.42]	0.0%	0.1%
Olaniran et al., 2012	83	93	0.89	[0.81; 0.95]	0.0%	0.1%
Omalu et al., 2012	27	30	0.90	[0.73; 0.98]	0.0%	0.1%
Omoti et al., 2013	254	342	0.74	[0.69; 0.79]	0.0%	0.1%
Onyenekwe et al., 2007	28	149	0.19	[0.13; 0.26]	0.0%	0.1%
Osuji et al., 2012	74	134	0.55	[0.46; 0.64]	0.0%	0.1%
Sanyaolu et al., 2013	29	65	0.45	[0.32; 0.57]	0.0%	0.1%
Tagoe and Boachie, 2012	34	220	0.15	[0.11; 0.21]	0.0%	0.1%
Anabire et al., 2019	36	191	0.19	[0.14; 0.25]	0.0%	0.1%
Onosakponome et al., 2020	300	300	1.00	[0.99; 1.00]	1.0%	0.1%
Sandie et al., 2021	31	190	0.16	[0.11; 0.22]	0.0%	0.1%
Stiffler et al., 2021	44	129	0.34	[0.26; 0.43]	0.0%	0.1%
Alonso et al., 2021	26	144	0.18	[0.12; 0.25]	0.0%	0.1%
Common effect model		8307	0.66	[0.65; 0.66]	1.7%	--
Random effects model			0.36	[0.25; 0.46]	--	3.8%
Heterogeneity: $I^2 = 100\%$, $\tau^2 = 0.0854$, $p = 0$						
**Protozoa_Species = Leishmania spp.**						
Almeida et al., 2012	5	10	0.50	[0.19; 0.81]	0.0%	0.1%
Carranza et al., 2009	26	163	0.16	[0.11; 0.22]	0.0%	0.1%
Cavalcanti et al., 2012	5	10	0.50	[0.19; 0.81]	0.0%	0.1%
Cota et al., 2013	41	113	0.36	[0.27; 0.46]	0.0%	0.1%
Garcia et al., 2006	28	92	0.30	[0.21; 0.41]	0.0%	0.1%
Júnior et al., 2015	7	15	0.47	[0.21; 0.73]	0.0%	0.1%
Pandey et al., 2018	176	305	0.58	[0.52; 0.63]	0.0%	0.1%
Shafiei et al., 2014	9	49	0.18	[0.09; 0.32]	0.0%	0.1%
Abdullahi et al., 2020	28	58	0.48	[0.35; 0.62]	0.0%	0.1%
Barbosa et al., 2020	65	113	0.58	[0.48; 0.67]	0.0%	0.1%
Barbosa et al., 2020	110	309	0.36	[0.30; 0.41]	0.0%	0.1%
Cunha et al., 2020	36	240	0.15	[0.11; 0.20]	0.0%	0.1%
Common effect model		1477	0.32	[0.30; 0.34]	0.0%	--
Random effects model			0.37	[0.27; 0.47]	--	1.3%
Heterogeneity: $I^2 = 95\%$, $\tau^2 = 0.0237$, $p < 0.01$						
**Protozoa_Species = T. cruzi**						
Almeida et al., 2010	9	716	0.01	[0.01; 0.02]	0.3%	0.1%
Llenas et al., 2012	4	155	0.03	[0.01; 0.06]	0.0%	0.1%
Rodrigues et al., 2011	2	19	0.11	[0.01; 0.33]	0.0%	0.1%
Rodrigues et al., 2005	18	28	0.64	[0.44; 0.81]	0.0%	0.1%
McAtee et al., 2021	32	116	0.28	[0.20; 0.37]	0.0%	0.1%
Common effect model		1034	0.02	[0.01; 0.03]	0.3%	--
Random effects model			0.20	[0.00; 0.42]	--	0.6%
Heterogeneity: $I^2 = 96\%$, $\tau^2 = 0.0604$, $p < 0.01$						
**Protozoa_Species = G. duodenalis**						
Asma et al., 2011	11	346	0.03	[0.02; 0.06]	0.1%	0.1%
Kurniawan et al., 2009	16	318	0.05	[0.03; 0.08]	0.0%	0.1%
Mtapuri et al., 2014	2	29	0.07	[0.01; 0.23]	0.0%	0.1%
Navarro et al., 2006	4	103	0.04	[0.01; 0.10]	0.0%	0.1%
Pavie et al., 2012	7	143	0.05	[0.02; 0.10]	0.0%	0.1%
Rodrigues et al., 2008	39	582	0.07	[0.05; 0.09]	0.1%	0.1%
Roka et al., 2012	32	260	0.12	[0.09; 0.17]	0.0%	0.1%
Roka et al., 2013	14	273	0.05	[0.03; 0.08]	0.0%	0.1%
Lengongo et al., 2020	2	115	0.02	[0.00; 0.06]	0.0%	0.1%
Barreto et al., 2022	5	178	0.03	[0.01; 0.06]	0.0%	0.1%
Common effect model		2347	0.05	[0.04; 0.05]	0.3%	--
Random effects model			0.05	[0.03; 0.07]	--	1.2%
Heterogeneity: $I^2 = 69\%$, $\tau^2 = 0.0005$, $p < 0.01$						
**Protozoa_Species = Cyclospora spp.**						
Asma et al., 2011	17	346	0.05	[0.03; 0.08]	0.0%	0.1%
Babatunde et al., 2010	16	90	0.18	[0.11; 0.27]	0.0%	0.1%
Getaneh et al., 2018	13	220	0.06	[0.03; 0.10]	0.0%	0.1%
Jaiswal et al., 2014	2	103	0.02	[0.00; 0.07]	0.0%	0.1%
Kammili et al., 2008	2	175	0.01	[0.00; 0.04]	0.1%	0.1%
Kulkarni et al., 2009	1	137	0.01	[0.00; 0.04]	0.1%	0.1%
Mathur et al., 2013	12	544	0.02	[0.01; 0.04]	0.1%	0.1%
Mitra et al., 2016	2	194	0.01	[0.00; 0.04]	0.1%	0.1%
Mohan et al., 2014	1	150	0.01	[0.00; 0.04]	0.1%	0.1%
Pennap et al., 2015	15	200	0.07	[0.04; 0.12]	0.0%	0.1%
Ramana et al., 2010	27	452	0.06	[0.04; 0.09]	0.0%	0.1%
Shimelis et al., 2016	2	491	0.00	[0.00; 0.01]	0.6%	0.1%
Tuli et al., 2008	88	366	0.24	[0.20; 0.29]	0.0%	0.1%
Chhangte et al., 2020	1	250	0.00	[0.00; 0.02]	0.2%	0.1%
Namaji et al., 2020	7	361	0.02	[0.01; 0.04]	0.1%	0.1%
Ajayi et al., 2021	2	375	0.01	[0.00; 0.02]	0.3%	0.1%
Common effect model		4454	0.01	[0.01; 0.01]	1.8%	--
Random effects model			0.04	[0.01; 0.07]	--	2.0%
Heterogeneity: $I^2 = 92\%$, $\tau^2 = 0.0035$, $p < 0.01$						
**Protozoa_Species = T. hominis**						
Assefa et al., 2009	2	214	0.01	[0.00; 0.03]	0.1%	0.1%
Mtapuri et al., 2014	1	29	0.03	[0.00; 0.18]	0.0%	0.1%
Stark et al., 2007	2	628	0.00	[0.00; 0.01]	0.9%	0.1%
Betancourth et al., 2021	1	102	0.01	[0.00; 0.05]	0.0%	0.1%
Common effect model		973	0.00	[0.00; 0.01]	1.1%	--
Random effects model			0.00	[0.00; 0.01]	--	0.5%
Heterogeneity: $I^2 = 0\%$, $\tau^2 = 0$, $p = 0.58$						
**Protozoa_Species = Entamoeba spp.**						
Assefa et al., 2013	43	272	0.16	[0.12; 0.21]	0.0%	0.1%
Jaiswal et al., 2014	11	103	0.11	[0.05; 0.18]	0.0%	0.1%
Mitra et al., 2016	38	194	0.20	[0.14; 0.26]	0.0%	0.1%
Nkenfou et al., 2017	10	68	0.15	[0.07; 0.25]	0.0%	0.1%
Roka et al., 2013	101	273	0.37	[0.31; 0.43]	0.0%	0.1%
Samie et al., 2014	40	151	0.26	[0.20; 0.34]	0.0%	0.1%
Tian et al., 2012	5	302	0.02	[0.01; 0.04]	0.1%	0.1%
Tian et al., 2013	3	79	0.04	[0.01; 0.11]	0.0%	0.1%
Tuli et al., 2008	11	366	0.03	[0.02; 0.05]	0.1%	0.1%
Sannella et al., 2019	2	155	0.01	[0.00; 0.05]	0.1%	0.1%
Wolday et al., 2021	109	751	0.15	[0.12; 0.17]	0.0%	0.1%
Common effect model		2714	0.05	[0.05; 0.06]	0.3%	--
Random effects model			0.13	[0.07; 0.20]	--	1.4%
Heterogeneity: $I^2 = 97\%$, $\tau^2 = 0.0120$, $p < 0.01$						
**Protozoa_Species = Isospora spp.**						
Babatunde et al., 2010	10	90	0.11	[0.05; 0.19]	0.0%	0.1%
Jaiswal et al., 2014	3	103	0.03	[0.01; 0.08]	0.0%	0.1%
Kammili et al., 2008	28	175	0.16	[0.11; 0.22]	0.0%	0.1%
Mehta et al., 2013	18	100	0.18	[0.11; 0.27]	0.0%	0.1%
Mitra et al., 2016	28	194	0.14	[0.10; 0.20]	0.0%	0.1%
Ramana et al., 2010	46	452	0.10	[0.08; 0.13]	0.0%	0.1%
Stensvold et al., 2011	1	96	0.01	[0.00; 0.06]	0.0%	0.1%
Sannella et al., 2019	4	155	0.03	[0.01; 0.06]	0.0%	0.1%
Ajayi et al., 2021	5	375	0.01	[0.00; 0.03]	0.1%	0.1%
Common effect model		1740	0.03	[0.03; 0.04]	0.3%	--
Random effects model			0.08	[0.04; 0.12]	--	1.1%
Heterogeneity: $I^2 = 92\%$, $\tau^2 = 0.0039$, $p < 0.01$						
**Protozoa_Species = I. butschlii**						
Babatunde et al., 2010	7	90	0.08	[0.03; 0.15]	0.0%	0.1%
Barcelos et al., 2018	1	90	0.01	[0.00; 0.06]	0.0%	0.1%
Gupta et al., 2013	1	100	0.01	[0.00; 0.05]	0.0%	0.1%
Moran et al., 2005	3	203	0.01	[0.00; 0.04]	0.1%	0.1%
Mtapuri et al., 2014	1	29	0.03	[0.00; 0.18]	0.0%	0.1%
Ngum et al., 2015	7	320	0.02	[0.01; 0.04]	0.1%	0.1%
Nkenfou et al., 2017	3	68	0.04	[0.01; 0.12]	0.0%	0.1%
Patel et al., 2011	1	100	0.01	[0.00; 0.05]	0.0%	0.1%
Rodrigues et al., 2008	37	582	0.06	[0.05; 0.09]	0.1%	0.1%
Roka et al., 2012	1	260	0.00	[0.00; 0.02]	0.3%	0.1%
Roka et al., 2013	16	273	0.06	[0.03; 0.09]	0.0%	0.1%
Sanyaolu et al., 2011	2	65	0.03	[0.00; 0.11]	0.0%	0.1%
Stark et al., 2007	8	628	0.01	[0.01; 0.02]	0.3%	0.1%
Wumba et al., 2012	1	242	0.00	[0.00; 0.02]	0.2%	0.1%
Abange et al., 2020	2	119	0.02	[0.00; 0.06]	0.0%	0.1%
Lengongo et al., 2020	1	115	0.01	[0.00; 0.05]	0.1%	0.1%
Barreto et al., 2022	2	178	0.01	[0.00; 0.04]	0.1%	0.1%
Betancourth et al., 2021	1	102	0.01	[0.00; 0.05]	0.0%	0.1%
Common effect model		3564	0.01	[0.01; 0.02]	1.3%	--
Random effects model			0.02	[0.01; 0.03]	--	2.2%
Heterogeneity: $I^2 = 69\%$, $\tau^2 = 0.0002$, $p < 0.01$						
**Protozoa_Species = E. hartmanni**						
Barcelos et al., 2018	7	90	0.08	[0.03; 0.15]	0.0%	0.1%
Paboriboune et al., 2014	5	137	0.04	[0.01; 0.08]	0.0%	0.1%
Roka et al., 2012	1	260	0.00	[0.00; 0.02]	0.3%	0.1%
Roka et al., 2013	1	273	0.00	[0.00; 0.02]	0.3%	0.1%
Stark et al., 2007	9	628	0.01	[0.01; 0.03]	0.2%	0.1%
Betancourth et al., 2021	1	102	0.01	[0.00; 0.05]	0.0%	0.1%
Common effect model		1490	0.01	[0.00; 0.01]	0.9%	--
Random effects model			0.01	[0.00; 0.02]	--	0.7%
Heterogeneity: $I^2 = 63\%$, $\tau^2 = < 0.0001$, $p = 0.02$						
**Protozoa_Species = E. nana**						
Barcelos et al., 2018	15	90	0.17	[0.10; 0.26]	0.0%	0.1%
Cardoso et al., 2011	3	500	0.01	[0.00; 0.02]	0.4%	0.1%
Gupta et al., 2013	2	100	0.02	[0.00; 0.07]	0.0%	0.1%
Moran et al., 2005	13	203	0.06	[0.03; 0.11]	0.0%	0.1%
Mtapuri et al., 2014	1	29	0.03	[0.00; 0.18]	0.0%	0.1%
Rodrigues et al., 2008	38	582	0.07	[0.05; 0.09]	0.1%	0.1%
Roka et al., 2012	13	260	0.05	[0.03; 0.08]	0.0%	0.1%
Roka et al., 2013	58	273	0.21	[0.17; 0.27]	0.0%	0.1%
Stark et al., 2007	62	628	0.10	[0.08; 0.12]	0.0%	0.1%
Tamirat et al., 2017	1	226	0.00	[0.00; 0.02]	0.2%	0.1%
Wumba et al., 2012	4	242	0.02	[0.00; 0.04]	0.1%	0.1%
Yosefi et al., 2012	5	60	0.08	[0.03; 0.18]	0.0%	0.1%
Abange et al., 2020	2	119	0.02	[0.00; 0.06]	0.0%	0.1%
Lengongo et al., 2020	1	115	0.01	[0.00; 0.05]	0.1%	0.1%
Barreto et al., 2022	18	178	0.10	[0.06; 0.16]	0.0%	0.1%
Betancourth et al., 2021	7	102	0.07	[0.03; 0.14]	0.0%	0.1%
Common effect model		3707	0.02	[0.01; 0.02]	1.0%	--
Random effects model			0.06	[0.03; 0.09]	--	2.0%
Heterogeneity: $I^2 = 92\%$, $\tau^2 = 0.0028$, $p < 0.01$						
**Protozoa_Species = C. belli**						
Barcelos et al., 2018	1	90	0.01	[0.00; 0.06]	0.0%	0.1%
Ngum et al., 2015	6	320	0.02	[0.01; 0.04]	0.1%	0.1%
Paboriboune et al., 2014	6	137	0.04	[0.02; 0.09]	0.0%	0.1%
Chhangte et al., 2020	3	250	0.01	[0.00; 0.03]	0.1%	0.1%
Namaji et al., 2020	30	361	0.08	[0.06; 0.12]	0.0%	0.1%
Betancourth et al., 2021	1	102	0.01	[0.00; 0.05]	0.0%	0.1%
Botero et al., 2021	1	192	0.01	[0.00; 0.03]	0.1%	0.1%
Common effect model		1452	0.01	[0.01; 0.02]	0.5%	--
Random effects model			0.02	[0.00; 0.04]	--	0.9%
Heterogeneity: $I^2 = 79\%$, $\tau^2 = 0.0005$, $p < 0.01$						
**Protozoa_Species = C. mesnili**						
Berenji et al., 2010	7	31	0.23	[0.10; 0.41]	0.0%	0.1%
Daryani et al., 2009	1	64	0.02	[0.00; 0.08]	0.0%	0.1%
Gupta et al., 2013	1	100	0.01	[0.00; 0.05]	0.0%	0.1%
Rodrigues et al., 2008	3	582	0.01	[0.00; 0.01]	0.6%	0.1%
Roka et al., 2012	11	260	0.04	[0.02; 0.07]	0.0%	0.1%
Roka et al., 2013	3	273	0.01	[0.00; 0.03]	0.1%	0.1%
Stark et al., 2007	3	628	0.00	[0.00; 0.01]	0.6%	0.1%
Lengongo et al., 2020	5	115	0.04	[0.01; 0.10]	0.0%	0.1%
Barreto et al., 2022	1	178	0.01	[0.00; 0.03]	0.1%	0.1%
Betancourth et al., 2021	1	102	0.01	[0.00; 0.05]	0.0%	0.1%
Common effect model		2333	0.01	[0.00; 0.01]	1.6%	--
Random effects model			0.01	[0.00; 0.01]	--	1.2%
Heterogeneity: $I^2 = 59\%$, $\tau^2 = 0$, $p < 0.01$						
**Protozoa_Species = Enteromonas spp.**						
Berenji et al., 2010	1	31	0.03	[0.00; 0.17]	0.0%	0.1%
Common effect model		31	0.03	[0.00; 0.09]	0.0%	--
Random effects model			0.03	[0.00; 0.09]	--	0.1%
Heterogeneity: not applicable						
**Protozoa_Species = P. vivax**						
Bharti et al., 2012	33	460	0.07	[0.05; 0.10]	0.0%	0.1%
Desta et al., 2005	5	49	0.10	[0.03; 0.22]	0.0%	0.1%
Magalhaes et al., 2014	44	628	0.07	[0.05; 0.09]	0.1%	0.1%
Mohapatra et al., 2017	4	333	0.01	[0.00; 0.03]	0.1%	0.1%
Common effect model		1470	0.03	[0.03; 0.04]	0.2%	--
Random effects model			0.06	[0.02; 0.09]	--	0.5%
Heterogeneity: $I^2 = 92\%$, $\tau^2 = 0.0011$, $p < 0.01$						
**Protozoa_Species = L. infantum**						
Colomba et al., 2009	24	145	0.17	[0.11; 0.24]	0.0%	0.1%
Rezaei et al., 2018	19	251	0.08	[0.05; 0.12]	0.0%	0.1%
Common effect model		396	0.10	[0.07; 0.12]	0.0%	--
Random effects model			0.12	[0.03; 0.20]	--	0.2%
Heterogeneity: $I^2 = 85\%$, $\tau^2 = 0.0034$, $p = 0.01$						
**Protozoa_Species = T. vaginalis**						
Davis et al., 2016	16	44	0.36	[0.22; 0.52]	0.0%	0.1%
De Lemos and Garcia-Zapata, 2010	23	125	0.18	[0.12; 0.26]	0.0%	0.1%
Kissinger et al., 2008	11	60	0.18	[0.10; 0.30]	0.0%	0.1%
Lemos et al., 2009	23	125	0.18	[0.12; 0.26]	0.0%	0.1%
Masese et al., 2011	31	147	0.21	[0.15; 0.29]	0.0%	0.1%
Nangammbi et al., 2013	32	155	0.21	[0.15; 0.28]	0.0%	0.1%
Nwadioha et al., 2012	84	350	0.24	[0.20; 0.29]	0.0%	0.1%
Nweze and Mouneke, 2011	355	970	0.37	[0.34; 0.40]	0.0%	0.1%
Price et al., 2018	76	359	0.21	[0.17; 0.26]	0.0%	0.1%
Medina et al., 2020	86	427	0.20	[0.16; 0.24]	0.0%	0.1%
Common effect model		2762	0.25	[0.24; 0.27]	0.1%	--
Random effects model			0.23	[0.19; 0.27]	--	1.2%
Heterogeneity: $I^2 = 89\%$, $\tau^2 = 0.0035$, $p < 0.01$						
**Protozoa_Species = C. cyatinis**						
Eshetu et al., 2017	6	223	0.03	[0.01; 0.06]	0.0%	0.1%
Tegegne et al., 2017	6	223	0.03	[0.01; 0.06]	0.0%	0.1%
Common effect model		446	0.03	[0.01; 0.04]	0.1%	--
Random effects model			0.03	[0.01; 0.04]	--	0.2%
Heterogeneity: $I^2 = 0\%$, $\tau^2 = 0$, $p = 1.00$						
**Protozoa_Species = Trichomonas spp.**						
Gupta et al., 2013	2	100	0.02	[0.00; 0.07]	0.0%	0.1%
Common effect model		100	0.02	[0.00; 0.05]	0.0%	--
Random effects model			0.02	[0.00; 0.05]	--	0.1%
Heterogeneity: not applicable						
**Protozoa_Species = Blastocystis spp.**						
Haileeyesus et al., 2013	24	378	0.06	[0.04; 0.09]	0.0%	0.1%
Mariam et al., 2008	2	109	0.02	[0.00; 0.06]	0.0%	0.1%
Lengongo et al., 2020	26	115	0.23	[0.15; 0.31]	0.0%	0.1%
Botero et al., 2021	23	192	0.12	[0.08; 0.17]	0.0%	0.1%
Common effect model		794	0.06	[0.04; 0.08]	0.1%	--
Random effects model			0.10	[0.02; 0.19]	--	0.5%
Heterogeneity: $I^2 = 91\%$, $\tau^2 = 0.0067$, $p < 0.01$						
**Protozoa_Species = D. fragilis**						
Huibers et al., 2018	6	35	0.17	[0.07; 0.34]	0.0%	0.1%
Stark et al., 2007	2	628	0.00	[0.00; 0.01]	0.9%	0.1%
Stensvold et al., 2011	3	96	0.03	[0.01; 0.09]	0.0%	0.1%
Yosefi et al., 2012	2	60	0.03	[0.00; 0.12]	0.0%	0.1%
Common effect model		819	0.00	[0.00; 0.01]	0.9%	--
Random effects model			0.03	[0.00; 0.06]	--	0.5%
Heterogeneity: $I^2 = 73\%$, $\tau^2 = 0.0006$, $p = 0.01$						
**Protozoa_Species = Giardia spp.**						
Jaiswal et al., 2014	7	103	0.07	[0.03; 0.14]	0.0%	0.1%
Jayalakshmi et al., 2008	3	89	0.03	[0.01; 0.10]	0.0%	0.1%
Mitra et al., 2016	20	194	0.10	[0.06; 0.15]	0.0%	0.1%
Tuli et al., 2008	37	366	0.10	[0.07; 0.14]	0.0%	0.1%
Gebrecherkos et al., 2019	7	150	0.05	[0.02; 0.09]	0.0%	0.1%
Sannella et al., 2019	3	155	0.02	[0.00; 0.06]	0.0%	0.1%
Purbey., 2021	1	75	0.01	[0.00; 0.07]	0.0%	0.1%
Common effect model		1132	0.04	[0.03; 0.06]	0.1%	--
Random effects model			0.05	[0.03; 0.08]	--	0.9%
Heterogeneity: $I^2 = 81\%$, $\tau^2 = 0.0011$, $p < 0.01$						
**Protozoa_Species = P. ovale**						
Kapito et al., 2011	1	1142	0.00	[0.00; 0.00]	4.9%	0.1%
Common effect model		1142	0.00	[0.00; 0.00]	4.9%	--
Random effects model			0.00	[0.00; 0.00]	--	0.1%
Heterogeneity: not applicable						
**Protozoa_Species = E. dispar**						
Moran et al., 2009	7	100	0.07	[0.03; 0.14]	0.0%	0.1%
Moran et al., 2005	35	203	0.17	[0.12; 0.23]	0.0%	0.1%
Stensvold et al., 2011	10	96	0.10	[0.05; 0.18]	0.0%	0.1%
Purbey., 2021	1	75	0.01	[0.00; 0.07]	0.0%	0.1%
Common effect model		474	0.06	[0.04; 0.08]	0.0%	--
Random effects model			0.09	[0.02; 0.15]	--	0.5%
Heterogeneity: $I^2 = 91\%$, $\tau^2 = 0.0041$, $p < 0.01$						
**Protozoa_Species = Cryptosporidium Spp.**						
Morawski et al., 2017	1	202	0.00	[0.00; 0.03]	0.2%	0.1%
Common effect model		202	0.00	[0.00; 0.01]	0.2%	--
Random effects model			0.00	[0.00; 0.01]	--	0.1%
Heterogeneity: not applicable						
**Protozoa_Species = L. braziliensis**						
Oliveira et al., 2011	13	169	0.08	[0.04; 0.13]	0.0%	0.1%
Common effect model		169	0.08	[0.04; 0.12]	0.0%	--
Random effects model			0.08	[0.04; 0.12]	--	0.1%
Heterogeneity: not applicable						
**Protozoa_Species = Blastocystis Spp.**						
Paboriboune et al., 2014	36	137	0.26	[0.19; 0.34]	0.0%	0.1%
Saksirisampant et al., 2009	2	90	0.02	[0.00; 0.08]	0.0%	0.1%
Stensvold et al., 2011	22	96	0.23	[0.15; 0.33]	0.0%	0.1%
Common effect model		323	0.07	[0.05; 0.10]	0.0%	--
Random effects model			0.17	[0.02; 0.32]	--	0.4%
Heterogeneity: $I^2 = 96\%$, $\tau^2 = 0.0167$, $p < 0.01$						
**Protozoa_Species = P. hominis**						
Roka et al., 2012	11	260	0.04	[0.02; 0.07]	0.0%	0.1%
Roka et al., 2013	7	273	0.03	[0.01; 0.05]	0.1%	0.1%
Common effect model		533	0.03	[0.02; 0.05]	0.1%	--
Random effects model			0.03	[0.02; 0.05]	--	0.2%
Heterogeneity: $I^2 = 11\%$, $\tau^2 = < 0.0001$, $p = 0.29$						
**Protozoa_Species = P. malariae**						
Sanyaolu et al., 2013	6	65	0.09	[0.03; 0.19]	0.0%	0.1%
Common effect model		65	0.09	[0.02; 0.16]	0.0%	--
Random effects model			0.09	[0.02; 0.16]	--	0.1%
Heterogeneity: not applicable						
**Protozoa_Species = E. hominis**						
Stark et al., 2007	10	628	0.02	[0.01; 0.03]	0.2%	0.1%
Common effect model		628	0.02	[0.01; 0.03]	0.2%	--
Random effects model			0.02	[0.01; 0.03]	--	0.1%
Heterogeneity: not applicable						
**Protozoa_Species = R. hominis**						
Stark et al., 2007	1	628	0.00	[0.00; 0.01]	1.5%	0.1%
Common effect model		628	0.00	[0.00; 0.00]	1.5%	--
Random effects model			0.00	[0.00; 0.00]	--	0.1%
Heterogeneity: not applicable						
**Protozoa_Species = C. suis**						
Wang et al., 2013	1	683	0.00	[0.00; 0.01]	1.8%	0.1%
Sannella et al., 2019	6	155	0.04	[0.01; 0.08]	0.0%	0.1%
Common effect model		838	0.00	[0.00; 0.00]	1.8%	--
Random effects model			0.02	[0.00; 0.05]	--	0.2%
Heterogeneity: $I^2 = 83\%$, $\tau^2 = 0.0006$, $p = 0.02$						
**Protozoa_Species = S. hominis**						
Wumba et al., 2010	1	175	0.01	[0.00; 0.03]	0.1%	0.1%
Common effect model		175	0.01	[0.00; 0.02]	0.1%	--
Random effects model			0.01	[0.00; 0.02]	--	0.1%
Heterogeneity: not applicable						
**Protozoa_Species = Cystoisospora spp.**						
Gebrecherkos et al., 2019	2	150	0.01	[0.00; 0.05]	0.1%	0.1%
Common effect model		150	0.01	[0.00; 0.03]	0.1%	--
Random effects model			0.01	[0.00; 0.03]	--	0.1%
Heterogeneity: not applicable						
**Protozoa_Species = T. intestinalis**						
Abange et al., 2020	1	119	0.01	[0.00; 0.05]	0.1%	0.1%
Common effect model		119	0.01	[0.00; 0.02]	0.1%	--
Random effects model			0.01	[0.00; 0.02]	--	0.1%
Heterogeneity: not applicable						
**Protozoa_Species = L. siamensis**						
Charoensakulchai et al., 2020	63	526	0.12	[0.09; 0.15]	0.0%	0.1%
Common effect model		526	0.12	[0.09; 0.15]	0.0%	--
Random effects model			0.12	[0.09; 0.15]	--	0.1%
Heterogeneity: not applicable						
**Protozoa_Species = L. martiniquensis**						
Charoensakulchai et al., 2020	24	526	0.05	[0.03; 0.07]	0.1%	0.1%
Common effect model		526	0.05	[0.03; 0.06]	0.1%	--
Random effects model			0.05	[0.03; 0.06]	--	0.1%
Heterogeneity: not applicable						
**Protozoa_Species = L. donovani**						
Charoensakulchai et al., 2020	23	526	0.04	[0.03; 0.06]	0.1%	0.1%
Common effect model		526	0.04	[0.03; 0.06]	0.1%	--
Random effects model			0.04	[0.03; 0.06]	--	0.1%
Heterogeneity: not applicable						
**Protozoa_Species = B. coli**						
Ajayi et al., 2021	4	375	0.01	[0.00; 0.03]	0.2%	0.1%
Common effect model		375	0.01	[0.00; 0.02]	0.2%	--
Random effects model			0.01	[0.00; 0.02]	--	0.1%
Heterogeneity: not applicable						
**Common effect model**		**201768**	**0.04**	**[0.04; 0.04]**	**100.0%**	**--**
**Random effects model**			**0.14**	**[0.12; 0.15]**	**--**	**100.0%**
Heterogeneity: $I^2 = 100\%$, $\tau^2 = 0.0357$, $p = 0$						

Test for subgroup differences (fixed effect): $\chi^2_{57} = 202652.36$, df = 57 (p = 0)

Test for subgroup differences (random effects): $\chi^2_{57} = 1375.16$, df = 57 ($p < 0.01$)

0 0.2 0.4 0.6 0.8 1

Figure S4A: Forest plots of the prevalence rates of helminth co-infections in HIV-infected people at genus level by meta-analysis using common effect and random-effects models

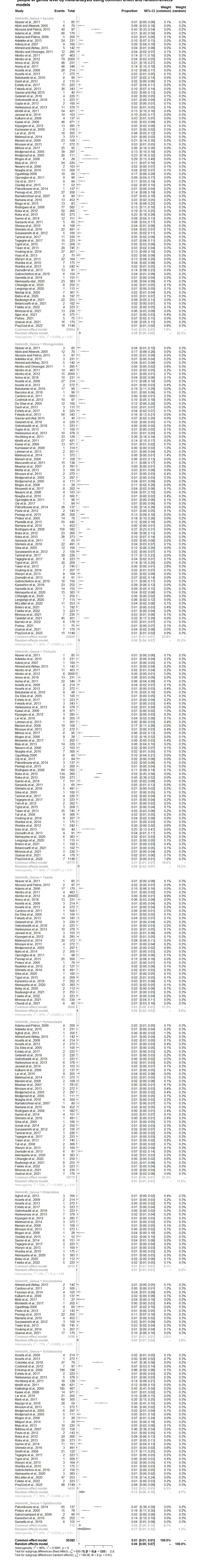

Figure S4B: rest plots of the prevalence rates of protozoan co-infections in HIV-infected people at genus level by meta-analysis using common effect and random-effects models

Study	Events	Total	Proportion	95%-CI	Weight (common)	Weight (random)
**Protozoa_Genus = Entamoeba**						
Abaver et al., 2011	9	85	0.11	[0.05; 0.19]	0.0%	0.1%
Abdollahi et al., 2015	28	91	0.31	[0.22; 0.41]	0.0%	0.1%
Abilo and Afework, 2005	3	70	0.04	[0.01; 0.12]	0.0%	0.1%
Adamu and Petros, 2009	26	200	0.13	[0.09; 0.18]	0.0%	0.1%
Adesij et al. 2007	6	100	0.06	[0.02; 0.13]	0.0%	0.1%
Agholi et al., 2013	7	356	0.02	[0.01; 0.04]	0.2%	0.1%
Ahmed and Abhay, 2015	7	142	0.05	[0.02; 0.10]	0.0%	0.1%
Akinbo et al., 2011	5	463	0.01	[0.00; 0.03]	0.5%	0.1%
Akinbo et al., 2012	6	2000	0.00	[0.00; 0.01]	7.2%	0.1%
Asma et al., 2011	58	346	0.17	[0.13; 0.21]	0.0%	0.1%
Assefa et al., 2009	57	214	0.27	[0.21; 0.33]	0.0%	0.1%
Assefa et al., 2013	43	272	0.16	[0.12; 0.21]	0.0%	0.1%
Babatunde et al., 2010	31	90	0.34	[0.25; 0.45]	0.0%	0.1%
Barcelos et al., 2018	14	90	0.16	[0.09; 0.25]	0.0%	0.1%
Berenji et al., 2010	3	31	0.10	[0.02; 0.26]	0.0%	0.1%
Cardoso et al., 2011	4	500	0.01	[0.00; 0.02]	0.7%	0.1%
Daryani et al., 2009	2	64	0.03	[0.00; 0.11]	0.0%	0.1%
Dash et al., 2013	9	115	0.08	[0.04; 0.14]	0.0%	0.1%
Eshetu et al., 2017	19	223	0.09	[0.05; 0.13]	0.0%	0.1%
Feyisayo et al., 2014	7	105	0.07	[0.03; 0.13]	0.0%	0.1%
Gaurav and Atul, 2015	2	40	0.05	[0.01; 0.17]	0.0%	0.1%
Gebretsadik et al., 2018	16	223	0.07	[0.04; 0.11]	0.0%	0.1%
Getaneh et al., 2018	8	220	0.04	[0.02; 0.07]	0.1%	0.1%
Gupta et al., 2013	11	100	0.11	[0.06; 0.19]	0.0%	0.1%
Haileeyesus et al., 2013	16	378	0.04	[0.02; 0.07]	0.1%	0.1%
Hung et al., 2008	66	991	0.07	[0.05; 0.08]	0.2%	0.1%
Jaiswal et al., 2014	11	103	0.11	[0.05; 0.18]	0.0%	0.1%
Jayalakshmi et al., 2008	3	89	0.03	[0.01; 0.10]	0.0%	0.1%
Javid et al., 2005	35	200	0.17	[0.13; 0.23]	0.0%	0.1%
Jegede et al., 2014	7	105	0.07	[0.03; 0.13]	0.0%	0.1%
Kipyegen et al., 2012	97	285	0.34	[0.29; 0.40]	0.0%	0.1%
Kiros et al., 2015	77	399	0.19	[0.16; 0.24]	0.0%	0.1%
Kulkarni et al., 2009	9	137	0.07	[0.03; 0.12]	0.0%	0.1%
Kurniawan et al., 2009	3	318	0.01	[0.00; 0.03]	0.3%	0.1%
Lar et al., 2016	33	205	0.16	[0.11; 0.22]	0.0%	0.1%
Lehman et al., 2013	6	201	0.03	[0.01; 0.06]	0.1%	0.1%
Mahmud et al., 2014	147	372	0.40	[0.35; 0.45]	0.0%	0.1%
Mariam et al., 2008	21	109	0.19	[0.12; 0.28]	0.0%	0.1%
Mathur et al., 2013	49	544	0.09	[0.07; 0.12]	0.1%	0.1%
Minti et al., 2017	1	27	0.04	[0.00; 0.19]	0.0%	0.1%
Missaye et al., 2013	43	272	0.16	[0.12; 0.21]	0.0%	0.1%
Mitra et al., 2016	38	194	0.20	[0.14; 0.26]	0.0%	0.1%
Moges et al., 2006	1	28	0.04	[0.00; 0.18]	0.0%	0.1%
Moran et al., 2009	31	100	0.31	[0.22; 0.41]	0.0%	0.1%
Moran et al., 2005	90	203	0.44	[0.37; 0.51]	0.0%	0.1%
Morawski et al., 2017	3	202	0.01	[0.00; 0.04]	0.1%	0.1%
Mtapuri et al., 2014	9	29	0.31	[0.15; 0.51]	0.0%	0.1%
Navarro et al., 2006	10	103	0.10	[0.05; 0.17]	0.0%	0.1%
Ngum et al., 2015	79	320	0.25	[0.20; 0.30]	0.0%	0.1%
Nkenfou et al., 2013	17	42	0.40	[0.26; 0.57]	0.0%	0.1%
Nkenfou et al., 2017	20	68	0.29	[0.19; 0.42]	0.0%	0.1%
Nsagha et al., 2016	22	300	0.07	[0.05; 0.11]	0.1%	0.1%
Nuhu et al., 2014	2	205	0.01	[0.00; 0.03]	0.2%	0.1%
Oguntibeju 2006	10	60	0.17	[0.08; 0.29]	0.0%	0.1%
Ojurongbe et al., 2011	56	96	0.58	[0.48; 0.68]	0.0%	0.1%
Paboriboune et al., 2014	23	137	0.17	[0.11; 0.24]	0.0%	0.1%
Patel et al., 2011	2	100	0.02	[0.00; 0.07]	0.0%	0.1%
Pavie et al., 2012	4	143	0.03	[0.01; 0.07]	0.1%	0.1%
Pennap et al., 2015	20	200	0.10	[0.06; 0.15]	0.0%	0.1%
Ramakrishnan et al., 2007	30	80	0.38	[0.27; 0.49]	0.0%	0.1%
Ramana et al., 2010	70	452	0.15	[0.12; 0.19]	0.0%	0.1%
Raytekar et al., 2012	13	127	0.10	[0.06; 0.17]	0.0%	0.1%
Ringo et al., 2015	4	83	0.05	[0.01; 0.12]	0.0%	0.1%
Rodrigues et al., 2008	107	582	0.18	[0.15; 0.22]	0.0%	0.1%
Roka et al., 2012	154	260	0.59	[0.53; 0.65]	0.0%	0.1%
Roka et al., 2013	135	273	0.49	[0.43; 0.56]	0.0%	0.1%
Samie et al., 2014	40	151	0.26	[0.20; 0.34]	0.0%	0.1%
Sanyaolu et al., 2011	11	65	0.17	[0.09; 0.28]	0.0%	0.1%
Sarfati et al., 2006	7	154	0.05	[0.02; 0.09]	0.0%	0.1%
Sherchan et al., 2012	8	146	0.05	[0.02; 0.11]	0.0%	0.1%
Shimelis et al., 2016	50	491	0.10	[0.08; 0.13]	0.1%	0.1%
Sintayehu et al., 2013	45	343	0.13	[0.10; 0.17]	0.0%	0.1%
Sonali et al., 2014	10	250	0.04	[0.02; 0.07]	0.1%	0.1%
Stark et al., 2007	47	628	0.07	[0.06; 0.10]	0.1%	0.1%
Stensvold et al., 2011	10	96	0.10	[0.05; 0.18]	0.0%	0.1%
Suryawanshi et al., 2012	9	100	0.09	[0.04; 0.16]	0.0%	0.1%
Tamirat et al., 2017	23	226	0.10	[0.07; 0.15]	0.0%	0.1%
Tegegne et al., 2017	19	223	0.09	[0.05; 0.13]	0.0%	0.1%
Tian et al., 2012	5	302	0.02	[0.01; 0.04]	0.2%	0.1%
Tian et al., 2013	3	79	0.04	[0.01; 0.11]	0.0%	0.1%
Tigist et al., 2015	7	209	0.03	[0.01; 0.07]	0.1%	0.1%
Tiwari et al. 2013	19	745	0.03	[0.02; 0.04]	0.3%	0.1%
Tuli et al., 2008	11	366	0.03	[0.02; 0.05]	0.1%	0.1%
Vouking et al., 2014	46	207	0.22	[0.17; 0.29]	0.0%	0.1%
Vyas et al., 2013	4	75	0.05	[0.01; 0.13]	0.0%	0.1%
Wanyiri et al., 2013	15	164	0.09	[0.05; 0.15]	0.0%	0.1%
Wumba et al., 2010	9	175	0.05	[0.02; 0.10]	0.0%	0.1%
Wumba et al., 2012	23	242	0.10	[0.06; 0.14]	0.0%	0.1%
Yosefi et al., 2012	7	60	0.12	[0.05; 0.23]	0.0%	0.1%
Zeynudin et al., 2013	9	91	0.10	[0.05; 0.18]	0.0%	0.1%
Gebrecherkos et al., 2019	31	150	0.21	[0.14; 0.28]	0.0%	0.1%
Sannella et al., 2019	2	155	0.01	[0.00; 0.05]	0.1%	0.1%
Abange et al., 2020	4	119	0.03	[0.01; 0.08]	0.0%	0.1%
Alemayehu et al., 2020	7	383	0.02	[0.01; 0.04]	0.2%	0.1%
Belay et al., 2020	8	112	0.07	[0.03; 0.14]	0.0%	0.1%
Chhangte et al., 2020	32	250	0.13	[0.09; 0.18]	0.0%	0.1%
Lengongo et al., 2020	17	115	0.15	[0.09; 0.23]	0.0%	0.1%
Namaji et al., 2020	2	361	0.01	[0.00; 0.02]	0.6%	0.1%
Purbey., 2021	4	75	0.05	[0.01; 0.13]	0.0%	0.1%
Ajayi et al., 2021	64	375	0.17	[0.13; 0.21]	0.0%	0.1%
Bayleyegn et al., 2021	25	255	0.10	[0.06; 0.14]	0.0%	0.1%
Betancourth et al., 2021	5	102	0.05	[0.02; 0.11]	0.0%	0.1%
Botero et al., 2021	25	192	0.13	[0.09; 0.19]	0.0%	0.1%
Chandi et al., 2021	5	80	0.06	[0.02; 0.14]	0.0%	0.1%
Feleke et al., 2022	12	223	0.05	[0.03; 0.09]	0.0%	0.1%
Miressa et al., 2021	23	230	0.10	[0.06; 0.15]	0.0%	0.1%
Common effect model		24027	0.02	[0.02; 0.02]	13.4%	--
Random effects model			0.12	[0.10; 0.15]	--	15.4%
Heterogeneity: $I^2 = 97\%$, $\tau^2 = 0.0131$, $p = 0$						
**Protozoa_Genus = Giardia**						
Abaver et al., 2011	3	85	0.04	[0.01; 0.10]	0.0%	0.1%
Abilo and Afework, 2005	6	70	0.09	[0.03; 0.18]	0.0%	0.1%
Adamu and Petros, 2009	32	200	0.16	[0.11; 0.22]	0.0%	0.1%
Agholi et al., 2013	23	356	0.06	[0.04; 0.10]	0.1%	0.1%
Ahmed and Abhay, 2015	5	142	0.04	[0.01; 0.08]	0.0%	0.1%
Akinbo et al., 2012	2	2000	0.00	[0.00; 0.00]	18.8%	0.1%
Asma et al., 2011	11	346	0.03	[0.02; 0.06]	0.1%	0.1%
Assefa et al., 2009	24	214	0.11	[0.07; 0.16]	0.0%	0.1%
Assefa et al., 2013	20	272	0.07	[0.05; 0.11]	0.0%	0.1%
Babatunde et al., 2010	16	90	0.18	[0.11; 0.27]	0.0%	0.1%
Barcelos et al., 2018	1	90	0.01	[0.00; 0.06]	0.1%	0.1%
Berenji et al., 2010	7	31	0.23	[0.10; 0.41]	0.0%	0.1%
Cardoso et al., 2011	5	500	0.01	[0.00; 0.02]	0.5%	0.1%
da Silva et al., 2005	1	100	0.01	[0.00; 0.05]	0.1%	0.1%
Daryani et al., 2009	2	64	0.03	[0.00; 0.11]	0.0%	0.1%
Dash et al., 2013	1	115	0.01	[0.00; 0.05]	0.1%	0.1%
Eshetu et al., 2017	4	223	0.02	[0.00; 0.05]	0.1%	0.1%
Gaurav and Atul, 2015	2	40	0.05	[0.01; 0.17]	0.0%	0.1%
Gebretsadik et al., 2018	3	223	0.01	[0.00; 0.04]	0.2%	0.1%
Getaneh et al., 2018	4	220	0.02	[0.00; 0.05]	0.1%	0.1%
Guk et al., 2005	1	105	0.01	[0.00; 0.05]	0.1%	0.1%
Gupta et al., 2013	8	100	0.08	[0.04; 0.15]	0.0%	0.1%
Haileeyesus et al., 2013	15	378	0.04	[0.02; 0.06]	0.1%	0.1%
Huibers et al., 2018	9	35	0.26	[0.12; 0.43]	0.0%	0.1%
Jaiswal et al., 2014	7	103	0.07	[0.03; 0.14]	0.0%	0.1%
Jayalakshmi et al., 2008	3	89	0.03	[0.01; 0.10]	0.0%	0.1%
Javid et al., 2005	45	200	0.22	[0.17; 0.29]	0.0%	0.1%
Kipyegen et al., 2012	25	285	0.09	[0.06; 0.13]	0.0%	0.1%
Kiros et al., 2015	17	399	0.04	[0.03; 0.07]	0.1%	0.1%
Kurniawan et al., 2009	16	318	0.05	[0.03; 0.08]	0.1%	0.1%
Mahmud et al., 2014	47	372	0.13	[0.09; 0.16]	0.0%	0.1%
Mariam et al., 2008	11	109	0.10	[0.05; 0.17]	0.0%	0.1%
Meamar et al., 2007	33	781	0.04	[0.03; 0.06]	0.2%	0.1%
Mehta et al., 2013	5	100	0.05	[0.02; 0.11]	0.0%	0.1%
Missaye et al., 2013	20	272	0.07	[0.05; 0.11]	0.0%	0.1%
Mitra et al., 2016	20	194	0.10	[0.06; 0.15]	0.0%	0.1%
Moges et al., 2006	2	28	0.07	[0.01; 0.24]	0.0%	0.1%
Moran et al., 2005	4	203	0.02	[0.01; 0.05]	0.1%	0.1%
Morawski et al., 2017	12	202	0.06	[0.03; 0.10]	0.0%	0.1%
Mtapuri et al., 2014	2	29	0.07	[0.01; 0.23]	0.0%	0.1%
Navarro et al., 2006	4	103	0.04	[0.01; 0.10]	0.0%	0.1%
Nkenfou et al., 2013	1	42	0.02	[0.00; 0.13]	0.0%	0.1%
Nkenfou et al., 2017	2	68	0.03	[0.00; 0.10]	0.0%	0.1%
Nsagha et al., 2016	10	300	0.03	[0.02; 0.06]	0.1%	0.1%
Nuhu et al., 2014	13	205	0.06	[0.03; 0.11]	0.0%	0.1%
Oguntibeju 2006	5	60	0.08	[0.03; 0.18]	0.0%	0.1%
Paboriboune et al., 2014	12	137	0.09	[0.05; 0.15]	0.0%	0.1%
Patel et al., 2011	2	100	0.02	[0.00; 0.07]	0.1%	0.1%
Pavie et al., 2012	7	143	0.05	[0.02; 0.10]	0.0%	0.1%
Pennap et al., 2015	12	200	0.06	[0.03; 0.10]	0.0%	0.1%
Pinlaor et al., 2005	1	78	0.01	[0.00; 0.07]	0.1%	0.1%
Ramakrishnan et al., 2007	3	80	0.04	[0.01; 0.11]	0.0%	0.1%
Ramana et al., 2010	34	452	0.08	[0.05; 0.10]	0.1%	0.1%
Raytekar et al., 2012	3	127	0.02	[0.00; 0.07]	0.1%	0.1%
Ringo et al., 2015	8	83	0.10	[0.04; 0.18]	0.0%	0.1%
Rodrigues et al., 2008	39	582	0.07	[0.05; 0.09]	0.1%	0.1%
Roka et al., 2012	32	260	0.12	[0.09; 0.17]	0.0%	0.1%
Roka et al., 2013	14	273	0.05	[0.03; 0.08]	0.1%	0.1%
Samie et al., 2014	18	151	0.12	[0.07; 0.18]	0.0%	0.1%
Sanyaolu et al., 2011	6	65	0.09	[0.03; 0.19]	0.0%	0.1%
Sarfati et al., 2006	1	154	0.01	[0.00; 0.04]	0.2%	0.1%
Sherchan et al., 2012	14	146	0.10	[0.05; 0.16]	0.0%	0.1%
Sherpa et al., 2010	3	106	0.03	[0.01; 0.08]	0.0%	0.1%
Shimelis et al., 2016	39	491	0.08	[0.06; 0.11]	0.1%	0.1%
Silva et al., 2005	1	100	0.01	[0.00; 0.05]	0.1%	0.1%
Sintayehu et al., 2013	21	343	0.06	[0.04; 0.09]	0.1%	0.1%
Sonali et al., 2014	4	250	0.02	[0.00; 0.04]	0.2%	0.1%
Stark et al., 2007	28	628	0.04	[0.03; 0.06]	0.2%	0.1%
Stensvold et al., 2011	2	96	0.02	[0.00; 0.07]	0.0%	0.1%
Suryawanshi et al., 2012	6	100	0.06	[0.02; 0.13]	0.0%	0.1%
Tamirat et al., 2017	8	226	0.04	[0.02; 0.07]	0.1%	0.1%
Tegegne et al., 2017	4	223	0.02	[0.00; 0.05]	0.1%	0.1%
Tian et al., 2012	4	302	0.01	[0.00; 0.03]	0.2%	0.1%
Tian et al., 2013	1	79	0.01	[0.00; 0.07]	0.1%	0.1%
Tigist et al., 2015	31	209	0.15	[0.10; 0.20]	0.0%	0.1%
Tiwari et al. 2013	26	745	0.03	[0.02; 0.05]	0.3%	0.1%
Tuli et al., 2008	37	366	0.10	[0.07; 0.14]	0.0%	0.1%
Vyas et al., 2013	3	75	0.04	[0.01; 0.11]	0.0%	0.1%
Wanyiri et al., 2013	13	164	0.08	[0.04; 0.13]	0.0%	0.1%
Wumba et al., 2010	3	175	0.02	[0.00; 0.05]	0.1%	0.1%
Wumba et al., 2012	2	242	0.01	[0.00; 0.03]	0.3%	0.1%
Yosefi et al., 2012	3	60	0.05	[0.01; 0.14]	0.0%	0.1%
Zeynudin et al., 2013	8	91	0.09	[0.04; 0.17]	0.0%	0.1%
Gebrecherkos et al., 2019	13	150	0.09	[0.05; 0.14]	0.0%	0.1%
Masoumi et al., 2019	1	102	0.01	[0.00; 0.05]	0.1%	0.1%
Sannella et al., 2019	3	155	0.02	[0.00; 0.06]	0.1%	0.1%
Abange et al., 2020	5	119	0.04	[0.01; 0.10]	0.0%	0.1%
Alemayehu et al., 2020	8	383	0.02	[0.01; 0.04]	0.2%	0.1%
Ayele et al., 2020	16	180	0.09	[0.05; 0.14]	0.0%	0.1%
Belay et al., 2020	4	112	0.04	[0.01; 0.09]	0.0%	0.1%
Chhangte et al., 2020	7	250	0.03	[0.01; 0.06]	0.1%	0.1%
Lengongo et al., 2020	2	115	0.02	[0.00; 0.06]	0.1%	0.1%
Namaji et al., 2020	1	361	0.00	[0.00; 0.02]	1.0%	0.1%
Purbey., 2021	1	75	0.01	[0.00; 0.07]	0.1%	0.1%
Bayleyegn et al., 2021	3	255	0.01	[0.00; 0.03]	0.2%	0.1%
Betancourth et al., 2021	2	102	0.02	[0.00; 0.07]	0.1%	0.1%
Feleke et al., 2022	4	223	0.02	[0.00; 0.05]	0.1%	0.1%
Miressa et al., 2021	50	230	0.22	[0.17; 0.28]	0.0%	0.1%
Common effect model		21375	0.01	[0.01; 0.01]	26.6%	--
Random effects model			0.05	[0.04; 0.06]	--	14.4%
Heterogeneity: $I^2 = 90\%$, $\tau^2 = 0.0014$, $p < 0.01$						
**Protozoa_Genus = Toxoplasma**						
Abdollahi et al., 2013	65	100	0.65	[0.55; 0.74]	0.0%	0.1%
Addebbous et al., 2012	59	95	0.62	[0.52; 0.72]	0.0%	0.1%
Aghaee et al., 2017	11	49	0.22	[0.12; 0.37]	0.0%	0.1%
Alavi et al., 2013	31	42	0.74	[0.58; 0.86]	0.0%	0.1%
Amuta et al., 2012	39	360	0.11	[0.08; 0.15]	0.0%	0.1%
Angal et al., 2016	84	133	0.63	[0.54; 0.71]	0.0%	0.1%
Anteneh et al., 2014	178	190	0.94	[0.89; 0.97]	0.0%	0.1%
Arefkhah et al., 2018	42	251	0.17	[0.12; 0.22]	0.0%	0.1%
Assob et al., 2011	93	133	0.70	[0.61; 0.78]	0.0%	0.1%
Ayi et al., 2016	72	125	0.58	[0.48; 0.66]	0.0%	0.1%
Bamba et al., 2014	11	16	0.69	[0.41; 0.89]	0.0%	0.1%
Beena et al., 2015	141	661	0.21	[0.18; 0.25]	0.0%	0.1%
Bessong and Mathomu 2010	29	160	0.18	[0.12; 0.25]	0.0%	0.1%
Chemoh et al., 2015	109	300	0.36	[0.31; 0.42]	0.0%	0.1%
Daryani et al., 2011	54	62	0.87	[0.76; 0.94]	0.0%	0.1%
Davarpanah et al., 2007	38	208	0.18	[0.13; 0.24]	0.0%	0.1%
Dechassa et al., 2016	109	135	0.81	[0.73; 0.87]	0.0%	0.1%
Domingos et al., 2013	92	200	0.46	[0.39; 0.53]	0.0%	0.1%
Ebrahim et al., 2018	51	246	0.21	[0.16; 0.26]	0.0%	0.1%
Fisseha et al., 2013	90	103	0.87	[0.79; 0.93]	0.0%	0.1%
Ganiem et al., 2013	21	64	0.33	[0.22; 0.46]	0.0%	0.1%
Hari et al., 2007	25	307	0.08	[0.05; 0.12]	0.0%	0.1%
Hung et al., 2005	56	550	0.10	[0.08; 0.13]	0.1%	0.1%
Kodym et al., 2015	502	1235	0.41	[0.38; 0.43]	0.1%	0.1%
Lago et al., 2009	121	168	0.72	[0.65; 0.79]	0.0%	0.1%
Li et al., 2010	58	183	0.32	[0.25; 0.39]	0.0%	0.1%
Machala et al., 2009	268	626	0.43	[0.39; 0.47]	0.0%	0.1%
Malla et al., 2005	14	100	0.14	[0.08; 0.22]	0.0%	0.1%
Mengistu et al., 2014	38	43	0.88	[0.75; 0.96]	0.0%	0.1%
Mohraz et al., 2011	100	201	0.50	[0.43; 0.57]	0.0%	0.1%
Nazari et al., 2018	167	385	0.43	[0.38; 0.48]	0.0%	0.1%
Ngobeni and Samie, 2017	38	100	0.38	[0.28; 0.48]	0.0%	0.1%
Nissapatorn et al., 2007	329	693	0.47	[0.44; 0.51]	0.0%	0.1%
Ogoina et al., 2013	43	111	0.39	[0.30; 0.48]	0.0%	0.1%
Oshinaike et al., 2010	71	83	0.86	[0.76; 0.92]	0.0%	0.1%
Osunkalu et al., 2011	206	380	0.54	[0.49; 0.59]	0.0%	0.1%
Ouermi et al., 2009	44	138	0.32	[0.24; 0.40]	0.0%	0.1%
Pappoe et al., 2017	293	394	0.74	[0.70; 0.79]	0.0%	0.1%
Rahimi et al., 2015	79	82	0.96	[0.90; 0.99]	0.0%	0.1%
Reema and Dharmendra, 2017	52	100	0.52	[0.42; 0.62]	0.0%	0.1%
Rezanezhad et al., 2017	19	90	0.21	[0.13; 0.31]	0.0%	0.1%
Rostami et al., 2014	19	94	0.20	[0.13; 0.30]	0.0%	0.1%
Shafieenia et al., 2018	142	379	0.37	[0.33; 0.43]	0.0%	0.1%
Shafiei et al., 2011	49	121	0.40	[0.32; 0.50]	0.0%	0.1%
Shen et al., 2016	25	259	0.10	[0.06; 0.14]	0.0%	0.1%
Simpore et al., 2006	59	207	0.29	[0.22; 0.35]	0.0%	0.1%
Sitoe et al., 2010	18	58	0.31	[0.20; 0.45]	0.0%	0.1%
Tsegaye et al., 2014	150	170	0.88	[0.82; 0.93]	0.0%	0.1%
Xavier et al., 2013	200	250	0.80	[0.74; 0.85]	0.0%	0.1%
Zeleke and Melsew, 2017	255	270	0.94	[0.91; 0.97]	0.1%	0.1%
Bavand et al., 2019	66	149	0.44	[0.36; 0.53]	0.0%	0.1%
Bokharaei et al., 2020	95	108	0.88	[0.80; 0.93]	0.0%	0.1%
Dutta et al., 2020	292	400	0.73	[0.68; 0.77]	0.0%	0.1%
Hoshina et al., 2020	33	399	0.08	[0.06; 0.11]	0.1%	0.1%
Moro et al., 2020	81	332	0.24	[0.20; 0.29]	0.0%	0.1%
Zakari et al., 2020	54	160	0.34	[0.26; 0.42]	0.0%	0.1%
Steinberg et al., 2021	173	215	0.80	[0.75; 0.86]	0.0%	0.1%
Fang et al., 2021	164	325	0.50	[0.45; 0.56]	0.0%	0.1%
Sajedi et al., 2021	73	121	0.60	[0.51; 0.69]	0.0%	0.1%
Common effect model		13619	0.44	[0.43; 0.44]	1.0%	--
Random effects model			0.49	[0.42; 0.56]	--	8.5%
Heterogeneity: $I^2 = 99\%$, $\tau^2 = 0.0705$, $p = 0$						
**Protozoa_Genus = Cryptosporidium**						
Abilo and Afework, 2005	20	70	0.29	[0.18; 0.41]	0.0%	0.1%
Adamu and Petros, 2009	16	200	0.08	[0.05; 0.13]	0.0%	0.1%
Adamu et al., 2014	139	520	0.27	[0.23; 0.31]	0.0%	0.1%
Adesij et al. 2007	79	100	0.79	[0.70; 0.87]	0.0%	0.1%
Agholi et al., 2013	34	356	0.10	[0.07; 0.13]	0.0%	0.1%
Ahmed and Abhay, 2015	6	142	0.04	[0.02; 0.09]	0.0%	0.1%
Ajjampur et al., 2007	48	534	0.09	[0.07; 0.12]	0.1%	0.1%
Akinbo et al., 2010	36	400	0.09	[0.06; 0.12]	0.1%	0.1%
Akinbo et al., 2012	74	2000	0.04	[0.03; 0.05]	0.7%	0.1%
Alberto et al., 2011	38	131	0.29	[0.21; 0.38]	0.0%	0.1%
Amoo et al., 2018	52	231	0.23	[0.17; 0.28]	0.0%	0.1%
Asma et al., 2011	43	346	0.12	[0.09; 0.16]	0.0%	0.1%
Assefa et al., 2009	43	214	0.20	[0.15; 0.26]	0.0%	0.1%
Assefa et al., 2013	2	272	0.01	[0.00; 0.03]	0.4%	0.1%
Babatunde et al., 2010	29	90	0.32	[0.23; 0.43]	0.0%	0.1%
Barcelos et al., 2018	1	90	0.01	[0.00; 0.06]	0.1%	0.1%
Cardoso et al., 2011	1	500	0.00	[0.00; 0.01]	2.0%	0.1%
da Silva et al., 2005	4	100	0.04	[0.01; 0.10]	0.0%	0.1%
Daryani et al., 2009	6	64	0.09	[0.04; 0.19]	0.0%	0.1%
Dash et al., 2013	14	115	0.12	[0.07; 0.20]	0.0%	0.1%
Djieyep et al., 2014	127	305	0.42	[0.36; 0.47]	0.0%	0.1%
Echoru et al., 2015	4	111	0.04	[0.01; 0.09]	0.0%	0.1%
Erhabor et al., 2011	3	105	0.03	[0.01; 0.08]	0.0%	0.1%
Eshetu et al., 2017	7	223	0.03	[0.01; 0.06]	0.1%	0.1%
Gabriela et al., 2005	59	397	0.15	[0.12; 0.19]	0.0%	0.1%
Gaurav and Atul, 2015	6	40	0.15	[0.06; 0.30]	0.0%	0.1%
Gebretsadik et al., 2018	3	223	0.01	[0.00; 0.04]	0.2%	0.1%
Getaneh et al., 2018	19	220	0.09	[0.05; 0.13]	0.0%	0.1%
Ghafari et al., 2018	27	250	0.11	[0.07; 0.15]	0.0%	0.1%
Gholami et al., 2016	4	53	0.08	[0.02; 0.18]	0.0%	0.1%
Girma et al., 2014	92	268	0.34	[0.29; 0.40]	0.0%	0.1%
Guk et al., 2005	7	105	0.07	[0.03; 0.13]	0.0%	0.1%
Gupta et al., 2013	4	100	0.04	[0.01; 0.10]	0.0%	0.1%
Haileeyesus et al., 2013	32	378	0.08	[0.06; 0.12]	0.1%	0.1%
Huibers et al., 2018	4	35	0.11	[0.03; 0.27]	0.0%	0.1%
Hung et al., 2007	5	1376	0.00	[0.00; 0.01]	4.1%	0.1%
Hussain et al., 2016	25	127	0.20	[0.13; 0.28]	0.0%	0.1%
Iqbal et al., 2015	43	346	0.12	[0.09; 0.16]	0.0%	0.1%
Jaiswal et al., 2014	3	103	0.03	[0.01; 0.08]	0.0%	0.1%
Jayalakshmi et al., 2008	12	89	0.13	[0.07; 0.22]	0.0%	0.1%
Javid et al., 2005	84	200	0.42	[0.35; 0.49]	0.0%	0.1%
Kammili et al., 2008	12	175	0.07	[0.04; 0.12]	0.0%	0.1%
Kiros et al., 2015	23	399	0.06	[0.04; 0.09]	0.1%	0.1%
Kucerova et al., 2011	19	46	0.41	[0.27; 0.57]	0.0%	0.1%
Kulkarni et al., 2009	16	137	0.12	[0.07; 0.18]	0.0%	0.1%
Kurniawan et al., 2013	33	36	0.92	[0.78; 0.98]	0.0%	0.1%
Kurniawan et al., 2009	32	318	0.10	[0.07; 0.14]	0.0%	0.1%
Langoni and Souza, 2007	11	105	0.10	[0.05; 0.18]	0.0%	0.1%
Lar et al., 2016	17	205	0.08	[0.05; 0.13]	0.0%	0.1%
Lehman et al., 2013	13	201	0.06	[0.03; 0.11]	0.0%	0.1%
Maikai et al., 2012	1	61	0.02	[0.00; 0.09]	0.0%	0.1%
Mariam et al., 2008	8	109	0.07	[0.03; 0.14]	0.0%	0.1%
Masarat et al., 2012	45	45	1.00	[0.92; 1.00]	0.1%	0.1%
Mathur et al., 2013	135	544	0.25	[0.21; 0.29]	0.0%	0.1%
Meamar et al., 2007	7	781	0.01	[0.00; 0.02]	1.0%	0.1%
Mehta et al., 2013	2	100	0.02	[0.00; 0.07]	0.0%	0.1%
Mekonnen et al., 2014	92	268	0.34	[0.29; 0.40]	0.0%	0.1%
Missaye et al., 2013	2	272	0.01	[0.00; 0.03]	0.4%	0.1%
Mitra et al., 2016	57	194	0.29	[0.23; 0.36]	0.0%	0.1%
Mohan et al., 2014	2	150	0.01	[0.00; 0.05]	0.1%	0.1%
Morawski et al., 2017	1	202	0.00	[0.00; 0.03]	0.3%	0.1%
Mtapuri et al., 2014	5	29	0.17	[0.06; 0.36]	0.0%	0.1%
Ngum et al., 2015	46	320	0.14	[0.11; 0.19]	0.0%	0.1%
Nkenfou et al., 2013	8	42	0.19	[0.09; 0.34]	0.0%	0.1%
Nkenfou et al., 2017	5	68	0.07	[0.02; 0.16]	0.0%	0.1%
Nsagha et al., 2016	132	300	0.44	[0.38; 0.50]	0.0%	0.1%
Nuhu et al., 2014	19	205	0.09	[0.06; 0.14]	0.0%	0.1%
Oguntibeju 2006	6	60	0.10	[0.04; 0.21]	0.0%	0.1%
Ojuromi et al., 2016	5	90	0.06	[0.02; 0.12]	0.0%	0.1%
Olopade et al., 2017	10	226	0.04	[0.02; 0.08]	0.1%	0.1%
Oyedeji et al., 2015	10	52	0.19	[0.10; 0.33]	0.0%	0.1%
Paboriboune et al., 2014	9	137	0.07	[0.03; 0.12]	0.0%	0.1%
Patel et al., 2011	20	100	0.20	[0.13; 0.29]	0.0%	0.1%
Pavie et al., 2012	9	143	0.06	[0.03; 0.12]	0.0%	0.1%
Pennap et al., 2015	9	200	0.04	[0.02; 0.08]	0.1%	0.1%
Pinlaor et al., 2005	9	78	0.12	[0.05; 0.21]	0.0%	0.1%
Ramakrishnan et al., 2007	23	80	0.29	[0.19; 0.40]	0.0%	0.1%
Ramana et al., 2010	48	452	0.11	[0.08; 0.14]	0.1%	0.1%
Rashmi and Ravi, 2013	15	90	0.17	[0.10; 0.26]	0.0%	0.1%
Raytekar et al., 2012	9	127	0.07	[0.03; 0.13]	0.0%	0.1%
Rodrigues et al., 2008	39	582	0.07	[0.05; 0.09]	0.1%	0.1%
Roka et al., 2012	24	260	0.09	[0.06; 0.13]	0.0%	0.1%
Roka et al., 2013	8	273	0.03	[0.01; 0.06]	0.1%	0.1%
Saksirisampant et al., 2009	31	90	0.34	[0.25; 0.45]	0.0%	0.1%
Samie et al., 2014	40	151	0.26	[0.20; 0.34]	0.0%	0.1%
Sanyaolu et al., 2011	5	65	0.08	[0.03; 0.17]	0.0%	0.1%
Sarfati et al., 2006	6	154	0.04	[0.01; 0.08]	0.0%	0.1%
Sherchan et al., 2012	4	146	0.03	[0.01; 0.07]	0.1%	0.1%
Sherpa et al., 2010	8	106	0.08	[0.03; 0.14]	0.0%	0.1%
Shimelis and Tadesse, 2014	32	250	0.13	[0.09; 0.18]	0.0%	0.1%
Shimelis et al., 2016	65	491	0.13	[0.10; 0.17]	0.0%	0.1%
Silva et al., 2005	4	100	0.04	[0.01; 0.10]	0.0%	0.1%
Sintayehu et al., 2013	10	343	0.03	[0.01; 0.05]	0.1%	0.1%
Sonali et al., 2014	52	250	0.21	[0.16; 0.26]	0.0%	0.1%
Srisuphanunt et al., 2008	23	143	0.16	[0.10; 0.23]	0.0%	0.1%
Stark et al., 2007	14	628	0.02	[0.01; 0.04]	0.3%	0.1%
Stensvold et al., 2011	1	96	0.01	[0.00; 0.06]	0.1%	0.1%
Sucilathangam et al., 2012	48	103	0.47	[0.37; 0.57]	0.0%	0.1%
Suryawanshi et al., 2012	15	100	0.15	[0.09; 0.24]	0.0%	0.1%
Taherkhani et al., 2007	20	75	0.27	[0.17; 0.38]	0.0%	0.1%
Tegegne et al., 2017	7	223	0.03	[0.01; 0.06]	0.1%	0.1%
Tellevik et al., 2015	8	33	0.24	[0.11; 0.42]	0.0%	0.1%
Tian et al., 2012	25	302	0.08	[0.05; 0.12]	0.0%	0.1%
Tian et al., 2013	8	79	0.10	[0.04; 0.19]	0.0%	0.1%
Tiwari et al. 2013	23	745	0.03	[0.02; 0.05]	0.3%	0.1%
Tuli et al., 2008	146	366	0.40	[0.35; 0.45]	0.0%	0.1%
Tumwine et al., 2005	67	91	0.74	[0.63; 0.82]	0.0%	0.1%
Uysal et al., 2017	3	115	0.03	[0.01; 0.07]	0.0%	0.1%
Vouking et al., 2014	15	207	0.07	[0.04; 0.12]	0.0%	0.1%
Vyas et al., 2013	11	75	0.15	[0.08; 0.25]	0.0%	0.1%
Wang et al., 2013	11	683	0.02	[0.01; 0.03]	0.5%	0.1%
Wanyiri et al., 2013	17	164	0.10	[0.06; 0.16]	0.0%	0.1%
Wanyiri et al., 2014	56	164	0.34	[0.27; 0.42]	0.0%	0.1%
Wumba et al., 2010	17	175	0.10	[0.06; 0.15]	0.0%	0.1%
Yosefi et al., 2012	8	60	0.13	[0.06; 0.25]	0.0%	0.1%
Zeynudin et al., 2013	8	91	0.09	[0.04; 0.17]	0.0%	0.1%
Gebrecherkos et al., 2019	10	150	0.07	[0.03; 0.12]	0.0%	0.1%
Sannella et al., 2019	104	155	0.67	[0.59; 0.74]	0.0%	0.1%
Abange et al., 2020	7	119	0.06	[0.02; 0.12]	0.0%	0.1%
Ayele et al., 2020	15	180	0.08	[0.05; 0.13]	0.0%	0.1%
Belay et al., 2020	9	112	0.08	[0.04; 0.15]	0.0%	0.1%
Chhangte et al., 2020	1	250	0.00	[0.00; 0.02]	0.5%	0.1%
Lengongo et al., 2020	1	115	0.01	[0.00; 0.05]	0.1%	0.1%
Namaji et al., 2020	89	361	0.25	[0.20; 0.29]	0.0%	0.1%
Purbey., 2021	2	75	0.03	[0.00; 0.09]	0.0%	0.1%
Sepahvand et al., 2022	7	26	0.27	[0.12; 0.48]	0.0%	0.1%
Uzairue et al., 2021	25	170	0.15	[0.10; 0.21]	0.0%	0.1%
Yulfi et al., 2021	13	54	0.24	[0.13; 0.38]	0.0%	0.1%
Weinreich et al., 2021	86	967	0.09	[0.07; 0.11]	0.1%	0.1%
Ajayi et al., 2021	19	375	0.05	[0.03; 0.08]	0.1%	0.1%
Bayleyegn et al., 2021	2	255	0.01	[0.00; 0.03]	0.3%	0.1%
Betancourth et al., 2021	4	102	0.04	[0.01; 0.10]	0.0%	0.1%
Botero et al., 2021	1	192	0.01	[0.00; 0.03]	0.3%	0.1%
Chandi et al., 2021	5	80	0.06	[0.02; 0.14]	0.0%	0.1%
Dankwa et al., 2021	26	418	0.06	[0.04; 0.09]	0.1%	0.1%
Feleke et al., 2022	4	223	0.02	[0.00; 0.05]	0.1%	0.1%
Wumba et al., 2012	13	121	0.11	[0.06; 0.18]	0.0%	0.1%
Common effect model		31155	0.03	[0.03; 0.03]	14.9%	--
Random effects model			0.14	[0.12; 0.17]	--	19.9%
Heterogeneity: $I^2 = 98\%$, $\tau^2 = 0.0280$, $p = 0$						
**Protozoa_Genus = Isospora**						
Abilo and Afework, 2005	1	70	0.01	[0.00; 0.08]	0.0%	0.1%
Adamu and Petros, 2009	10	200	0.05	[0.02; 0.09]	0.0%	0.1%
Agholi et al., 2013	2	356	0.01	[0.00; 0.02]	0.6%	0.1%
Ahmed and Abhay, 2015	22	142	0.15	[0.10; 0.23]	0.0%	0.1%
Akinbo et al., 2011	16	463	0.03	[0.02; 0.06]	0.2%	0.1%
Akinbo et al., 2012	21	2000	0.01	[0.01; 0.02]	2.2%	0.1%
Archana et al., 2017	17	104	0.16	[0.10; 0.25]	0.0%	0.1%
Asma et al., 2011	35	346	0.10	[0.07; 0.14]	0.0%	0.1%
Assefa et al., 2009	26	214	0.12	[0.08; 0.17]	0.0%	0.1%
Assefa et al., 2013	1	272	0.00	[0.00; 0.02]	0.6%	0.1%
Babatunde et al., 2010	10	90	0.11	[0.05; 0.19]	0.0%	0.1%
Cardoso et al., 2011	6	500	0.01	[0.00; 0.03]	0.5%	0.1%
da Silva et al., 2005	7	100	0.07	[0.03; 0.14]	0.0%	0.1%
Dash et al., 2013	19	115	0.17	[0.10; 0.25]	0.0%	0.1%
Djieyep et al., 2014	95	305	0.31	[0.26; 0.37]	0.0%	0.1%
Echoru et al., 2015	2	111	0.02	[0.00; 0.06]	0.1%	0.1%
Gaurav and Atul, 2015	4	40	0.10	[0.03; 0.24]	0.0%	0.1%
Gebretsadik et al., 2018	1	223	0.00	[0.00; 0.02]	0.4%	0.1%
Getaneh et al., 2018	3	220	0.01	[0.00; 0.04]	0.2%	0.1%
Girma et al., 2014	4	268	0.01	[0.00; 0.04]	0.2%	0.1%
Guk et al., 2005	5	105	0.05	[0.02; 0.11]	0.0%	0.1%
Gupta et al., 2013	10	100	0.10	[0.05; 0.18]	0.0%	0.1%
Haileeyesus et al., 2013	6	378	0.02	[0.01; 0.03]	0.3%	0.1%
Jaiswal et al., 2014	3	103	0.03	[0.01; 0.08]	0.0%	0.1%
Jayalakshmi et al., 2008	4	89	0.04	[0.01; 0.11]	0.0%	0.1%
Kammili et al., 2008	28	175	0.16	[0.11; 0.22]	0.0%	0.1%
Kiros et al., 2015	5	399	0.01	[0.00; 0.03]	0.3%	0.1%
Kulkarni et al., 2009	11	137	0.08	[0.04; 0.14]	0.0%	0.1%
Kurniawan et al., 2009	2	318	0.01	[0.00; 0.02]	0.5%	0.1%
Lehman et al., 2013	1	201	0.00	[0.00; 0.03]	0.3%	0.1%
Mariam et al., 2008	2	109	0.02	[0.00; 0.06]	0.1%	0.1%
Mathur et al., 2013	42	544	0.08	[0.06; 0.10]	0.1%	0.1%
Meamar et al., 2007	2	781	0.00	[0.00; 0.01]	2.9%	0.1%
Mehta et al., 2013	18	100	0.18	[0.11; 0.27]	0.0%	0.1%
Mekonnen et al., 2014	4	268	0.01	[0.00; 0.04]	0.2%	0.1%
Missaye et al., 2013	1	272	0.00	[0.00; 0.02]	0.6%	0.1%
Mitra et al., 2016	28	194	0.14	[0.10; 0.20]	0.0%	0.1%
Mohan et al., 2014	11	150	0.07	[0.04; 0.13]	0.0%	0.1%
Moran et al., 2005	1	203	0.00	[0.00; 0.03]	0.3%	0.1%
Navarro et al., 2006	2	103	0.02	[0.00; 0.07]	0.1%	0.1%
Nsagha et al., 2016	13	300	0.04	[0.02; 0.07]	0.1%	0.1%
Oguntibeju 2006	2	60	0.03	[0.00; 0.12]	0.0%	0.1%
Patel et al., 2011	3	100	0.03	[0.01; 0.09]	0.0%	0.1%
Pavie et al., 2012	1	143	0.01	[0.00; 0.04]	0.2%	0.1%
Pennap et al., 2015	7	200	0.04	[0.01; 0.07]	0.1%	0.1%
Pinlaor et al., 2005	1	78	0.01	[0.00; 0.07]	0.0%	0.1%
Ramakrishnan et al., 2007	1	80	0.01	[0.00; 0.07]	0.1%	0.1%
Ramana et al., 2010	46	452	0.10	[0.08; 0.13]	0.1%	0.1%
Rodrigues et al., 2008	24	582	0.04	[0.03; 0.06]	0.2%	0.1%
Saksirisampant et al., 2009	1	90	0.01	[0.00; 0.06]	0.1%	0.1%
Sanyaolu et al., 2011	2	65	0.03	[0.00; 0.11]	0.0%	0.1%
Sarfati et al., 2006	3	154	0.02	[0.00; 0.06]	0.1%	0.1%
Sherchan et al., 2012	3	146	0.02	[0.00; 0.06]	0.1%	0.1%
Shimelis et al., 2016	11	491	0.02	[0.01; 0.04]	0.3%	0.1%
Silva et al., 2005	7	100	0.07	[0.03; 0.14]	0.0%	0.1%
Sintayehu et al., 2013	2	343	0.01	[0.00; 0.02]	0.6%	0.1%
Sonali et al., 2014	2	250	0.01	[0.00; 0.03]	0.3%	0.1%
Stensvold et al., 2011	1	96	0.01	[0.00; 0.06]	0.1%	0.1%
Suryawanshi et al., 2012	13	100	0.13	[0.07; 0.21]	0.0%	0.1%
Tuli et al., 2008	2	366	0.01	[0.00; 0.02]	0.6%	0.1%
Vouking et al., 2014	12	207	0.06	[0.03; 0.10]	0.0%	0.1%
Vyas et al., 2013	9	75	0.12	[0.06; 0.22]	0.0%	0.1%
Wanyiri et al., 2013	18	164	0.11	[0.07; 0.17]	0.0%	0.1%
Wumba et al., 2010	3	175	0.02	[0.00; 0.05]	0.1%	0.1%
Zeynudin et al., 2013	6	91	0.07	[0.02; 0.14]	0.0%	0.1%
Gebrecherkos et al., 2019	3	150	0.02	[0.00; 0.06]	0.1%	0.1%
Sannella et al., 2019	4	155	0.03	[0.01; 0.06]	0.1%	0.1%
Lengongo et al., 2020	1	115	0.01	[0.00; 0.05]	0.1%	0.1%
Ajayi et al., 2021	5	375	0.01	[0.00; 0.03]	0.3%	0.1%
Feleke et al., 2022	2	223	0.01	[0.00; 0.03]	0.2%	0.1%
Common effect model		16794	0.01	[0.01; 0.01]	14.6%	--
Random effects model			0.05	[0.03; 0.06]	--	10.3%
Heterogeneity: $I^2 = 88\%$, $\tau^2 = 0.0024$, $p < 0.01$						
**Protozoa_Genus = Blastocystis**						
Adamu and Petros, 2009	3	200	0.01	[0.00; 0.04]	0.1%	0.1%
Agholi et al., 2013	14	356	0.04	[0.02; 0.07]	0.1%	0.1%
Barcelos et al., 2018	2	90	0.02	[0.00; 0.08]	0.0%	0.1%
Berenji et al., 2010	7	31	0.23	[0.10; 0.41]	0.0%	0.1%
Feyisayo et al., 2014	1	105	0.01	[0.00; 0.05]	0.1%	0.1%
Gupta et al., 2013	1	100	0.01	[0.00; 0.05]	0.1%	0.1%
Haileeyesus et al., 2013	24	378	0.06	[0.04; 0.09]	0.1%	0.1%
Jegede et al., 2014	1	105	0.01	[0.00; 0.05]	0.1%	0.1%
Kurniawan et al., 2009	234	318	0.74	[0.68; 0.78]	0.0%	0.1%
Mariam et al., 2008	2	109	0.02	[0.00; 0.06]	0.1%	0.1%
Meamar et al., 2007	48	781	0.06	[0.05; 0.08]	0.2%	0.1%
Mohan et al., 2014	1	150	0.01	[0.00; 0.04]	0.2%	0.1%
Moran et al., 2005	4	203	0.02	[0.01; 0.05]	0.1%	0.1%
Mtapuri et al., 2014	1	29	0.03	[0.00; 0.18]	0.0%	0.1%
Nkenfou et al., 2017	30	68	0.44	[0.32; 0.57]	0.0%	0.1%
Nsagha et al., 2016	75	300	0.25	[0.20; 0.30]	0.0%	0.1%
Oguntibeju 2006	2	60	0.03	[0.00; 0.12]	0.0%	0.1%
Paboriboune et al., 2014	36	137	0.26	[0.19; 0.34]	0.0%	0.1%
Pennap et al., 2015	11	200	0.06	[0.03; 0.10]	0.0%	0.1%
Pinlaor et al., 2005	1	78	0.01	[0.00; 0.07]	0.0%	0.1%
Ramana et al., 2010	139	452	0.31	[0.27; 0.35]	0.0%	0.1%
Roka et al., 2012	28	260	0.11	[0.07; 0.15]	0.0%	0.1%
Roka et al., 2013	16	273	0.06	[0.03; 0.09]	0.1%	0.1%
Saksirisampant et al., 2009	2	90	0.02	[0.00; 0.08]	0.0%	0.1%
Samie et al., 2014	3	151	0.02	[0.00; 0.06]	0.1%	0.1%
Sherchan et al., 2012	9	146	0.06	[0.03; 0.11]	0.0%	0.1%
Stark et al., 2007	111	628	0.18	[0.15; 0.21]	0.0%	0.1%
Stensvold et al., 2011	22	96	0.23	[0.15; 0.33]	0.0%	0.1%
Tamirat et al., 2017	14	226	0.06	[0.03; 0.10]	0.0%	0.1%
Tian et al., 2012	49	302	0.16	[0.12; 0.21]	0.0%	0.1%
Tian et al., 2013	11	79	0.14	[0.07; 0.24]	0.0%	0.1%
Tiwari et al. 2013	2	745	0.00	[0.00; 0.01]	2.6%	0.1%
Yosefi et al., 2012	2	60	0.03	[0.00; 0.12]	0.0%	0.1%
Masoumi et al., 2019	2	102	0.02	[0.00; 0.07]	0.1%	0.1%
Abange et al., 2020	3	119	0.03	[0.01; 0.07]	0.0%	0.1%
Chhangte et al., 2020	17	250	0.07	[0.04; 0.11]	0.0%	0.1%
Lengongo et al., 2020	26	115	0.23	[0.15; 0.31]	0.0%	0.1%
Yulfi et al., 2021	5	54	0.09	[0.03; 0.20]	0.0%	0.1%
Betancourth et al., 2021	11	102	0.11	[0.06; 0.18]	0.0%	0.1%
Botero et al., 2021	23	192	0.12	[0.08; 0.17]	0.0%	0.1%
Common effect model		8240	0.02	[0.02; 0.02]	4.5%	--
Random effects model			0.11	[0.06; 0.15]	--	5.8%
Heterogeneity: $I^2 = 98\%$, $\tau^2 = 0.0191$, $p < 0.01$						
**Protozoa_Genus = Plasmodium**						
Adu-Gyasi et al., 2013	15	933	0.02	[0.01; 0.03]	0.7%	0.1%
Akinbo et al., 2016	32	411	0.08	[0.05; 0.11]	0.1%	0.1%
Akinbo et al., 2012	87	2000	0.04	[0.03; 0.05]	0.6%	0.1%
Ako-Nai et al., 2013	34	44	0.77	[0.62; 0.89]	0.0%	0.1%
Amuta et al., 2012	343	1080	0.32	[0.29; 0.35]	0.1%	0.1%
Bate et al., 2016	58	234	0.25	[0.19; 0.31]	0.0%	0.1%
Bharti et al., 2012	41	460	0.09	[0.06; 0.12]	0.1%	0.1%
Chalwe et al., 2009	42	55	0.76	[0.63; 0.87]	0.0%	0.1%
Chukwuanukwu et al., 2016	110	239	0.46	[0.40; 0.53]	0.0%	0.1%
Cohen et al., 2005	18	110	0.16	[0.10; 0.25]	0.0%	0.1%
Desta et al., 2005	8	49	0.16	[0.07; 0.30]	0.0%	0.1%
Franke et al., 2010	199	1066	0.19	[0.16; 0.21]	0.1%	0.1%
Frosch et al., 2017	11	138	0.08	[0.04; 0.14]	0.0%	0.1%
Gallagher et al., 2005	34	83	0.41	[0.30; 0.52]	0.0%	0.1%
Goselle et al., 2007	64	200	0.32	[0.26; 0.39]	0.0%	0.1%
Hasang et al., 2014	100	216	0.46	[0.40; 0.53]	0.0%	0.1%
Idindili et al., 2011	105	421	0.25	[0.21; 0.29]	0.0%	0.1%
Igbeneghu et al., 2012	18	38	0.47	[0.31; 0.64]	0.0%	0.1%
Iroezindu et al., 2012	24	97	0.25	[0.17; 0.35]	0.0%	0.1%
Iroezindu et al., 2012	21	63	0.33	[0.22; 0.46]	0.0%	0.1%
Izuka et al., 2017	70	102	0.69	[0.59; 0.77]	0.0%	0.1%
Johnbull et al., 2014	149	301	0.50	[0.44; 0.55]	0.0%	0.1%
Kapito et al., 2011	118	1142	0.10	[0.09; 0.12]	0.1%	0.1%
Kawai et al., 2009	177	971	0.18	[0.16; 0.21]	0.1%	0.1%
Kfutwah et al., 2009	14	80	0.17	[0.10; 0.28]	0.0%	0.1%
Kiying et al., 2010	120	160	0.75	[0.68; 0.81]	0.0%	0.1%
Manyanga et al., 2014	19	420	0.05	[0.03; 0.07]	0.1%	0.1%
Marete et al., 2014	201	245	0.82	[0.77; 0.87]	0.0%	0.1%
Mohapatra et al., 2017	23	333	0.07	[0.04; 0.10]	0.1%	0.1%
Morona et al., 2018	56	250	0.22	[0.17; 0.28]	0.0%	0.1%
Njunda et al., 2016	26	355	0.07	[0.05; 0.11]	0.1%	0.1%
Nkhoma et al., 2012	70	185	0.38	[0.31; 0.45]	0.0%	0.1%
Okogun et al., 2010	5	16	0.31	[0.11; 0.59]	0.0%	0.1%
Okonkwo et al., 2018	61	179	0.34	[0.27; 0.42]	0.0%	0.1%
Olaniran et al., 2012	83	93	0.89	[0.81; 0.95]	0.0%	0.1%
Omoti et al., 2013	254	342	0.74	[0.69; 0.79]	0.0%	0.1%
Onyenekwe et al., 2007	28	149	0.19	[0.13; 0.26]	0.0%	0.1%
Osuji et al., 2012	74	134	0.55	[0.46; 0.64]	0.0%	0.1%
Quigley et al., 2005	154	222	0.69	[0.63; 0.75]	0.0%	0.1%
Sanyaolu et al., 2013	35	65	0.54	[0.41; 0.66]	0.0%	0.1%
Saracino et al., 2012	29	220	0.13	[0.09; 0.18]	0.0%	0.1%
Tagoe and Boachie, 2012	34	220	0.15	[0.11; 0.21]	0.0%	0.1%
Tay et al., 2015	47	400	0.12	[0.09; 0.15]	0.0%	0.1%
Tegenaw et al., 2013	2	23	0.09	[0.01; 0.28]	0.0%	0.1%
Uneke et al., 2005	103	490	0.21	[0.17; 0.25]	0.0%	0.1%
Wumba et al., 2015	19	25	0.76	[0.55; 0.91]	0.0%	0.1%
Zheng et al., 2017	14	101	0.14	[0.08; 0.22]	0.0%	0.1%
Chukwuocha et al., 2019	398	398	1.00	[0.99; 1.00]	3.7%	0.1%
Amodu et al., 2020	100	140	0.71	[0.63; 0.79]	0.0%	0.1%
Anyanwu et al., 2020	142	420	0.34	[0.29; 0.39]	0.0%	0.1%
Jegede et al., 2020	386	1521	0.25	[0.23; 0.28]	0.1%	0.1%
Rabiu et al., 2020	49	197	0.25	[0.19; 0.32]	0.0%	0.1%
Sandie et al., 2021	31	190	0.16	[0.11; 0.22]	0.0%	0.1%
Stiffler et al., 2021	44	129	0.34	[0.26; 0.43]	0.0%	0.1%
Alonso et al., 2021	26	144	0.18	[0.12; 0.25]	0.0%	0.1%
Jemikalajah et al., 2021	228	300	0.76	[0.71; 0.81]	0.0%	0.1%
Common effect model		18599	0.64	[0.64; 0.65]	6.3%	--
Random effects model			0.35	[0.28; 0.42]	--	8.0%
Heterogeneity: $I^2 = 100\%$, $\tau^2 = 0.0673$, $p = 0$						
**Protozoa_Genus = Cyclospora**						
Agholi et al., 2013	1	356	0.00	[0.00; 0.02]	1.0%	0.1%
Ahmed and Abhay, 2015	3	142	0.02	[0.00; 0.06]	0.1%	0.1%
Asma et al., 2011	17	346	0.05	[0.03; 0.08]	0.1%	0.1%
Babatunde et al., 2010	16	90	0.18	[0.11; 0.27]	0.0%	0.1%
Dash et al., 2013	1	115	0.01	[0.00; 0.05]	0.1%	0.1%
Eshetu et al., 2017	6	223	0.03	[0.01; 0.06]	0.1%	0.1%
Getaneh et al., 2018	13	220	0.06	[0.03; 0.10]	0.0%	0.1%
Jaiswal et al., 2014	2	103	0.02	[0.00; 0.07]	0.1%	0.1%
Kammili et al., 2008	2	175	0.01	[0.00; 0.04]	0.1%	0.1%
Kulkarni et al., 2009	1	137	0.01	[0.00; 0.04]	0.2%	0.1%
Kurniawan et al., 2009	21	318	0.07	[0.04; 0.10]	0.1%	0.1%
Lehman et al., 2013	1	201	0.00	[0.00; 0.03]	0.3%	0.1%
Mathur et al., 2013	12	544	0.02	[0.01; 0.04]	0.3%	0.1%
Mitra et al., 2016	2	194	0.01	[0.00; 0.04]	0.2%	0.1%
Mohan et al., 2014	1	150	0.01	[0.00; 0.04]	0.2%	0.1%
Mtapuri et al., 2014	1	29	0.03	[0.00; 0.18]	0.0%	0.1%
Nkenfou et al., 2017	2	68	0.03	[0.00; 0.10]	0.0%	0.1%
Nsagha et al., 2016	11	300	0.04	[0.02; 0.06]	0.1%	0.1%
Paboriboune et al., 2014	3	137	0.02	[0.00; 0.06]	0.1%	0.1%
Pennap et al., 2015	15	200	0.07	[0.04; 0.12]	0.0%	0.1%
Pinlaor et al., 2005	1	78	0.01	[0.00; 0.07]	0.0%	0.1%
Ramana et al., 2010	27	452	0.06	[0.04; 0.09]	0.1%	0.1%
Saksirisampant et al., 2009	1	90	0.01	[0.00; 0.06]	0.1%	0.1%
Sherchan et al., 2012	6	146	0.04	[0.02; 0.09]	0.0%	0.1%
Sherpa et al., 2010	4	106	0.04	[0.01; 0.09]	0.0%	0.1%
Shimelis et al., 2016	2	491	0.00	[0.00; 0.01]	1.1%	0.1%
Sucilathangam et al., 2012	6	103	0.06	[0.02; 0.12]	0.0%	0.1%
Tegegne et al., 2017	6	223	0.03	[0.01; 0.06]	0.1%	0.1%
Tiwari et al. 2013	14	745	0.02	[0.01; 0.03]	0.5%	0.1%
Tuli et al., 2008	88	366	0.24	[0.20; 0.29]	0.0%	0.1%
Uysal et al., 2017	3	115	0.03	[0.01; 0.07]	0.0%	0.1%
Wanyiri et al., 2013	5	164	0.03	[0.01; 0.07]	0.1%	0.1%
Masoumi et al., 2019	1	102	0.01	[0.00; 0.05]	0.1%	0.1%
Belay et al., 2020	1	112	0.01	[0.00; 0.05]	0.1%	0.1%
Chhangte et al., 2020	1	250	0.00	[0.00; 0.02]	0.5%	0.1%
Namaji et al., 2020	7	361	0.02	[0.01; 0.04]	0.2%	0.1%
Ajayi et al., 2021	2	375	0.01	[0.00; 0.02]	0.7%	0.1%
Common effect model		8327	0.01	[0.01; 0.01]	6.7%	--
Random effects model			0.03	[0.02; 0.04]	--	5.5%
Heterogeneity: $I^2 = 85\%$, $\tau^2 = 0.0012$, $p < 0.01$						
**Protozoa_Genus = Leishmania**						
Almeida et al., 2012	5	10	0.50	[0.19; 0.81]	0.0%	0.1%
Carranza et al., 2009	26	163	0.16	[0.11; 0.22]	0.0%	0.1%
Cavalcanti et al., 2012	5	10	0.50	[0.19; 0.81]	0.0%	0.1%
Colomba et al., 2009	24	145	0.17	[0.11; 0.24]	0.0%	0.1%
Cota et al., 2013	41	113	0.36	[0.27; 0.46]	0.0%	0.1%
Garcia et al., 2006	28	92	0.30	[0.21; 0.41]	0.0%	0.1%
Júnior et al., 2015	7	15	0.47	[0.21; 0.73]	0.0%	0.1%
Oliveira et al., 2011	13	169	0.08	[0.04; 0.13]	0.0%	0.1%
Pandey et al., 2018	176	305	0.58	[0.52; 0.63]	0.0%	0.1%
Rezaei et al., 2018	19	251	0.08	[0.05; 0.12]	0.0%	0.1%
Shafiei et al., 2014	9	49	0.18	[0.09; 0.32]	0.0%	0.1%
Abdullahi et al., 2020	28	58	0.48	[0.35; 0.62]	0.0%	0.1%
Barbosa et al., 2020	65	113	0.58	[0.48; 0.67]	0.0%	0.1%
Barbosa et al., 2020	110	309	0.36	[0.30; 0.41]	0.0%	0.1%
Charoensakulchai et al., 2020	110	526	0.21	[0.18; 0.25]	0.0%	0.1%
Cunha et al., 2020	36	240	0.15	[0.11; 0.20]	0.0%	0.1%
Common effect model		2568	0.21	[0.20; 0.23]	0.2%	--
Random effects model			0.30	[0.22; 0.39]	--	2.2%
Heterogeneity: $I^2 = 96\%$, $\tau^2 = 0.0287$, $p < 0.01$						
**Protozoa_Genus = Trypanosoma**						
Almeida et al., 2010	9	716	0.01	[0.01; 0.02]	0.6%	0.1%
Llenas et al., 2012	4	155	0.03	[0.01; 0.06]	0.1%	0.1%
Rodrigues et al., 2011	2	19	0.11	[0.01; 0.33]	0.0%	0.1%
Rodrigues et al., 2005	18	28	0.64	[0.44; 0.81]	0.0%	0.1%
McAtee et al., 2021	32	116	0.28	[0.20; 0.37]	0.0%	0.1%
Common effect model		1034	0.02	[0.01; 0.03]	0.7%	--
Random effects model			0.20	[0.00; 0.42]	--	0.7%
Heterogeneity: $I^2 = 96\%$, $\tau^2 = 0.0604$, $p < 0.01$						
**Protozoa_Genus = Trichomonas**						
Assefa et al., 2009	2	214	0.01	[0.00; 0.03]	0.2%	0.1%
Davis et al., 2016	16	44	0.36	[0.22; 0.52]	0.0%	0.1%
De Lemos and Garcia-Zapata, 2010	23	125	0.18	[0.12; 0.26]	0.0%	0.1%
Gupta et al., 2013	2	100	0.02	[0.00; 0.07]	0.0%	0.1%
Kissinger et al., 2008	11	60	0.18	[0.10; 0.30]	0.0%	0.1%
Lemos et al., 2009	23	125	0.18	[0.12; 0.26]	0.0%	0.1%
Masese et al., 2011	31	147	0.21	[0.15; 0.29]	0.0%	0.1%
Mtapuri et al., 2014	1	29	0.03	[0.00; 0.18]	0.0%	0.1%
Nangammbi et al., 2013	32	155	0.21	[0.15; 0.28]	0.0%	0.1%
Nwadioha et al., 2012	84	350	0.24	[0.20; 0.29]	0.0%	0.1%
Nweze and Mouneke, 2011	355	970	0.37	[0.34; 0.40]	0.0%	0.1%
Price et al., 2018	76	359	0.21	[0.17; 0.26]	0.0%	0.1%
Stark et al., 2007	2	628	0.00	[0.00; 0.01]	1.9%	0.1%
Abange et al., 2020	1	119	0.01	[0.00; 0.05]	0.1%	0.1%
Medina et al., 2020	86	427	0.20	[0.16; 0.24]	0.0%	0.1%
Betancourth et al., 2021	1	102	0.01	[0.00; 0.05]	0.1%	0.1%
Common effect model		3954	0.02	[0.01; 0.02]	2.5%	--
Random effects model			0.15	[0.09; 0.21]	--	2.3%
Heterogeneity: $I^2 = 98\%$, $\tau^2 = 0.0141$, $p < 0.01$						
**Protozoa_Genus = Iodamoeba**						
Babatunde et al., 2010	7	90	0.08	[0.03; 0.15]	0.0%	0.1%
Barcelos et al., 2018	1	90	0.01	[0.00; 0.06]	0.1%	0.1%
Gupta et al., 2013	1	100	0.01	[0.00; 0.05]	0.1%	0.1%
Moran et al., 2005	3	203	0.01	[0.00; 0.04]	0.1%	0.1%
Mtapuri et al., 2014	1	29	0.03	[0.00; 0.18]	0.0%	0.1%
Ngum et al., 2015	7	320	0.02	[0.01; 0.04]	0.2%	0.1%
Nkenfou et al., 2017	3	68	0.04	[0.01; 0.12]	0.0%	0.1%
Patel et al., 2011	1	100	0.01	[0.00; 0.05]	0.1%	0.1%
Rodrigues et al., 2008	37	582	0.06	[0.05; 0.09]	0.1%	0.1%
Roka et al., 2012	1	260	0.00	[0.00; 0.02]	0.5%	0.1%
Roka et al., 2013	16	273	0.06	[0.03; 0.09]	0.1%	0.1%
Sanyaolu et al., 2011	2	65	0.03	[0.00; 0.11]	0.0%	0.1%
Stark et al., 2007	8	628	0.01	[0.01; 0.02]	0.6%	0.1%
Wumba et al., 2012	1	242	0.00	[0.00; 0.02]	0.5%	0.1%
Abange et al., 2020	2	119	0.02	[0.00; 0.06]	0.1%	0.1%
Lengongo et al., 2020	1	115	0.01	[0.00; 0.05]	0.1%	0.1%
Betancourth et al., 2021	1	102	0.01	[0.00; 0.05]	0.1%	0.1%
Common effect model		3386	0.01	[0.01; 0.02]	2.6%	--
Random effects model			0.02	[0.01; 0.03]	--	2.5%
Heterogeneity: $I^2 = 71\%$, $\tau^2 = 0.0002$, $p < 0.01$						
**Protozoa_Genus = Endolimax**						
Barcelos et al., 2018	15	90	0.17	[0.10; 0.26]	0.0%	0.1%
Cardoso et al., 2011	3	500	0.01	[0.00; 0.02]	0.8%	0.1%
Gupta et al., 2013	2	100	0.02	[0.00; 0.07]	0.0%	0.1%
Moran et al., 2005	13	203	0.06	[0.03; 0.11]	0.0%	0.1%
Mtapuri et al., 2014	1	29	0.03	[0.00; 0.18]	0.0%	0.1%
Rodrigues et al., 2008	38	582	0.07	[0.05; 0.09]	0.1%	0.1%
Roka et al., 2012	13	260	0.05	[0.03; 0.08]	0.1%	0.1%
Roka et al., 2013	58	273	0.21	[0.17; 0.27]	0.0%	0.1%
Stark et al., 2007	62	628	0.10	[0.08; 0.12]	0.1%	0.1%
Tamirat et al., 2017	1	226	0.00	[0.00; 0.02]	0.4%	0.1%
Wumba et al., 2012	4	242	0.02	[0.00; 0.04]	0.2%	0.1%
Yosefi et al., 2012	5	60	0.08	[0.03; 0.18]	0.0%	0.1%
Abange et al., 2020	2	119	0.02	[0.00; 0.06]	0.1%	0.1%
Betancourth et al., 2021	7	102	0.07	[0.03; 0.14]	0.0%	0.1%
Common effect model		3414	0.02	[0.01; 0.02]	1.9%	--
Random effects model			0.06	[0.03; 0.09]	--	2.1%
Heterogeneity: $I^2 = 93\%$, $\tau^2 = 0.0030$, $p < 0.01$						
**Protozoa_Genus = Cystoisospora**						
Barcelos et al., 2018	1	90	0.01	[0.00; 0.06]	0.1%	0.1%
Ngum et al., 2015	6	320	0.02	[0.01; 0.04]	0.2%	0.1%
Paboriboune et al., 2014	6	137	0.04	[0.02; 0.09]	0.0%	0.1%
Gebrecherkos et al., 2019	2	150	0.01	[0.00; 0.05]	0.1%	0.1%
Chhangte et al., 2020	3	250	0.01	[0.00; 0.03]	0.2%	0.1%
Namaji et al., 2020	30	361	0.08	[0.06; 0.12]	0.1%	0.1%
Betancourth et al., 2021	1	102	0.01	[0.00; 0.05]	0.1%	0.1%
Botero et al., 2021	1	192	0.01	[0.00; 0.03]	0.3%	0.1%
Common effect model		1602	0.01	[0.01; 0.02]	1.0%	--
Random effects model			0.02	[0.01; 0.04]	--	1.2%
Heterogeneity: $I^2 = 76\%$, $\tau^2 = 0.0004$, $p < 0.01$						
**Protozoa_Genus = Chilomastix**						
Berenji et al., 2010	7	31	0.23	[0.10; 0.41]	0.0%	0.1%
Daryani et al., 2009	1	64	0.02	[0.00; 0.08]	0.0%	0.1%
Gupta et al., 2013	1	100	0.01	[0.00; 0.05]	0.1%	0.1%
Rodrigues et al., 2008	3	582	0.01	[0.00; 0.01]	1.1%	0.1%
Roka et al., 2012	11	260	0.04	[0.02; 0.07]	0.1%	0.1%
Roka et al., 2013	3	273	0.01	[0.00; 0.03]	0.3%	0.1%
Stark et al., 2007	3	628	0.00	[0.00; 0.01]	1.3%	0.1%
Lengongo et al., 2020	5	115	0.04	[0.01; 0.10]	0.0%	0.1%
Betancourth et al., 2021	1	102	0.01	[0.00; 0.05]	0.1%	0.1%
Common effect model		2155	0.01	[0.00; 0.01]	3.0%	--
Random effects model			0.01	[0.00; 0.02]	--	1.3%
Heterogeneity: $I^2 = 64\%$, $\tau^2 = < 0.0001$, $p < 0.01$						
**Common effect model**		**160249**	**0.06**	**[0.06; 0.06]**	**100.0%**	**--**
**Random effects model**			**0.15**	**[0.14; 0.17]**	**--**	**100.0%**

Heterogeneity: $I^2 = 100\%$, $\tau^2 = 0.0390$, $p = 0$

Test for subgroup differences (fixed effect): $\chi^2_{14} = 215717.20$, df = 14 ($p = 0$)

Test for subgroup differences (random effects): $\chi^2_{14} = 485.74$, df = 14 ($p < 0.01$)

0 0.2 0.4 0.6 0.8 1

Figure S5A: Subgroup analysis of the prevalence of helminth infections in people living with viruses by age

Study	Events	Total	Proportion	95%-CI	Weight (common)	Weight (random)
**Age = <35**						
Abaver et al., 2011	9	85	0.11	[0.05; 0.19]	0.1%	0.8%
Adeleke et al., 2015	57	231	0.25	[0.19; 0.31]	0.2%	0.8%
Adesij et al. 2007	12	100	0.12	[0.06; 0.20]	0.1%	0.8%
Assefa et al., 2009	81	214	0.38	[0.31; 0.45]	0.1%	0.8%
Assefa et al., 2013	11	272	0.04	[0.02; 0.07]	1.1%	0.8%
Erikstrup et al., 2008	190	198	0.96	[0.92; 0.98]	0.8%	0.8%
Eshetu et al., 2017	36	223	0.16	[0.12; 0.22]	0.3%	0.8%
Feyisayo et al., 2014	4	105	0.04	[0.01; 0.09]	0.4%	0.8%
Gupta et al., 2013	3	100	0.03	[0.01; 0.09]	0.5%	0.8%
Haileeyesus et al., 2013	59	378	0.16	[0.12; 0.20]	0.4%	0.8%
Jegede et al., 2014	4	105	0.04	[0.01; 0.09]	0.4%	0.8%
Kulkarni et al., 2009	9	137	0.07	[0.03; 0.12]	0.3%	0.8%
Kallestrup et al., 2005	185	407	0.45	[0.41; 0.50]	0.3%	0.8%
Kawai et al., 2009	178	971	0.18	[0.16; 0.21]	1.0%	0.8%
Kipyegen et al., 2012	29	285	0.10	[0.07; 0.14]	0.5%	0.8%
Kroidl et al., 2016	69	187	0.37	[0.30; 0.44]	0.1%	0.8%
Kurniawan et al., 2009	3	318	0.01	[0.00; 0.03]	5.2%	0.8%
Lar et al., 2016	77	205	0.38	[0.31; 0.45]	0.1%	0.8%
Marti et al., 2017	60	305	0.20	[0.15; 0.25]	0.3%	0.8%
Mathur et al., 2013	34	544	0.06	[0.04; 0.09]	1.4%	0.8%
Mazigo et al., 2016	28	50	0.56	[0.41; 0.70]	0.0%	0.7%
Missaye et al., 2013	19	272	0.07	[0.04; 0.11]	0.6%	0.8%
Modjarrad et al., 2005	74	297	0.25	[0.20; 0.30]	0.2%	0.8%
Modjarrad et al., 2005	54	111	0.49	[0.39; 0.58]	0.1%	0.8%
Ndhlovu et al., 2007	72	156	0.46	[0.38; 0.54]	0.1%	0.8%
Ojurongbe et al., 2011	16	96	0.17	[0.10; 0.26]	0.1%	0.8%
Orji et al., 2017	24	84	0.29	[0.19; 0.39]	0.1%	0.8%
Oyedeji et al., 2015	1	52	0.02	[0.00; 0.10]	0.4%	0.8%
Pennap et al., 2015	103	200	0.52	[0.44; 0.59]	0.1%	0.8%
Raytekar et al., 2012	2	127	0.02	[0.00; 0.06]	1.3%	0.8%
Sherpa et al., 2010	4	106	0.04	[0.01; 0.09]	0.4%	0.8%
Shimelis et al., 2016	54	491	0.11	[0.08; 0.14]	0.8%	0.8%
Suryawanshi et al., 2012	12	100	0.12	[0.06; 0.20]	0.1%	0.8%
Talaat et al., 2015	28	80	0.35	[0.25; 0.46]	0.1%	0.8%
Tegegne et al., 2017	36	223	0.16	[0.12; 0.22]	0.3%	0.8%
Tigist et al., 2015	57	209	0.27	[0.21; 0.34]	0.2%	0.8%
Tiwari et al., 2013	83	745	0.11	[0.09; 0.14]	1.2%	0.8%
Vyas et al., 2013	2	75	0.03	[0.00; 0.09]	0.4%	0.8%
Zeynudin et al., 2013	29	91	0.32	[0.22; 0.42]	0.1%	0.8%
Gebrecherkos et al., 2019	26	150	0.17	[0.12; 0.24]	0.2%	0.8%
Sannella et al., 2019	23	155	0.15	[0.10; 0.21]	0.2%	0.8%
Rabiu et al., 2020	6	197	0.03	[0.01; 0.07]	1.0%	0.8%
**Common effect model**		9437	0.12	[0.11; 0.12]	21.8%	--
**Random effects model**			0.21	[0.15; 0.27]	--	34.2%
Heterogeneity: I^2 = 99%, τ^2 = 0.0373, p = 0						
**Age = ≥35**						
Abbas et al., 2009	55	99	0.56	[0.45; 0.66]	0.1%	0.8%
Adamu and Petros, 2009	9	200	0.04	[0.02; 0.08]	0.7%	0.8%
Agholi et al., 2013	4	356	0.01	[0.00; 0.03]	4.9%	0.8%
Ahmed and Abhay, 2015	14	142	0.10	[0.05; 0.16]	0.2%	0.8%
Akinbo and Omoregie, 2011	15	285	0.05	[0.03; 0.09]	0.9%	0.8%
Akinbo et al., 2012	156	2000	0.08	[0.07; 0.09]	4.3%	0.8%
Allam et al., 2014	68	141	0.48	[0.40; 0.57]	0.1%	0.8%
Asma et al., 2011	66	346	0.19	[0.15; 0.24]	0.3%	0.8%
Babatunde et al., 2010	39	90	0.43	[0.33; 0.54]	0.1%	0.8%
Barcelos et al., 2018	1	90	0.01	[0.00; 0.06]	1.3%	0.8%
Cardoso et al., 2011	4	500	0.01	[0.00; 0.02]	9.7%	0.8%
Colombe et al., 2018	37	79	0.47	[0.36; 0.58]	0.0%	0.8%
Costiniuk et al., 2012	21	97	0.22	[0.14; 0.31]	0.1%	0.8%
Da Silva et al., 2005	19	100	0.19	[0.12; 0.28]	0.1%	0.8%
Dash et al., 2013	6	115	0.05	[0.02; 0.11]	0.4%	0.8%
Fekadu et al., 2013	118	343	0.34	[0.29; 0.40]	0.2%	0.8%
Guk et al., 2005	3	105	0.03	[0.01; 0.08]	0.6%	0.8%
Hochberg et al., 2011	48	128	0.38	[0.29; 0.46]	0.1%	0.8%
Idindili et al., 2011	148	421	0.35	[0.31; 0.40]	0.3%	0.8%
Lehman et al., 2013	3	201	0.01	[0.00; 0.04]	2.1%	0.8%
Loffredo et al., 2015	16	31	0.52	[0.33; 0.70]	0.0%	0.7%
Mascarello et al., 2011	15	138	0.11	[0.06; 0.17]	0.2%	0.8%
Minti et al., 2017	1	27	0.04	[0.00; 0.19]	0.1%	0.8%
Mkhize et al., 2017	31	95	0.33	[0.23; 0.43]	0.1%	0.8%
Morawski et al., 2017	65	202	0.32	[0.26; 0.39]	0.1%	0.8%
Nielsen et al., 2007	18	34	0.53	[0.35; 0.70]	0.0%	0.7%
Nsagha et al., 2016	34	300	0.11	[0.08; 0.15]	0.5%	0.8%
Paboriboune et al., 2014	116	137	0.85	[0.78; 0.90]	0.2%	0.8%
Pavie et al., 2012	4	143	0.03	[0.01; 0.07]	0.8%	0.8%
Ringo et al., 2015	13	83	0.16	[0.09; 0.25]	0.1%	0.8%
Rodrigues et al., 2008	366	582	0.63	[0.59; 0.67]	0.4%	0.8%
Roka et al., 2012	242	260	0.93	[0.89; 0.96]	0.6%	0.8%
Roka et al., 2013	246	273	0.90	[0.86; 0.93]	0.5%	0.8%
Saksirisampant et al., 2009	2	90	0.02	[0.00; 0.08]	0.6%	0.8%
Sanyaolu et al., 2011	12	65	0.18	[0.10; 0.30]	0.1%	0.8%
Smith et al., 2008	21	122	0.17	[0.11; 0.25]	0.1%	0.8%
Tian et al., 2012	14	302	0.05	[0.03; 0.08]	1.0%	0.8%
Tian et al., 2013	5	79	0.06	[0.02; 0.14]	0.2%	0.8%
Vouking et al., 2014	46	207	0.22	[0.17; 0.29]	0.2%	0.8%
Wanyiri et al., 2013	42	164	0.26	[0.19; 0.33]	0.1%	0.8%
Wumba et al., 2010	12	175	0.07	[0.04; 0.12]	0.4%	0.8%
Wumba et al., 2012	6	242	0.02	[0.01; 0.05]	1.5%	0.8%
Xiao et al., 2015	22	40	0.55	[0.38; 0.71]	0.0%	0.7%
Kaneshiro et al., 2019	87	252	0.35	[0.29; 0.41]	0.2%	0.8%
Alemayehu etal., 2020	74	383	0.19	[0.15; 0.24]	0.4%	0.8%
Belay et al., 2020	4	112	0.04	[0.01; 0.09]	0.5%	0.8%
Lengongo et al., 2020	9	115	0.08	[0.04; 0.14]	0.2%	0.8%
Botero et al., 2021	2	192	0.01	[0.00; 0.04]	2.9%	0.8%
Betancourth et al., 2021	3	102	0.03	[0.01; 0.08]	0.5%	0.8%
Chandi et al., 2021	6	80	0.07	[0.03; 0.16]	0.2%	0.8%
Ajayi et al., 2021	10	375	0.03	[0.01; 0.05]	2.2%	0.8%
Talukder et al., 2021	45	201	0.22	[0.17; 0.29]	0.2%	0.8%
Wolday et al., 2021	184	751	0.25	[0.21; 0.28]	0.6%	0.8%
Purbey., 2021	13	75	0.17	[0.10; 0.28]	0.1%	0.8%
**Common effect model**		12267	0.08	[0.08; 0.08]	42.3%	--
**Random effects model**			0.23	[0.17; 0.29]	--	43.8%
Heterogeneity: I^2 = 99%, τ^2 = 0.0552, p = 0						
**Age = N/A**						
Abbas et al., 2009	74	119	0.62	[0.53; 0.71]	0.1%	0.8%
Abilo and Afework, 2005	18	70	0.26	[0.16; 0.38]	0.1%	0.8%
Abossie and Petros, 2015	55	97	0.57	[0.46; 0.67]	0.1%	0.8%
Adams et al., 2006	103	170	0.61	[0.53; 0.68]	0.1%	0.8%
Akinbo et al., 2011	125	463	0.27	[0.23; 0.31]	0.4%	0.8%
Gaurav and Atul, 2015	6	40	0.15	[0.06; 0.30]	0.0%	0.8%
Getaneh et al., 2018	15	220	0.07	[0.04; 0.11]	0.5%	0.8%
Gebretsadik et al., 2018	8	223	0.04	[0.02; 0.07]	1.0%	0.8%
Meamar et al., 2007	5	781	0.01	[0.00; 0.01]	18.8%	0.8%
Mehta et al., 2013	3	100	0.03	[0.01; 0.09]	0.5%	0.8%
Mtapuri et al., 2014	1	29	0.03	[0.00; 0.18]	0.1%	0.8%
Mulu et al., 2013	87	220	0.40	[0.33; 0.46]	0.1%	0.8%
Nuhu et al., 2014	8	205	0.04	[0.02; 0.08]	0.8%	0.8%
Pinlaor et al., 2005	37	78	0.47	[0.36; 0.59]	0.0%	0.8%
Plumelle et al., 2015	55	445	0.12	[0.09; 0.16]	0.6%	0.8%
Ramakrishnan et al., 2007	7	80	0.09	[0.04; 0.17]	0.2%	0.8%
Ramana et al., 2010	32	452	0.07	[0.05; 0.10]	1.1%	0.8%
Samie et al., 2014	62	151	0.41	[0.33; 0.49]	0.1%	0.8%
Sonali et al., 2014	2	250	0.01	[0.00; 0.03]	4.8%	0.8%
Talaat et al., 2008	40	632	0.06	[0.05; 0.09]	1.6%	0.8%
Chhangte et al., 2020	8	250	0.03	[0.01; 0.06]	1.2%	0.8%
McLellan et al., 2020	58	253	0.23	[0.18; 0.29]	0.2%	0.8%
Namaji et al., 2020	19	361	0.05	[0.03; 0.08]	1.1%	0.8%
Bayleyegn et al., 2021	32	255	0.13	[0.09; 0.17]	0.4%	0.8%
Feleke et al., 2022	23	223	0.10	[0.07; 0.15]	0.4%	0.8%
Uzairue et al., 2021	51	170	0.30	[0.23; 0.37]	0.1%	0.8%
PrayGod et al., 2022	176	1149	0.15	[0.13; 0.18]	1.4%	0.8%
**Common effect model**		7486	0.04	[0.04; 0.04]	35.9%	--
**Random effects model**			0.19	[0.12; 0.27]	--	22.0%
Heterogeneity: I^2 = 98%, τ^2 = 0.0357, p < 0.01						
**Common effect model**		**29190**	**0.07**	**[0.07; 0.08]**	**100.0%**	**--**
**Random effects model**			**0.21**	**[0.18; 0.25]**	**--**	**100.0%**

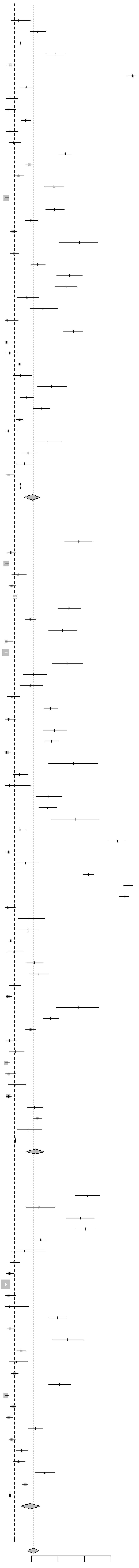

Heterogeneity: I^2 = 99%, τ^2 = 0.0443, p = 0
Test for subgroup differences (fixed effect): χ^2_2 = 555.83, df = 2 (p < 0.01)
Test for subgroup differences (random effects): χ^2_2 = 0.54, df = 2 (p = 0.76)

Figure S5B: Subgroup analysis of the prevalence of protozoan infections in people living with viruses by age

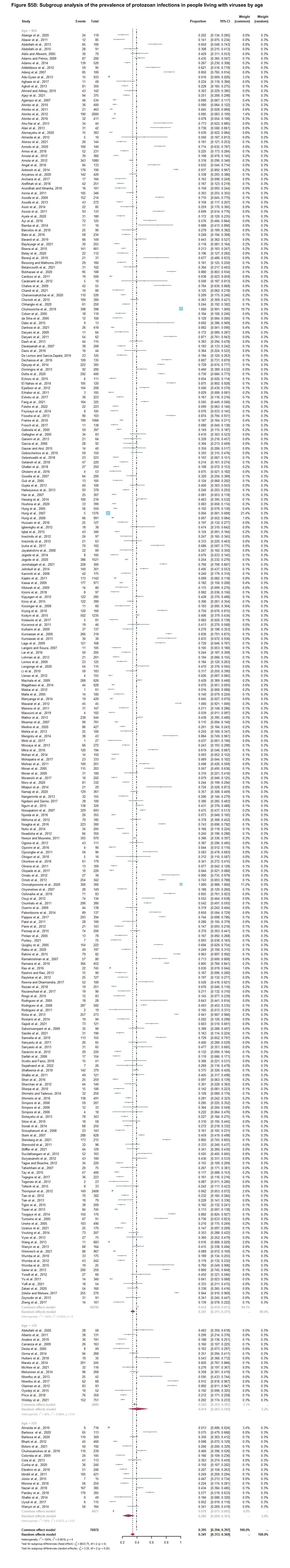

Figure S6A:

Subgroup analysis of the prevalence of helminth infections in people living with viruses by gender

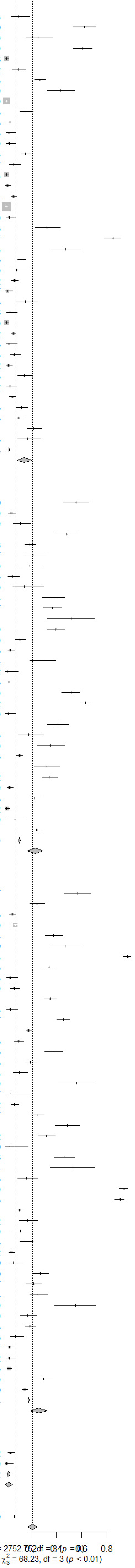

Study	Events	Total	Proportion	95%-CI	Weight (common)	Weight (random)
**Gender_at_Risk = N/A**						
Abaver et al., 2011	9	85	0.11	[0.05; 0.19]	0.1%	0.8%
Abbas et al., 2009	74	119	0.62	[0.53; 0.71]	0.1%	0.8%
Abilo and Afework, 2005	18	70	0.26	[0.16; 0.38]	0.1%	0.8%
Adams et al., 2006	103	170	0.61	[0.53; 0.68]	0.1%	0.8%
Agholi et al., 2013	4	356	0.01	[0.00; 0.03]	4.9%	0.8%
Ahmed and Abhay, 2015	14	142	0.10	[0.05; 0.16]	0.2%	0.8%
Akinbo et al., 2011	125	463	0.27	[0.23; 0.31]	0.4%	0.8%
Babatunde et al., 2010	39	90	0.43	[0.33; 0.54]	0.1%	0.8%
Cardoso et al., 2011	4	500	0.01	[0.00; 0.02]	9.7%	0.8%
Eshetu et al., 2017	36	223	0.16	[0.12; 0.22]	0.3%	0.8%
Gebretsadik et al., 2018	8	223	0.04	[0.02; 0.07]	1.0%	0.8%
Guk et al., 2005	3	105	0.03	[0.01; 0.08]	0.6%	0.8%
Gupta et al., 2013	3	100	0.03	[0.01; 0.09]	0.5%	0.8%
Haileeyesus et al., 2013	59	378	0.16	[0.12; 0.20]	0.4%	0.8%
Kulkarni et al., 2009	9	137	0.07	[0.03; 0.12]	0.3%	0.8%
Kurniawan et al., 2009	3	318	0.01	[0.00; 0.03]	5.2%	0.8%
Lehman et al., 2013	3	201	0.01	[0.00; 0.04]	2.1%	0.8%
Mathur et al., 2013	34	544	0.06	[0.04; 0.09]	1.4%	0.8%
Meamar et al., 2007	5	781	0.01	[0.00; 0.01]	18.8%	0.8%
Mehta et al., 2013	3	100	0.03	[0.01; 0.09]	0.5%	0.8%
Mkhize et al., 2017	31	95	0.33	[0.23; 0.43]	0.1%	0.8%
Paboriboune et al., 2014	116	137	0.85	[0.78; 0.90]	0.2%	0.8%
Pinlaor et al., 2005	37	78	0.47	[0.36; 0.59]	0.0%	0.8%
Plumelle et al., 2015	55	445	0.12	[0.09; 0.16]	0.6%	0.8%
Ramakrishnan et al., 2007	7	80	0.09	[0.04; 0.17]	0.2%	0.8%
Ramana et al., 2010	32	452	0.07	[0.05; 0.10]	1.1%	0.8%
Raytekar et al., 2012	2	127	0.02	[0.00; 0.06]	1.3%	0.8%
Ringo et al., 2015	13	83	0.16	[0.09; 0.25]	0.1%	0.8%
Sherpa et al., 2010	4	106	0.04	[0.01; 0.09]	0.4%	0.8%
Sonali et al., 2014	2	250	0.01	[0.00; 0.03]	4.8%	0.8%
Talaat et al., 2008	40	632	0.06	[0.05; 0.09]	1.6%	0.8%
Vyas et al., 2013	2	75	0.03	[0.00; 0.09]	0.4%	0.8%
Wumba et al., 2010	12	175	0.07	[0.04; 0.12]	0.4%	0.8%
Wumba et al., 2012	6	242	0.02	[0.01; 0.05]	1.5%	0.8%
Sannella et al., 2019	23	155	0.15	[0.10; 0.21]	0.2%	0.8%
Belay et al., 2020	4	112	0.04	[0.01; 0.09]	0.5%	0.8%
Namaji et al., 2020	19	361	0.05	[0.03; 0.08]	1.1%	0.8%
Bayleyegn et al., 2021	32	255	0.13	[0.09; 0.17]	0.4%	0.8%
Feleke et al., 2022	23	223	0.10	[0.07; 0.15]	0.4%	0.8%
Talukder et al., 2021	45	201	0.22	[0.17; 0.29]	0.2%	0.8%
Purbey., 2021	13	75	0.17	[0.10; 0.28]	0.1%	0.8%
Common effect model		9464	0.03	[0.03; 0.03]	62.4%	--
Random effects model			0.15	[0.09; 0.21]	--	33.5%
Heterogeneity: I^2 = 98%, τ^2 = 0.0349, p = 0						
**Gender_at_Risk = Male**						
Abbas et al., 2009	55	99	0.56	[0.45; 0.66]	0.1%	0.8%
Adamu and Petros, 2009	9	200	0.04	[0.02; 0.08]	0.7%	0.8%
Adesij et al. 2007	12	100	0.12	[0.06; 0.20]	0.1%	0.8%
Allam et al., 2014	68	141	0.48	[0.40; 0.57]	0.1%	0.8%
Asma et al., 2011	66	346	0.19	[0.15; 0.24]	0.3%	0.8%
Costiniuk et al., 2012	21	97	0.22	[0.14; 0.31]	0.1%	0.8%
Da Silva et al., 2005	19	100	0.19	[0.12; 0.28]	0.1%	0.8%
Dash et al., 2013	6	115	0.05	[0.02; 0.11]	0.4%	0.8%
Gaurav and Atul, 2015	6	40	0.15	[0.06; 0.30]	0.0%	0.8%
Hochberg et al., 2011	48	128	0.38	[0.29; 0.46]	0.1%	0.8%
Kroidl et al., 2016	69	187	0.37	[0.30; 0.44]	0.1%	0.8%
Loffredo et al., 2015	16	31	0.52	[0.33; 0.70]	0.0%	0.7%
Mulu et al., 2013	87	220	0.40	[0.33; 0.46]	0.1%	0.8%
Nsagha et al., 2016	34	300	0.11	[0.08; 0.15]	0.5%	0.8%
Nuhu et al., 2014	8	205	0.04	[0.02; 0.08]	0.8%	0.8%
Orji et al., 2017	24	84	0.29	[0.19; 0.39]	0.1%	0.8%
Oyedeji et al., 2015	1	52	0.02	[0.00; 0.10]	0.4%	0.8%
Pavie et al., 2012	4	143	0.03	[0.01; 0.07]	0.8%	0.8%
Pennap et al., 2015	103	200	0.52	[0.44; 0.59]	0.1%	0.8%
Rodrigues et al., 2008	366	582	0.63	[0.59; 0.67]	0.4%	0.8%
Saksirisampant et al., 2009	2	90	0.02	[0.00; 0.08]	0.6%	0.8%
Samie et al., 2014	62	151	0.41	[0.33; 0.49]	0.1%	0.8%
Sanyaolu et al., 2011	12	65	0.18	[0.10; 0.30]	0.1%	0.8%
Talaat et al., 2015	28	80	0.35	[0.25; 0.46]	0.1%	0.8%
Tiwari et al., 2013	83	745	0.11	[0.09; 0.14]	1.2%	0.8%
Zeynudin et al., 2013	29	91	0.32	[0.22; 0.42]	0.1%	0.8%
Kaneshiro et al., 2019	87	252	0.35	[0.29; 0.41]	0.2%	0.8%
Chhangte et al., 2020	8	250	0.03	[0.01; 0.06]	1.2%	0.8%
McLellan et al., 2020	58	253	0.23	[0.18; 0.29]	0.2%	0.8%
Botero et al., 2021	2	192	0.01	[0.00; 0.04]	2.9%	0.8%
Chandi et al., 2021	6	80	0.07	[0.03; 0.16]	0.2%	0.8%
Wolday et al., 2021	184	751	0.25	[0.21; 0.28]	0.6%	0.8%
Common effect model		6370	0.11	[0.10; 0.12]	12.8%	--
Random effects model			0.23	[0.17; 0.30]	--	25.8%
Heterogeneity: I^2 = 98%, τ^2 = 0.0316, p = 0						
**Gender_at_Risk = Female**						
Abossie and Petros, 2015	55	97	0.57	[0.46; 0.67]	0.1%	0.8%
Adeleke et al., 2015	57	231	0.25	[0.19; 0.31]	0.2%	0.8%
Akinbo and Omoregie, 2011	15	285	0.05	[0.03; 0.09]	0.9%	0.8%
Akinbo et al., 2012	156	2000	0.08	[0.07; 0.09]	4.3%	0.8%
Assefa et al., 2009	81	214	0.38	[0.31; 0.45]	0.1%	0.8%
Colombe et al., 2018	37	79	0.47	[0.36; 0.58]	0.0%	0.8%
Erikstrup et al., 2008	190	198	0.96	[0.92; 0.98]	0.8%	0.8%
Fekadu et al., 2013	118	343	0.34	[0.29; 0.40]	0.2%	0.8%
Feyisayo et al., 2014	4	105	0.04	[0.01; 0.09]	0.4%	0.8%
Getaneh et al., 2018	15	220	0.07	[0.04; 0.11]	0.5%	0.8%
Idindili et al., 2011	148	421	0.35	[0.31; 0.40]	0.3%	0.8%
Jegede et al., 2014	4	105	0.04	[0.01; 0.09]	0.4%	0.8%
Kallestrup et al., 2005	185	407	0.45	[0.41; 0.50]	0.3%	0.8%
Kawai et al., 2009	178	971	0.18	[0.16; 0.21]	1.0%	0.8%
Kipyegen et al., 2012	29	285	0.10	[0.07; 0.14]	0.5%	0.8%
Lar et al., 2016	77	205	0.38	[0.31; 0.45]	0.1%	0.8%
Marti et al., 2017	60	305	0.20	[0.15; 0.25]	0.3%	0.8%
Mascarello et al., 2011	15	138	0.11	[0.06; 0.17]	0.2%	0.8%
Mazigo et al., 2016	28	50	0.56	[0.41; 0.70]	0.0%	0.7%
Minti et al., 2017	1	27	0.04	[0.00; 0.19]	0.1%	0.8%
Missaye et al., 2013	19	272	0.07	[0.04; 0.11]	0.6%	0.8%
Modjarrad et al., 2005	74	297	0.25	[0.20; 0.30]	0.2%	0.8%
Modjarrad et al., 2005	54	111	0.49	[0.39; 0.58]	0.1%	0.8%
Morawski et al., 2017	65	202	0.32	[0.26; 0.39]	0.1%	0.8%
Mtapuri et al., 2014	1	29	0.03	[0.00; 0.18]	0.1%	0.8%
Ndhlovu et al., 2007	72	156	0.46	[0.38; 0.54]	0.1%	0.8%
Nielsen et al., 2007	18	34	0.53	[0.35; 0.70]	0.0%	0.7%
Ojurongbe et al., 2011	16	96	0.17	[0.10; 0.26]	0.1%	0.8%
Roka et al., 2012	242	260	0.93	[0.89; 0.96]	0.6%	0.8%
Roka et al., 2013	246	273	0.90	[0.86; 0.93]	0.5%	0.8%
Shimelis et al., 2016	54	491	0.11	[0.08; 0.14]	0.8%	0.8%
Smith et al., 2008	21	122	0.17	[0.11; 0.25]	0.1%	0.8%
Suryawanshi et al., 2012	12	100	0.12	[0.06; 0.20]	0.1%	0.8%
Tegegne et al., 2017	36	223	0.16	[0.12; 0.22]	0.3%	0.8%
Tian et al., 2012	14	302	0.05	[0.03; 0.08]	1.0%	0.8%
Tian et al., 2013	5	79	0.06	[0.02; 0.14]	0.2%	0.8%
Tigist et al., 2015	57	209	0.27	[0.21; 0.34]	0.2%	0.8%
Vouking et al., 2014	46	207	0.22	[0.17; 0.29]	0.2%	0.8%
Wanyiri et al., 2013	42	164	0.26	[0.19; 0.33]	0.1%	0.8%
Xiao et al., 2015	22	40	0.55	[0.38; 0.71]	0.0%	0.7%
Gebrecherkos et al., 2019	26	150	0.17	[0.12; 0.24]	0.2%	0.8%
Alemayehu etal., 2020	74	383	0.19	[0.15; 0.24]	0.4%	0.8%
Lengongo et al., 2020	9	115	0.08	[0.04; 0.14]	0.2%	0.8%
Rabiu et al., 2020	6	197	0.03	[0.01; 0.07]	1.0%	0.8%
Betancourth et al., 2021	3	102	0.03	[0.01; 0.08]	0.5%	0.8%
Ajayi et al., 2021	10	375	0.03	[0.01; 0.05]	2.2%	0.8%
Uzairue et al., 2021	51	170	0.30	[0.23; 0.37]	0.1%	0.8%
PrayGod et al., 2022	176	1149	0.15	[0.13; 0.18]	1.4%	0.8%
Common effect model		12994	0.18	[0.18; 0.19]	22.5%	--
Random effects model			0.26	[0.20; 0.33]	--	39.0%
Heterogeneity: I^2 = 99%, τ^2 = 0.0564, p = 0						
**Gender_at_Risk = Both**						
Assefa et al., 2013	11	272	0.04	[0.02; 0.07]	1.1%	0.8%
Barcelos et al., 2018	1	90	0.01	[0.00; 0.06]	1.3%	0.8%
Common effect model		362	0.02	[0.01; 0.04]	2.3%	--
Random effects model			0.03	[0.00; 0.05]	--	1.7%
Heterogeneity: I^2 = 69%, τ^2 = 0.0003, p = 0.07						
**Common effect model**		**29190**	**0.07**	**[0.07; 0.08]**	**100.0%**	**--**
**Random effects model**			**0.21**	**[0.18; 0.25]**	**--**	**100.0%**

Heterogeneity: I^2 = 99%, τ^2 = 0.0443, p = 0

Test for subgroup differences (fixed effect): χ^2_3 = 2752.7[illegible], df = [illegible] (p = [illegible])

Test for subgroup differences (random effects): χ^2_3 = 68.23, df = 3 (p < 0.01)

Figure S6B: Subgroup analysis of the prevalence of protozoan infections in people living with viruses by gender

Study	Events	Total	Proportion	95%-CI	Weight (common)	Weight (random)
**Gender_at_risk = Female**						
Thompson et al., 2012	149	2408	0.06	[0.05; 0.07]	2.6%	0.3%
Amodu et al., 2020	100	140	0.71	[0.63; 0.79]	0.0%	0.3%
Kiying et al., 2010	120	160	0.75	[0.68; 0.81]	0.1%	0.3%
Oyedeji et al., 2015	10	52	0.19	[0.10; 0.33]	0.0%	0.3%
Bate et al., 2016	58	234	0.25	[0.19; 0.31]	0.1%	0.3%
Ako-Nai et al., 2013	34	44	0.77	[0.62; 0.89]	0.0%	0.3%
Anteneh et al., 2014	178	190	0.94	[0.89; 0.97]	0.2%	0.3%
Nkhoma et al., 2012	70	185	0.38	[0.31; 0.45]	0.0%	0.3%
Franke et al., 2010	199	1066	0.19	[0.16; 0.21]	0.4%	0.3%
Kawai et al., 2009	177	971	0.18	[0.16; 0.21]	0.4%	0.3%
Ojurongbe et al., 2011	56	96	0.58	[0.48; 0.68]	0.0%	0.3%
Akinbo et al., 2010	36	400	0.09	[0.06; 0.12]	0.3%	0.3%
Goselle et al., 2007	64	200	0.32	[0.26; 0.39]	0.1%	0.3%
Kinimi et al., 2018	8	97	0.08	[0.04; 0.16]	0.1%	0.3%
Tigist et al., 2015	38	209	0.18	[0.13; 0.24]	0.1%	0.3%
Djieyep et al., 2014	222	305	0.73	[0.67; 0.78]	0.1%	0.3%
Nwadioha et al., 2012	84	350	0.24	[0.20; 0.29]	0.1%	0.3%
Izuka et al., 2017	70	102	0.69	[0.59; 0.77]	0.0%	0.3%
Kfutwah et al., 2009	14	80	0.17	[0.10; 0.28]	0.0%	0.3%
Abdullahi et al., 2020	28	58	0.48	[0.35; 0.62]	0.0%	0.3%
Alonso et al., 2021	26	144	0.18	[0.12; 0.25]	0.1%	0.3%
Fisseha et al., 2013	90	103	0.87	[0.79; 0.93]	0.1%	0.3%
Anabire et al., 2019	36	191	0.19	[0.14; 0.25]	0.1%	0.3%
Manyanga et al., 2014	19	420	0.05	[0.03; 0.07]	0.6%	0.3%
Wumba et al., 2015	19	25	0.76	[0.55; 0.91]	0.0%	0.3%
Price et al., 2018	76	359	0.21	[0.17; 0.26]	0.1%	0.3%
Frosch et al., 2017	11	138	0.08	[0.04; 0.14]	0.1%	0.3%
Zheng et al., 2017	14	101	0.14	[0.08; 0.22]	0.1%	0.3%
Olaniran et al., 2012	83	93	0.89	[0.81; 0.95]	0.1%	0.3%
Domingos et al., 2013	92	200	0.46	[0.39; 0.53]	0.0%	0.3%
Kipyegen et al., 2012	122	285	0.43	[0.37; 0.49]	0.1%	0.3%
Mengistu et al., 2014	38	43	0.88	[0.75; 0.96]	0.0%	0.3%
Ngobeni and Samie, 2017	38	100	0.38	[0.28; 0.48]	0.0%	0.3%
Nkenfou et al., 2013	25	42	0.60	[0.43; 0.74]	0.0%	0.3%
Srisuphanunt et al., 2008	23	143	0.16	[0.10; 0.23]	0.1%	0.3%
Johnbull et al., 2014	149	301	0.50	[0.44; 0.55]	0.1%	0.3%
Assefa et al., 2009	152	214	0.71	[0.64; 0.77]	0.1%	0.3%
Rabiu et al., 2020	49	197	0.25	[0.19; 0.32]	0.1%	0.3%
Tegegne et al., 2017	36	223	0.16	[0.12; 0.22]	0.1%	0.3%
Zeleke and Melsew, 2017	255	270	0.94	[0.91; 0.97]	0.3%	0.3%
Amoo et al., 2018	52	231	0.23	[0.17; 0.28]	0.1%	0.3%
Nkenfou et al., 2017	62	68	0.91	[0.82; 0.97]	0.0%	0.3%
Sitoe et al., 2010	18	58	0.31	[0.20; 0.45]	0.0%	0.3%
Tay et al., 2015	47	400	0.12	[0.09; 0.15]	0.2%	0.3%
Hasang et al., 2014	100	216	0.46	[0.40; 0.53]	0.1%	0.3%
Shimelis et al., 2016	138	491	0.28	[0.24; 0.32]	0.2%	0.3%
Daryani et al., 2009	11	64	0.17	[0.09; 0.29]	0.0%	0.3%
Missaye et al., 2013	66	272	0.24	[0.19; 0.30]	0.1%	0.3%
Dechassa et al., 2016	109	135	0.81	[0.73; 0.87]	0.1%	0.3%
Girma et al., 2014	94	268	0.35	[0.29; 0.41]	0.1%	0.3%
Mekonnen et al., 2014	96	268	0.36	[0.30; 0.42]	0.1%	0.3%
Suryawanshi et al., 2012	43	100	0.43	[0.33; 0.53]	0.0%	0.3%
Feyisayo et al., 2014	8	105	0.08	[0.03; 0.14]	0.1%	0.3%
Jegede et al., 2014	8	105	0.08	[0.03; 0.14]	0.1%	0.3%
Lar et al., 2016	50	205	0.24	[0.19; 0.31]	0.1%	0.3%
Gebrecherkos et al., 2019	59	150	0.39	[0.31; 0.48]	0.0%	0.3%
Kiros et al., 2015	122	399	0.31	[0.26; 0.35]	0.1%	0.3%
Morawski et al., 2017	16	202	0.08	[0.05; 0.13]	0.2%	0.3%
Nweze and Mouneke, 2011	355	970	0.37	[0.34; 0.40]	0.3%	0.3%
Ogoina et al., 2013	43	111	0.39	[0.30; 0.48]	0.0%	0.3%
Ouermi et al., 2009	44	138	0.32	[0.24; 0.40]	0.0%	0.3%
Njunda et al., 2016	26	355	0.07	[0.05; 0.11]	0.3%	0.3%
Idindili et al., 2011	105	421	0.25	[0.21; 0.29]	0.1%	0.3%
Minti et al., 2017	1	27	0.04	[0.00; 0.19]	0.0%	0.3%
Saracino et al., 2012	29	220	0.13	[0.09; 0.18]	0.1%	0.3%
Sandie et al., 2021	31	190	0.16	[0.11; 0.22]	0.1%	0.3%
Tsegaye et al., 2014	150	170	0.88	[0.82; 0.93]	0.1%	0.3%
Roka et al., 2013	257	273	0.94	[0.91; 0.97]	0.3%	0.3%
Iroezindu et al., 2012	21	63	0.33	[0.22; 0.46]	0.0%	0.3%
Masese et al., 2011	31	147	0.21	[0.15; 0.29]	0.1%	0.3%
Wanyiri et al., 2013	68	164	0.41	[0.34; 0.49]	0.0%	0.3%
Vouking et al., 2014	73	207	0.35	[0.29; 0.42]	0.1%	0.3%
Erhabor et al., 2011	3	105	0.03	[0.01; 0.08]	0.2%	0.3%
Wanyiri et al., 2014	56	164	0.34	[0.27; 0.42]	0.0%	0.3%
Olopade et al., 2017	10	226	0.04	[0.02; 0.08]	0.3%	0.3%
Akinbo et al., 2012	190	2000	0.10	[0.08; 0.11]	1.4%	0.3%
Alemayehu et al., 2020	15	383	0.04	[0.02; 0.06]	0.6%	0.3%
Jegede et al., 2020	386	1521	0.25	[0.23; 0.28]	0.5%	0.3%
Sintayehu et al., 2013	78	343	0.23	[0.18; 0.28]	0.1%	0.3%
Yu et al. 2011	14	340	0.04	[0.02; 0.07]	0.5%	0.3%
Kissinger et al., 2008	11	60	0.18	[0.10; 0.30]	0.0%	0.3%
Omoti et al., 2013	254	342	0.74	[0.69; 0.79]	0.1%	0.3%
Cota et al., 2013	41	113	0.36	[0.27; 0.46]	0.0%	0.3%
Davis et al., 2016	16	44	0.36	[0.22; 0.52]	0.0%	0.3%
Pappoe et al., 2017	293	394	0.74	[0.70; 0.79]	0.1%	0.3%
Ajayi et al., 2021	94	375	0.25	[0.21; 0.30]	0.1%	0.3%
Fang et al., 2021	164	325	0.50	[0.45; 0.56]	0.1%	0.3%
Almeida et al., 2010	9	716	0.01	[0.01; 0.02]	3.4%	0.3%
Chukwuanukwu et al., 2016	110	239	0.46	[0.40; 0.53]	0.1%	0.3%
Ojuromi et al., 2016	4	90	0.04	[0.01; 0.11]	0.1%	0.3%
Tian et al., 2012	70	302	0.23	[0.19; 0.28]	0.1%	0.3%
Tian et al., 2013	18	79	0.23	[0.14; 0.34]	0.0%	0.3%
Pandey et al., 2018	176	305	0.58	[0.52; 0.63]	0.1%	0.3%
Betancourth et al., 2021	31	102	0.30	[0.22; 0.40]	0.0%	0.3%
Wang et al., 2013	11	683	0.02	[0.01; 0.03]	2.6%	0.3%
Lengongo et al., 2020	54	115	0.47	[0.38; 0.56]	0.0%	0.3%
Dankwa et al., 2021	26	418	0.06	[0.04; 0.09]	0.4%	0.3%
Akinbo et al., 2016	32	411	0.08	[0.05; 0.11]	0.3%	0.3%
Amuta et al., 2012	39	360	0.11	[0.08; 0.15]	0.2%	0.3%
Angal et al., 2016	84	133	0.63	[0.54; 0.71]	0.0%	0.3%
Assob et al., 2011	93	133	0.70	[0.61; 0.78]	0.0%	0.3%
Bamba et al., 2014	11	16	0.69	[0.41; 0.89]	0.0%	0.3%
Bessong and Mathomu 2010	29	160	0.18	[0.12; 0.25]	0.1%	0.3%
De Lemos and Garcia-Zapata, 2010	23	125	0.18	[0.12; 0.26]	0.1%	0.3%
Gallagher et al., 2005	34	83	0.41	[0.30; 0.52]	0.0%	0.3%
Getaneh et al., 2018	47	220	0.21	[0.16; 0.27]	0.1%	0.3%
Lago et al., 2009	121	168	0.72	[0.65; 0.79]	0.1%	0.3%
Lemos et al., 2009	23	125	0.18	[0.12; 0.26]	0.1%	0.3%
Mtapuri et al., 2014	21	29	0.72	[0.53; 0.87]	0.0%	0.3%
Nangammbi et al., 2013	32	155	0.21	[0.15; 0.28]	0.1%	0.3%
Okogun et al., 2010	5	16	0.31	[0.11; 0.59]	0.0%	0.3%
Omalu et al., 2012	27	30	0.90	[0.73; 0.98]	0.0%	0.3%
Tegenaw et al., 2013	2	23	0.09	[0.01; 0.28]	0.0%	0.3%
Chukwuocha et al., 2019	398	398	1.00	[0.99; 1.00]	19.7%	0.3%
Ayele et al., 2020	31	180	0.17	[0.12; 0.24]	0.1%	0.3%
Medina et al., 2020	86	427	0.20	[0.16; 0.24]	0.2%	0.3%
Zakari et al., 2020	54	160	0.34	[0.26; 0.42]	0.0%	0.3%
Uzairue et al., 2021	25	170	0.15	[0.10; 0.21]	0.1%	0.3%
Jemikalajah et al., 2021	228	300	0.76	[0.71; 0.81]	0.1%	0.3%
Common effect model		31738	0.56	[0.56; 0.56]	42.1%	--
Random effects model			0.36	[0.31; 0.41]	--	38.8%
Heterogeneity: $I^2 = 100\%$, $\tau^2 = 0.0716$, $p = 0$						
**Gender_at_risk = N/A**						
Tumwine et al., 2005	67	91	0.74	[0.63; 0.82]	0.0%	0.3%
Abange et al., 2020	24	119	0.20	[0.13; 0.29]	0.0%	0.3%
Rao et al., 2016	22	745	0.03	[0.02; 0.04]	1.6%	0.3%
Scotto and Fazio, 2018	15	41	0.37	[0.22; 0.53]	0.0%	0.3%
Simpore et al., 2006	59	207	0.29	[0.22; 0.35]	0.1%	0.3%
Simpore et al., 2006	12	33	0.36	[0.20; 0.55]	0.0%	0.3%
Simpore et al., 2006	4	18	0.22	[0.06; 0.48]	0.0%	0.3%
Kurniawan et al., 2013	30	36	0.83	[0.67; 0.94]	0.0%	0.3%
Kapito et al., 2011	113	1142	0.10	[0.08; 0.12]	0.8%	0.3%
Sherpa et al., 2010	15	106	0.14	[0.08; 0.22]	0.1%	0.3%
Abaver et al., 2011	12	85	0.14	[0.08; 0.23]	0.0%	0.3%
Mohan et al., 2014	14	150	0.09	[0.05; 0.15]	0.1%	0.3%
Mathur et al., 2013	238	544	0.44	[0.40; 0.48]	0.1%	0.3%
Haileeyesus et al., 2013	93	378	0.25	[0.20; 0.29]	0.1%	0.3%
Kurniawan et al., 2009	266	318	0.84	[0.79; 0.88]	0.1%	0.3%
Eshetu et al., 2017	36	223	0.16	[0.12; 0.22]	0.1%	0.3%
Masoumi et al., 2019	4	102	0.04	[0.01; 0.10]	0.2%	0.3%
Vyas et al., 2013	27	75	0.36	[0.25; 0.48]	0.0%	0.3%
Mohapatra et al., 2017	23	333	0.07	[0.04; 0.10]	0.3%	0.3%
McAtee et al., 2021	32	116	0.28	[0.20; 0.37]	0.0%	0.3%
Daryani et al., 2011	54	62	0.87	[0.76; 0.94]	0.0%	0.3%
Chalwe et al., 2009	42	55	0.76	[0.63; 0.87]	0.0%	0.3%
Gupta et al., 2013	40	100	0.40	[0.30; 0.50]	0.0%	0.3%
Kulkarni et al., 2009	37	137	0.27	[0.20; 0.35]	0.0%	0.3%
Raytekar et al., 2012	25	127	0.20	[0.13; 0.28]	0.0%	0.3%
Mitra et al., 2016	120	194	0.62	[0.55; 0.69]	0.1%	0.3%
Alavi et al., 2013	31	42	0.74	[0.58; 0.86]	0.0%	0.3%
Sannella et al., 2019	113	155	0.73	[0.65; 0.80]	0.0%	0.3%
Kolawole et al., 2017	5	81	0.06	[0.02; 0.14]	0.1%	0.3%
Belay et al., 2020	22	112	0.20	[0.13; 0.28]	0.0%	0.3%
Ahmed and Abhay, 2015	43	142	0.30	[0.23; 0.39]	0.0%	0.3%
Langoni and Souza, 2007	11	105	0.10	[0.05; 0.18]	0.1%	0.3%
Hari et al., 2007	25	307	0.08	[0.05; 0.12]	0.3%	0.3%
Iroezindu et al., 2012	24	97	0.25	[0.17; 0.35]	0.0%	0.3%
Paboriboune et al., 2014	89	137	0.65	[0.56; 0.73]	0.0%	0.3%
Ringo et al., 2015	12	83	0.14	[0.08; 0.24]	0.0%	0.3%
Rodrigues et al., 2011	2	19	0.11	[0.01; 0.33]	0.0%	0.3%
Sarfati et al., 2006	17	154	0.11	[0.07; 0.17]	0.1%	0.3%
Purbey., 2021	7	75	0.09	[0.04; 0.18]	0.1%	0.3%
Agholi et al., 2013	81	356	0.23	[0.18; 0.27]	0.1%	0.3%
Lehman et al., 2013	21	201	0.10	[0.07; 0.16]	0.1%	0.3%
Osuji et al., 2012	74	134	0.55	[0.46; 0.64]	0.0%	0.3%
Guk et al., 2005	13	105	0.12	[0.07; 0.20]	0.1%	0.3%
Wumba et al., 2012	43	242	0.18	[0.13; 0.23]	0.1%	0.3%
Adu-Gyasi et al., 2013	15	933	0.02	[0.01; 0.03]	3.5%	0.3%
Echoru et al., 2015	6	111	0.05	[0.02; 0.11]	0.1%	0.3%
Morona et al., 2018	56	250	0.22	[0.17; 0.28]	0.1%	0.3%
Rezaei et al., 2018	19	251	0.08	[0.05; 0.12]	0.2%	0.3%
Garcia et al., 2006	28	92	0.30	[0.21; 0.41]	0.0%	0.3%
Cardoso et al., 2011	19	500	0.04	[0.02; 0.06]	0.8%	0.3%
Wumba et al., 2010	33	175	0.19	[0.13; 0.25]	0.1%	0.3%
El Nahas et al., 2014	105	120	0.88	[0.80; 0.93]	0.1%	0.3%
Abilo and Afework, 2005	30	70	0.43	[0.31; 0.55]	0.0%	0.3%
Akinbo et al., 2011	21	463	0.05	[0.03; 0.07]	0.6%	0.3%
Amuta et al., 2012	343	1080	0.32	[0.29; 0.35]	0.3%	0.3%
Arundhati and Niharika, 2016	16	101	0.16	[0.09; 0.24]	0.0%	0.3%
Carranza et al., 2009	26	163	0.16	[0.11; 0.22]	0.1%	0.3%
Gebretsadik et al., 2018	23	223	0.10	[0.07; 0.15]	0.1%	0.3%
Ghafari et al., 2018	27	250	0.11	[0.07; 0.15]	0.2%	0.3%
Igbeneghu et al., 2012	18	38	0.47	[0.31; 0.64]	0.0%	0.3%
Jayalakshmi et al., 2008	22	89	0.25	[0.16; 0.35]	0.0%	0.3%
Kammili et al., 2008	42	175	0.24	[0.18; 0.31]	0.1%	0.3%
Kucerova et al., 2011	19	46	0.41	[0.27; 0.57]	0.0%	0.3%
Malla et al., 2005	14	100	0.14	[0.08; 0.22]	0.1%	0.3%
Meamar et al., 2007	90	781	0.12	[0.09; 0.14]	0.5%	0.3%
Mehta et al., 2013	25	100	0.25	[0.17; 0.35]	0.0%	0.3%
Moran et al., 2009	31	100	0.31	[0.22; 0.41]	0.0%	0.3%
Moran et al., 2005	115	203	0.57	[0.50; 0.64]	0.1%	0.3%
Oliveira et al., 2011	13	169	0.08	[0.04; 0.13]	0.1%	0.3%
Oshinaike et al., 2010	71	83	0.86	[0.76; 0.92]	0.0%	0.3%
Patel et al., 2011	28	100	0.28	[0.19; 0.38]	0.0%	0.3%
Pinlaor et al., 2005	13	78	0.17	[0.09; 0.27]	0.0%	0.3%
Ramakrishnan et al., 2007	57	80	0.71	[0.60; 0.81]	0.0%	0.3%
Ramana et al., 2010	364	452	0.81	[0.77; 0.84]	0.2%	0.3%
Rashmi and Ravi, 2013	15	90	0.17	[0.10; 0.26]	0.0%	0.3%
Sanyaolu et al., 2013	31	65	0.48	[0.35; 0.60]	0.0%	0.3%
Shafieenia et al., 2018	142	379	0.37	[0.33; 0.43]	0.1%	0.3%
Shimelis and Tadesse, 2014	32	250	0.13	[0.09; 0.18]	0.1%	0.3%
Sonali et al., 2014	68	250	0.27	[0.22; 0.33]	0.1%	0.3%
Tagoe and Boachie, 2012	34	220	0.15	[0.11; 0.21]	0.1%	0.3%
Tellevik et al., 2015	8	33	0.24	[0.11; 0.42]	0.0%	0.3%
Namaji et al., 2020	129	361	0.36	[0.31; 0.41]	0.1%	0.3%
Stiffler et al., 2021	44	129	0.34	[0.26; 0.43]	0.0%	0.3%
Yulfi et al., 2021	18	54	0.33	[0.21; 0.47]	0.0%	0.3%
Weinreich et al., 2021	86	967	0.09	[0.07; 0.11]	0.7%	0.3%
Bayleyegn et al., 2021	30	255	0.12	[0.08; 0.16]	0.2%	0.3%
Feleke et al., 2022	22	223	0.10	[0.06; 0.15]	0.2%	0.3%
Common effect model		18736	0.13	[0.12; 0.13]	14.5%	--
Random effects model			0.29	[0.24; 0.34]	--	28.3%
Heterogeneity: $I^2 = 99\%$, $\tau^2 = 0.0543$, $p = 0$						
**Gender_at_risk = Male**						
Okonkwo et al., 2018	61	179	0.34	[0.27; 0.42]	0.0%	0.3%
Marete et al., 2014	201	245	0.82	[0.77; 0.87]	0.1%	0.3%
Alberto et al., 2011	38	131	0.29	[0.21; 0.38]	0.0%	0.3%
Huibers et al., 2018	19	35	0.54	[0.37; 0.71]	0.0%	0.3%
Assir et al., 2014	22	85	0.26	[0.17; 0.37]	0.0%	0.3%
Gholami et al., 2016	4	53	0.08	[0.02; 0.18]	0.0%	0.3%
Pennap et al., 2015	74	200	0.37	[0.30; 0.44]	0.1%	0.3%
Rezanezhad et al., 2017	19	90	0.21	[0.13; 0.31]	0.0%	0.3%
Iqbal et al., 2015	43	346	0.12	[0.09; 0.16]	0.2%	0.3%
Adesij et al. 2007	85	100	0.85	[0.76; 0.91]	0.0%	0.3%
Masarat et al., 2012	45	45	1.00	[0.92; 1.00]	0.3%	0.3%
Cohen et al., 2005	18	110	0.16	[0.10; 0.25]	0.0%	0.3%
Rahimi et al., 2015	79	82	0.96	[0.90; 0.99]	0.1%	0.3%
Uneke et al., 2005	103	490	0.21	[0.17; 0.25]	0.2%	0.3%
Li et al., 2010	58	183	0.32	[0.25; 0.39]	0.1%	0.3%
Beena et al., 2015	141	661	0.21	[0.18; 0.25]	0.2%	0.3%
Ganiem et al., 2013	21	64	0.33	[0.22; 0.46]	0.0%	0.3%
Reema and Dharmendra, 2017	52	100	0.52	[0.42; 0.62]	0.0%	0.3%
Machala et al., 2009	268	626	0.43	[0.39; 0.47]	0.2%	0.3%
Tiwari et al. 2013	84	745	0.11	[0.09; 0.14]	0.5%	0.3%
Sherchan et al., 2012	44	146	0.30	[0.23; 0.38]	0.0%	0.3%
Zeynudin et al., 2013	31	91	0.34	[0.24; 0.45]	0.0%	0.3%
Nissapatorn et al., 2007	329	693	0.47	[0.44; 0.51]	0.2%	0.3%
Hung et al., 2008	66	991	0.07	[0.05; 0.08]	1.0%	0.3%
Hung et al., 2007	5	1376	0.00	[0.00; 0.01]	21.4%	0.3%
Yosefi et al., 2012	27	60	0.45	[0.32; 0.58]	0.0%	0.3%
Maikai et al., 2012	1	61	0.02	[0.00; 0.09]	0.2%	0.3%
Ngum et al., 2015	138	320	0.43	[0.38; 0.49]	0.1%	0.3%
Onyenekwe et al., 2007	28	149	0.19	[0.13; 0.26]	0.1%	0.3%
Quigley et al., 2005	154	222	0.69	[0.63; 0.75]	0.1%	0.3%
Ajjampur et al., 2007	48	534	0.09	[0.07; 0.12]	0.4%	0.3%
Sanyaolu et al., 2011	26	65	0.40	[0.28; 0.53]	0.0%	0.3%
Rodrigues et al., 2008	287	582	0.49	[0.45; 0.53]	0.1%	0.3%
Sucilathangam et al., 2012	52	103	0.50	[0.40; 0.60]	0.0%	0.3%
Shafiei et al., 2011	49	121	0.40	[0.32; 0.50]	0.0%	0.3%
Nazari et al., 2018	167	385	0.43	[0.38; 0.48]	0.1%	0.3%
Aghaee et al., 2017	11	49	0.22	[0.12; 0.37]	0.0%	0.3%
Mohraz et al., 2011	100	201	0.50	[0.43; 0.57]	0.0%	0.3%
Barbosa et al., 2020	65	113	0.58	[0.48; 0.67]	0.0%	0.3%
Barbosa et al., 2020	110	309	0.36	[0.30; 0.41]	0.1%	0.3%
Steinberg et al., 2021	173	215	0.80	[0.75; 0.86]	0.1%	0.3%
Abdollahi et al., 2013	65	100	0.65	[0.55; 0.74]	0.0%	0.3%
Cavalcanti et al., 2012	5	10	0.50	[0.19; 0.81]	0.0%	0.2%
Desta et al., 2005	8	49	0.16	[0.07; 0.30]	0.0%	0.3%
Hung et al., 2005	56	550	0.10	[0.08; 0.13]	0.4%	0.3%
Júnior et al., 2015	7	15	0.47	[0.21; 0.73]	0.0%	0.3%
Llenas et al., 2012	4	155	0.03	[0.01; 0.06]	0.3%	0.3%
Wolday et al., 2021	152	751	0.20	[0.17; 0.23]	0.3%	0.3%
Almeida et al., 2012	5	10	0.50	[0.19; 0.81]	0.0%	0.2%
Abdollahi et al., 2015	28	91	0.31	[0.22; 0.41]	0.0%	0.3%
Asma et al., 2011	105	346	0.30	[0.26; 0.35]	0.1%	0.3%
Anyanwu et al., 2020	142	420	0.34	[0.29; 0.39]	0.1%	0.3%
Dash et al., 2013	44	115	0.38	[0.29; 0.48]	0.0%	0.3%
Gabriela et al., 2005	59	397	0.15	[0.12; 0.19]	0.2%	0.3%
Addebbous et al., 2012	59	95	0.62	[0.52; 0.72]	0.0%	0.3%
Rostami et al., 2014	19	94	0.20	[0.13; 0.30]	0.0%	0.3%
Ebrahim et al., 2018	51	246	0.21	[0.16; 0.26]	0.1%	0.3%
da Silva et al., 2005	12	100	0.12	[0.06; 0.20]	0.1%	0.3%
Berenji et al., 2010	21	31	0.68	[0.49; 0.83]	0.0%	0.3%
Xavier et al., 2013	200	250	0.80	[0.74; 0.85]	0.1%	0.3%
Osunkalu et al., 2011	206	380	0.54	[0.49; 0.59]	0.1%	0.3%
Davarpanah et al., 2007	38	208	0.18	[0.13; 0.24]	0.1%	0.3%
Chandi et al., 2021	10	80	0.12	[0.06; 0.22]	0.0%	0.3%
Saksirisampant et al., 2009	35	90	0.39	[0.29; 0.50]	0.0%	0.3%
Bavand et al., 2019	66	149	0.44	[0.36; 0.53]	0.0%	0.3%
Bharti et al., 2012	45	460	0.10	[0.07; 0.13]	0.3%	0.3%
Nsagha et al., 2016	223	300	0.74	[0.69; 0.79]	0.1%	0.3%
Chemoh et al., 2015	109	300	0.36	[0.31; 0.42]	0.1%	0.3%
Sajedi et al., 2021	73	121	0.60	[0.51; 0.69]	0.0%	0.3%
Arefkhah et al., 2018	42	251	0.17	[0.12; 0.22]	0.1%	0.3%
Adamu et al., 2014	139	520	0.27	[0.23; 0.31]	0.2%	0.3%
Pavie et al., 2012	21	143	0.15	[0.09; 0.22]	0.1%	0.3%
Adamu and Petros, 2009	87	200	0.44	[0.37; 0.51]	0.1%	0.3%
Colomba et al., 2009	24	145	0.17	[0.11; 0.24]	0.1%	0.3%
Ayi et al., 2016	72	125	0.58	[0.48; 0.66]	0.0%	0.3%
Shafiei et al., 2014	9	49	0.18	[0.09; 0.32]	0.0%	0.3%
Hoshina et al., 2020	33	399	0.08	[0.06; 0.11]	0.3%	0.3%
Botero et al., 2021	50	192	0.26	[0.20; 0.33]	0.1%	0.3%
Bokharaei et al., 2020	95	108	0.88	[0.80; 0.93]	0.1%	0.3%
Cunha et al., 2020	36	240	0.15	[0.11; 0.20]	0.1%	0.3%
Archana et al., 2017	17	104	0.16	[0.10; 0.25]	0.0%	0.3%
Dabo et al., 2015	9	13	0.69	[0.39; 0.91]	0.0%	0.3%
Epelboin et al., 2012	104	208	0.50	[0.43; 0.57]	0.1%	0.3%
Gaurav and Atul, 2015	14	40	0.35	[0.21; 0.52]	0.0%	0.3%
Hussain et al., 2016	25	127	0.20	[0.13; 0.28]	0.0%	0.3%
Kodym et al., 2015	502	1235	0.41	[0.38; 0.43]	0.3%	0.3%
Nuhu et al., 2014	34	205	0.17	[0.12; 0.22]	0.1%	0.3%
Rodrigues et al., 2005	18	28	0.64	[0.44; 0.81]	0.0%	0.3%
Shen et al., 2016	25	259	0.10	[0.06; 0.14]	0.2%	0.3%
Stark et al., 2007	288	628	0.46	[0.42; 0.50]	0.2%	0.3%
Stensvold et al., 2011	32	96	0.33	[0.24; 0.44]	0.0%	0.3%
Taherkhani et al., 2007	20	75	0.27	[0.17; 0.38]	0.0%	0.3%
Uysal et al., 2017	6	115	0.05	[0.02; 0.11]	0.1%	0.3%
Chhangte et al., 2020	61	250	0.24	[0.19; 0.30]	0.1%	0.3%
Dutta et al., 2020	292	400	0.73	[0.68; 0.77]	0.1%	0.3%
Onosakponome et al., 2020	300	300	1.00	[0.99; 1.00]	11.2%	0.3%
Sepahvand et al., 2022	7	26	0.27	[0.12; 0.48]	0.0%	0.3%
Common effect model		23750	0.33	[0.33; 0.33]	42.4%	--
Random effects model			0.37	[0.32; 0.42]	--	31.3%
Heterogeneity: $I^2 = 100\%$, $\tau^2 = 0.0566$, $p = 0$						
**Gender_at_risk = Both**						
Magalhaes et al., 2014	44	628	0.07	[0.05; 0.09]	0.6%	0.3%
Assefa et al., 2013	43	272	0.16	[0.12; 0.21]	0.1%	0.3%
Barcelos et al., 2018	25	90	0.28	[0.19; 0.38]	0.0%	0.3%
Charoensakulchai et al., 2020	110	526	0.21	[0.18; 0.25]	0.2%	0.3%
Moro et al., 2020	81	332	0.24	[0.20; 0.29]	0.1%	0.3%
Common effect model		1848	0.13	[0.12; 0.15]	1.1%	--
Random effects model			0.19	[0.12; 0.26]	--	1.6%
Heterogeneity: $I^2 = 96\%$, $\tau^2 = 0.0060$, $p < 0.01$						
**Common effect model**		**76072**	**0.40**	**[0.39; 0.40]**	**100.0%**	**--**
**Random effects model**			**0.34**	**[0.31; 0.37]**	**--**	**100.0%**

Heterogeneity: $I^2 = 100\%$, $\tau^2 = 0.0619$, $p = 0$

Test for subgroup differences (fixed effect): $\chi^2_3 = 39449.48$, df = 3 ($p = 0$)

Test for subgroup differences (random effects): $\chi^2_3 = 21.13$, df = 3 ($p < 0.01$)

Axis: 0.2 0.4 0.6 0.8 1

Fighure S7A:

Subgroup analysis of the prevalence of helminth infections in people living with viruses by publication year

Study	Events	Total	Proportion	95%-CI	Weight (common)	Weight (random)
**year = <2014**						
Abaver et al., 2011	9	85	0.11	[0.05; 0.19]	0.1%	0.8%
Abbas et al., 2009	55	99	0.56	[0.45; 0.66]	0.1%	0.8%
Abbas et al., 2009	74	119	0.62	[0.53; 0.71]	0.1%	0.8%
Abilo and Afework, 2005	18	70	0.26	[0.16; 0.38]	0.1%	0.8%
Adams et al., 2006	103	170	0.61	[0.53; 0.68]	0.1%	0.8%
Adamu and Petros, 2009	9	200	0.04	[0.02; 0.08]	0.7%	0.8%
Adesij et al. 2007	12	100	0.12	[0.06; 0.20]	0.1%	0.8%
Agholi et al., 2013	4	356	0.01	[0.00; 0.03]	4.9%	0.8%
Akinbo and Omoregie, 2011	15	285	0.05	[0.03; 0.09]	0.9%	0.8%
Akinbo et al., 2011	125	463	0.27	[0.23; 0.31]	0.4%	0.8%
Akinbo et al., 2012	156	2000	0.08	[0.07; 0.09]	4.3%	0.8%
Asma et al., 2011	66	346	0.19	[0.15; 0.24]	0.3%	0.8%
Assefa et al., 2009	81	214	0.38	[0.31; 0.45]	0.1%	0.8%
Assefa et al., 2013	11	272	0.04	[0.02; 0.07]	1.1%	0.8%
Babatunde et al., 2010	39	90	0.43	[0.33; 0.54]	0.1%	0.8%
Cardoso et al., 2011	4	500	0.01	[0.00; 0.02]	9.7%	0.8%
Costiniuk et al., 2012	21	97	0.22	[0.14; 0.31]	0.1%	0.8%
Da Silva et al., 2005	19	100	0.19	[0.12; 0.28]	0.1%	0.8%
Dash et al., 2013	6	115	0.05	[0.02; 0.11]	0.4%	0.8%
Erikstrup et al., 2008	190	198	0.96	[0.92; 0.98]	0.8%	0.8%
Fekadu et al., 2013	118	343	0.34	[0.29; 0.40]	0.2%	0.8%
Guk et al., 2005	3	105	0.03	[0.01; 0.08]	0.6%	0.8%
Gupta et al., 2013	3	100	0.03	[0.01; 0.09]	0.5%	0.8%
Haileeyesus et al., 2013	59	378	0.16	[0.12; 0.20]	0.4%	0.8%
Hochberg et al., 2011	48	128	0.38	[0.29; 0.46]	0.1%	0.8%
Idindili et al., 2011	148	421	0.35	[0.31; 0.40]	0.3%	0.8%
Kulkarni et al., 2009	9	137	0.07	[0.03; 0.12]	0.3%	0.8%
Kallestrup et al., 2005	185	407	0.45	[0.41; 0.50]	0.3%	0.8%
Kawai et al., 2009	178	971	0.18	[0.16; 0.21]	1.0%	0.8%
Kipyegen et al., 2012	29	285	0.10	[0.07; 0.14]	0.5%	0.8%
Kurniawan et al., 2009	3	318	0.01	[0.00; 0.03]	5.2%	0.8%
Lehman et al., 2013	3	201	0.01	[0.00; 0.04]	2.1%	0.8%
Mascarello et al., 2011	15	138	0.11	[0.06; 0.17]	0.2%	0.8%
Mathur et al., 2013	34	544	0.06	[0.04; 0.09]	1.4%	0.8%
Meamar et al., 2007	5	781	0.01	[0.00; 0.01]	18.8%	0.8%
Mehta et al., 2013	3	100	0.03	[0.01; 0.09]	0.5%	0.8%
Missaye et al., 2013	19	272	0.07	[0.04; 0.11]	0.6%	0.8%
Modjarrad et al., 2005	74	297	0.25	[0.20; 0.30]	0.2%	0.8%
Modjarrad et al., 2005	54	111	0.49	[0.39; 0.58]	0.1%	0.8%
Mulu et al., 2013	87	220	0.40	[0.33; 0.46]	0.1%	0.8%
Ndhlovu et al., 2007	72	156	0.46	[0.38; 0.54]	0.1%	0.8%
Nielsen et al., 2007	18	34	0.53	[0.35; 0.70]	0.0%	0.7%
Ojurongbe et al., 2011	16	96	0.17	[0.10; 0.26]	0.1%	0.8%
Pavie et al., 2012	4	143	0.03	[0.01; 0.07]	0.8%	0.8%
Pinlaor et al., 2005	37	78	0.47	[0.36; 0.59]	0.0%	0.8%
Ramakrishnan et al., 2007	7	80	0.09	[0.04; 0.17]	0.2%	0.8%
Ramana et al., 2010	32	452	0.07	[0.05; 0.10]	1.1%	0.8%
Raytekar et al., 2012	2	127	0.02	[0.00; 0.06]	1.3%	0.8%
Rodrigues et al., 2008	366	582	0.63	[0.59; 0.67]	0.4%	0.8%
Roka et al., 2012	242	260	0.93	[0.89; 0.96]	0.6%	0.8%
Roka et al., 2013	246	273	0.90	[0.86; 0.93]	0.5%	0.8%
Saksirisampant et al., 2009	2	90	0.02	[0.00; 0.08]	0.6%	0.8%
Sanyaolu et al., 2011	12	65	0.18	[0.10; 0.30]	0.1%	0.8%
Sherpa et al., 2010	4	106	0.04	[0.01; 0.09]	0.4%	0.8%
Smith et al., 2008	21	122	0.17	[0.11; 0.25]	0.1%	0.8%
Suryawanshi et al., 2012	12	100	0.12	[0.06; 0.20]	0.1%	0.8%
Talaat et al., 2008	40	632	0.06	[0.05; 0.09]	1.6%	0.8%
Tian et al., 2012	14	302	0.05	[0.03; 0.08]	1.0%	0.8%
Tian et al., 2013	5	79	0.06	[0.02; 0.14]	0.2%	0.8%
Tiwari et al., 2013	83	745	0.11	[0.09; 0.14]	1.2%	0.8%
Vyas et al., 2013	2	75	0.03	[0.00; 0.09]	0.4%	0.8%
Wanyiri et al., 2013	42	164	0.26	[0.19; 0.33]	0.1%	0.8%
Wumba et al., 2010	12	175	0.07	[0.04; 0.12]	0.4%	0.8%
Wumba et al., 2012	6	242	0.02	[0.01; 0.05]	1.5%	0.8%
Zeynudin et al., 2013	29	91	0.32	[0.22; 0.42]	0.1%	0.8%
Common effect model		17405	0.07	[0.07; 0.07]	71.1%	--
Random effects model			0.22	[0.16; 0.28]	--	53.0%
Heterogeneity: I^2 = 100%, τ^2 = 0.0558, p = 0						
**year = ≥2014**						
Abossie and Petros, 2015	55	97	0.57	[0.46; 0.67]	0.1%	0.8%
Adeleke et al., 2015	57	231	0.25	[0.19; 0.31]	0.2%	0.8%
Ahmed and Abhay, 2015	14	142	0.10	[0.05; 0.16]	0.2%	0.8%
Allam et al., 2014	68	141	0.48	[0.40; 0.57]	0.1%	0.8%
Barcelos et al., 2018	1	90	0.01	[0.00; 0.06]	1.3%	0.8%
Colombe et al., 2018	37	79	0.47	[0.36; 0.58]	0.0%	0.8%
Eshetu et al., 2017	36	223	0.16	[0.12; 0.22]	0.3%	0.8%
Feyisayo et al., 2014	4	105	0.04	[0.01; 0.09]	0.4%	0.8%
Gaurav and Atul, 2015	6	40	0.15	[0.06; 0.30]	0.0%	0.8%
Getaneh et al., 2018	15	220	0.07	[0.04; 0.11]	0.5%	0.8%
Gebretsadik et al., 2018	8	223	0.04	[0.02; 0.07]	1.0%	0.8%
Jegede et al., 2014	4	105	0.04	[0.01; 0.09]	0.4%	0.8%
Kroidl et al., 2016	69	187	0.37	[0.30; 0.44]	0.1%	0.8%
Lar et al., 2016	77	205	0.38	[0.31; 0.45]	0.1%	0.8%
Loffredo et al., 2015	16	31	0.52	[0.33; 0.70]	0.0%	0.7%
Marti et al., 2017	60	305	0.20	[0.15; 0.25]	0.3%	0.8%
Mazigo et al., 2016	28	50	0.56	[0.41; 0.70]	0.0%	0.7%
Minti et al., 2017	1	27	0.04	[0.00; 0.19]	0.1%	0.8%
Mkhize et al., 2017	31	95	0.33	[0.23; 0.43]	0.1%	0.8%
Morawski et al., 2017	65	202	0.32	[0.26; 0.39]	0.1%	0.8%
Mtapuri et al., 2014	1	29	0.03	[0.00; 0.18]	0.1%	0.8%
Nsagha et al., 2016	34	300	0.11	[0.08; 0.15]	0.5%	0.8%
Nuhu et al., 2014	8	205	0.04	[0.02; 0.08]	0.8%	0.8%
Orji et al., 2017	24	84	0.29	[0.19; 0.39]	0.1%	0.8%
Oyedeji et al., 2015	1	52	0.02	[0.00; 0.10]	0.4%	0.8%
Paboriboune et al., 2014	116	137	0.85	[0.78; 0.90]	0.2%	0.8%
Pennap et al., 2015	103	200	0.52	[0.44; 0.59]	0.1%	0.8%
Plumelle et al., 2015	55	445	0.12	[0.09; 0.16]	0.6%	0.8%
Ringo et al., 2015	13	83	0.16	[0.09; 0.25]	0.1%	0.8%
Samie et al., 2014	62	151	0.41	[0.33; 0.49]	0.1%	0.8%
Shimelis et al., 2016	54	491	0.11	[0.08; 0.14]	0.8%	0.8%
Sonali et al., 2014	2	250	0.01	[0.00; 0.03]	4.8%	0.8%
Talaat et al., 2015	28	80	0.35	[0.25; 0.46]	0.1%	0.8%
Tegegne et al., 2017	36	223	0.16	[0.12; 0.22]	0.3%	0.8%
Tigist et al., 2015	57	209	0.27	[0.21; 0.34]	0.2%	0.8%
Vouking et al., 2014	46	207	0.22	[0.17; 0.29]	0.2%	0.8%
Xiao et al., 2015	22	40	0.55	[0.38; 0.71]	0.0%	0.7%
Gebrecherkos et al., 2019	26	150	0.17	[0.12; 0.24]	0.2%	0.8%
Kaneshiro et al., 2019	87	252	0.35	[0.29; 0.41]	0.2%	0.8%
Sannella et al., 2019	23	155	0.15	[0.10; 0.21]	0.2%	0.8%
Alemayehu etal., 2020	74	383	0.19	[0.15; 0.24]	0.4%	0.8%
Belay et al., 2020	4	112	0.04	[0.01; 0.09]	0.5%	0.8%
Chhangte et al., 2020	8	250	0.03	[0.01; 0.06]	1.2%	0.8%
Lengongo et al., 2020	9	115	0.08	[0.04; 0.14]	0.2%	0.8%
McLellan et al., 2020	58	253	0.23	[0.18; 0.29]	0.2%	0.8%
Namaji et al., 2020	19	361	0.05	[0.03; 0.08]	1.1%	0.8%
Rabiu et al., 2020	6	197	0.03	[0.01; 0.07]	1.0%	0.8%
Botero et al., 2021	2	192	0.01	[0.00; 0.04]	2.9%	0.8%
Bayleyegn et al., 2021	32	255	0.13	[0.09; 0.17]	0.4%	0.8%
Betancourth et al., 2021	3	102	0.03	[0.01; 0.08]	0.5%	0.8%
Feleke et al., 2022	23	223	0.10	[0.07; 0.15]	0.4%	0.8%
Chandi et al., 2021	6	80	0.07	[0.03; 0.16]	0.2%	0.8%
Ajayi et al., 2021	10	375	0.03	[0.01; 0.05]	2.2%	0.8%
Talukder et al., 2021	45	201	0.22	[0.17; 0.29]	0.2%	0.8%
Wolday et al., 2021	184	751	0.25	[0.21; 0.28]	0.6%	0.8%
Purbey., 2021	13	75	0.17	[0.10; 0.28]	0.1%	0.8%
Uzairue et al., 2021	51	170	0.30	[0.23; 0.37]	0.1%	0.8%
PrayGod et al., 2022	176	1149	0.15	[0.13; 0.18]	1.4%	0.8%
Common effect model		11785	0.08	[0.08; 0.09]	28.9%	--
Random effects model			0.20	[0.16; 0.25]	--	47.0%
Heterogeneity: I^2 = 98%, τ^2 = 0.0314, p = 0						
**Common effect model**		**29190**	**0.07**	**[0.07; 0.08]**	**100.0%**	**--**
**Random effects model**			**0.21**	**[0.18; 0.25]**	**--**	**100.0%**

Heterogeneity: I^2 = 99%, τ^2 = 0.0443, p = 0

Test for subgroup differences (fixed effect): χ^2_1 = 17.99, df = 1 (p < 0.01)

Test for subgroup differences (random effects): χ^2_1 = 0.24, df = 1 (p = 0.63)

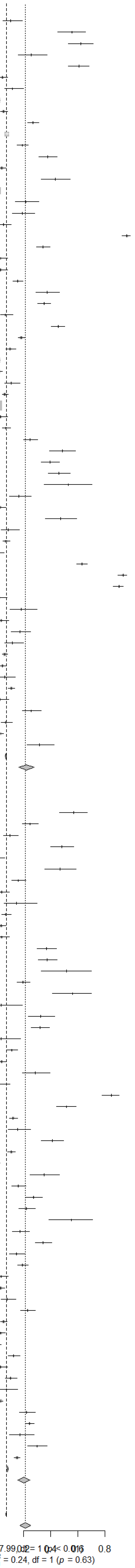

Figure S7B:

Subgroup analysis of the prevalence of protozoan infections in people living with viruses by publication year

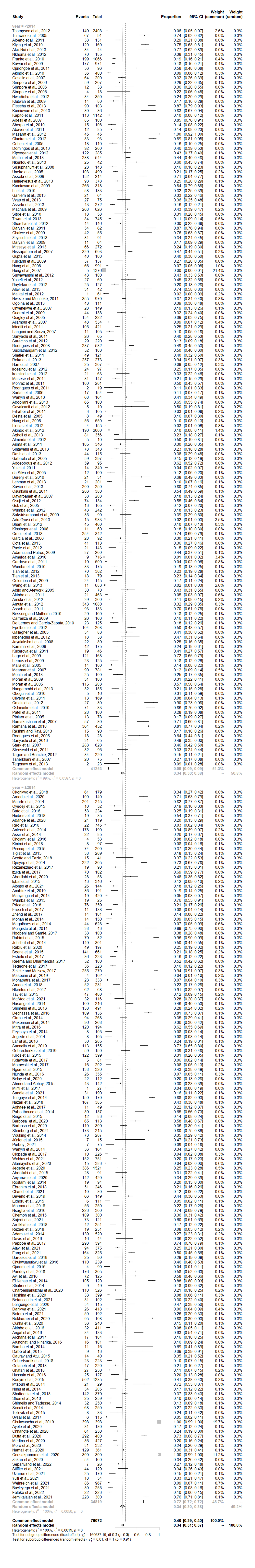

Figure S8A: Subgroup analysis of the prevalence of helminth infections in people living with HIV by income level

Study	Events	Total	Proportion	95%-CI	Weight (common)	Weight (random)
**Income_Level = Middle**						
Abaver et al., 2011	9	85	0.11	[0.05; 0.19]	0.1%	0.9%
Adams et al., 2006	103	170	0.61	[0.53; 0.68]	0.1%	0.9%
Adeleke et al., 2015	57	231	0.25	[0.19; 0.31]	0.2%	0.9%
Adesij et al. 2007	12	100	0.12	[0.06; 0.20]	0.1%	0.9%
Agholi et al., 2013	4	356	0.01	[0.00; 0.03]	5.0%	0.9%
Ahmed and Abhay, 2015	14	142	0.10	[0.05; 0.16]	0.2%	0.9%
Akinbo and Omoregie, 2011	15	285	0.05	[0.03; 0.09]	0.9%	0.9%
Akinbo et al., 2011	125	463	0.27	[0.23; 0.31]	0.4%	0.9%
Akinbo et al., 2012	156	2000	0.08	[0.07; 0.09]	4.3%	0.9%
Asma et al., 2011	66	346	0.19	[0.15; 0.24]	0.3%	0.9%
Babatunde et al., 2010	39	90	0.43	[0.33; 0.54]	0.1%	0.8%
Barcelos et al., 2018	1	90	0.01	[0.00; 0.06]	1.3%	0.9%
Cardoso et al., 2011	4	500	0.01	[0.00; 0.02]	9.8%	0.9%
Da Silva et al., 2005	19	100	0.19	[0.12; 0.28]	0.1%	0.8%
Dash et al., 2013	6	115	0.05	[0.02; 0.11]	0.4%	0.9%
Eshetu et al., 2017	36	223	0.16	[0.12; 0.22]	0.3%	0.9%
Feyisayo et al., 2014	4	105	0.04	[0.01; 0.09]	0.4%	0.9%
Gaurav and Atul, 2015	6	40	0.15	[0.06; 0.30]	0.0%	0.8%
Gupta et al., 2013	3	100	0.03	[0.01; 0.09]	0.5%	0.9%
Jegede et al., 2014	4	105	0.04	[0.01; 0.09]	0.4%	0.9%
Kulkarni et al., 2009	9	137	0.07	[0.03; 0.12]	0.3%	0.9%
Kipyegen et al., 2012	29	285	0.10	[0.07; 0.14]	0.5%	0.9%
Kurniawan et al., 2009	3	318	0.01	[0.00; 0.03]	5.3%	0.9%
Lar et al., 2016	77	205	0.38	[0.31; 0.45]	0.1%	0.9%
Lehman et al., 2013	3	201	0.01	[0.00; 0.04]	2.1%	0.9%
Mathur et al., 2013	34	544	0.06	[0.04; 0.09]	1.4%	0.9%
Meamar et al., 2007	5	781	0.01	[0.00; 0.01]	19.0%	0.9%
Mehta et al., 2013	3	100	0.03	[0.01; 0.09]	0.5%	0.9%
Minti et al., 2017	1	27	0.04	[0.00; 0.19]	0.1%	0.9%
Mkhize et al., 2017	31	95	0.33	[0.23; 0.43]	0.1%	0.8%
Nsagha et al., 2016	34	300	0.11	[0.08; 0.15]	0.5%	0.9%
Nuhu et al., 2014	8	205	0.04	[0.02; 0.08]	0.8%	0.9%
Ojurongbe et al., 2011	16	96	0.17	[0.10; 0.26]	0.1%	0.9%
Orji et al., 2017	24	84	0.29	[0.19; 0.39]	0.1%	0.8%
Oyedeji et al., 2015	1	52	0.02	[0.00; 0.10]	0.4%	0.9%
Paboriboune et al., 2014	116	137	0.85	[0.78; 0.90]	0.2%	0.9%
Pennap et al., 2015	103	200	0.52	[0.44; 0.59]	0.1%	0.9%
Pinlaor et al., 2005	37	78	0.47	[0.36; 0.59]	0.0%	0.8%
Ramakrishnan et al., 2007	7	80	0.09	[0.04; 0.17]	0.2%	0.9%
Ramana et al., 2010	32	452	0.07	[0.05; 0.10]	1.1%	0.9%
Raytekar et al., 2012	2	127	0.02	[0.00; 0.06]	1.3%	0.9%
Rodrigues et al., 2008	366	582	0.63	[0.59; 0.67]	0.4%	0.9%
Saksirisampant et al., 2009	2	90	0.02	[0.00; 0.08]	0.6%	0.9%
Samie et al., 2014	62	151	0.41	[0.33; 0.49]	0.1%	0.8%
Sanyaolu et al., 2011	12	65	0.18	[0.10; 0.30]	0.1%	0.8%
Sherpa et al., 2010	4	106	0.04	[0.01; 0.09]	0.5%	0.9%
Smith et al., 2008	21	122	0.17	[0.11; 0.25]	0.1%	0.9%
Sonali et al., 2014	2	250	0.01	[0.00; 0.03]	4.9%	0.9%
Suryawanshi et al., 2012	12	100	0.12	[0.06; 0.20]	0.1%	0.9%
Talaat et al., 2015	28	80	0.35	[0.25; 0.46]	0.1%	0.8%
Talaat et al., 2008	40	632	0.06	[0.05; 0.09]	1.7%	0.9%
Tian et al., 2012	14	302	0.05	[0.03; 0.08]	1.1%	0.9%
Tian et al., 2013	5	79	0.06	[0.02; 0.14]	0.2%	0.9%
Vouking et al., 2014	46	207	0.22	[0.17; 0.29]	0.2%	0.9%
Vyas et al., 2013	2	75	0.03	[0.00; 0.09]	0.4%	0.9%
Wanyiri et al., 2013	42	164	0.26	[0.19; 0.33]	0.1%	0.9%
Kaneshiro et al., 2019	87	252	0.35	[0.29; 0.41]	0.2%	0.9%
Sannella et al., 2019	23	155	0.15	[0.10; 0.21]	0.2%	0.9%
Chhangte et al., 2020	8	250	0.03	[0.01; 0.06]	1.3%	0.9%
Lengongo et al., 2020	9	115	0.08	[0.04; 0.14]	0.2%	0.9%
Namaji et al., 2020	19	361	0.05	[0.03; 0.08]	1.1%	0.9%
Rabiu et al., 2020	6	197	0.03	[0.01; 0.07]	1.0%	0.9%
Botero et al., 2021	2	192	0.01	[0.00; 0.04]	2.9%	0.9%
Chandi et al., 2021	6	80	0.07	[0.03; 0.16]	0.2%	0.9%
Ajayi et al., 2021	10	375	0.03	[0.01; 0.05]	2.2%	0.9%
Purbey., 2021	13	75	0.17	[0.10; 0.28]	0.1%	0.8%
Common effect model		15207	0.04	[0.03; 0.04]	79.3%	--
Random effects model			0.15	[0.11; 0.19]	--	57.0%
Heterogeneity: $I^2 = 98\%$, $\tau^2 = 0.0294$, $p = 0$						
**Income_Level = Low**						
Abilo and Afework, 2005	18	70	0.26	[0.16; 0.38]	0.1%	0.8%
Abossie and Petros, 2015	55	97	0.57	[0.46; 0.67]	0.1%	0.8%
Adamu and Petros, 2009	9	200	0.04	[0.02; 0.08]	0.7%	0.9%
Assefa et al., 2009	81	214	0.38	[0.31; 0.45]	0.1%	0.9%
Assefa et al., 2013	11	272	0.04	[0.02; 0.07]	1.1%	0.9%
Colombe et al., 2018	37	79	0.47	[0.36; 0.58]	0.0%	0.8%
Erikstrup et al., 2008	190	198	0.96	[0.92; 0.98]	0.8%	0.9%
Fekadu et al., 2013	118	343	0.34	[0.29; 0.40]	0.2%	0.9%
Getaneh et al., 2018	15	220	0.07	[0.04; 0.11]	0.5%	0.9%
Gebretsadik et al., 2018	8	223	0.04	[0.02; 0.07]	1.0%	0.9%
Haileeyesus et al., 2013	59	378	0.16	[0.12; 0.20]	0.4%	0.9%
Idindili et al., 2011	148	421	0.35	[0.31; 0.40]	0.3%	0.9%
Kallestrup et al., 2005	185	407	0.45	[0.41; 0.50]	0.3%	0.9%
Kawai et al., 2009	178	971	0.18	[0.16; 0.21]	1.0%	0.9%
Kroidl et al., 2016	69	187	0.37	[0.30; 0.44]	0.1%	0.9%
Marti et al., 2017	60	305	0.20	[0.15; 0.25]	0.3%	0.9%
Mazigo et al., 2016	28	50	0.56	[0.41; 0.70]	0.0%	0.8%
Missaye et al., 2013	19	272	0.07	[0.04; 0.11]	0.6%	0.9%
Modjarrad et al., 2005	74	297	0.25	[0.20; 0.30]	0.2%	0.9%
Modjarrad et al., 2005	54	111	0.49	[0.39; 0.58]	0.1%	0.8%
Morawski et al., 2017	65	202	0.32	[0.26; 0.39]	0.1%	0.9%
Mtapuri et al., 2014	1	29	0.03	[0.00; 0.18]	0.1%	0.9%
Mulu et al., 2013	87	220	0.40	[0.33; 0.46]	0.1%	0.9%
Ndhlovu et al., 2007	72	156	0.46	[0.38; 0.54]	0.1%	0.8%
Nielsen et al., 2007	18	34	0.53	[0.35; 0.70]	0.0%	0.7%
Ringo et al., 2015	13	83	0.16	[0.09; 0.25]	0.1%	0.8%
Roka et al., 2012	242	260	0.93	[0.89; 0.96]	0.6%	0.9%
Roka et al., 2013	246	273	0.90	[0.86; 0.93]	0.5%	0.9%
Shimelis et al., 2016	54	491	0.11	[0.08; 0.14]	0.8%	0.9%
Tegegne et al., 2017	36	223	0.16	[0.12; 0.22]	0.3%	0.9%
Tigist et al., 2015	57	209	0.27	[0.21; 0.34]	0.2%	0.9%
Tiwari et al., 2013	83	745	0.11	[0.09; 0.14]	1.2%	0.9%
Wumba et al., 2010	12	175	0.07	[0.04; 0.12]	0.4%	0.9%
Wumba et al., 2012	6	242	0.02	[0.01; 0.05]	1.6%	0.9%
Zeynudin et al., 2013	29	91	0.32	[0.22; 0.42]	0.1%	0.8%
Gebrecherkos et al., 2019	26	150	0.17	[0.12; 0.24]	0.2%	0.9%
Alemayehu etal., 2020	74	383	0.19	[0.15; 0.24]	0.4%	0.9%
Belay et al., 2020	4	112	0.04	[0.01; 0.09]	0.5%	0.9%
Bayleyegn et al., 2021	32	255	0.13	[0.09; 0.17]	0.4%	0.9%
Betancourth et al., 2021	3	102	0.03	[0.01; 0.08]	0.6%	0.9%
Feleke et al., 2022	23	223	0.10	[0.07; 0.15]	0.4%	0.9%
Uzairue et al., 2021	51	170	0.30	[0.23; 0.37]	0.1%	0.9%
PrayGod et al., 2022	176	1149	0.15	[0.13; 0.18]	1.4%	0.9%
Common effect model		11292	0.22	[0.21; 0.22]	18.1%	--
Random effects model			0.28	[0.21; 0.35]	--	36.9%
Heterogeneity: $I^2 = 100\%$, $\tau^2 = 0.0577$, $p = 0$						
**Income_Level = High**						
Costiniuk et al., 2012	21	97	0.22	[0.14; 0.31]	0.1%	0.8%
Guk et al., 2005	3	105	0.03	[0.01; 0.08]	0.6%	0.9%
Hochberg et al., 2011	48	128	0.38	[0.29; 0.46]	0.1%	0.8%
Mascarello et al., 2011	15	138	0.11	[0.06; 0.17]	0.2%	0.9%
Pavie et al., 2012	4	143	0.03	[0.01; 0.07]	0.8%	0.9%
Plumelle et al., 2015	55	445	0.12	[0.09; 0.16]	0.6%	0.9%
McLellan et al., 2020	58	253	0.23	[0.18; 0.29]	0.2%	0.9%
Common effect model		1309	0.09	[0.08; 0.11]	2.7%	--
Random effects model			0.15	[0.06; 0.24]	--	6.0%
Heterogeneity: $I^2 = 95\%$, $\tau^2 = 0.0141$, $p < 0.01$						
**Common effect model**		**27808**	**0.07**	**[0.07; 0.07]**	**100.0%**	**--**
**Random effects model**			**0.20**	**[0.16; 0.24]**	**--**	**100.0%**

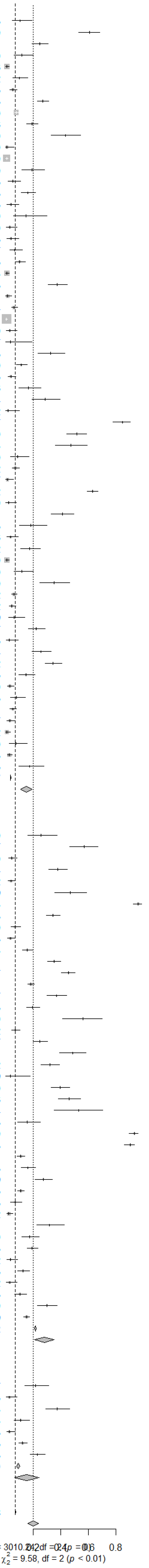

Heterogeneity: $I^2 = 99\%$, $\tau^2 = 0.0423$, $p = 0$

Test for subgroup differences (fixed effect): $\chi^2_2 = 3010.24$, df = 2 ($p = 0$)

Test for subgroup differences (random effects): $\chi^2_2 = 9.58$, df = 2 ($p < 0.01$)

Figure S8B: Subgroup analysis of the prevalence of protozoan infections in people living with HIV by income level

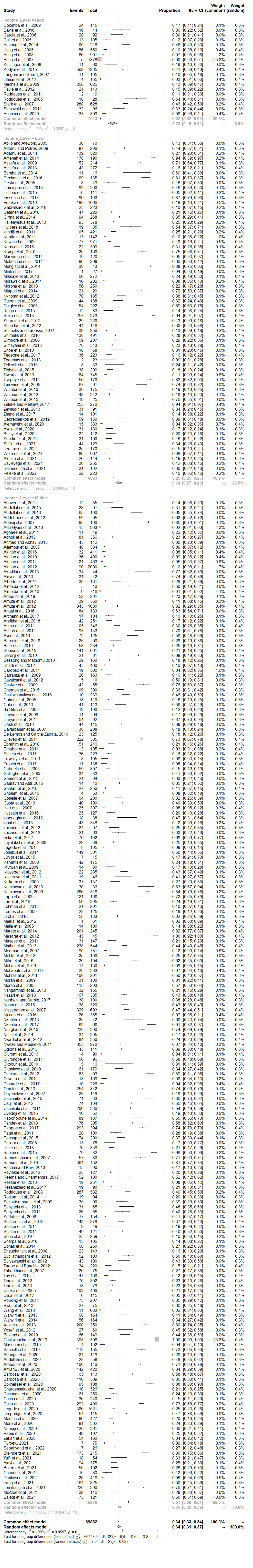

Figure S9A: Subgroup analysis of the prevalence of helminth infections in people living with HIV by age

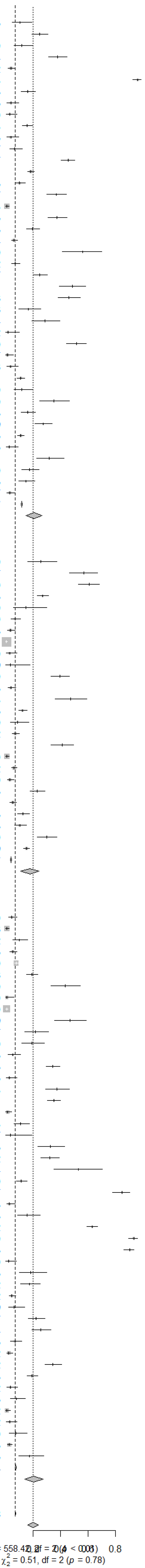

Study	Events	Total	Proportion	95%-CI	Weight (common)	Weight (random)
**Age = <35**						
Abaver et al., 2011	9	85	0.11	[0.05; 0.19]	0.1%	0.9%
Adeleke et al., 2015	57	231	0.25	[0.19; 0.31]	0.2%	0.9%
Adesij et al. 2007	12	100	0.12	[0.06; 0.20]	0.1%	0.9%
Assefa et al., 2009	81	214	0.38	[0.31; 0.45]	0.1%	0.9%
Assefa et al., 2013	11	272	0.04	[0.02; 0.07]	1.1%	0.9%
Erikstrup et al., 2008	190	198	0.96	[0.92; 0.98]	0.8%	0.9%
Eshetu et al., 2017	36	223	0.16	[0.12; 0.22]	0.3%	0.9%
Feyisayo et al., 2014	4	105	0.04	[0.01; 0.09]	0.4%	0.9%
Gupta et al., 2013	3	100	0.03	[0.01; 0.09]	0.5%	0.9%
Haileeyesus et al., 2013	59	378	0.16	[0.12; 0.20]	0.4%	0.9%
Jegede et al., 2014	4	105	0.04	[0.01; 0.09]	0.4%	0.9%
Kulkarni et al., 2009	9	137	0.07	[0.03; 0.12]	0.3%	0.9%
Kallestrup et al., 2005	185	407	0.45	[0.41; 0.50]	0.3%	0.9%
Kawai et al., 2009	178	971	0.18	[0.16; 0.21]	1.0%	0.9%
Kipyegen et al., 2012	29	285	0.10	[0.07; 0.14]	0.5%	0.9%
Kroidl et al., 2016	69	187	0.37	[0.30; 0.44]	0.1%	0.9%
Kurniawan et al., 2009	3	318	0.01	[0.00; 0.03]	5.3%	0.9%
Lar et al., 2016	77	205	0.38	[0.31; 0.45]	0.1%	0.9%
Marti et al., 2017	60	305	0.20	[0.15; 0.25]	0.3%	0.9%
Mathur et al., 2013	34	544	0.06	[0.04; 0.09]	1.4%	0.9%
Mazigo et al., 2016	28	50	0.56	[0.41; 0.70]	0.0%	0.8%
Missaye et al., 2013	19	272	0.07	[0.04; 0.11]	0.6%	0.9%
Modjarrad et al., 2005	74	297	0.25	[0.20; 0.30]	0.2%	0.9%
Modjarrad et al., 2005	54	111	0.49	[0.39; 0.58]	0.1%	0.8%
Ndhlovu et al., 2007	72	156	0.46	[0.38; 0.54]	0.1%	0.8%
Ojurongbe et al., 2011	16	96	0.17	[0.10; 0.26]	0.1%	0.9%
Orji et al., 2017	24	84	0.29	[0.19; 0.39]	0.1%	0.8%
Oyedeji et al., 2015	1	52	0.02	[0.00; 0.10]	0.4%	0.9%
Pennap et al., 2015	103	200	0.52	[0.44; 0.59]	0.1%	0.9%
Raytekar et al., 2012	2	127	0.02	[0.00; 0.06]	1.3%	0.9%
Sherpa et al., 2010	4	106	0.04	[0.01; 0.09]	0.5%	0.9%
Shimelis et al., 2016	54	491	0.11	[0.08; 0.14]	0.8%	0.9%
Suryawanshi et al., 2012	12	100	0.12	[0.06; 0.20]	0.1%	0.9%
Talaat et al., 2015	28	80	0.35	[0.25; 0.46]	0.1%	0.8%
Tegegne et al., 2017	36	223	0.16	[0.12; 0.22]	0.3%	0.9%
Tigist et al., 2015	57	209	0.27	[0.21; 0.34]	0.2%	0.9%
Tiwari et al., 2013	83	745	0.11	[0.09; 0.14]	1.2%	0.9%
Vyas et al., 2013	2	75	0.03	[0.00; 0.09]	0.4%	0.9%
Zeynudin et al., 2013	29	91	0.32	[0.22; 0.42]	0.1%	0.8%
Gebrecherkos et al., 2019	26	150	0.17	[0.12; 0.24]	0.2%	0.9%
Sannella et al., 2019	23	155	0.15	[0.10; 0.21]	0.2%	0.9%
Rabiu et al., 2020	6	197	0.03	[0.01; 0.07]	1.0%	0.9%
**Common effect model**		9437	0.12	[0.11; 0.12]	22.0%	--
**Random effects model**			0.21	[0.15; 0.27]	--	36.2%
Heterogeneity: I^2 = 99%, τ^2 = 0.0373, p = 0						
**Age = N/A**						
Abilo and Afework, 2005	18	70	0.26	[0.16; 0.38]	0.1%	0.8%
Abossie and Petros, 2015	55	97	0.57	[0.46; 0.67]	0.1%	0.8%
Adams et al., 2006	103	170	0.61	[0.53; 0.68]	0.1%	0.9%
Akinbo et al., 2011	125	463	0.27	[0.23; 0.31]	0.4%	0.9%
Gaurav and Atul, 2015	6	40	0.15	[0.06; 0.30]	0.0%	0.8%
Getaneh et al., 2018	15	220	0.07	[0.04; 0.11]	0.5%	0.9%
Gebretsadik et al., 2018	8	223	0.04	[0.02; 0.07]	1.0%	0.9%
Meamar et al., 2007	5	781	0.01	[0.00; 0.01]	19.0%	0.9%
Mehta et al., 2013	3	100	0.03	[0.01; 0.09]	0.5%	0.9%
Mtapuri et al., 2014	1	29	0.03	[0.00; 0.18]	0.1%	0.9%
Mulu et al., 2013	87	220	0.40	[0.33; 0.46]	0.1%	0.9%
Nuhu et al., 2014	8	205	0.04	[0.02; 0.08]	0.8%	0.9%
Pinlaor et al., 2005	37	78	0.47	[0.36; 0.59]	0.0%	0.8%
Plumelle et al., 2015	55	445	0.12	[0.09; 0.16]	0.6%	0.9%
Ramakrishnan et al., 2007	7	80	0.09	[0.04; 0.17]	0.2%	0.9%
Ramana et al., 2010	32	452	0.07	[0.05; 0.10]	1.1%	0.9%
Samie et al., 2014	62	151	0.41	[0.33; 0.49]	0.1%	0.8%
Sonali et al., 2014	2	250	0.01	[0.00; 0.03]	4.9%	0.9%
Talaat et al., 2008	40	632	0.06	[0.05; 0.09]	1.7%	0.9%
Chhangte et al., 2020	8	250	0.03	[0.01; 0.06]	1.3%	0.9%
McLellan et al., 2020	58	253	0.23	[0.18; 0.29]	0.2%	0.9%
Namaji et al., 2020	19	361	0.05	[0.03; 0.08]	1.1%	0.9%
Bayleyegn et al., 2021	32	255	0.13	[0.09; 0.17]	0.4%	0.9%
Feleke et al., 2022	23	223	0.10	[0.07; 0.15]	0.4%	0.9%
Uzairue et al., 2021	51	170	0.30	[0.23; 0.37]	0.1%	0.9%
PrayGod et al., 2022	176	1149	0.15	[0.13; 0.18]	1.4%	0.9%
**Common effect model**		7367	0.04	[0.03; 0.04]	36.3%	--
**Random effects model**			0.18	[0.11; 0.24]	--	22.4%
Heterogeneity: I^2 = 98%, τ^2 = 0.0296, p < 0.01						
**Age = ≥35**						
Adamu and Petros, 2009	9	200	0.04	[0.02; 0.08]	0.7%	0.9%
Agholi et al., 2013	4	356	0.01	[0.00; 0.03]	5.0%	0.9%
Ahmed and Abhay, 2015	14	142	0.10	[0.05; 0.16]	0.2%	0.9%
Akinbo and Omoregie, 2011	15	285	0.05	[0.03; 0.09]	0.9%	0.9%
Akinbo et al., 2012	156	2000	0.08	[0.07; 0.09]	4.3%	0.9%
Asma et al., 2011	66	346	0.19	[0.15; 0.24]	0.3%	0.9%
Babatunde et al., 2010	39	90	0.43	[0.33; 0.54]	0.1%	0.8%
Barcelos et al., 2018	1	90	0.01	[0.00; 0.06]	1.3%	0.9%
Cardoso et al., 2011	4	500	0.01	[0.00; 0.02]	9.8%	0.9%
Colombe et al., 2018	37	79	0.47	[0.36; 0.58]	0.0%	0.8%
Costiniuk et al., 2012	21	97	0.22	[0.14; 0.31]	0.1%	0.8%
Da Silva et al., 2005	19	100	0.19	[0.12; 0.28]	0.1%	0.8%
Dash et al., 2013	6	115	0.05	[0.02; 0.11]	0.4%	0.9%
Fekadu et al., 2013	118	343	0.34	[0.29; 0.40]	0.2%	0.9%
Guk et al., 2005	3	105	0.03	[0.01; 0.08]	0.6%	0.9%
Hochberg et al., 2011	48	128	0.38	[0.29; 0.46]	0.1%	0.8%
Idindili et al., 2011	148	421	0.35	[0.31; 0.40]	0.3%	0.9%
Lehman et al., 2013	3	201	0.01	[0.00; 0.04]	2.1%	0.9%
Mascarello et al., 2011	15	138	0.11	[0.06; 0.17]	0.2%	0.9%
Minti et al., 2017	1	27	0.04	[0.00; 0.19]	0.1%	0.9%
Mkhize et al., 2017	31	95	0.33	[0.23; 0.43]	0.1%	0.8%
Morawski et al., 2017	65	202	0.32	[0.26; 0.39]	0.1%	0.9%
Nielsen et al., 2007	18	34	0.53	[0.35; 0.70]	0.0%	0.7%
Nsagha et al., 2016	34	300	0.11	[0.08; 0.15]	0.5%	0.9%
Paboriboune et al., 2014	116	137	0.85	[0.78; 0.90]	0.2%	0.9%
Pavie et al., 2012	4	143	0.03	[0.01; 0.07]	0.8%	0.9%
Ringo et al., 2015	13	83	0.16	[0.09; 0.25]	0.1%	0.8%
Rodrigues et al., 2008	366	582	0.63	[0.59; 0.67]	0.4%	0.9%
Roka et al., 2012	242	260	0.93	[0.89; 0.96]	0.6%	0.9%
Roka et al., 2013	246	273	0.90	[0.86; 0.93]	0.5%	0.9%
Saksirisampant et al., 2009	2	90	0.02	[0.00; 0.08]	0.6%	0.9%
Sanyaolu et al., 2011	12	65	0.18	[0.10; 0.30]	0.1%	0.8%
Smith et al., 2008	21	122	0.17	[0.11; 0.25]	0.1%	0.9%
Tian et al., 2012	14	302	0.05	[0.03; 0.08]	1.1%	0.9%
Tian et al., 2013	5	79	0.06	[0.02; 0.14]	0.2%	0.9%
Vouking et al., 2014	46	207	0.22	[0.17; 0.29]	0.2%	0.9%
Wanyiri et al., 2013	42	164	0.26	[0.19; 0.33]	0.1%	0.9%
Wumba et al., 2010	12	175	0.07	[0.04; 0.12]	0.4%	0.9%
Wumba et al., 2012	6	242	0.02	[0.01; 0.05]	1.6%	0.9%
Kaneshiro et al., 2019	87	252	0.35	[0.29; 0.41]	0.2%	0.9%
Alemayehu etal., 2020	74	383	0.19	[0.15; 0.24]	0.4%	0.9%
Belay et al., 2020	4	112	0.04	[0.01; 0.09]	0.5%	0.9%
Lengongo et al., 2020	9	115	0.08	[0.04; 0.14]	0.2%	0.9%
Botero et al., 2021	2	192	0.01	[0.00; 0.04]	2.9%	0.9%
Betancourth et al., 2021	3	102	0.03	[0.01; 0.08]	0.6%	0.9%
Chandi et al., 2021	6	80	0.07	[0.03; 0.16]	0.2%	0.9%
Ajayi et al., 2021	10	375	0.03	[0.01; 0.05]	2.2%	0.9%
Purbey., 2021	13	75	0.17	[0.10; 0.28]	0.1%	0.8%
**Common effect model**		11004	0.08	[0.07; 0.08]	41.8%	--
**Random effects model**			0.21	[0.14; 0.27]	--	41.4%
Heterogeneity: I^2 = 99%, τ^2 = 0.0547, p = 0						
**Common effect model**		**27808**	**0.07**	**[0.07; 0.07]**	**100.0%**	**--**
**Random effects model**			**0.20**	**[0.16; 0.24]**	**--**	**100.0%**

Heterogeneity: I^2 = 99%, τ^2 = 0.0423, p = 0
Test for subgroup differences (fixed effect): χ^2_2 = 558.4[illegible], df = 2 (p < 0.0[illegible])
Test for subgroup differences (random effects): χ^2_2 = 0.51, df = 2 (p = 0.78)

Figure S9B :Subgroup analysis of the prevalence of protozoan infections in people living with HIV by age

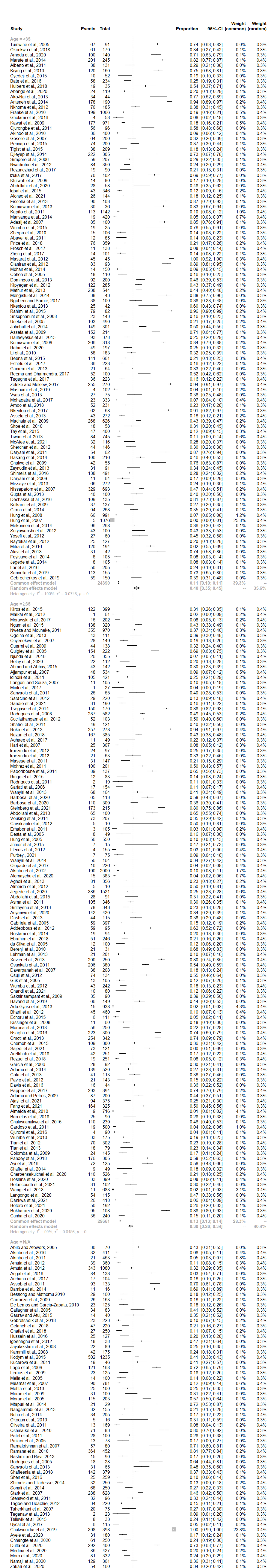

Figure S10A: Subgroup analysis of the prevalence of helminth infections in people living with HIV by gender

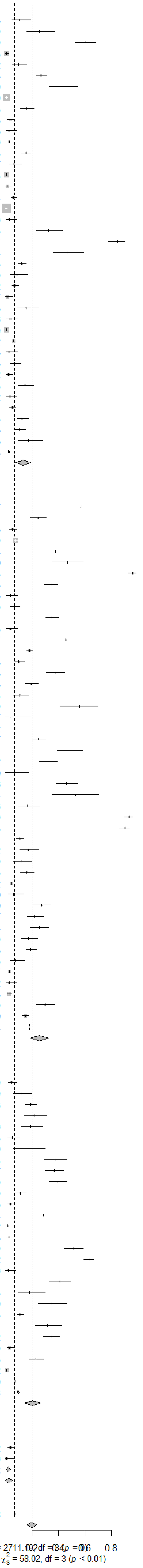

Study	Events	Total	Proportion	95%-CI	Weight (common)	Weight (random)
**Gender_at_Risk = N/A**						
Abaver et al., 2011	9	85	0.11	[0.05; 0.19]	0.1%	0.9%
Abilo and Afework, 2005	18	70	0.26	[0.16; 0.38]	0.1%	0.8%
Adams et al., 2006	103	170	0.61	[0.53; 0.68]	0.1%	0.9%
Agholi et al., 2013	4	356	0.01	[0.00; 0.03]	5.0%	0.9%
Ahmed and Abbay, 2015	14	142	0.10	[0.05; 0.16]	0.2%	0.9%
Akinbo et al., 2011	125	463	0.27	[0.23; 0.31]	0.4%	0.9%
Babatunde et al., 2010	39	90	0.43	[0.33; 0.54]	0.1%	0.8%
Cardoso et al., 2011	4	500	0.01	[0.00; 0.02]	9.8%	0.9%
Eshetu et al., 2017	36	223	0.16	[0.12; 0.22]	0.3%	0.9%
Gebretsadik et al., 2018	8	223	0.04	[0.02; 0.07]	1.0%	0.9%
Guk et al., 2005	3	105	0.03	[0.01; 0.08]	0.6%	0.9%
Gupta et al., 2013	3	100	0.03	[0.01; 0.09]	0.5%	0.9%
Haileeyesus et al., 2013	59	378	0.16	[0.12; 0.20]	0.4%	0.9%
Kulkarni et al., 2009	9	137	0.07	[0.03; 0.12]	0.3%	0.9%
Kurniawan et al., 2009	3	318	0.01	[0.00; 0.03]	5.3%	0.9%
Lehman et al., 2013	3	201	0.01	[0.00; 0.04]	2.1%	0.9%
Mathur et al., 2013	34	544	0.06	[0.04; 0.09]	1.4%	0.9%
Meamar et al., 2007	5	781	0.01	[0.00; 0.01]	19.0%	0.9%
Mehta et al., 2013	3	100	0.03	[0.01; 0.09]	0.5%	0.9%
Mkhize et al., 2017	31	95	0.33	[0.23; 0.43]	0.1%	0.8%
Paboriboune et al., 2014	116	137	0.85	[0.78; 0.90]	0.2%	0.9%
Pinlaor et al., 2005	37	78	0.47	[0.36; 0.59]	0.0%	0.8%
Plumelle et al., 2015	55	445	0.12	[0.09; 0.16]	0.6%	0.9%
Ramakrishnan et al., 2007	7	80	0.09	[0.04; 0.17]	0.2%	0.9%
Ramana et al., 2010	32	452	0.07	[0.05; 0.10]	1.1%	0.9%
Raytekar et al., 2012	2	127	0.02	[0.00; 0.06]	1.3%	0.9%
Ringo et al., 2015	13	83	0.16	[0.09; 0.25]	0.1%	0.8%
Sherpa et al., 2010	4	106	0.04	[0.01; 0.09]	0.5%	0.9%
Sonali et al., 2014	2	250	0.01	[0.00; 0.03]	4.9%	0.9%
Talaat et al., 2008	40	632	0.06	[0.05; 0.09]	1.7%	0.9%
Vyas et al., 2013	2	75	0.03	[0.00; 0.09]	0.4%	0.9%
Wumba et al., 2010	12	175	0.07	[0.04; 0.12]	0.4%	0.9%
Wumba et al., 2012	6	242	0.02	[0.01; 0.05]	1.6%	0.9%
Sannella et al., 2019	23	155	0.15	[0.10; 0.21]	0.2%	0.9%
Belay et al., 2020	4	112	0.04	[0.01; 0.09]	0.5%	0.9%
Namaji et al., 2020	19	361	0.05	[0.03; 0.08]	1.1%	0.9%
Bayleyegn et al., 2021	32	255	0.13	[0.09; 0.17]	0.4%	0.9%
Feleke et al., 2022	23	223	0.10	[0.07; 0.15]	0.4%	0.9%
Purbey., 2021	13	75	0.17	[0.10; 0.28]	0.1%	0.8%
**Common effect model**		9144	0.03	[0.02; 0.03]	62.9%	--
**Random effects model**			0.13	[0.08; 0.19]	--	33.8%
Heterogeneity: $I^2 = 98\%$, $\tau^2 = 0.0307$, $p < 0.01$						
**Gender_at_Risk = Female**						
Abossie and Petros, 2015	55	97	0.57	[0.46; 0.67]	0.1%	0.8%
Adeleke et al., 2015	57	231	0.25	[0.19; 0.31]	0.2%	0.9%
Akinbo and Omoregie, 2011	15	285	0.05	[0.03; 0.09]	0.9%	0.9%
Akinbo et al., 2012	156	2000	0.08	[0.07; 0.09]	4.3%	0.9%
Assefa et al., 2009	81	214	0.38	[0.31; 0.45]	0.1%	0.9%
Colombe et al., 2018	37	79	0.47	[0.36; 0.58]	0.0%	0.8%
Erikstrup et al., 2008	190	198	0.96	[0.92; 0.98]	0.8%	0.9%
Fekadu et al., 2013	118	343	0.34	[0.29; 0.40]	0.2%	0.9%
Feyisayo et al., 2014	4	105	0.04	[0.01; 0.09]	0.4%	0.9%
Getaneh et al., 2018	15	220	0.07	[0.04; 0.11]	0.5%	0.9%
Idindili et al., 2011	148	421	0.35	[0.31; 0.40]	0.3%	0.9%
Jegede et al., 2014	4	105	0.04	[0.01; 0.09]	0.4%	0.9%
Kallestrup et al., 2005	185	407	0.45	[0.41; 0.50]	0.3%	0.9%
Kawai et al., 2009	178	971	0.18	[0.16; 0.21]	1.0%	0.9%
Kipyegen et al., 2012	29	285	0.10	[0.07; 0.14]	0.5%	0.9%
Lar et al., 2016	77	205	0.38	[0.31; 0.45]	0.1%	0.9%
Marti et al., 2017	60	305	0.20	[0.15; 0.25]	0.3%	0.9%
Mascarello et al., 2011	15	138	0.11	[0.06; 0.17]	0.2%	0.9%
Mazigo et al., 2016	28	50	0.56	[0.41; 0.70]	0.0%	0.8%
Minti et al., 2017	1	27	0.04	[0.00; 0.19]	0.1%	0.9%
Missaye et al., 2013	19	272	0.07	[0.04; 0.11]	0.6%	0.9%
Modjarrad et al., 2005	74	297	0.25	[0.20; 0.30]	0.2%	0.9%
Modjarrad et al., 2005	54	111	0.49	[0.39; 0.58]	0.1%	0.8%
Morawski et al., 2017	65	202	0.32	[0.26; 0.39]	0.1%	0.9%
Mtapuri et al., 2014	1	29	0.03	[0.00; 0.18]	0.1%	0.9%
Ndhlovu et al., 2007	72	156	0.46	[0.38; 0.54]	0.1%	0.8%
Nielsen et al., 2007	18	34	0.53	[0.35; 0.70]	0.0%	0.7%
Ojurongbe et al., 2011	16	96	0.17	[0.10; 0.26]	0.1%	0.9%
Roka et al., 2012	242	260	0.93	[0.89; 0.96]	0.6%	0.9%
Roka et al., 2013	246	273	0.90	[0.86; 0.93]	0.5%	0.9%
Shimelis et al., 2016	54	491	0.11	[0.08; 0.14]	0.8%	0.9%
Smith et al., 2008	21	122	0.17	[0.11; 0.25]	0.1%	0.9%
Suryawanshi et al., 2012	12	100	0.12	[0.06; 0.20]	0.1%	0.9%
Tegegne et al., 2017	36	223	0.16	[0.12; 0.22]	0.3%	0.9%
Tian et al., 2012	14	302	0.05	[0.03; 0.08]	1.1%	0.9%
Tian et al., 2013	5	79	0.06	[0.02; 0.14]	0.2%	0.9%
Tigist et al., 2015	57	209	0.27	[0.21; 0.34]	0.2%	0.9%
Vouking et al., 2014	46	207	0.22	[0.17; 0.29]	0.2%	0.9%
Wanyiri et al., 2013	42	164	0.26	[0.19; 0.33]	0.1%	0.9%
Gebrecherkos et al., 2019	26	150	0.17	[0.12; 0.24]	0.2%	0.9%
Alemayehu etal., 2020	74	383	0.19	[0.15; 0.24]	0.4%	0.9%
Lengongo et al., 2020	9	115	0.08	[0.04; 0.14]	0.2%	0.9%
Rabiu et al., 2020	6	197	0.03	[0.01; 0.07]	1.0%	0.9%
Betancourth et al., 2021	3	102	0.03	[0.01; 0.08]	0.6%	0.9%
Ajayi et al., 2021	10	375	0.03	[0.01; 0.05]	2.2%	0.9%
Uzairue et al., 2021	51	170	0.30	[0.23; 0.37]	0.1%	0.9%
PrayGod et al., 2022	176	1149	0.15	[0.13; 0.18]	1.4%	0.9%
**Common effect model**		12954	0.18	[0.18; 0.19]	22.7%	--
**Random effects model**			0.26	[0.19; 0.33]	--	40.4%
Heterogeneity: $I^2 = 99\%$, $\tau^2 = 0.0559$, $p = 0$						
**Gender_at_Risk = Male**						
Adamu and Petros, 2009	9	200	0.04	[0.02; 0.08]	0.7%	0.9%
Adesij et al. 2007	12	100	0.12	[0.06; 0.20]	0.1%	0.9%
Asma et al., 2011	66	346	0.19	[0.15; 0.24]	0.3%	0.9%
Costiniuk et al., 2012	21	97	0.22	[0.14; 0.31]	0.1%	0.8%
Da Silva et al., 2005	19	100	0.19	[0.12; 0.28]	0.1%	0.8%
Dash et al., 2013	6	115	0.05	[0.02; 0.11]	0.4%	0.9%
Gaurav and Atul, 2015	6	40	0.15	[0.06; 0.30]	0.0%	0.8%
Hochberg et al., 2011	48	128	0.38	[0.29; 0.46]	0.1%	0.8%
Kroidl et al., 2016	69	187	0.37	[0.30; 0.44]	0.1%	0.9%
Mulu et al., 2013	87	220	0.40	[0.33; 0.46]	0.1%	0.9%
Nsagha et al., 2016	34	300	0.11	[0.08; 0.15]	0.5%	0.9%
Nuhu et al., 2014	8	205	0.04	[0.02; 0.08]	0.8%	0.9%
Orji et al., 2017	24	84	0.29	[0.19; 0.39]	0.1%	0.8%
Oyedeji et al., 2015	1	52	0.02	[0.00; 0.10]	0.4%	0.9%
Pavie et al., 2012	4	143	0.03	[0.01; 0.07]	0.8%	0.9%
Pennap et al., 2015	103	200	0.52	[0.44; 0.59]	0.1%	0.9%
Rodrigues et al., 2008	366	582	0.63	[0.59; 0.67]	0.4%	0.9%
Saksirisampant et al., 2009	2	90	0.02	[0.00; 0.08]	0.6%	0.9%
Samie et al., 2014	62	151	0.41	[0.33; 0.49]	0.1%	0.8%
Sanyaolu et al., 2011	12	65	0.18	[0.10; 0.30]	0.1%	0.8%
Talaat et al., 2015	28	80	0.35	[0.25; 0.46]	0.1%	0.8%
Tiwari et al., 2013	83	745	0.11	[0.09; 0.14]	1.2%	0.9%
Zeynudin et al., 2013	29	91	0.32	[0.22; 0.42]	0.1%	0.8%
Kaneshiro et al., 2019	87	252	0.35	[0.29; 0.41]	0.2%	0.9%
Chhangte et al., 2020	8	250	0.03	[0.01; 0.06]	1.3%	0.9%
McLellan et al., 2020	58	253	0.23	[0.18; 0.29]	0.2%	0.9%
Botero et al., 2021	2	192	0.01	[0.00; 0.04]	2.9%	0.9%
Chandi et al., 2021	6	80	0.07	[0.03; 0.16]	0.2%	0.9%
**Common effect model**		5348	0.10	[0.09; 0.11]	12.1%	--
**Random effects model**			0.21	[0.14; 0.27]	--	24.0%
Heterogeneity: $I^2 = 98\%$, $\tau^2 = 0.0277$, $p < 0.01$						
**Gender_at_Risk = Both**						
Assefa et al., 2013	11	272	0.04	[0.02; 0.07]	1.1%	0.9%
Barcelos et al., 2018	1	90	0.01	[0.00; 0.06]	1.3%	0.9%
**Common effect model**		362	0.02	[0.01; 0.04]	2.4%	--
**Random effects model**			0.03	[0.00; 0.05]	--	1.8%
Heterogeneity: $I^2 = 69\%$, $\tau^2 = 0.0003$, $p = 0.07$						
**Common effect model**		**27808**	**0.07**	**[0.07; 0.07]**	**100.0%**	**--**
**Random effects model**			**0.20**	**[0.16; 0.24]**	**--**	**100.0%**

Heterogeneity: $I^2 = 99\%$, $\tau^2 = 0.0423$, $p = 0$

Test for subgroup differences (fixed effect): $\chi^2_3 = 2711.$[illegible], df = [illegible] ($p =$ [illegible])

Test for subgroup differences (random effects): $\chi^2_3 = 58.02$, df = 3 ($p < 0.01$)

Figure S10B: Subgroup analysis of the prevalence of protozoan infections in people living with HIV by gender

Study	Events	Total	Proportion	95%-CI	Weight (common)	Weight (random)
**Gender_at_risk = N/A**						
Tumwine et al., 2005	67	91	0.74	[0.63; 0.82]	0.0%	0.3%
Abange et al., 2020	24	119	0.20	[0.13; 0.29]	0.1%	0.3%
Simpore et al., 2006	59	207	0.29	[0.22; 0.35]	0.1%	0.3%
Kurniawan et al., 2013	30	36	0.83	[0.67; 0.94]	0.0%	0.3%
Kapito et al., 2011	113	1142	0.10	[0.08; 0.12]	1.0%	0.4%
Sherpa et al., 2010	15	106	0.14	[0.08; 0.22]	0.1%	0.3%
Abaver et al., 2011	12	85	0.14	[0.08; 0.23]	0.1%	0.3%
Mohan et al., 2014	14	150	0.09	[0.05; 0.15]	0.1%	0.4%
Mathur et al., 2013	238	544	0.44	[0.40; 0.48]	0.2%	0.4%
Haileeyesus et al., 2013	93	378	0.25	[0.20; 0.29]	0.2%	0.4%
Kurniawan et al., 2009	266	318	0.84	[0.79; 0.88]	0.2%	0.4%
Eshetu et al., 2017	36	223	0.16	[0.12; 0.22]	0.1%	0.4%
Masoumi et al., 2019	4	102	0.04	[0.01; 0.10]	0.2%	0.4%
Vyas et al., 2013	27	75	0.36	[0.25; 0.48]	0.0%	0.3%
Mohapatra et al., 2017	23	333	0.07	[0.04; 0.10]	0.4%	0.4%
McAtee et al., 2021	32	116	0.28	[0.20; 0.37]	0.0%	0.3%
Daryani et al., 2011	54	62	0.87	[0.76; 0.94]	0.0%	0.3%
Chalwe et al., 2009	42	55	0.76	[0.63; 0.87]	0.0%	0.3%
Gupta et al., 2013	40	100	0.40	[0.30; 0.50]	0.0%	0.3%
Kulkarni et al., 2009	37	137	0.27	[0.20; 0.35]	0.1%	0.3%
Raytekar et al., 2012	25	127	0.20	[0.13; 0.28]	0.1%	0.3%
Mitra et al., 2016	120	194	0.62	[0.55; 0.69]	0.1%	0.3%
Alavi et al., 2013	31	42	0.74	[0.58; 0.86]	0.0%	0.3%
Sannella et al., 2019	113	155	0.73	[0.65; 0.80]	0.1%	0.3%
Belay et al., 2020	22	112	0.20	[0.13; 0.28]	0.1%	0.3%
Ahmed and Abhay, 2015	43	142	0.30	[0.23; 0.39]	0.1%	0.3%
Langoni and Souza, 2007	11	105	0.10	[0.05; 0.18]	0.1%	0.3%
Hari et al., 2007	25	307	0.08	[0.05; 0.12]	0.3%	0.4%
Iroezindu et al., 2012	24	97	0.25	[0.17; 0.35]	0.0%	0.3%
Paboriboune et al., 2014	89	137	0.65	[0.56; 0.73]	0.0%	0.3%
Ringo et al., 2015	12	83	0.14	[0.08; 0.24]	0.0%	0.3%
Rodrigues et al., 2011	2	19	0.11	[0.01; 0.33]	0.0%	0.3%
Sarfati et al., 2006	17	154	0.11	[0.07; 0.17]	0.1%	0.4%
Purbey., 2021	7	75	0.09	[0.04; 0.18]	0.1%	0.3%
Agholi et al., 2013	81	356	0.23	[0.18; 0.27]	0.2%	0.4%
Lehman et al., 2013	21	201	0.10	[0.07; 0.16]	0.2%	0.4%
Osuji et al., 2012	74	134	0.55	[0.46; 0.64]	0.0%	0.3%
Guk et al., 2005	13	105	0.12	[0.07; 0.20]	0.1%	0.3%
Wumba et al., 2012	43	242	0.18	[0.13; 0.23]	0.1%	0.4%
Adu-Gyasi et al., 2013	15	933	0.02	[0.01; 0.03]	4.3%	0.4%
Echoru et al., 2015	6	111	0.05	[0.02; 0.11]	0.2%	0.4%
Morona et al., 2018	56	250	0.22	[0.17; 0.28]	0.1%	0.3%
Rezaei et al., 2018	19	251	0.08	[0.05; 0.12]	0.3%	0.4%
Garcia et al., 2006	28	92	0.30	[0.21; 0.41]	0.0%	0.3%
Cardoso et al., 2011	19	500	0.04	[0.02; 0.06]	1.0%	0.4%
Wumba et al., 2010	33	175	0.19	[0.13; 0.25]	0.1%	0.3%
Abilo and Afework, 2005	30	70	0.43	[0.31; 0.55]	0.0%	0.3%
Akinbo et al., 2011	21	463	0.05	[0.03; 0.07]	0.8%	0.4%
Amuta et al., 2012	343	1080	0.32	[0.29; 0.35]	0.4%	0.4%
Carranza et al., 2009	26	163	0.16	[0.11; 0.22]	0.1%	0.3%
Gebretsadik et al., 2018	23	223	0.10	[0.07; 0.15]	0.2%	0.4%
Ghafari et al., 2018	27	250	0.11	[0.07; 0.15]	0.2%	0.4%
Igbeneghu et al., 2012	18	38	0.47	[0.31; 0.64]	0.0%	0.3%
Jayalakshmi et al., 2008	22	89	0.25	[0.16; 0.35]	0.0%	0.3%
Kammili et al., 2008	42	175	0.24	[0.18; 0.31]	0.1%	0.3%
Kucerova et al., 2011	19	46	0.41	[0.27; 0.57]	0.0%	0.3%
Malla et al., 2005	14	100	0.14	[0.08; 0.22]	0.1%	0.3%
Meamar et al., 2007	90	781	0.12	[0.09; 0.14]	0.6%	0.4%
Mehta et al., 2013	25	100	0.25	[0.17; 0.35]	0.0%	0.3%
Moran et al., 2009	31	100	0.31	[0.22; 0.41]	0.0%	0.3%
Moran et al., 2005	115	203	0.57	[0.50; 0.64]	0.1%	0.3%
Oliveira et al., 2011	13	169	0.08	[0.04; 0.13]	0.2%	0.4%
Oshinaike et al., 2010	71	83	0.86	[0.76; 0.92]	0.0%	0.3%
Patel et al., 2011	28	100	0.28	[0.19; 0.38]	0.0%	0.3%
Pinlaor et al., 2005	13	78	0.17	[0.09; 0.27]	0.0%	0.3%
Ramakrishnan et al., 2007	57	80	0.71	[0.60; 0.81]	0.0%	0.3%
Ramana et al., 2010	364	452	0.81	[0.77; 0.84]	0.2%	0.4%
Rashmi and Ravi, 2013	15	90	0.17	[0.10; 0.26]	0.0%	0.3%
Sanyaolu et al., 2013	31	65	0.48	[0.35; 0.60]	0.0%	0.3%
Shafieenia et al., 2018	142	379	0.37	[0.33; 0.43]	0.1%	0.4%
Shimelis and Tadesse, 2014	32	250	0.13	[0.09; 0.18]	0.2%	0.4%
Sonali et al., 2014	68	250	0.27	[0.22; 0.33]	0.1%	0.3%
Tagoe and Boachie, 2012	34	220	0.15	[0.11; 0.21]	0.1%	0.4%
Tellevik et al., 2015	8	33	0.24	[0.11; 0.42]	0.0%	0.3%
Namaji et al., 2020	129	361	0.36	[0.31; 0.41]	0.1%	0.4%
Stiffler et al., 2021	44	129	0.34	[0.26; 0.43]	0.0%	0.3%
Yulfi et al., 2021	18	54	0.33	[0.21; 0.47]	0.0%	0.3%
Weinreich et al., 2021	86	967	0.09	[0.07; 0.11]	0.9%	0.4%
Bayleyegn et al., 2021	30	255	0.12	[0.08; 0.16]	0.2%	0.4%
Feleke et al., 2022	22	223	0.10	[0.06; 0.15]	0.2%	0.4%
Common effect model		17597	0.13	[0.13; 0.14]	15.3%	--
Random effects model			0.29	[0.24; 0.34]	--	27.7%
Heterogeneity: $I^2 = 99\%$, $\tau^2 = 0.0527$, $p = 0$						
**Gender_at_risk = Male**						
Okonkwo et al., 2018	61	179	0.34	[0.27; 0.42]	0.1%	0.3%
Marete et al., 2014	201	245	0.82	[0.77; 0.87]	0.1%	0.4%
Alberto et al., 2011	38	131	0.29	[0.21; 0.38]	0.0%	0.3%
Huibers et al., 2018	19	35	0.54	[0.37; 0.71]	0.0%	0.3%
Gholami et al., 2016	4	53	0.08	[0.02; 0.18]	0.1%	0.3%
Pennap et al., 2015	74	200	0.37	[0.30; 0.44]	0.1%	0.3%
Rezanezhad et al., 2017	19	90	0.21	[0.13; 0.31]	0.0%	0.3%
Iqbal et al., 2015	43	346	0.12	[0.09; 0.16]	0.2%	0.4%
Adesij et al. 2007	85	100	0.85	[0.76; 0.91]	0.1%	0.3%
Masarat et al., 2012	45	45	1.00	[0.92; 1.00]	0.3%	0.4%
Cohen et al., 2005	18	110	0.16	[0.10; 0.25]	0.1%	0.3%
Rahimi et al., 2015	79	82	0.96	[0.90; 0.99]	0.2%	0.4%
Uneke et al., 2005	103	490	0.21	[0.17; 0.25]	0.2%	0.4%
Li et al., 2010	58	183	0.32	[0.25; 0.39]	0.1%	0.3%
Beena et al., 2015	141	661	0.21	[0.18; 0.25]	0.3%	0.4%
Ganiem et al., 2013	21	64	0.33	[0.22; 0.46]	0.0%	0.3%
Reema and Dharmendra, 2017	52	100	0.52	[0.42; 0.62]	0.0%	0.3%
Machala et al., 2009	268	626	0.43	[0.39; 0.47]	0.2%	0.4%
Tiwari et al. 2013	84	745	0.11	[0.09; 0.14]	0.6%	0.4%
Sherchan et al., 2012	44	146	0.30	[0.23; 0.38]	0.1%	0.3%
Zeynudin et al., 2013	31	91	0.34	[0.24; 0.45]	0.0%	0.3%
Nissapatorn et al., 2007	329	693	0.47	[0.44; 0.51]	0.2%	0.4%
Hung et al., 2008	66	991	0.07	[0.05; 0.08]	1.2%	0.4%
Hung et al., 2007	5	1376	0.00	[0.00; 0.01]	25.8%	0.4%
Yosefi et al., 2012	27	60	0.45	[0.32; 0.58]	0.0%	0.3%
Maikai et al., 2012	1	61	0.02	[0.00; 0.09]	0.2%	0.4%
Ngum et al., 2015	138	320	0.43	[0.38; 0.49]	0.1%	0.3%
Onyenekwe et al., 2007	28	149	0.19	[0.13; 0.26]	0.1%	0.3%
Quigley et al., 2005	154	222	0.69	[0.63; 0.75]	0.1%	0.3%
Ajjampur et al., 2007	48	534	0.09	[0.07; 0.12]	0.5%	0.4%
Sanyaolu et al., 2011	26	65	0.40	[0.28; 0.53]	0.0%	0.3%
Rodrigues et al., 2008	287	582	0.49	[0.45; 0.53]	0.2%	0.4%
Sucilathangam et al., 2012	52	103	0.50	[0.40; 0.60]	0.0%	0.3%
Shafiei et al., 2011	49	121	0.40	[0.32; 0.50]	0.0%	0.3%
Nazari et al., 2018	167	385	0.43	[0.38; 0.48]	0.1%	0.4%
Aghaee et al., 2017	11	49	0.22	[0.12; 0.37]	0.0%	0.3%
Mohraz et al., 2011	100	201	0.50	[0.43; 0.57]	0.1%	0.3%
Barbosa et al., 2020	65	113	0.58	[0.48; 0.67]	0.0%	0.3%
Barbosa et al., 2020	110	309	0.36	[0.30; 0.41]	0.1%	0.3%
Steinberg et al., 2021	173	215	0.80	[0.75; 0.86]	0.1%	0.3%
Abdollahi et al., 2013	65	100	0.65	[0.55; 0.74]	0.0%	0.3%
Cavalcanti et al., 2012	5	10	0.50	[0.19; 0.81]	0.0%	0.3%
Desta et al., 2005	8	49	0.16	[0.07; 0.30]	0.0%	0.3%
Hung et al., 2005	56	550	0.10	[0.08; 0.13]	0.4%	0.4%
Júnior et al., 2015	7	15	0.47	[0.21; 0.73]	0.0%	0.3%
Llenas et al., 2012	4	155	0.03	[0.01; 0.06]	0.4%	0.4%
Almeida et al., 2012	5	10	0.50	[0.19; 0.81]	0.0%	0.3%
Abdollahi et al., 2015	28	91	0.31	[0.22; 0.41]	0.0%	0.3%
Asma et al., 2011	105	346	0.30	[0.26; 0.35]	0.1%	0.4%
Anyanwu et al., 2020	142	420	0.34	[0.29; 0.39]	0.1%	0.4%
Dash et al., 2013	44	115	0.38	[0.29; 0.48]	0.0%	0.3%
Gabriela et al., 2005	59	397	0.15	[0.12; 0.19]	0.2%	0.4%
Addebbous et al., 2012	59	95	0.62	[0.52; 0.72]	0.0%	0.3%
Rostami et al., 2014	19	94	0.20	[0.13; 0.30]	0.0%	0.3%
Ebrahim et al., 2018	51	246	0.21	[0.16; 0.26]	0.1%	0.4%
da Silva et al., 2005	12	100	0.12	[0.06; 0.20]	0.1%	0.3%
Berenji et al., 2010	21	31	0.68	[0.49; 0.83]	0.0%	0.3%
Xavier et al., 2013	200	250	0.80	[0.74; 0.85]	0.1%	0.4%
Osunkalu et al., 2011	206	380	0.54	[0.49; 0.59]	0.1%	0.4%
Davarpanah et al., 2007	38	208	0.18	[0.13; 0.24]	0.1%	0.3%
Chandi et al., 2021	10	80	0.12	[0.06; 0.22]	0.1%	0.3%
Saksirisampant et al., 2009	35	90	0.39	[0.29; 0.50]	0.0%	0.3%
Bavand et al., 2019	66	149	0.44	[0.36; 0.53]	0.0%	0.3%
Bharti et al., 2012	45	460	0.10	[0.07; 0.13]	0.4%	0.4%
Nsagha et al., 2016	223	300	0.74	[0.69; 0.79]	0.1%	0.4%
Chemoh et al., 2015	109	300	0.36	[0.31; 0.42]	0.1%	0.3%
Sajedi et al., 2021	73	121	0.60	[0.51; 0.69]	0.0%	0.3%
Arefkhah et al., 2018	42	251	0.17	[0.12; 0.22]	0.1%	0.4%
Adamu et al., 2014	139	520	0.27	[0.23; 0.31]	0.2%	0.4%
Pavie et al., 2012	21	143	0.15	[0.09; 0.22]	0.1%	0.3%
Adamu and Petros, 2009	87	200	0.44	[0.37; 0.51]	0.1%	0.3%
Colomba et al., 2009	24	145	0.17	[0.11; 0.24]	0.1%	0.3%
Ayi et al., 2016	72	125	0.58	[0.48; 0.66]	0.0%	0.3%
Shafiei et al., 2014	9	49	0.18	[0.09; 0.32]	0.0%	0.3%
Hoshina et al., 2020	33	399	0.08	[0.06; 0.11]	0.4%	0.4%
Botero et al., 2021	50	192	0.26	[0.20; 0.33]	0.1%	0.3%
Bokharaei et al., 2020	95	108	0.88	[0.80; 0.93]	0.1%	0.3%
Cunha et al., 2020	36	240	0.15	[0.11; 0.20]	0.1%	0.4%
Archana et al., 2017	17	104	0.16	[0.10; 0.25]	0.1%	0.3%
Gaurav and Atul, 2015	14	40	0.35	[0.21; 0.52]	0.0%	0.3%
Hussain et al., 2016	25	127	0.20	[0.13; 0.28]	0.1%	0.3%
Kodym et al., 2015	502	1235	0.41	[0.38; 0.43]	0.4%	0.4%
Nuhu et al., 2014	34	205	0.17	[0.12; 0.22]	0.1%	0.4%
Rodrigues et al., 2005	18	28	0.64	[0.44; 0.81]	0.0%	0.3%
Shen et al., 2016	25	259	0.10	[0.06; 0.14]	0.2%	0.4%
Stark et al., 2007	288	628	0.46	[0.42; 0.50]	0.2%	0.4%
Stensvold et al., 2011	32	96	0.33	[0.24; 0.44]	0.0%	0.3%
Taherkhani et al., 2007	20	75	0.27	[0.17; 0.38]	0.0%	0.3%
Uysal et al., 2017	6	115	0.05	[0.02; 0.11]	0.2%	0.4%
Chhangte et al., 2020	61	250	0.24	[0.19; 0.30]	0.1%	0.3%
Dutta et al., 2020	292	400	0.73	[0.68; 0.77]	0.2%	0.4%
Sepahvand et al., 2022	7	26	0.27	[0.12; 0.48]	0.0%	0.3%
Common effect model		22393	0.09	[0.09; 0.09]	37.2%	--
Random effects model			0.36	[0.31; 0.41]	--	31.5%
Heterogeneity: $I^2 = 99\%$, $\tau^2 = 0.0536$, $p = 0$						
**Gender_at_risk = Female**						
Amodu et al., 2020	100	140	0.71	[0.63; 0.79]	0.1%	0.3%
Kiying et al., 2010	120	160	0.75	[0.68; 0.81]	0.1%	0.3%
Oyedeji et al., 2015	10	52	0.19	[0.10; 0.33]	0.0%	0.3%
Bate et al., 2016	58	234	0.25	[0.19; 0.31]	0.1%	0.3%
Ako-Nai et al., 2013	34	44	0.77	[0.62; 0.89]	0.0%	0.3%
Anteneh et al., 2014	178	190	0.94	[0.89; 0.97]	0.2%	0.4%
Nkhoma et al., 2012	70	185	0.38	[0.31; 0.45]	0.1%	0.3%
Franke et al., 2010	199	1066	0.19	[0.16; 0.21]	0.5%	0.4%
Kawai et al., 2009	177	971	0.18	[0.16; 0.21]	0.5%	0.4%
Ojurongbe et al., 2011	56	96	0.58	[0.48; 0.68]	0.0%	0.3%
Akinbo et al., 2010	36	400	0.09	[0.06; 0.12]	0.4%	0.4%
Goselle et al., 2007	64	200	0.32	[0.26; 0.39]	0.1%	0.3%
Tigist et al., 2015	38	209	0.18	[0.13; 0.24]	0.1%	0.3%
Djieyep et al., 2014	222	305	0.73	[0.67; 0.78]	0.1%	0.4%
Nwadioha et al., 2012	84	350	0.24	[0.20; 0.29]	0.1%	0.4%
Izuka et al., 2017	70	102	0.69	[0.59; 0.77]	0.0%	0.3%
Kfutwah et al., 2009	14	80	0.17	[0.10; 0.28]	0.0%	0.3%
Abdullahi et al., 2020	28	58	0.48	[0.35; 0.62]	0.0%	0.3%
Alonso et al., 2021	26	144	0.18	[0.12; 0.25]	0.1%	0.3%
Fisseha et al., 2013	90	103	0.87	[0.79; 0.93]	0.1%	0.3%
Manyanga et al., 2014	19	420	0.05	[0.03; 0.07]	0.7%	0.4%
Wumba et al., 2015	19	25	0.76	[0.55; 0.91]	0.0%	0.3%
Price et al., 2018	76	359	0.21	[0.17; 0.26]	0.2%	0.4%
Frosch et al., 2017	11	138	0.08	[0.04; 0.14]	0.1%	0.4%
Zheng et al., 2017	14	101	0.14	[0.08; 0.22]	0.1%	0.3%
Olaniran et al., 2012	83	93	0.89	[0.81; 0.95]	0.1%	0.3%
Domingos et al., 2013	92	200	0.46	[0.39; 0.53]	0.1%	0.3%
Kipyegen et al., 2012	122	285	0.43	[0.37; 0.49]	0.1%	0.3%
Mengistu et al., 2014	38	43	0.88	[0.75; 0.96]	0.0%	0.3%
Ngobeni and Samie, 2017	38	100	0.38	[0.28; 0.48]	0.0%	0.3%
Nkenfou et al., 2013	25	42	0.60	[0.43; 0.74]	0.0%	0.3%
Srisuphanunt et al., 2008	23	143	0.16	[0.10; 0.23]	0.1%	0.3%
Johnbull et al., 2014	149	301	0.50	[0.44; 0.55]	0.1%	0.3%
Assefa et al., 2009	152	214	0.71	[0.64; 0.77]	0.1%	0.3%
Rabiu et al., 2020	49	197	0.25	[0.19; 0.32]	0.1%	0.3%
Tegegne et al., 2017	36	223	0.16	[0.12; 0.22]	0.1%	0.4%
Zeleke and Melsew, 2017	255	270	0.94	[0.91; 0.97]	0.4%	0.4%
Amoo et al., 2018	52	231	0.23	[0.17; 0.28]	0.1%	0.3%
Nkenfou et al., 2017	62	68	0.91	[0.82; 0.97]	0.1%	0.3%
Sitoe et al., 2010	18	58	0.31	[0.20; 0.45]	0.0%	0.3%
Tay et al., 2015	47	400	0.12	[0.09; 0.15]	0.3%	0.4%
Hasang et al., 2014	100	216	0.46	[0.40; 0.53]	0.1%	0.3%
Shimelis et al., 2016	138	491	0.28	[0.24; 0.32]	0.2%	0.4%
Daryani et al., 2009	11	64	0.17	[0.09; 0.29]	0.0%	0.3%
Missaye et al., 2013	66	272	0.24	[0.19; 0.30]	0.1%	0.4%
Dechassa et al., 2016	109	135	0.81	[0.73; 0.87]	0.1%	0.3%
Girma et al., 2014	94	268	0.35	[0.29; 0.41]	0.1%	0.3%
Mekonnen et al., 2014	96	268	0.36	[0.30; 0.42]	0.1%	0.3%
Suryawanshi et al., 2012	43	100	0.43	[0.33; 0.53]	0.0%	0.3%
Feyisayo et al., 2014	8	105	0.08	[0.03; 0.14]	0.1%	0.3%
Jegede et al., 2014	8	105	0.08	[0.03; 0.14]	0.1%	0.3%
Lar et al., 2016	50	205	0.24	[0.19; 0.31]	0.1%	0.3%
Gebrecherkos et al., 2019	59	150	0.39	[0.31; 0.48]	0.0%	0.3%
Kiros et al., 2015	122	399	0.31	[0.26; 0.35]	0.1%	0.4%
Morawski et al., 2017	16	202	0.08	[0.05; 0.13]	0.2%	0.4%
Nweze and Mouneke, 2011	355	970	0.37	[0.34; 0.40]	0.3%	0.4%
Ogoina et al., 2013	43	111	0.39	[0.30; 0.48]	0.0%	0.3%
Ouermi et al., 2009	44	138	0.32	[0.24; 0.40]	0.0%	0.3%
Njunda et al., 2016	26	355	0.07	[0.05; 0.11]	0.4%	0.4%
Idindili et al., 2011	105	421	0.25	[0.21; 0.29]	0.2%	0.4%
Minti et al., 2017	1	27	0.04	[0.00; 0.19]	0.0%	0.3%
Saracino et al., 2012	29	220	0.13	[0.09; 0.18]	0.1%	0.4%
Sandie et al., 2021	31	190	0.16	[0.11; 0.22]	0.1%	0.3%
Tsegaye et al., 2014	150	170	0.88	[0.82; 0.93]	0.1%	0.4%
Roka et al., 2013	257	273	0.94	[0.91; 0.97]	0.4%	0.4%
Iroezindu et al., 2012	21	63	0.33	[0.22; 0.46]	0.0%	0.3%
Masese et al., 2011	31	147	0.21	[0.15; 0.29]	0.1%	0.3%
Wanyiri et al., 2013	68	164	0.41	[0.34; 0.49]	0.1%	0.3%
Vouking et al., 2014	73	207	0.35	[0.29; 0.42]	0.1%	0.3%
Erhabor et al., 2011	3	105	0.03	[0.01; 0.08]	0.2%	0.4%
Wanyiri et al., 2014	56	164	0.34	[0.27; 0.42]	0.1%	0.3%
Olopade et al., 2017	10	226	0.04	[0.02; 0.08]	0.4%	0.4%
Akinbo et al., 2012	190	2000	0.10	[0.08; 0.11]	1.7%	0.4%
Alemayehu et al., 2020	15	383	0.04	[0.02; 0.06]	0.7%	0.4%
Jegede et al., 2020	386	1521	0.25	[0.23; 0.28]	0.6%	0.4%
Sintayehu et al., 2013	78	343	0.23	[0.18; 0.28]	0.1%	0.4%
Kissinger et al., 2008	11	60	0.18	[0.10; 0.30]	0.0%	0.3%
Omoti et al., 2013	254	342	0.74	[0.69; 0.79]	0.1%	0.4%
Cota et al., 2013	41	113	0.36	[0.27; 0.46]	0.0%	0.3%
Davis et al., 2016	16	44	0.36	[0.22; 0.52]	0.0%	0.3%
Pappoe et al., 2017	293	394	0.74	[0.70; 0.79]	0.2%	0.4%
Ajayi et al., 2021	94	375	0.25	[0.21; 0.30]	0.1%	0.4%
Fang et al., 2021	164	325	0.50	[0.45; 0.56]	0.1%	0.3%
Almeida et al., 2010	9	716	0.01	[0.01; 0.02]	4.1%	0.4%
Chukwuanukwu et al., 2016	110	239	0.46	[0.40; 0.53]	0.1%	0.3%
Ojuromi et al., 2016	4	90	0.04	[0.01; 0.11]	0.1%	0.4%
Tian et al., 2012	70	302	0.23	[0.19; 0.28]	0.1%	0.4%
Tian et al., 2013	18	79	0.23	[0.14; 0.34]	0.0%	0.3%
Pandey et al., 2018	176	305	0.58	[0.52; 0.63]	0.1%	0.3%
Betancourth et al., 2021	31	102	0.30	[0.22; 0.40]	0.0%	0.3%
Wang et al., 2013	11	683	0.02	[0.01; 0.03]	3.1%	0.4%
Lengongo et al., 2020	54	115	0.47	[0.38; 0.56]	0.0%	0.3%
Dankwa et al., 2021	26	418	0.06	[0.04; 0.09]	0.5%	0.4%
Akinbo et al., 2016	32	411	0.08	[0.05; 0.11]	0.4%	0.4%
Amuta et al., 2012	39	360	0.11	[0.08; 0.15]	0.3%	0.4%
Angal et al., 2016	84	133	0.63	[0.54; 0.71]	0.0%	0.3%
Assob et al., 2011	93	133	0.70	[0.61; 0.78]	0.0%	0.3%
Bamba et al., 2014	11	16	0.69	[0.41; 0.89]	0.0%	0.3%
Bessong and Mathomu 2010	29	160	0.18	[0.12; 0.25]	0.1%	0.3%
De Lemos and Garcia-Zapata, 2010	23	125	0.18	[0.12; 0.26]	0.1%	0.3%
Gallagher et al., 2005	34	83	0.41	[0.30; 0.52]	0.0%	0.3%
Getaneh et al., 2018	47	220	0.21	[0.16; 0.27]	0.1%	0.3%
Lago et al., 2009	121	168	0.72	[0.65; 0.79]	0.1%	0.3%
Lemos et al., 2009	23	125	0.18	[0.12; 0.26]	0.1%	0.3%
Mtapuri et al., 2014	21	29	0.72	[0.53; 0.87]	0.0%	0.3%
Nangammbi et al., 2013	32	155	0.21	[0.15; 0.28]	0.1%	0.3%
Okogun et al., 2010	5	16	0.31	[0.11; 0.59]	0.0%	0.3%
Tegenaw et al., 2013	2	23	0.09	[0.01; 0.28]	0.0%	0.3%
Chukwuocha et al., 2019	398	398	1.00	[0.99; 1.00]	23.8%	0.4%
Ayele et al., 2020	31	180	0.17	[0.12; 0.24]	0.1%	0.3%
Medina et al., 2020	86	427	0.20	[0.16; 0.24]	0.2%	0.4%
Zakari et al., 2020	54	160	0.34	[0.26; 0.42]	0.1%	0.3%
Uzairue et al., 2021	25	170	0.15	[0.10; 0.21]	0.1%	0.3%
Jemikalajah et al., 2021	228	300	0.76	[0.71; 0.81]	0.1%	0.4%
Common effect model		28672	0.60	[0.60; 0.60]	46.9%	--
Random effects model			0.37	[0.32; 0.41]	--	39.4%
Heterogeneity: $I^2 = 100\%$, $\tau^2 = 0.0697$, $p = 0$						
**Gender_at_risk = Both**						
Assefa et al., 2013	43	272	0.16	[0.12; 0.21]	0.2%	0.4%
Barcelos et al., 2018	25	90	0.28	[0.19; 0.38]	0.0%	0.3%
Charoensakulchai et al., 2020	110	526	0.21	[0.18; 0.25]	0.2%	0.4%
Moro et al., 2020	81	332	0.24	[0.20; 0.29]	0.1%	0.4%
Common effect model		1220	0.21	[0.19; 0.23]	0.6%	--
Random effects model			0.21	[0.17; 0.26]	--	1.4%
Heterogeneity: $I^2 = 69\%$, $\tau^2 = 0.0015$, $p = 0.02$						
**Common effect model**		**69882**	**0.34**	**[0.33; 0.34]**	**100.0%**	**--**
**Random effects model**			**0.34**	**[0.31; 0.37]**	**--**	**100.0%**

Heterogeneity: $I^2 = 100\%$, $\tau^2 = 0.0597$, $p = 0$

Test for subgroup differences (fixed effect): $\chi^2_3 = 82854.69$, df = 3 ($p = 0$)

Test for subgroup differences (random effects): $\chi^2_3 = 26.23$, df = 3 ($p < 0.01$)

0.2 0.4 0.6 0.8 1

Figure S11A: Subgroup analysis of the prevalence of helminth infections in people living with HIV by sample size

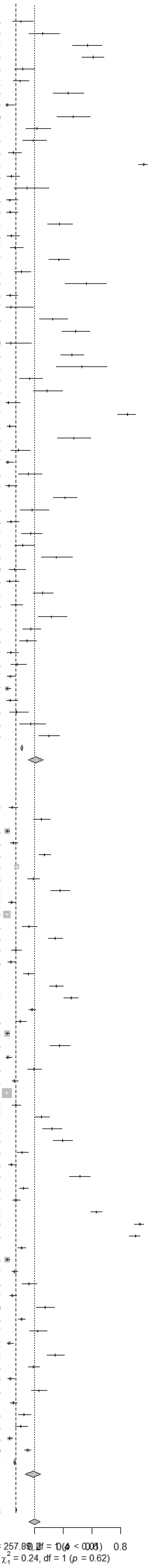

Study	Events	Total	Proportion	95%-CI	Weight (common)	Weight (random)
**Sample_Size = <200**						
Abaver et al., 2011	9	85	0.11	[0.05; 0.19]	0.1%	0.9%
Abilo and Afework, 2005	18	70	0.26	[0.16; 0.38]	0.1%	0.8%
Abossie and Petros, 2015	55	97	0.57	[0.46; 0.67]	0.1%	0.8%
Adams et al., 2006	103	170	0.61	[0.53; 0.68]	0.1%	0.9%
Adesij et al. 2007	12	100	0.12	[0.06; 0.20]	0.1%	0.9%
Ahmed and Abhay, 2015	14	142	0.10	[0.05; 0.16]	0.2%	0.9%
Babatunde et al., 2010	39	90	0.43	[0.33; 0.54]	0.1%	0.8%
Barcelos et al., 2018	1	90	0.01	[0.00; 0.06]	1.3%	0.9%
Colombe et al., 2018	37	79	0.47	[0.36; 0.58]	0.0%	0.8%
Costiniuk et al., 2012	21	97	0.22	[0.14; 0.31]	0.1%	0.8%
Da Silva et al., 2005	19	100	0.19	[0.12; 0.28]	0.1%	0.8%
Dash et al., 2013	6	115	0.05	[0.02; 0.11]	0.4%	0.9%
Erikstrup et al., 2008	190	198	0.96	[0.92; 0.98]	0.8%	0.9%
Feyisayo et al., 2014	4	105	0.04	[0.01; 0.09]	0.4%	0.9%
Gaurav and Atul, 2015	6	40	0.15	[0.06; 0.30]	0.0%	0.8%
Guk et al., 2005	3	105	0.03	[0.01; 0.08]	0.6%	0.9%
Gupta et al., 2013	3	100	0.03	[0.01; 0.09]	0.5%	0.9%
Hochberg et al., 2011	48	128	0.38	[0.29; 0.46]	0.1%	0.8%
Jegede et al., 2014	4	105	0.04	[0.01; 0.09]	0.4%	0.9%
Kulkarni et al., 2009	9	137	0.07	[0.03; 0.12]	0.3%	0.9%
Kroidl et al., 2016	69	187	0.37	[0.30; 0.44]	0.1%	0.9%
Mascarello et al., 2011	15	138	0.11	[0.06; 0.17]	0.2%	0.9%
Mazigo et al., 2016	28	50	0.56	[0.41; 0.70]	0.0%	0.8%
Mehta et al., 2013	3	100	0.03	[0.01; 0.09]	0.5%	0.9%
Minti et al., 2017	1	27	0.04	[0.00; 0.19]	0.1%	0.9%
Mkhize et al., 2017	31	95	0.33	[0.23; 0.43]	0.1%	0.8%
Modjarrad et al., 2005	54	111	0.49	[0.39; 0.58]	0.1%	0.8%
Mtapuri et al., 2014	1	29	0.03	[0.00; 0.18]	0.1%	0.9%
Ndhlovu et al., 2007	72	156	0.46	[0.38; 0.54]	0.1%	0.8%
Nielsen et al., 2007	18	34	0.53	[0.35; 0.70]	0.0%	0.7%
Ojurongbe et al., 2011	16	96	0.17	[0.10; 0.26]	0.1%	0.9%
Orji et al., 2017	24	84	0.29	[0.19; 0.39]	0.1%	0.8%
Oyedeji et al., 2015	1	52	0.02	[0.00; 0.10]	0.4%	0.9%
Paboriboune et al., 2014	116	137	0.85	[0.78; 0.90]	0.2%	0.9%
Pavie et al., 2012	4	143	0.03	[0.01; 0.07]	0.8%	0.9%
Pinlaor et al., 2005	37	78	0.47	[0.36; 0.59]	0.0%	0.8%
Ramakrishnan et al., 2007	7	80	0.09	[0.04; 0.17]	0.2%	0.9%
Raytekar et al., 2012	2	127	0.02	[0.00; 0.06]	1.3%	0.9%
Ringo et al., 2015	13	83	0.16	[0.09; 0.25]	0.1%	0.8%
Saksirisampant et al., 2009	2	90	0.02	[0.00; 0.08]	0.6%	0.9%
Samie et al., 2014	62	151	0.41	[0.33; 0.49]	0.1%	0.8%
Sanyaolu et al., 2011	12	65	0.18	[0.10; 0.30]	0.1%	0.8%
Sherpa et al., 2010	4	106	0.04	[0.01; 0.09]	0.5%	0.9%
Smith et al., 2008	21	122	0.17	[0.11; 0.25]	0.1%	0.9%
Suryawanshi et al., 2012	12	100	0.12	[0.06; 0.20]	0.1%	0.9%
Talaat et al., 2015	28	80	0.35	[0.25; 0.46]	0.1%	0.8%
Tian et al., 2013	5	79	0.06	[0.02; 0.14]	0.2%	0.9%
Vyas et al., 2013	2	75	0.03	[0.00; 0.09]	0.4%	0.9%
Wanyiri et al., 2013	42	164	0.26	[0.19; 0.33]	0.1%	0.9%
Wumba et al., 2010	12	175	0.07	[0.04; 0.12]	0.4%	0.9%
Zeynudin et al., 2013	29	91	0.32	[0.22; 0.42]	0.1%	0.8%
Gebrecherkos et al., 2019	26	150	0.17	[0.12; 0.24]	0.2%	0.9%
Sannella et al., 2019	23	155	0.15	[0.10; 0.21]	0.2%	0.9%
Belay et al., 2020	4	112	0.04	[0.01; 0.09]	0.5%	0.9%
Lengongo et al., 2020	9	115	0.08	[0.04; 0.14]	0.2%	0.9%
Rabiu et al., 2020	6	197	0.03	[0.01; 0.07]	1.0%	0.9%
Botero et al., 2021	2	192	0.01	[0.00; 0.04]	2.9%	0.9%
Betancourth et al., 2021	3	102	0.03	[0.01; 0.08]	0.6%	0.9%
Chandi et al., 2021	6	80	0.07	[0.03; 0.16]	0.2%	0.9%
Purbey., 2021	13	75	0.17	[0.10; 0.28]	0.1%	0.8%
Uzairue et al., 2021	51	170	0.30	[0.23; 0.37]	0.1%	0.9%
**Common effect model**		**6606**	**0.11**	**[0.11; 0.12]**	**19.4%**	**--**
**Random effects model**			**0.21**	**[0.15; 0.26]**	**--**	**52.1%**
Heterogeneity: $I^2 = 99\%$, $\tau^2 = 0.0447$, $p = 0$						
**Sample_Size = ≥200**						
Adamu and Petros, 2009	9	200	0.04	[0.02; 0.08]	0.7%	0.9%
Adeleke et al., 2015	57	231	0.25	[0.19; 0.31]	0.2%	0.9%
Agholi et al., 2013	4	356	0.01	[0.00; 0.03]	5.0%	0.9%
Akinbo and Omoregie, 2011	15	285	0.05	[0.03; 0.09]	0.9%	0.9%
Akinbo et al., 2011	125	463	0.27	[0.23; 0.31]	0.4%	0.9%
Akinbo et al., 2012	156	2000	0.08	[0.07; 0.09]	4.3%	0.9%
Asma et al., 2011	66	346	0.19	[0.15; 0.24]	0.3%	0.9%
Assefa et al., 2009	81	214	0.38	[0.31; 0.45]	0.1%	0.9%
Assefa et al., 2013	11	272	0.04	[0.02; 0.07]	1.1%	0.9%
Cardoso et al., 2011	4	500	0.01	[0.00; 0.02]	9.8%	0.9%
Eshetu et al., 2017	36	223	0.16	[0.12; 0.22]	0.3%	0.9%
Fekadu et al., 2013	118	343	0.34	[0.29; 0.40]	0.2%	0.9%
Getaneh et al., 2018	15	220	0.07	[0.04; 0.11]	0.5%	0.9%
Gebretsadik et al., 2018	8	223	0.04	[0.02; 0.07]	1.0%	0.9%
Haileeyesus et al., 2013	59	378	0.16	[0.12; 0.20]	0.4%	0.9%
Idindili et al., 2011	148	421	0.35	[0.31; 0.40]	0.3%	0.9%
Kallestrup et al., 2005	185	407	0.45	[0.41; 0.50]	0.3%	0.9%
Kawai et al., 2009	178	971	0.18	[0.16; 0.21]	1.0%	0.9%
Kipyegen et al., 2012	29	285	0.10	[0.07; 0.14]	0.5%	0.9%
Kurniawan et al., 2009	3	318	0.01	[0.00; 0.03]	5.3%	0.9%
Lar et al., 2016	77	205	0.38	[0.31; 0.45]	0.1%	0.9%
Lehman et al., 2013	3	201	0.01	[0.00; 0.04]	2.1%	0.9%
Marti et al., 2017	60	305	0.20	[0.15; 0.25]	0.3%	0.9%
Mathur et al., 2013	34	544	0.06	[0.04; 0.09]	1.4%	0.9%
Meamar et al., 2007	5	781	0.01	[0.00; 0.01]	19.0%	0.9%
Missaye et al., 2013	19	272	0.07	[0.04; 0.11]	0.6%	0.9%
Modjarrad et al., 2005	74	297	0.25	[0.20; 0.30]	0.2%	0.9%
Morawski et al., 2017	65	202	0.32	[0.26; 0.39]	0.1%	0.9%
Mulu et al., 2013	87	220	0.40	[0.33; 0.46]	0.1%	0.9%
Nsagha et al., 2016	34	300	0.11	[0.08; 0.15]	0.5%	0.9%
Nuhu et al., 2014	8	205	0.04	[0.02; 0.08]	0.8%	0.9%
Pennap et al., 2015	103	200	0.52	[0.44; 0.59]	0.1%	0.9%
Plumelle et al., 2015	55	445	0.12	[0.09; 0.16]	0.6%	0.9%
Ramana et al., 2010	32	452	0.07	[0.05; 0.10]	1.1%	0.9%
Rodrigues et al., 2008	366	582	0.63	[0.59; 0.67]	0.4%	0.9%
Roka et al., 2012	242	260	0.93	[0.89; 0.96]	0.6%	0.9%
Roka et al., 2013	246	273	0.90	[0.86; 0.93]	0.5%	0.9%
Shimelis et al., 2016	54	491	0.11	[0.08; 0.14]	0.8%	0.9%
Sonali et al., 2014	2	250	0.01	[0.00; 0.03]	4.9%	0.9%
Talaat et al., 2008	40	632	0.06	[0.05; 0.09]	1.7%	0.9%
Tegegne et al., 2017	36	223	0.16	[0.12; 0.22]	0.3%	0.9%
Tian et al., 2012	14	302	0.05	[0.03; 0.08]	1.1%	0.9%
Tigist et al., 2015	57	209	0.27	[0.21; 0.34]	0.2%	0.9%
Tiwari et al., 2013	83	745	0.11	[0.09; 0.14]	1.2%	0.9%
Vouking et al., 2014	46	207	0.22	[0.17; 0.29]	0.2%	0.9%
Wumba et al., 2012	6	242	0.02	[0.01; 0.05]	1.6%	0.9%
Kaneshiro et al., 2019	87	252	0.35	[0.29; 0.41]	0.2%	0.9%
Alemayehu etal., 2020	74	383	0.19	[0.15; 0.24]	0.4%	0.9%
Chhangte et al., 2020	8	250	0.03	[0.01; 0.06]	1.3%	0.9%
McLellan et al., 2020	58	253	0.23	[0.18; 0.29]	0.2%	0.9%
Namaji et al., 2020	19	361	0.05	[0.03; 0.08]	1.1%	0.9%
Bayleyegn et al., 2021	32	255	0.13	[0.09; 0.17]	0.4%	0.9%
Feleke et al., 2022	23	223	0.10	[0.07; 0.15]	0.4%	0.9%
Ajayi et al., 2021	10	375	0.03	[0.01; 0.05]	2.2%	0.9%
PrayGod et al., 2022	176	1149	0.15	[0.13; 0.18]	1.4%	0.9%
**Common effect model**		**21202**	**0.06**	**[0.06; 0.06]**	**80.6%**	**--**
**Random effects model**			**0.19**	**[0.14; 0.24]**	**--**	**47.9%**
Heterogeneity: $I^2 = 99\%$, $\tau^2 = 0.0405$, $p = 0$						
**Common effect model**		**27808**	**0.07**	**[0.07; 0.07]**	**100.0%**	**--**
**Random effects model**			**0.20**	**[0.16; 0.24]**	**--**	**100.0%**

Heterogeneity: $I^2 = 99\%$, $\tau^2 = 0.0423$, $p = 0$

Test for subgroup differences (fixed effect): $\chi^2_1 = 257.89$, df = 1 (p < 0.0[illegible])

Test for subgroup differences (random effects): $\chi^2_1 = 0.24$, df = 1 ($p = 0.62$)

Figure S11B: Subgroup analysis of the prevalence of protozoan infections in people living with HIV by sample size

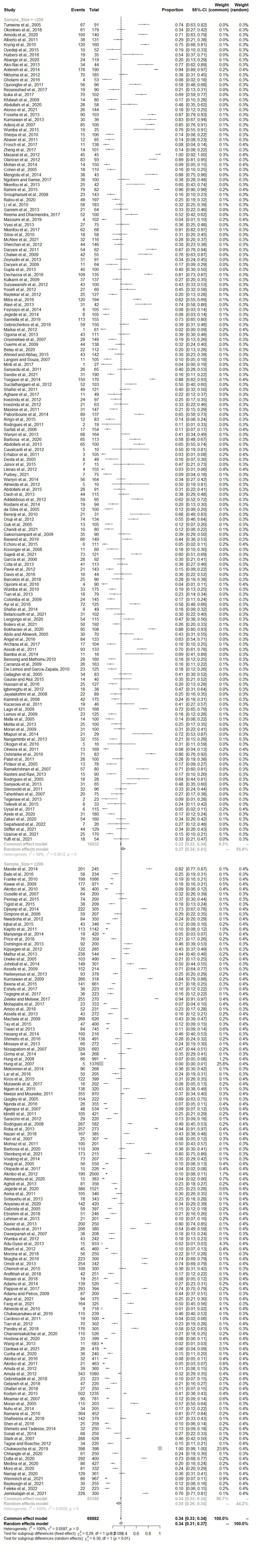

Figure S12A: Subgroup analysis of the prevalence of helminth infections in people living with HIV by publication year

Study	Events	Total	Proportion	95%-CI	Weight (common)	Weight (random)
**year = <2014**						
Abaver et al., 2011	9	85	0.11	[0.05; 0.19]	0.1%	0.9%
Abilo and Afework, 2005	18	70	0.26	[0.16; 0.38]	0.1%	0.8%
Adams et al., 2006	103	170	0.61	[0.53; 0.68]	0.1%	0.9%
Adamu and Petros, 2009	9	200	0.04	[0.02; 0.08]	0.7%	0.9%
Adesiji et al. 2007	12	100	0.12	[0.06; 0.20]	0.1%	0.9%
Agholi et al., 2013	4	356	0.01	[0.00; 0.03]	5.0%	0.9%
Akinbo and Omoregie, 2011	15	285	0.05	[0.03; 0.09]	0.9%	0.9%
Akinbo et al., 2011	125	463	0.27	[0.23; 0.31]	0.4%	0.9%
Akinbo et al., 2012	156	2000	0.08	[0.07; 0.09]	4.3%	0.9%
Asma et al., 2011	66	346	0.19	[0.15; 0.24]	0.3%	0.9%
Assefa et al., 2009	81	214	0.38	[0.31; 0.45]	0.1%	0.9%
Assefa et al., 2013	11	272	0.04	[0.02; 0.07]	1.1%	0.9%
Babatunde et al., 2010	39	90	0.43	[0.33; 0.54]	0.1%	0.8%
Cardoso et al., 2011	4	500	0.01	[0.00; 0.02]	9.8%	0.9%
Costiniuk et al., 2012	21	97	0.22	[0.14; 0.31]	0.1%	0.8%
Da Silva et al., 2005	19	100	0.19	[0.12; 0.28]	0.1%	0.8%
Dash et al., 2013	6	115	0.05	[0.02; 0.11]	0.4%	0.9%
Erikstrup et al., 2008	190	198	0.96	[0.92; 0.98]	0.8%	0.9%
Fekadu et al., 2013	118	343	0.34	[0.29; 0.40]	0.2%	0.9%
Guk et al., 2005	3	105	0.03	[0.01; 0.08]	0.6%	0.9%
Gupta et al., 2013	3	100	0.03	[0.01; 0.09]	0.5%	0.9%
Haileeyesus et al., 2013	59	378	0.16	[0.12; 0.20]	0.4%	0.9%
Hochberg et al., 2011	48	128	0.38	[0.29; 0.46]	0.1%	0.8%
Idindili et al., 2011	148	421	0.35	[0.31; 0.40]	0.3%	0.9%
Kulkarni et al., 2009	9	137	0.07	[0.03; 0.12]	0.3%	0.9%
Kallestrup et al., 2005	185	407	0.45	[0.41; 0.50]	0.3%	0.9%
Kawai et al., 2009	178	971	0.18	[0.16; 0.21]	1.0%	0.9%
Kipyegen et al., 2012	29	285	0.10	[0.07; 0.14]	0.5%	0.9%
Kurniawan et al., 2009	3	318	0.01	[0.00; 0.03]	5.3%	0.9%
Lehman et al., 2013	3	201	0.01	[0.00; 0.04]	2.1%	0.9%
Mascarello et al., 2011	15	138	0.11	[0.06; 0.17]	0.2%	0.9%
Mathur et al., 2013	34	544	0.06	[0.04; 0.09]	1.4%	0.9%
Meamar et al., 2007	5	781	0.01	[0.00; 0.01]	19.0%	0.9%
Mehta et al., 2013	3	100	0.03	[0.01; 0.09]	0.5%	0.9%
Missaye et al., 2013	19	272	0.07	[0.04; 0.11]	0.6%	0.9%
Modjarrad et al., 2005	74	297	0.25	[0.20; 0.30]	0.2%	0.9%
Modjarrad et al., 2005	54	111	0.49	[0.39; 0.58]	0.1%	0.8%
Mulu et al., 2013	87	220	0.40	[0.33; 0.46]	0.1%	0.9%
Ndhlovu et al., 2007	72	156	0.46	[0.38; 0.54]	0.1%	0.8%
Nielsen et al., 2007	18	34	0.53	[0.35; 0.70]	0.0%	0.7%
Ojurongbe et al., 2011	16	96	0.17	[0.10; 0.26]	0.1%	0.9%
Pavie et al., 2012	4	143	0.03	[0.01; 0.07]	0.8%	0.9%
Pinlaor et al., 2005	37	78	0.47	[0.36; 0.59]	0.0%	0.8%
Ramakrishnan et al., 2007	7	80	0.09	[0.04; 0.17]	0.2%	0.9%
Ramana et al., 2010	32	452	0.07	[0.05; 0.10]	1.1%	0.9%
Raytekar et al., 2012	2	127	0.02	[0.00; 0.06]	1.3%	0.9%
Rodrigues et al., 2008	366	582	0.63	[0.59; 0.67]	0.4%	0.9%
Roka et al., 2012	242	260	0.93	[0.89; 0.96]	0.6%	0.9%
Roka et al., 2013	246	273	0.90	[0.86; 0.93]	0.5%	0.9%
Saksirisampant et al., 2009	2	90	0.02	[0.00; 0.08]	0.6%	0.9%
Sanyaolu et al., 2011	12	65	0.18	[0.10; 0.30]	0.1%	0.8%
Sherpa et al., 2010	4	106	0.04	[0.01; 0.09]	0.5%	0.9%
Smith et al., 2008	21	122	0.17	[0.11; 0.25]	0.1%	0.9%
Suryawanshi et al., 2012	12	100	0.12	[0.06; 0.20]	0.1%	0.9%
Talaat et al., 2008	40	632	0.06	[0.05; 0.09]	1.7%	0.9%
Tian et al., 2012	14	302	0.05	[0.03; 0.08]	1.1%	0.9%
Tian et al., 2013	5	79	0.06	[0.02; 0.14]	0.2%	0.9%
Tiwari et al., 2013	83	745	0.11	[0.09; 0.14]	1.2%	0.9%
Vyas et al., 2013	2	75	0.03	[0.00; 0.09]	0.4%	0.9%
Wanyiri et al., 2013	42	164	0.26	[0.19; 0.33]	0.1%	0.9%
Wumba et al., 2010	12	175	0.07	[0.04; 0.12]	0.4%	0.9%
Wumba et al., 2012	6	242	0.02	[0.01; 0.05]	1.6%	0.9%
Zeynudin et al., 2013	29	91	0.32	[0.22; 0.42]	0.1%	0.8%
Common effect model		17187	0.07	[0.07; 0.07]	71.7%	--
Random effects model			0.21	[0.15; 0.27]	--	54.4%
Heterogeneity: I^2 = 100%, τ^2 = 0.0533, p = 0						
**year = ≥2014**						
Abossie and Petros, 2015	55	97	0.57	[0.46; 0.67]	0.1%	0.8%
Adeleke et al., 2015	57	231	0.25	[0.19; 0.31]	0.2%	0.9%
Ahmed and Abhay, 2015	14	142	0.10	[0.05; 0.16]	0.2%	0.9%
Barcelos et al., 2018	1	90	0.01	[0.00; 0.06]	1.3%	0.9%
Colombe et al., 2018	37	79	0.47	[0.36; 0.58]	0.0%	0.8%
Eshetu et al., 2017	36	223	0.16	[0.12; 0.22]	0.3%	0.9%
Feyisayo et al., 2014	4	105	0.04	[0.01; 0.09]	0.4%	0.9%
Gaurav and Atul, 2015	6	40	0.15	[0.06; 0.30]	0.0%	0.8%
Getaneh et al., 2018	15	220	0.07	[0.04; 0.11]	0.5%	0.9%
Gebretsadik et al., 2018	8	223	0.04	[0.02; 0.07]	1.0%	0.9%
Jegede et al., 2014	4	105	0.04	[0.01; 0.09]	0.4%	0.9%
Kroidl et al., 2016	69	187	0.37	[0.30; 0.44]	0.1%	0.9%
Lar et al., 2016	77	205	0.38	[0.31; 0.45]	0.1%	0.9%
Marti et al., 2017	60	305	0.20	[0.15; 0.25]	0.3%	0.9%
Mazigo et al., 2016	28	50	0.56	[0.41; 0.70]	0.0%	0.8%
Minti et al., 2017	1	27	0.04	[0.00; 0.19]	0.1%	0.9%
Mkhize et al., 2017	31	95	0.33	[0.23; 0.43]	0.1%	0.8%
Morawski et al., 2017	65	202	0.32	[0.26; 0.39]	0.1%	0.9%
Mtapuri et al., 2014	1	29	0.03	[0.00; 0.18]	0.1%	0.9%
Nsagha et al., 2016	34	300	0.11	[0.08; 0.15]	0.5%	0.9%
Nuhu et al., 2014	8	205	0.04	[0.02; 0.08]	0.8%	0.9%
Orji et al., 2017	24	84	0.29	[0.19; 0.39]	0.1%	0.8%
Oyedeji et al., 2015	1	52	0.02	[0.00; 0.10]	0.4%	0.9%
Paboriboune et al., 2014	116	137	0.85	[0.78; 0.90]	0.2%	0.9%
Pennap et al., 2015	103	200	0.52	[0.44; 0.59]	0.1%	0.9%
Plumelle et al., 2015	55	445	0.12	[0.09; 0.16]	0.6%	0.9%
Ringo et al., 2015	13	83	0.16	[0.09; 0.25]	0.1%	0.8%
Samie et al., 2014	62	151	0.41	[0.33; 0.49]	0.1%	0.8%
Shimelis et al., 2016	54	491	0.11	[0.08; 0.14]	0.8%	0.9%
Sonali et al., 2014	2	250	0.01	[0.00; 0.03]	4.9%	0.9%
Talaat et al., 2015	28	80	0.35	[0.25; 0.46]	0.1%	0.8%
Tegegne et al., 2017	36	223	0.16	[0.12; 0.22]	0.3%	0.9%
Tigist et al., 2015	57	209	0.27	[0.21; 0.34]	0.2%	0.9%
Vouking et al., 2014	46	207	0.22	[0.17; 0.29]	0.2%	0.9%
Gebrecherkos et al., 2019	26	150	0.17	[0.12; 0.24]	0.2%	0.9%
Kaneshiro et al., 2019	87	252	0.35	[0.29; 0.41]	0.2%	0.9%
Sannella et al., 2019	23	155	0.15	[0.10; 0.21]	0.2%	0.9%
Alemayehu etal., 2020	74	383	0.19	[0.15; 0.24]	0.4%	0.9%
Belay et al., 2020	4	112	0.04	[0.01; 0.09]	0.5%	0.9%
Chhangte et al., 2020	8	250	0.03	[0.01; 0.06]	1.3%	0.9%
Lengongo et al., 2020	9	115	0.08	[0.04; 0.14]	0.2%	0.9%
McLellan et al., 2020	58	253	0.23	[0.18; 0.29]	0.2%	0.9%
Namaji et al., 2020	19	361	0.05	[0.03; 0.08]	1.1%	0.9%
Rabiu et al., 2020	6	197	0.03	[0.01; 0.07]	1.0%	0.9%
Botero et al., 2021	2	192	0.01	[0.00; 0.04]	2.9%	0.9%
Bayleyegn et al., 2021	32	255	0.13	[0.09; 0.17]	0.4%	0.9%
Betancourth et al., 2021	3	102	0.03	[0.01; 0.08]	0.6%	0.9%
Feleke et al., 2022	23	223	0.10	[0.07; 0.15]	0.4%	0.9%
Chandi et al., 2021	6	80	0.07	[0.03; 0.16]	0.2%	0.9%
Ajayi et al., 2021	10	375	0.03	[0.01; 0.05]	2.2%	0.9%
Purbey., 2021	13	75	0.17	[0.10; 0.28]	0.1%	0.8%
Uzairue et al., 2021	51	170	0.30	[0.23; 0.37]	0.1%	0.9%
PrayGod et al., 2022	176	1149	0.15	[0.13; 0.18]	1.4%	0.9%
Common effect model		10621	0.08	[0.07; 0.08]	28.3%	--
Random effects model			0.19	[0.14; 0.23]	--	45.6%
Heterogeneity: I^2 = 98%, τ^2 = 0.0294, p = 0						
**Common effect model**		**27808**	**0.07**	**[0.07; 0.07]**	**100.0%**	**--**
**Random effects model**			**0.20**	**[0.16; 0.24]**	**--**	**100.0%**

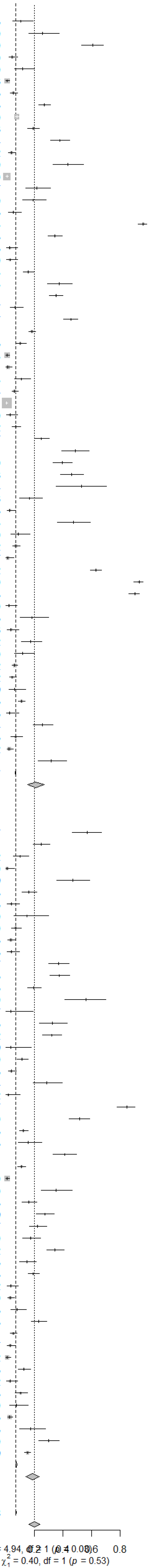

Heterogeneity: I^2 = 99%, τ^2 = 0.0423, p = 0
Test for subgroup differences (fixed effect): χ^2_1 = 4.94, df = 1 (p = 0.03)
Test for subgroup differences (random effects): χ^2_1 = 0.40, df = 1 (p = 0.53)

Figure S12B: Subgroup analysis of the prevalence of protozoan infections in people living with Hiv by publication year

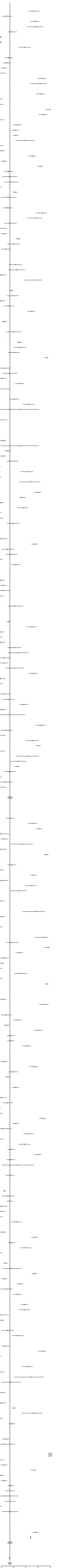

Study	Events	Total	Proportion	95%-CI	Weight (common)	Weight (random)
**year = <2014**						
Tumwine et al., 2005	67	91	0.74	[0.63; 0.82]	0.0%	0.3%
Alberto et al., 2011	38	131	0.29	[0.21; 0.38]	0.0%	0.3%
Kiying et al., 2010	120	160	0.75	[0.68; 0.81]	0.1%	0.3%
Ako-Nai et al., 2013	34	44	0.77	[0.62; 0.89]	0.0%	0.3%
Nkhoma et al., 2012	70	185	0.38	[0.31; 0.45]	0.1%	0.3%
Franke et al., 2010	199	1066	0.19	[0.16; 0.21]	0.5%	0.4%
Kawai et al., 2009	177	971	0.18	[0.16; 0.21]	0.5%	0.4%
Ojurongbe et al., 2011	56	96	0.58	[0.48; 0.68]	0.0%	0.3%
Akinbo et al., 2010	36	400	0.09	[0.06; 0.12]	0.4%	0.4%
Goselle et al., 2007	64	200	0.32	[0.26; 0.39]	0.1%	0.3%
Simpore et al., 2006	59	207	0.29	[0.22; 0.35]	0.1%	0.3%
Nwadioha et al., 2012	84	350	0.24	[0.20; 0.29]	0.1%	0.4%
Kfutwah et al., 2009	14	80	0.17	[0.10; 0.28]	0.0%	0.3%
Fisseha et al., 2013	90	103	0.87	[0.79; 0.93]	0.1%	0.3%
Kurniawan et al., 2013	30	36	0.83	[0.67; 0.94]	0.0%	0.3%
Kapito et al., 2011	113	1142	0.10	[0.08; 0.12]	1.0%	0.4%
Adesij et al. 2007	85	100	0.85	[0.76; 0.91]	0.1%	0.3%
Sherpa et al., 2010	15	106	0.14	[0.08; 0.22]	0.1%	0.3%
Abaver et al., 2011	12	85	0.14	[0.08; 0.23]	0.1%	0.3%
Masarat et al., 2012	45	45	1.00	[0.92; 1.00]	0.3%	0.4%
Olaniran et al., 2012	83	93	0.89	[0.81; 0.95]	0.1%	0.3%
Cohen et al., 2005	18	110	0.16	[0.10; 0.25]	0.1%	0.3%
Domingos et al., 2013	92	200	0.46	[0.39; 0.53]	0.1%	0.3%
Kipyegen et al., 2012	122	285	0.43	[0.37; 0.49]	0.1%	0.3%
Mathur et al., 2013	238	544	0.44	[0.40; 0.48]	0.2%	0.4%
Nkenfou et al., 2013	25	42	0.60	[0.43; 0.74]	0.0%	0.3%
Srisuphanunt et al., 2008	23	143	0.16	[0.10; 0.23]	0.1%	0.3%
Uneke et al., 2005	103	490	0.21	[0.17; 0.25]	0.2%	0.4%
Assefa et al., 2009	152	214	0.71	[0.64; 0.77]	0.1%	0.3%
Haileeyesus et al., 2013	93	378	0.25	[0.20; 0.29]	0.2%	0.4%
Kurniawan et al., 2009	266	318	0.84	[0.79; 0.88]	0.2%	0.4%
Li et al., 2010	58	183	0.32	[0.25; 0.39]	0.1%	0.3%
Ganiem et al., 2013	21	64	0.33	[0.22; 0.46]	0.0%	0.3%
Vyas et al., 2013	27	75	0.36	[0.25; 0.48]	0.0%	0.3%
Assefa et al., 2013	43	272	0.16	[0.12; 0.21]	0.2%	0.4%
Machala et al., 2009	268	626	0.43	[0.39; 0.47]	0.2%	0.4%
Sitoe et al., 2010	18	58	0.31	[0.20; 0.45]	0.0%	0.3%
Tiwari et al. 2013	84	745	0.11	[0.09; 0.14]	0.6%	0.4%
Sherchan et al., 2012	44	146	0.30	[0.23; 0.38]	0.1%	0.3%
Daryani et al., 2011	54	62	0.87	[0.76; 0.94]	0.0%	0.3%
Chalwe et al., 2009	42	55	0.76	[0.63; 0.87]	0.0%	0.3%
Zeynudin et al., 2013	31	91	0.34	[0.24; 0.45]	0.0%	0.3%
Daryani et al., 2009	11	64	0.17	[0.09; 0.29]	0.0%	0.3%
Missaye et al., 2013	66	272	0.24	[0.19; 0.30]	0.1%	0.4%
Nissapatorn et al., 2007	329	693	0.47	[0.44; 0.51]	0.2%	0.4%
Gupta et al., 2013	40	100	0.40	[0.30; 0.50]	0.0%	0.3%
Kulkarni et al., 2009	37	137	0.27	[0.20; 0.35]	0.1%	0.3%
Hung et al., 2008	66	991	0.07	[0.05; 0.08]	1.2%	0.4%
Hung et al., 2007	5	1376	0.00	[0.00; 0.01]	25.8%	0.4%
Suryawanshi et al., 2012	43	100	0.43	[0.33; 0.53]	0.0%	0.3%
Yosefi et al., 2012	27	60	0.45	[0.32; 0.58]	0.0%	0.3%
Raytekar et al., 2012	25	127	0.20	[0.13; 0.28]	0.1%	0.3%
Alavi et al., 2013	31	42	0.74	[0.58; 0.86]	0.0%	0.3%
Maikai et al., 2012	1	61	0.02	[0.00; 0.09]	0.2%	0.4%
Nweze and Mouneke, 2011	355	970	0.37	[0.34; 0.40]	0.3%	0.4%
Ogoina et al., 2013	43	111	0.39	[0.30; 0.48]	0.0%	0.3%
Onyenekwe et al., 2007	28	149	0.19	[0.13; 0.26]	0.1%	0.3%
Ouermi et al., 2009	44	138	0.32	[0.24; 0.40]	0.0%	0.3%
Quigley et al., 2005	154	222	0.69	[0.63; 0.75]	0.1%	0.3%
Ajjampur et al., 2007	48	534	0.09	[0.07; 0.12]	0.5%	0.4%
Idindili et al., 2011	105	421	0.25	[0.21; 0.29]	0.2%	0.4%
Langoni and Souza, 2007	11	105	0.10	[0.05; 0.18]	0.1%	0.3%
Sanyaolu et al., 2011	26	65	0.40	[0.28; 0.53]	0.0%	0.3%
Saracino et al., 2012	29	220	0.13	[0.09; 0.18]	0.1%	0.4%
Rodrigues et al., 2008	287	582	0.49	[0.45; 0.53]	0.2%	0.4%
Sucilathangam et al., 2012	52	103	0.50	[0.40; 0.60]	0.0%	0.3%
Shafiei et al., 2011	49	121	0.40	[0.32; 0.50]	0.0%	0.3%
Roka et al., 2013	257	273	0.94	[0.91; 0.97]	0.4%	0.4%
Hari et al., 2007	25	307	0.08	[0.05; 0.12]	0.3%	0.4%
Iroezindu et al., 2012	24	97	0.25	[0.17; 0.35]	0.0%	0.3%
Iroezindu et al., 2012	21	63	0.33	[0.22; 0.46]	0.0%	0.3%
Masese et al., 2011	31	147	0.21	[0.15; 0.29]	0.1%	0.3%
Mohraz et al., 2011	100	201	0.50	[0.43; 0.57]	0.1%	0.3%
Rodrigues et al., 2011	2	19	0.11	[0.01; 0.33]	0.0%	0.3%
Sarfati et al., 2006	17	154	0.11	[0.07; 0.17]	0.1%	0.4%
Wanyiri et al., 2013	68	164	0.41	[0.34; 0.49]	0.1%	0.3%
Abdollahi et al., 2013	65	100	0.65	[0.55; 0.74]	0.0%	0.3%
Cavalcanti et al., 2012	5	10	0.50	[0.19; 0.81]	0.0%	0.3%
Erhabor et al., 2011	3	105	0.03	[0.01; 0.08]	0.2%	0.4%
Desta et al., 2005	8	49	0.16	[0.07; 0.30]	0.0%	0.3%
Hung et al., 2005	56	550	0.10	[0.08; 0.13]	0.4%	0.4%
Llenas et al., 2012	4	155	0.03	[0.01; 0.06]	0.4%	0.4%
Akinbo et al., 2012	190	2000	0.10	[0.08; 0.11]	1.7%	0.4%
Agholi et al., 2013	81	356	0.23	[0.18; 0.27]	0.2%	0.4%
Almeida et al., 2012	5	10	0.50	[0.19; 0.81]	0.0%	0.3%
Asma et al., 2011	105	346	0.30	[0.26; 0.35]	0.1%	0.4%
Sintayehu et al., 2013	78	343	0.23	[0.18; 0.28]	0.1%	0.4%
Dash et al., 2013	44	115	0.38	[0.29; 0.48]	0.0%	0.3%
Gabriela et al., 2005	59	397	0.15	[0.12; 0.19]	0.2%	0.4%
Addebbous et al., 2012	59	95	0.62	[0.52; 0.72]	0.0%	0.3%
da Silva et al., 2005	12	100	0.12	[0.06; 0.20]	0.1%	0.3%
Berenji et al., 2010	21	31	0.68	[0.49; 0.83]	0.0%	0.3%
Lehman et al., 2013	21	201	0.10	[0.07; 0.16]	0.2%	0.4%
Xavier et al., 2013	200	250	0.80	[0.74; 0.85]	0.1%	0.4%
Osunkalu et al., 2011	206	380	0.54	[0.49; 0.59]	0.1%	0.4%
Davarpanah et al., 2007	38	208	0.18	[0.13; 0.24]	0.1%	0.3%
Osuji et al., 2012	74	134	0.55	[0.46; 0.64]	0.0%	0.3%
Guk et al., 2005	13	105	0.12	[0.07; 0.20]	0.1%	0.3%
Wumba et al., 2012	43	242	0.18	[0.13; 0.23]	0.1%	0.4%
Saksirisampant et al., 2009	35	90	0.39	[0.29; 0.50]	0.0%	0.3%
Adu-Gyasi et al., 2013	15	933	0.02	[0.01; 0.03]	4.3%	0.4%
Bharti et al., 2012	45	460	0.10	[0.07; 0.13]	0.4%	0.4%
Kissinger et al., 2008	11	60	0.18	[0.10; 0.30]	0.0%	0.3%
Omoti et al., 2013	254	342	0.74	[0.69; 0.79]	0.1%	0.4%
Garcia et al., 2006	28	92	0.30	[0.21; 0.41]	0.0%	0.3%
Cota et al., 2013	41	113	0.36	[0.27; 0.46]	0.0%	0.3%
Pavie et al., 2012	21	143	0.15	[0.09; 0.22]	0.1%	0.3%
Adamu and Petros, 2009	87	200	0.44	[0.37; 0.51]	0.1%	0.3%
Almeida et al., 2010	9	716	0.01	[0.01; 0.02]	4.1%	0.4%
Cardoso et al., 2011	19	500	0.04	[0.02; 0.06]	1.0%	0.4%
Wumba et al., 2010	33	175	0.19	[0.13; 0.25]	0.1%	0.3%
Tian et al., 2012	70	302	0.23	[0.19; 0.28]	0.1%	0.4%
Tian et al., 2013	18	79	0.23	[0.14; 0.34]	0.0%	0.3%
Colomba et al., 2009	24	145	0.17	[0.11; 0.24]	0.1%	0.3%
Wang et al., 2013	11	683	0.02	[0.01; 0.03]	3.1%	0.4%
Abilo and Afework, 2005	30	70	0.43	[0.31; 0.55]	0.0%	0.3%
Akinbo et al., 2011	21	463	0.05	[0.03; 0.07]	0.8%	0.4%
Amuta et al., 2012	39	360	0.11	[0.08; 0.15]	0.3%	0.4%
Amuta et al., 2012	343	1080	0.32	[0.29; 0.35]	0.4%	0.4%
Assob et al., 2011	93	133	0.70	[0.61; 0.78]	0.0%	0.3%
Bessong and Mathomu 2010	29	160	0.18	[0.12; 0.25]	0.1%	0.3%
Carranza et al., 2009	26	163	0.16	[0.11; 0.22]	0.1%	0.3%
De Lemos and Garcia-Zapata, 2010	23	125	0.18	[0.12; 0.26]	0.1%	0.3%
Gallagher et al., 2005	34	83	0.41	[0.30; 0.52]	0.0%	0.3%
Igbeneghu et al., 2012	18	38	0.47	[0.31; 0.64]	0.0%	0.3%
Jayalakshmi et al., 2008	22	89	0.25	[0.16; 0.35]	0.0%	0.3%
Kammili et al., 2008	42	175	0.24	[0.18; 0.31]	0.1%	0.3%
Kucerova et al., 2011	19	46	0.41	[0.27; 0.57]	0.0%	0.3%
Lago et al., 2009	121	168	0.72	[0.65; 0.79]	0.1%	0.3%
Lemos et al., 2009	23	125	0.18	[0.12; 0.26]	0.1%	0.3%
Malla et al., 2005	14	100	0.14	[0.08; 0.22]	0.1%	0.3%
Meamar et al., 2007	90	781	0.12	[0.09; 0.14]	0.6%	0.4%
Mehta et al., 2013	25	100	0.25	[0.17; 0.35]	0.0%	0.3%
Moran et al., 2009	31	100	0.31	[0.22; 0.41]	0.0%	0.3%
Moran et al., 2005	115	203	0.57	[0.50; 0.64]	0.1%	0.3%
Nangammbi et al., 2013	32	155	0.21	[0.15; 0.28]	0.1%	0.3%
Okogun et al., 2010	5	16	0.31	[0.11; 0.59]	0.0%	0.3%
Oliveira et al., 2011	13	169	0.08	[0.04; 0.13]	0.2%	0.4%
Oshinaike et al., 2010	71	83	0.86	[0.76; 0.92]	0.0%	0.3%
Patel et al., 2011	28	100	0.28	[0.19; 0.38]	0.0%	0.3%
Pinlaor et al., 2005	13	78	0.17	[0.09; 0.27]	0.0%	0.3%
Ramakrishnan et al., 2007	57	80	0.71	[0.60; 0.81]	0.0%	0.3%
Ramana et al., 2010	364	452	0.81	[0.77; 0.84]	0.2%	0.4%
Rashmi and Ravi, 2013	15	90	0.17	[0.10; 0.26]	0.0%	0.3%
Rodrigues et al., 2005	18	28	0.64	[0.44; 0.81]	0.0%	0.3%
Sanyaolu et al., 2013	31	65	0.48	[0.35; 0.60]	0.0%	0.3%
Stark et al., 2007	288	628	0.46	[0.42; 0.50]	0.2%	0.4%
Stensvold et al., 2011	32	96	0.33	[0.24; 0.44]	0.0%	0.3%
Tagoe and Boachie, 2012	34	220	0.15	[0.11; 0.21]	0.1%	0.4%
Taherkhani et al., 2007	20	75	0.27	[0.17; 0.38]	0.0%	0.3%
Tegenaw et al., 2013	2	23	0.09	[0.01; 0.28]	0.0%	0.3%
Common effect model		38216	0.09	[0.09; 0.09]	58.1%	--
Random effects model			0.34	[0.30; 0.38]	--	51.9%
Heterogeneity: $I^2 = 99\%$, $\tau^2 = 0.0576$, $p = 0$						
**year = ≥2014**						
Okonkwo et al., 2018	61	179	0.34	[0.27; 0.42]	0.1%	0.3%
Amodu et al., 2020	100	140	0.71	[0.63; 0.79]	0.1%	0.3%
Marete et al., 2014	201	245	0.82	[0.77; 0.87]	0.1%	0.4%
Oyedeji et al., 2015	10	52	0.19	[0.10; 0.33]	0.0%	0.3%
Bate et al., 2016	58	234	0.25	[0.19; 0.31]	0.1%	0.3%
Huibers et al., 2018	19	35	0.54	[0.37; 0.71]	0.0%	0.3%
Abange et al., 2020	24	119	0.20	[0.13; 0.29]	0.1%	0.3%
Anteneh et al., 2014	178	190	0.94	[0.89; 0.97]	0.2%	0.4%
Gholami et al., 2016	4	53	0.08	[0.02; 0.18]	0.1%	0.3%
Pennap et al., 2015	74	200	0.37	[0.30; 0.44]	0.1%	0.3%
Tigist et al., 2015	38	209	0.18	[0.13; 0.24]	0.1%	0.3%
Djieyep et al., 2014	222	305	0.73	[0.67; 0.78]	0.1%	0.4%
Rezanezhad et al., 2017	19	90	0.21	[0.13; 0.31]	0.0%	0.3%
Izuka et al., 2017	70	102	0.69	[0.59; 0.77]	0.0%	0.3%
Abdullahi et al., 2020	28	58	0.48	[0.35; 0.62]	0.0%	0.3%
Iqbal et al., 2015	43	346	0.12	[0.09; 0.16]	0.2%	0.4%
Alonso et al., 2021	26	144	0.18	[0.12; 0.25]	0.1%	0.3%
Manyanga et al., 2014	19	420	0.05	[0.03; 0.07]	0.7%	0.4%
Wumba et al., 2015	19	25	0.76	[0.55; 0.91]	0.0%	0.3%
Price et al., 2018	76	359	0.21	[0.17; 0.26]	0.2%	0.4%
Frosch et al., 2017	11	138	0.08	[0.04; 0.14]	0.1%	0.4%
Zheng et al., 2017	14	101	0.14	[0.08; 0.22]	0.1%	0.3%
Mohan et al., 2014	14	150	0.09	[0.05; 0.15]	0.1%	0.4%
Mengistu et al., 2014	38	43	0.88	[0.75; 0.96]	0.0%	0.3%
Ngobeni and Samie, 2017	38	100	0.38	[0.28; 0.48]	0.0%	0.3%
Rahimi et al., 2015	79	82	0.96	[0.90; 0.99]	0.2%	0.4%
Johnbull et al., 2014	149	301	0.50	[0.44; 0.55]	0.1%	0.3%
Rabiu et al., 2020	49	197	0.25	[0.19; 0.32]	0.1%	0.3%
Beena et al., 2015	141	661	0.21	[0.18; 0.25]	0.3%	0.4%
Eshetu et al., 2017	36	223	0.16	[0.12; 0.22]	0.1%	0.4%
Reema and Dharmendra, 2017	52	100	0.52	[0.42; 0.62]	0.0%	0.3%
Tegegne et al., 2017	36	223	0.16	[0.12; 0.22]	0.1%	0.4%
Zeleke and Melsew, 2017	255	270	0.94	[0.91; 0.97]	0.4%	0.4%
Masoumi et al., 2019	4	102	0.04	[0.01; 0.10]	0.2%	0.4%
Mohapatra et al., 2017	23	333	0.07	[0.04; 0.10]	0.4%	0.4%
Amoo et al., 2018	52	231	0.23	[0.17; 0.28]	0.1%	0.3%
Nkenfou et al., 2017	62	68	0.91	[0.82; 0.97]	0.1%	0.3%
Tay et al., 2015	47	400	0.12	[0.09; 0.15]	0.3%	0.4%
McAtee et al., 2021	32	116	0.28	[0.20; 0.37]	0.0%	0.3%
Hasang et al., 2014	100	216	0.46	[0.40; 0.53]	0.1%	0.3%
Shimelis et al., 2016	138	491	0.28	[0.24; 0.32]	0.2%	0.4%
Dechassa et al., 2016	109	135	0.81	[0.73; 0.87]	0.1%	0.3%
Girma et al., 2014	94	268	0.35	[0.29; 0.41]	0.1%	0.3%
Mekonnen et al., 2014	96	268	0.36	[0.30; 0.42]	0.1%	0.3%
Mitra et al., 2016	120	194	0.62	[0.55; 0.69]	0.1%	0.3%
Feyisayo et al., 2014	8	105	0.08	[0.03; 0.14]	0.1%	0.3%
Jegede et al., 2014	8	105	0.08	[0.03; 0.14]	0.1%	0.3%
Lar et al., 2016	50	205	0.24	[0.19; 0.31]	0.1%	0.3%
Sannella et al., 2019	113	155	0.73	[0.65; 0.80]	0.1%	0.3%
Gebrecherkos et al., 2019	59	150	0.39	[0.31; 0.48]	0.0%	0.3%
Kiros et al., 2015	122	399	0.31	[0.26; 0.35]	0.1%	0.4%
Morawski et al., 2017	16	202	0.08	[0.05; 0.13]	0.2%	0.4%
Ngum et al., 2015	138	320	0.43	[0.38; 0.49]	0.1%	0.3%
Njunda et al., 2016	26	355	0.07	[0.05; 0.11]	0.4%	0.4%
Belay et al., 2020	22	112	0.20	[0.13; 0.28]	0.1%	0.3%
Ahmed and Abhay, 2015	43	142	0.30	[0.23; 0.39]	0.1%	0.3%
Minti et al., 2017	1	27	0.04	[0.00; 0.19]	0.0%	0.3%
Sandie et al., 2021	31	190	0.16	[0.11; 0.22]	0.1%	0.3%
Tsegaye et al., 2014	150	170	0.88	[0.82; 0.93]	0.1%	0.4%
Nazari et al., 2018	167	385	0.43	[0.38; 0.48]	0.1%	0.4%
Aghaee et al., 2017	11	49	0.22	[0.12; 0.37]	0.0%	0.3%
Paboriboune et al., 2014	89	137	0.65	[0.56; 0.73]	0.0%	0.3%
Ringo et al., 2015	12	83	0.14	[0.08; 0.24]	0.0%	0.3%
Barbosa et al., 2020	65	113	0.58	[0.48; 0.67]	0.0%	0.3%
Barbosa et al., 2020	110	309	0.36	[0.30; 0.41]	0.1%	0.3%
Steinberg et al., 2021	173	215	0.80	[0.75; 0.86]	0.1%	0.3%
Vouking et al., 2014	73	207	0.35	[0.29; 0.42]	0.1%	0.3%
Júnior et al., 2015	7	15	0.47	[0.21; 0.73]	0.0%	0.3%
Purbey., 2021	7	75	0.09	[0.04; 0.18]	0.1%	0.3%
Wanyiri et al., 2014	56	164	0.34	[0.27; 0.42]	0.1%	0.3%
Olopade et al., 2017	10	226	0.04	[0.02; 0.08]	0.4%	0.4%
Alemayehu et al., 2020	15	383	0.04	[0.02; 0.06]	0.7%	0.4%
Jegede et al., 2020	386	1521	0.25	[0.23; 0.28]	0.6%	0.4%
Abdollahi et al., 2015	28	91	0.31	[0.22; 0.41]	0.0%	0.3%
Anyanwu et al., 2020	142	420	0.34	[0.29; 0.39]	0.1%	0.4%
Rostami et al., 2014	19	94	0.20	[0.13; 0.30]	0.0%	0.3%
Ebrahim et al., 2018	51	246	0.21	[0.16; 0.26]	0.1%	0.4%
Chandi et al., 2021	10	80	0.12	[0.06; 0.22]	0.1%	0.3%
Bavand et al., 2019	66	149	0.44	[0.36; 0.53]	0.0%	0.3%
Echoru et al., 2015	6	111	0.05	[0.02; 0.11]	0.2%	0.4%
Morona et al., 2018	56	250	0.22	[0.17; 0.28]	0.1%	0.3%
Nsagha et al., 2016	223	300	0.74	[0.69; 0.79]	0.1%	0.4%
Chemoh et al., 2015	109	300	0.36	[0.31; 0.42]	0.1%	0.3%
Sajedi et al., 2021	73	121	0.60	[0.51; 0.69]	0.0%	0.3%
Arefkhah et al., 2018	42	251	0.17	[0.12; 0.22]	0.1%	0.4%
Rezaei et al., 2018	19	251	0.08	[0.05; 0.12]	0.3%	0.4%
Adamu et al., 2014	139	520	0.27	[0.23; 0.31]	0.2%	0.4%
Davis et al., 2016	16	44	0.36	[0.22; 0.52]	0.0%	0.3%
Pappoe et al., 2017	293	394	0.74	[0.70; 0.79]	0.2%	0.4%
Ajayi et al., 2021	94	375	0.25	[0.21; 0.30]	0.1%	0.4%
Fang et al., 2021	164	325	0.50	[0.45; 0.56]	0.1%	0.3%
Barcelos et al., 2018	25	90	0.28	[0.19; 0.38]	0.0%	0.3%
Chukwuanukwu et al., 2016	110	239	0.46	[0.40; 0.53]	0.1%	0.3%
Ojuromi et al., 2016	4	90	0.04	[0.01; 0.11]	0.1%	0.4%
Pandey et al., 2018	176	305	0.58	[0.52; 0.63]	0.1%	0.3%
Ayi et al., 2016	72	125	0.58	[0.48; 0.66]	0.0%	0.3%
Shafiei et al., 2014	9	49	0.18	[0.09; 0.32]	0.0%	0.3%
Charoensakulchai et al., 2020	110	526	0.21	[0.18; 0.25]	0.2%	0.4%
Hoshina et al., 2020	33	399	0.08	[0.06; 0.11]	0.4%	0.4%
Betancourth et al., 2021	31	102	0.30	[0.22; 0.40]	0.0%	0.3%
Lengongo et al., 2020	54	115	0.47	[0.38; 0.56]	0.0%	0.3%
Dankwa et al., 2021	26	418	0.06	[0.04; 0.09]	0.5%	0.4%
Botero et al., 2021	50	192	0.26	[0.20; 0.33]	0.1%	0.3%
Bokharaei et al., 2020	95	108	0.88	[0.80; 0.93]	0.1%	0.3%
Cunha et al., 2020	36	240	0.15	[0.11; 0.20]	0.1%	0.4%
Akinbo et al., 2016	32	411	0.08	[0.05; 0.11]	0.4%	0.4%
Angal et al., 2016	84	133	0.63	[0.54; 0.71]	0.0%	0.3%
Archana et al., 2017	17	104	0.16	[0.10; 0.25]	0.1%	0.3%
Bamba et al., 2014	11	16	0.69	[0.41; 0.89]	0.0%	0.3%
Gaurav and Atul, 2015	14	40	0.35	[0.21; 0.52]	0.0%	0.3%
Gebretsadik et al., 2018	23	223	0.10	[0.07; 0.15]	0.2%	0.4%
Getaneh et al., 2018	47	220	0.21	[0.16; 0.27]	0.1%	0.3%
Ghafari et al., 2018	27	250	0.11	[0.07; 0.15]	0.2%	0.4%
Hussain et al., 2016	25	127	0.20	[0.13; 0.28]	0.1%	0.3%
Kodym et al., 2015	502	1235	0.41	[0.38; 0.43]	0.4%	0.4%
Mtapuri et al., 2014	21	29	0.72	[0.53; 0.87]	0.0%	0.3%
Nuhu et al., 2014	34	205	0.17	[0.12; 0.22]	0.1%	0.4%
Shafieenia et al., 2018	142	379	0.37	[0.33; 0.43]	0.1%	0.4%
Shen et al., 2016	25	259	0.10	[0.06; 0.14]	0.2%	0.4%
Shimelis and Tadesse, 2014	32	250	0.13	[0.09; 0.18]	0.2%	0.4%
Sonali et al., 2014	68	250	0.27	[0.22; 0.33]	0.1%	0.3%
Tellevik et al., 2015	8	33	0.24	[0.11; 0.42]	0.0%	0.3%
Uysal et al., 2017	6	115	0.05	[0.02; 0.11]	0.2%	0.4%
Chukwuocha et al., 2019	398	398	1.00	[0.99; 1.00]	23.8%	0.4%
Ayele et al., 2020	31	180	0.17	[0.12; 0.24]	0.1%	0.3%
Chhangte et al., 2020	61	250	0.24	[0.19; 0.30]	0.1%	0.3%
Dutta et al., 2020	292	400	0.73	[0.68; 0.77]	0.2%	0.4%
Medina et al., 2020	86	427	0.20	[0.16; 0.24]	0.2%	0.4%
Moro et al., 2020	81	332	0.24	[0.20; 0.29]	0.1%	0.4%
Namaji et al., 2020	129	361	0.36	[0.31; 0.41]	0.1%	0.4%
Zakari et al., 2020	54	160	0.34	[0.26; 0.42]	0.1%	0.3%
Sepahvand et al., 2022	7	26	0.27	[0.12; 0.48]	0.0%	0.3%
Stiffler et al., 2021	44	129	0.34	[0.26; 0.43]	0.0%	0.3%
Uzairue et al., 2021	25	170	0.15	[0.10; 0.21]	0.1%	0.3%
Yulfi et al., 2021	18	54	0.33	[0.21; 0.47]	0.0%	0.3%
Weinreich et al., 2021	86	967	0.09	[0.07; 0.11]	0.9%	0.4%
Bayleyegn et al., 2021	30	255	0.12	[0.08; 0.16]	0.2%	0.4%
Feleke et al., 2022	22	223	0.10	[0.06; 0.15]	0.2%	0.4%
Jemikalajah et al., 2021	228	300	0.76	[0.71; 0.81]	0.1%	0.4%
Common effect model		31666	0.68	[0.68; 0.68]	41.9%	--
Random effects model			0.34	[0.30; 0.38]	--	48.1%
Heterogeneity: $I^2 = 100\%$, $\tau^2 = 0.0625$, $p = 0$						
**Common effect model**		**69882**	**0.34**	**[0.33; 0.34]**	**100.0%**	**--**
**Random effects model**			**0.34**	**[0.31; 0.37]**	**--**	**100.0%**

Heterogeneity: $I^2 = 100\%$, $\tau^2 = 0.0597$, $p = 0$

Test for subgroup differences (fixed effect): $\chi^2_1 = 113468.85$, df = 1 (p [illegible])

Test for subgroup differences (random effects): $\chi^2_1 = 0.00$, df = 1 ($p = 0.95$)

Figure S13A: Funnel plot assessing distribution symmetry for the pooled prevalence of helminth in people living with viruses from 135 studies

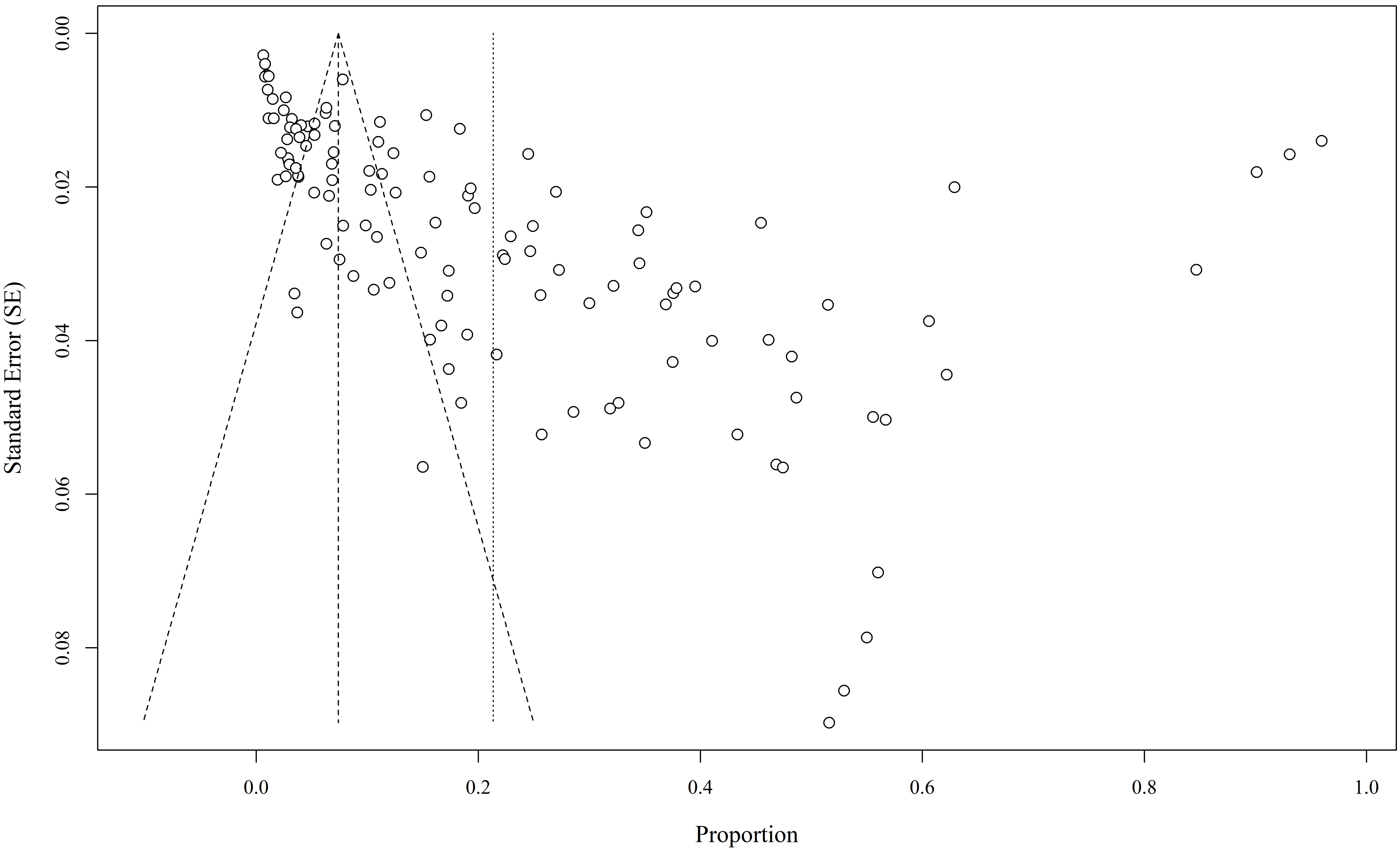

Figure S13B: Funnel plot assessing distribution symmetry for the pooled prevalence of protozoan in people living with viruses from 323 studies

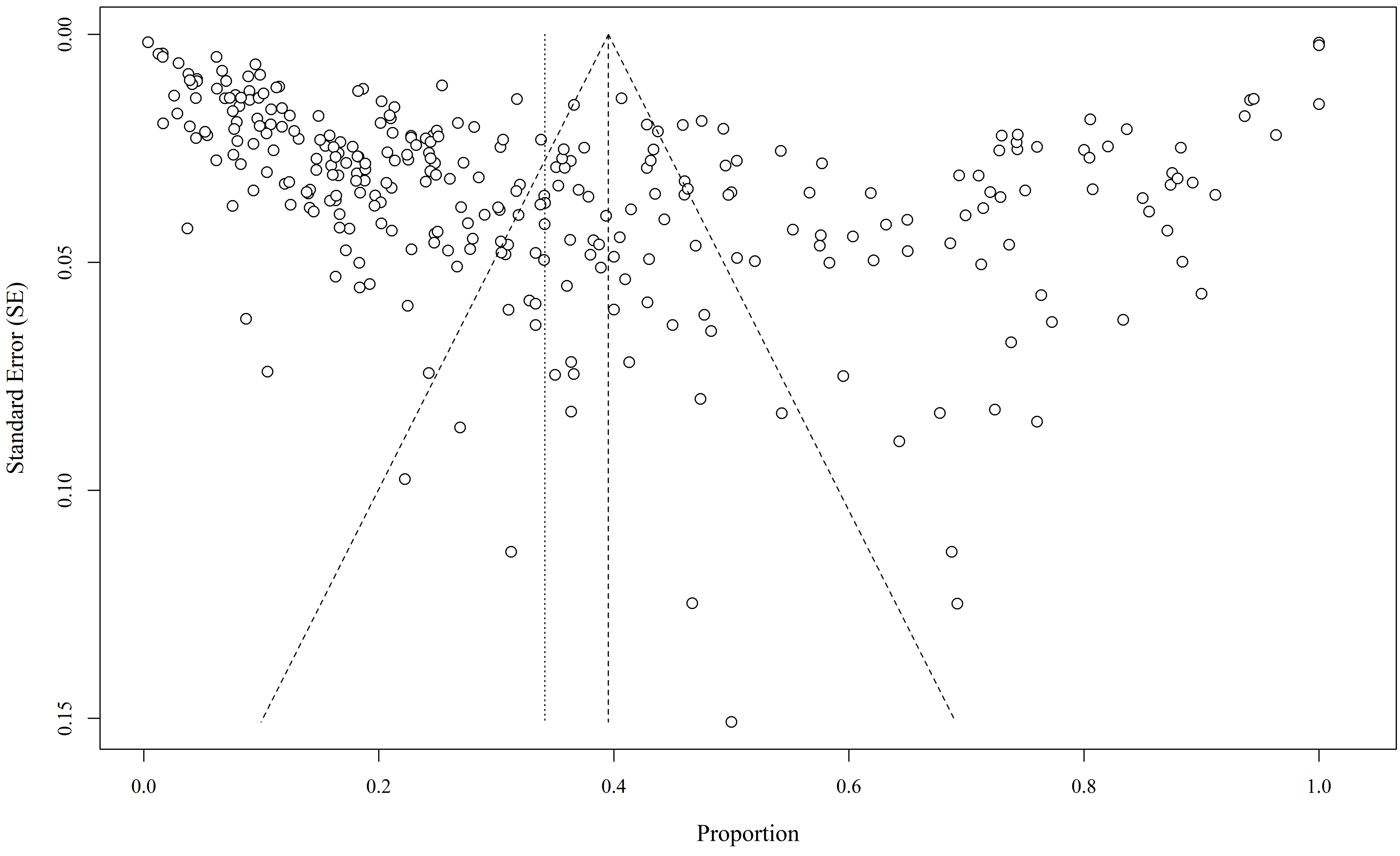

Table S1: PRISMA Checklist

Section and Topic	Item #	Checklist item	Location where item is reported
**TITLE**			
Title	1	Identify the report as a systematic review.	Title
**ABSTRACT**			
Abstract	2	See the PRISMA 2020 for Abstracts checklist.	Abstract
**INTRODUCTION**			
Rationale	3	Describe the rationale for the review in the context of existing knowledge.	Introduction, paragraphs 1-3
Objectives	4	Provide an explicit statement of the objective(s) or question(s) the review addresses.	Introduction, paragraph 3
**METHODS**			
Eligibility criteria	5	Specify the inclusion and exclusion criteria for the review and how studies were grouped for the syntheses.	Methods, paragraph 1; Supplementary Materials, Text S1
Information sources	6	Specify all databases, registers, websites, organisations, reference lists and other sources searched or consulted to identify studies. Specify the date when each source was last searched or consulted.	Methods, paragraph 1
Search strategy	7	Present the full search strategies for all databases, registers and websites, including any filters and limits used.	Methods, paragraph 1; Supplementary Materials, Text S1
Selection process	8	Specify the methods used to decide whether a study met the inclusion criteria of the review, including how many reviewers screened each record and each report retrieved, whether they worked independently, and if applicable, details of automation tools used in the process.	Methods, paragraph 1
Data collection process	9	Specify the methods used to collect data from reports, including how many reviewers collected data from each report, whether they worked independently, any processes for obtaining or confirming data from study investigators, and if applicable, details of automation tools used in the process.	Methods, paragraph 1
Data items	10a	List and define all outcomes for which data were sought. Specify whether all results that were compatible with each outcome domain in each study were sought (e.g. for all measures, time points, analyses), and if not, the methods used to decide which results to collect.	Methods, paragraphs 2, 3
	10b	List and define all other variables for which data were sought (e.g. participant and intervention characteristics, funding sources). Describe any assumptions made about any missing or unclear information.	Methods, paragraphs 2, 3
Study risk of bias assessment	11	Specify the methods used to assess risk of bias in the included studies, including details of the tool(s) used, how many reviewers assessed each study and whether they worked independently, and if applicable, details of automation tools used in the process.	Methods, paragraphs 1-4; Supplementary Materials, Text S1, S2
Effect measures	12	Specify for each outcome the effect measure(s) (e.g. risk ratio, mean difference) used in the synthesis or presentation of results.	Methods, paragraph 4
Synthesis methods	13a	Describe the processes used to decide which studies were eligible for each synthesis (e.g. tabulating the study intervention characteristics and comparing against the planned groups for each synthesis (item #5)).	Methods, paragraphs 1-3
	13b	Describe any methods required to prepare the data for presentation or synthesis, such as handling of missing summary statistics, or data conversions.	Methods, paragraphs 2, 3
	13c	Describe any methods used to tabulate or visually display results of individual studies and syntheses.	Methods, paragraph 4
	13d	Describe any methods used to synthesize results and provide a rationale for the choice(s). If meta-analysis was performed, describe the model(s), method(s) to identify the presence and extent of statistical heterogeneity, and software package(s) used.	Methods, paragraph 4
	13e	Describe any methods used to explore possible causes of heterogeneity among study results (e.g. subgroup analysis, meta-regression).	Methods, paragraph 4
	13f	Describe any sensitivity analyses conducted to assess robustness of the synthesized results.	Methods, paragraph 4
Reporting bias assessment	14	Describe any methods used to assess risk of bias due to missing results in a synthesis (arising from reporting biases).	Methods, paragraph 4
Certainty assessment	15	Describe any methods used to assess certainty (or confidence) in the body of evidence for an outcome.	Methods, paragraph 4
**RESULTS**			

Table S1: PRISMA Checklist

Section and Topic	Item #	Checklist item	Location where item is reported
Study selection	16a	Describe the results of the search and selection process, from the number of records identified in the search to the number of studies included in the review, ideally using a flow diagram.	Results, paragraph 1 and Figure 1
	16b	Cite studies that might appear to meet the inclusion criteria, but which were excluded, and explain why they were excluded.	N/A
Study characteristics	17	Cite each included study and present its characteristics.	Supplementary Materials, Tables S2, S3
Risk of bias in studies	18	Present assessments of risk of bias for each included study.	Results, paragraph 7; Supplementary Materials, Figures S13, Text S3, S4
Results of individual studies	19	For all outcomes, present, for each study: (a) summary statistics for each group (where appropriate) and (b) an effect estimate and its precision (e.g. confidence/credible interval), ideally using structured tables or plots.	Results, paragraphs 2-6, 8, Tables 1-4; Supplementary Materials, Figures 2, S1-S12
Results of syntheses	20a	For each synthesis, briefly summarise the characteristics and risk of bias among contributing studies.	Results, paragraphs 2, 4, 5, 7
	20b	Present results of all statistical syntheses conducted. If meta-analysis was done, present for each the summary estimate and its precision (e.g. confidence/credible interval) and measures of statistical heterogeneity. If comparing groups, describe the direction of the effect.	Results, paragraphs 2-6, 8, Tables 1-4; Supplementary Materials, Figures 2, S1-S12, Table S4
	20c	Present results of all investigations of possible causes of heterogeneity among study results.	Results, paragraphs 2-6, Tables 1-4; Supplementary Materials, Figures S1-S12
	20d	Present results of all sensitivity analyses conducted to assess the robustness of the synthesized results.	Results, paragraphs 2-6, Tables 1-4; Supplementary Materials, Figures S1-S12
Reporting biases	21	Present assessments of risk of bias due to missing results (arising from reporting biases) for each synthesis assessed.	Results, paragraph 7,<br>Supplementary Materials, Figures S11, Text S3, S4
Certainty of evidence	22	Present assessments of certainty (or confidence) in the body of evidence for each outcome assessed.	Results, paragraphs 2-6, 8<br>Tables 1-4; Supplementary Materials, Figures S1-S12, Table S4
**DISCUSSION**			
Discussion	23a	Provide a general interpretation of the results in the context of other evidence.	Discussion, paragraphs 1-7
	23b	Discuss any limitations of the evidence included in the review.	Discussion, paragraph 8
	23c	Discuss any limitations of the review processes used.	Discussion, paragraph

Table S1: PRISMA Checklist

Section and Topic	Item #	Checklist item	Location where item is reported
			8
	23d	Discuss implications of the results for practice, policy, and future research.	Discussion, paragraph 1; Conclusions
**OTHER INFORMATION**			
Registration and protocol	24a	Provide registration information for the review, including register name and registration number, or state that the review was not registered.	Methods, paragraph 1
	24b	Indicate where the review protocol can be accessed, or state that a protocol was not prepared.	Methods, paragraph 1
	24c	Describe and explain any amendments to information provided at registration or in the protocol.	Supplementary Materials, Text S1
Support	25	Describe sources of financial or non-financial support for the review, and the role of the funders or sponsors in the review.	After Conclusions, Funding
Competing interests	26	Declare any competing interests of review authors.	After Conclusions, Conflicts of interests
Availability of data, code and other materials	27	Report which of the following are publicly available and where they can be found: template data collection forms; data extracted from included studies; data used for all analyses; analytic code; any other materials used in the review.	Supplementary Materials, data sharing

From: Page MJ, McKenzie JE, Bossuyt PM, Boutron I, Hoffmann TC, Mulrow CD, et al. The PRISMA 2020 statement: an updated guideline for reporting systematic reviews. BMJ 2021;372:n71. doi: 10.1136/bmj.n71

For more information, visit: http://www.prisma-statement.org/

Table S2: Included studies of helminth coinfections in virus-infected people.

Reference	Country	Virus	Parasite	Income Level	Detection Method	Study Design	Coinfections (n/N)	Quality Score	References
Abaver et al., 2011	Nigeria	HIV	Ascaris, Strongyloides, Trichuris, Taenia	Lower middle	RDT, Western blot	Cross sectional	9/85	2	[53]
Abbas et al., 2009	Egypt	HCV	Schistosoma	Lower middle	PCR, IHA, FECT	Case control	55/99	1	[54]
Abbas et al., 2009	Egypt	HCV	Schistosoma	Lower middle	Latex Agglutination Assay, IHA	Cohort	74/119	0	[55]
Abillo and Afework, 2005	Ethiopia	HIV	Strongyloides, Ascaris	Low	DM, FECT, AFS, ELISA	Case series	18/70	1	[56]
Abossie and Petros, 2015	Ethiopia	HIV	Ascaris, Strongyloides, Taenia, Taenia	Low	RDT, FACS, DM, FECT	Non randomized	55/97	1	[57]
Adams et al., 2006	South Africa	HIV	Ascaris, Taenia	Upper middle	N/A	Cross sectional	103/170	2	[58]
Adamu and Petros, 2009	Ethiopia	HIV	Ascaris, Hymenolepis	Low	ZNS, WMT, FECT	Cross sectional	9/200	0	[59]
Adeleke et al., 2015	South Africa	HIV	Ascaris, Trichuris, Strongyloides, Hymenolepis, Diphyllobothrium, Fasci-olopsis	Upper middle	FECT, Scotch-tape technique, FACS	Cross sectional	57/231	2	[60]
Adesij et al. 2007	Nigeria	HIV	Ascaris, Trichuris	Lower middle	ZNS, ELISA, Western blot	Cross sectional	12/100	2	[61]
Agholi et al., 2013	Iran	HIV	Enterobius, Hymenolepis	Lower middle	DM, PCR	Cross sectional	4/356	3	[62]
Ahmed and Abhay, 2015	India	HIV	Strongyloides, Trichuris, Ancylostoma, Ascaris, Hymenolepis	Lower middle	ZNS, AFS, FACS	Cohort	14/142	0	[63]
Akinbo and Omoregie, 2011	Nigeria	HIV	Ascaris, Strongyloides	Lower middle	ZNS, FECT, FACS	Cross sectional	15/285	1	[64]
Akinbo et al., 2011	Nigeria	HIV	Ascaris, Strongyloides, Trichuris, Taenia	Lower middle	FECT, Ryan's modified Trichrome stain	Retrospective	125/463	1	[65]
Akinbo et al., 2012	Nigeria	HIV	Ascaris, Strongyloides, Trichuris, Taenia	Lower middle	DM, FACS	Cross sectional	156/2000	1	[66]
Allam et al., 2014	Egypt	HCV	Schistosoma	Lower middle	PCR, EIA	Cross sectional	68/141	2	[67]
Amoo et al., 2018	Nigeria	HIV	Ascaris, Strongyloides, Trichuris, Taenia	Lower middle	RDT, Weber's Chromotrope staining and Kinyoun staining, FACS	Cross sectional	99/231	1	[68]
Asma et al., 2011	Malaysia	HIV	Ascaris, Trichuris	Upper middle	FEST, ZNS, AFS	Cross sectional	66/346	2	[69]
Assefa et al., 2009	Ethiopia	HIV	Enterobius, Schistosoma, Strongyloides, Ascaris, Trichuris, Hymenolepis, Taenia,	Low	DM, FACS, AFS	Cross sectional	81/214	2	[70]
Assefa et al., 2013	Ethiopia	HIV	Enterobius, Schistosoma, Strongyloides, Ascaris, Taenia, Hymenolepis, Tri-churis	Low	DM, ZNS, FECT	Cross sectional	11/272	2	[71]
Babatunde et al., 2010	Nigeria	HIV	Strongyloides, Ascaris, Trichuris	Lower middle	ELISA, ZNS, FECT	Cross sectional	39/90	2	[72]
Barcelos et al., 2018	Brazil	HIV	Strongyloides	Upper middle	Rugai's method, Lutz technique, ZNS, Kinyoun staining, Safranin	Cross sectional	1/90	0	[73]

Reference	Country	Virus	Parasite	Income Level	Detection Method	Study Design	Coinfections (n/N)	Quality Score	References
					method, Ritchie method				
Cardoso et al., 2011	Brazil	HIV	Taenia, Strongyloides, Ancylostoma	Upper middle	ZNS, ELISA, Western blot	Cross sectional	4/500	3	[74]
Colombe et al., 2018	Tanzania	HIV	Schistosoma	Lower middle	Kato-Katz, CAA	Prospective	37/79	0	[75]
Costiniuk et al., 2012	Canada	HIV	Strongyloides, Schistosoma,	High	ELISA	Cross sectional	21/97	1	[76]
Da Silva et al., 2005	Brazil	HIV	Hymenolepis, Strongyloides, Trichuris, Taenia	Upper middle	ELISA, MEIA, Baermann method, Lutz Method, Ritchie method	Case control	19/100	1	[77]
Dash et al., 2013	India	HIV	Ascaris, Strongyloides	Lower middle	RDT, WMT, ZNS, FECT	Cross sectional	6/115	2	[78]
Erikstrup et al., 2008	Zimbabwe	HIV	Schistosoma, Schistosoma	Lower middle	RDT, ELISA, FACS, FECT	Randomized	190/198	2	[79]
Eshetu et al., 2017	Nigeria	HIV	Ascaris, Strongyloides, Trichuris, Enterobius, Hymenolepis, Schistosoma	Lower middle	DM, FECT, AFS, FACS	Cross sectional	36/223	2	[80]
Fekadu et al., 2013	Ethiopia	HIV	Strongyloides, Ascaris, Trichuris, Taenia	Low	FACS, FECT, DM	Cross sectional	118/343	2	[81]
Feyisayo et al., 2014	Nigeria	HIV	Ancylostoma	Lower middle	DM, ZNS, FACS	Cross sectional	4/105	0	[82]
Gaurav and Atul, 2015	India	HIV	Strongyloides, Ascaris	Lower middle	RDT, ZNS	Case control	6/40	0	[83]
Getaneh et al., 2018	Ethiopia	HIV	Strongyloides, Ascaris, Taenia, Hymenolepis	Low	ZNS, DM, FECT, FACS	Cross sectional	15/220	2	[84]
Gebretsadik et al., 2018	Ethiopia	HIV	Taenia, Enterobius, Ascaris, Hymenolepis, Strongyloides	Low	ZNS, FECT	Cross sectional	8/223	2	[85]
Guk et al., 2005	Korea	HIV	Clonorchis, Gymnophalloides	High	DM, FECT	Cross sectional	3/105	0	[86]
Gupta et al., 2013	India	HIV	Ascaris, Ascaris, Strongyloides	Lower middle	FECT, AFS, DM, FACS	Case control	3/100	1	[87]
Haileeyesus et al., 2013	Ethiopia	HIV	Trichuris, Ascaris, Strongyloides, Schistosoma, Taenia, Hymenolepis, Entero-bius	Low	DM, ZNS, FECT, FACS	Cross sectional	59/378	2	[88]
Hochberg et al., 2011	United State	HIV	Strongyloides, Schistosoma, Schistosoma	High	ELISA, EIA, IFA, PCR	Cross sectional	48/128	1	[89]
Idindili et al., 2011	Tanzania	HIV	Ascaris, Strongyloides, Schistosoma	Lower middle	ELISA, ZNS, FECT, Kato-Katz	Cross sectional	148/421	2	[90]
Jaiswal et al., 2014	Nepal	HIV	Hymenolepis, Ascaris, Taenia,	Lower middle	DM, WMT	Cross sectional	17/103	1	[91]
Jegede et al., 2014	Nigeria	HIV	Hookworm*	Lower middle	FACS, ZNS	Cross sectional	4/105	1	[92]
Kulkarni et al., 2009	India	HIV	Ascaris, Ancylostoma, Hymenolepis	Lower middle	ELISA, FECT	Cross sectional	9/137	1	[93]
Kallestrup et al., 2005	Zimbabwe	HIV	Schistosoma, Schistosoma	Lower middle	RDT, ELISA	Cross sectional	185/407	3	[94]
Kawai et al., 2009	Tanzania	HIV	Trichuris, Ascaris, Strongyloides, Schistosoma	Lower middle	HPLC, FACS, PCR	Cross sectional	178/971	3	[95]
Kipyegen et al., 2012	Kenya	HIV	Enterobius, Ascaris, Taenia, Trichuris,	Lower middle	WMT, ZNS, FECT	Cross sectional	29/285	2	[96]
Kroidl et al., 2016	Tanzania	HIV	Filaria*	Lower middle	RDT, ELISA, Western blot, PCR	Cohort	69/187	3	[97]

Table S2: Included studies of helminth coinfections in virus-infected people.

Reference	Country	Virus	Parasite	Income Level	Detection Method	Study Design	Coinfections (n/N)	Quality Score	References
Kurniawan et al., 2009	Indonesia	HIV	Ascaris, Strongyloides	Lower middle	AFS, FACS	Cross sectional	3/318	2	[98]
Lar et al., 2016	Nigeria	HIV	Hymenolepis, Ascaris, Schistosoma, Trichuris,	Lower middle	DM, ZNS	Cross sectional	77/205	2	[99]
Lehman et al., 2013	Cameroon	HIV	Strongyloides, Trichuris	Lower middle	DM, FECT, Kinyoun staining, AFS, FACS, Safranin method, FACS	Cross sectional	3/201	2	[100]
Loffredo et al., 2015	Egypt	HCV	Schistosoma	Lower middle	Kato-Katz, PCR, FACS, Biochemical analysis	Retrospective	16/31	1	[101]
Mahmud et al., 2014	Ethiopia	HIV	Strongyloides, Enterobius, Taenia, Hymenolepis, Ascaris,	Low	ZNS, Kato-Katz, FECT	Cross sectional	56/372	2	[102]
Marti et al., 2017	Tanzania	HIV	Schistosoma	Lower middle	RDT, CCA, FACS	Cross sectional	60/305	2	[103]
Mariam et al., 2008	Ethiopia	HIV	Trichuris, Strongyloides, Enterobius, Hymenolepis, Ascaris	Low	RDT, ELISA, ZNS	Cross sectional	58/109	2	[104]
Mascarello et al., 2011	Italy	HIV	Strongyloides	High	IFAT	Cross sectional	15/138	1	[105]
Mathur et al., 2013	India	HIV	Hookworm*	Lower middle	ZNS, WMT	Cross sectional	34/544	1	[106]
Mazigo et al., 2016	Tanzania	HIV	Schistosoma	Lower middle	Kato-Katz, RT-PCR, FACS	Cohort	28/50	3	[107]
Meamar et al., 2007	Iran	HIV	Strongyloides, Hymenolepis, Dicrocoelium, Rhabditis	Lower middle	ZNS, WMT, FECT	Cross sectional	5/781	1	[108]
Mehta et al., 2013	India	HIV	Strongyloides	Lower middle	AFS, FECT, FACS	Cross sectional	3/100	0	[109]
Minti et al., 2017	India	HIV	Ancylostoma	Lower middle	ZNS, FECT, DM, FACS	Cross sectional	1/27	1	[110]
Missaye et al., 2013	Ethiopia	HIV	Taenia, Enterobius, Hymenolepis, Ascaris, Schistosoma, Strongyloides, Trichuris	Low	ZNS, FECT, WMT	Cross sectional	19/272	2	[111]
Mkhize et al., 2017	South Africa	HIV	Ascaris, Trichuris	Upper middle	Kato-Katz, RDT	Cross sectional	31/95	2	[112]
Modjarrad et al., 2005	Zambia	HIV	Hymenolepis, Taenia, Schistosoma, Strongyloides, Ascaris, Hymenolepis, Schistosoma, Strongyloides	Low	Kato-Katz, RDT	Cross sectional	74/297	1	[113]
Modjarrad et al., 2005	Zambia	HIV	Hymenolepis, Taenia, Schistosoma, Strongyloides, Ascaris, Hymenolepis, Schistosoma, Strongyloides	Low	Kato-Katz, RDT, ELISA, FECT	Cross sectional	54/111	2	[114]
Moges et al., 2006	Ethiopia	HIV	Enterobius, Ascaris, Trichuris, Schistosoma, Strongyloides	Low	ELISA, FECT, DM	Cross sectional	29/28	2	[115]
Morawski et al., 2017	Uganda	HIV	Ancylostoma, Trichuris, Strongyloides, Necator	Low	PCR, FACS	Cohort	65/202	2	[116]
Mtapuri et al., 2014	Zimbabwe	HIV	Schistosoma	Lower middle	RDT, ZNS	Cross sectional	1/29	0	[117]
Mulu et al., 2013	Ethiopia	HIV	Schistosoma, Trichuris, Ascaris	Low	Kato-Katz, RT-PCR	Cohort	87/220	3	[31]
Navarro et al., 2006	Colombia	HIV	Trichuris, Strongyloides, Ascaris	Upper middle	PCR, FACS	Cross sectional	11/103	1	[118]

Table S2: Included studies of helminth coinfections in virus-infected people.

Reference	Country	Virus	Parasite	Income Level	Detection Method	Study Design	Coinfections (n/N)	Quality Score	References
Ndhlovu et al., 2007	Zimbabwe	HIV	Schistosoma	Lower middle	RDT, ELISA, Kato-Katz	Cross sectional	72/156	2	[119]
Nielsen et al., 2007	Tanzania	HIV	Wuchereria	Lower middle	ELISA, PCR	Cross sectional	18/34	2	[120]
Nsagha et al., 2016	Cameroon	HIV	Trichuris, Strongyloides, Ascaris, Hymenolepis	Lower middle	RDT, ZNS, Kato-Katz, FECT, AFS, FACS	Cross sectional	34/300	2	[121]
Nuhu et al., 2014	Nigeria	HIV	Taenia	Lower middle	RDT, ZNS, FECT	Case control	8/205	1	[122]
Oguntibeju 2006	Lesotho	HIV	Ascaris, Ancylostoma, Trichuris	Lower middle	DM, ZNS, FECT, AFS	Cross sectional	20/60	0	[123]
Ojurongbe et al., 2011	Nigeria	HIV	Ascaris, Taenia, Strongyloides	Lower middle	RDT, ZNS, FECT	Cross sectional	16/96	1	[124]
Orji et al., 2017	Nigeria	HIV	Ascaris, Strongyloides, Trichuris	Lower middle	Kato-Katz, FACS	Cross sectional	24/84	1	[125]
Oyedeji et al., 2015	Nigeria	HIV	Ascaris, Enterobius	Lower middle	ELISA, Western blot, PCR	Cohort	1/52	2	[126]
Paboriboune et al., 2014	Lao	HIV	Opisthorchis, Strongyloides, Trichuris, Ascaris	Lower middle	RDT, FACS, ZNS, Kato-Katz, ELISA, FECT, AFS	Cross sectional	116/137	1	[127]
Pavie et al., 2012	France	HIV	Strongyloides, Schistosoma, Ancylostoma	High	PCR	Cohort	4/143	2	[128]
Pennap et al., 2015	Nigeria	HIV	Ascaris, Ancylostoma, Taenia, Strongyloides, Trichuris	Lower middle	WMT, FECT	Cross sectional	103/200	1	[129]
Pinlaor et al., 2005	Thailand	HIV	Strongyloides, Taenia, Opisthorchis, Echinosoma	Upper middle	Kato-Katz, RDT	Case control	37/78	1	[130]
Plumelle et al., 2015	France	HIV	Strongyloides	High	ELISA, FACS	Cohort	55/445	1	[131]
Ramakrishnan et al., 2007	India	HIV	Hymenolepis, Ascaris	Lower middle	ZNS, RDT	Case control	7/80	0	[132]
Ramana et al., 2010	India	HIV	Ascaris, Ancylostoma, Hymenolepis, Strongyloides	Lower middle	ELISA, FECT, WMT, AFS	Cross sectional	32/452	2	[133]
Raytekar et al., 2012	India	HIV	Taenia	Lower middle	ELISA, RDT, ZNS	Cross sectional	2/127	1	[134]
Ringo et al., 2015	Tanzania	HIV	Ascaris	Lower middle	WMT, FACS	Cross sectional	13/83	1	[135]
Rodrigues et al., 2008	Brazil	HIV	Strongyloides, Ascaris, Trichuris, Hymenolepis	Upper middle	ZNS, Lutz Method, FACS	Cohort	366/582	2	[136]
Roka et al., 2012	Guinea	HIV	Schistosoma, Ascaris, Strongyloides, Trichuris	Low	RDT, ZNS, DM	Cross sectional	242/260	2	[137]
Roka et al., 2013	Guinea	HIV	Trichuris, Ascaris, Strongyloides, Schistosoma, Schistosoma	Low	RDT, ZNS, DM	Cross sectional	246/273	2	[138]
Saksirisampant et al., 2009	Thailand	HIV	Opisthorchis	Upper middle	ZNS, PCR	Cross sectional	2/90	2	[139]
Samie et al., 2014	South Africa	HIV	Ascaris, Enterobius, Trichuris, Schistosoma, Schistosoma, Hymenolepis, Diphyllobothrium	Upper middle	DM, ZNS, PCR	Cross sectional	62/151	1	[140]
Sanyaolu et al., 2011	Nigeria	HIV	Ascaris, Trichuris, Strongyloides	Lower middle	RDT, DM, FECT, ZNS	Cross sectional	12/65	2	[141]
Sherpa et al., 2010	India	HIV	Ascaris	Lower middle	ZNS, RDT	Cross sectional	4/106	0	[142]

Table S2: Included studies of helminth coinfections in virus-infected people.

Reference	Country	Virus	Parasite	Income Level	Detection Method	Study Design	Coinfections (n/N)	Quality Score	References
Shimelis et al., 2016	Ethiopia	HIV	Trichuris, Ascaris, Strongyloides, Schistosoma, Taenia, Hymenolepis	Low	RDT, ZNS, FECT	Cross sectional	54/491	2	[143]
Silva et al., 2005	Brazil	HIV	Strongyloides, Trichuris, Taenia, Hymenolepis	Upper middle	ELISA, MEIA, Baermann method, Lutz, Ritchie method	Case control	19/100	1	[144]
Smith et al., 2008	Sub-Saharan Africa**	HIV	Schistosoma	Upper middle	ELISA	Cross sectional	21/122	1	[145]
Sonali et al., 2014	India	HIV	Hymenolepis	Lower middle	FACS, ZNS	Cross sectional	2/250	1	[146]
Suryawanshi et al., 2012	India	HIV	Ascaris, Ancylostoma, Hymenolepis, Strongyloides	Lower middle	ELISA, FECT	Cross sectional	12/100	0	[147]
Talaat et al., 2015	India	HIV	Wuchereria	Lower middle	ELISA, Western blot	Case control	28/80	3	[148]
Talaat et al., 2008	India	HIV	Wuchereria	Lower middle	ELISA	Cross sectional	40/632	2	[149]
Tamirat et al., 2017	Ethiopia	HIV	Strongyloides, Ascaris, Trichuris, Hymenolepis	Low	RDT, Baermann method, DM, FECT	Cross sectional	60/226	2	[150]
Tian et al., 2012	China	HIV	Trichuris, Clonorchis,	Upper middle	ELISA, Kato-Katz	Case control	14/302	2	[151]
Tian et al., 2013	China	HIV	Hookworm*	Upper middle	FECT, ELISA, Kato-Katz	Case control	5/79	2	[152]
Tigist et al., 2015	Ethiopia	HIV	Ascaris, Trichuris, Taenia, Schistosoma, Strongyloides, Strongyloides	Low	FECT, RDT	Cross sectional	57/209	2	[153]
Tiwari et al., 2013	Nepal	HIV	Ascaris, Ancylostoma, Hymenolepis, Strongyloides, Trichuris	Lower middle	WMT, AFS, FECT, FACS	Cross sectional	83/745	2	[154]
Tuli et al., 2008	India	HIV	Trichuris, Hymenolepis	Lower middle	ELISA, FACS, AFS	Case control	28/366	2	[155]
Vouking et al., 2014	Cameroon	HIV	Ascaris, Trichuris, Ancylostoma, Necator, Strongyloides	Lower middle	Kato-Katz, FECT, ZNS	Cross sectional	46/207	2	[156]
Vyas et al., 2013	India	HIV	Ascaris	Lower middle	FACS, WMT, FECT	Cross sectional	2/75	0	[157]
Wanyiri et al., 2013	Kenya	HIV	Ascaris, Strongyloides, Schistosoma, Hymenolepis, Enterobius	Lower middle	FACS, ZNS, DM, AFS	Cross sectional	42/164	1	[158]
Wumba et al., 2010	Congo	HIV	Ascaris, Trichuris, Enterobius, Schistosoma	Lower middle	ZNS, PCR	Cross sectional	12/175	2	[159]
Wumba et al., 2012	Congo	HIV	Ascaris, Trichuris	Lower middle	PCR, ZNS	Cross sectional	6/242	2	[160]
Xiao et al., 2015	China	HBV	Ascaris, Trichuris	Upper middle	Kato-Katz, PCR	Cross sectional	22/40	3	[161]
Zeynudin et al., 2013	Ethiopia	HIV	Ascaris, Strongyloides, Hymenolepis, Trichuris	Low	ZNS	Cross sectional	29/91	1	[162]
Gebrecherkos et al., 2019	Ethiopia	HIV	Strongyloides, Ascaris, Schistosoma	Low	DM, ZNF, FECT, FACS	Cross sectional	26/150	1	[162]
Kaneshiro et al., 2019	Lao	HIV	Opisthorchis, Strongyloides, Taenia	Lower middle	DM, RDT,	Cross sectional	87/252	2	[163]
Sannella et al., 2019	Thailand	HIV	Strongyloides, Opisthorchis, Ascaris	Upper middle	DM, PCR	Cross sectional	23/155	2	[164]
Alemayehu etal., 2020	Ethiopia	HIV	Ascaris	Low	FECT, FACS, DM	Cross sectional	74/383	2	[165]
Belay et al., 2020	Ethiopia	HIV	Taenia, Enterobius	Low	FECT, ZNS, DM	Cross sectional	4/112	2	[166]

Table S2: Included studies of helminth coinfections in virus-infected people.

Reference	Country	Virus	Parasite	Income Level	Detection Method	Study Design	Coinfections (n/N)	Quality Score	References
Chhangte et al., 2020	India	HIV	Ascaris, Strongyloides, Hymenolepis	Lower middle	FECT, AFS, DM	Cross sectional	8/250	2	[167]
Lengongo et al., 2020	Gabon	HIV	Trichuris, Ascaris, Strongyloides	Upper middle	ZNS, MIFc, DM	Cross sectional	9/115	2	[168]
McLellan et al., 2020	Canada	HIV	Schistosoma, Strongyloides	High	Kato-Katz, EIA,	Cross sectional	58/253	2	[169]
Namaji et al., 2020	India	HIV	Ascaris	Lower middle	DM	Cross sectional	19/361	2	[170]
Rabiu et al., 2020	Nigeria	HIV	Ascaris	Lower middle	DM, Kato–Katz	Cross sectional	6/197	1	[171]
Botero et al., 2021	Colombia	HIV	Strongyloides, Trichuris	Upper middle	WMT, ZNS	Cross sectional	2/192	2	[172]
Bayleyegn et al., 2021	Ethiopia	HIV	Ascaris, Taenia, Hymenolepis	Low	Giemsa staining, FECT	Cross sectional	32/255	1	[173]
Betancourth et al., 2021	Honduras	HIV	Ascaris, Trichuris	Lower middle	PCR, DM, Kinyoun staining, RDT	Cross sectional	3/102	2	[174]
Feleke et al., 2022	Ethiopia	HIV	Enterobius, Ascaris, Hymenolepis, Strongyloides, Taenia, Schistosoma	Low	ZNS, FECT	Cross sectional	23/223	2	[175]
Miressa et al., 2021	Ethiopia	HIV	Ascaris, Taenia, Strongyloides, Hymenolepis, Trichuris	Low	FACS, DM	Cross sectional	67/230	1	[176]
Chandi et al., 2021	India	HIV	Taenia	Lower middle	ZNS	Cross sectional	6/80	1	[177]
Ajayi et al., 2021	Nigeria	HIV	Ascaris	Lower middle	FEST, ZNS	Cross sectional	10/375	2	[178]
Talukder et al., 2021	Australia	HTLV-1	Strongyloides	High	WB, PCR	Cross sectional	45/201	3	[179]
Barreto et al., 2022	Brazil	HTLV-1	Strongyloides, Ascaris, Trichuris, Enterobius, Schistosoma	Upper middle	ELISA, WB, DM, Baermann–Moraes method	Cross sectional	40/178	2	[180]
Wolday et al., 2021	Ethiopia	SARS-CoV-2	Schistosoma, Hymenolepis, Ascaris, Trichuris, Taenia	Low	RT-PCR	Cohort	184/751	3	[181]
Purbey., 2021	India	HIV	Ascaris, Strongyloides	Lower middle	DM, FECT	Case control	13/75	2	[182]
Uzairue et al., 2021	Nigeria	HIV	Ancylostoma, Ascaris, Strongyloides, Hymenolepis, Trichuris	Lower middle	FACS, ZNS, FECT	Cross sectional	51/170	1	[183]
PrayGod et al., 2022	Tanzania	HIV	Schistosoma, Ascaris, Trichuris, Strongyloides	Lower middle	ELISA, Kato-Katz	Cross sectional	176/1149	3	[184]

* Definitive genus is not available. ** Definitive country is not available.

Table S3: Included studies of protozoan coinfections in virus-infected people.

Reference	Country	Virus	Parasitic Genus	Income Level	Detection Method	Study Design	Coinfections	Quality Score	References
Abaver et al., 2011	Nigeria	HIV	Entamoeba, Giardia, Entamoeba	Lower middle	RDT, Western blot	Cross sectional	12/85	2	[53]
Abdollahi et al., 2015	Iran	HIV	Entamoeba	Lower middle	ELISA, PCR, Western Blot	Case control	28/91	2	[185]
Abdollahi et al., 2013	Iran	HIV	Toxoplasma	Lower middle	ELISA, Western blot	Case control	65/100	2	[186]
Abillo and Afework, 2005	Ethiopia	HIV	Cryptosporidium, Giardia, Entamoeba, Isospora	Low	DM, FECT, AFS, ELISA	Case series	30/70	1	[56]
Adamu and Petros, 2009	Ethiopia	HIV	Cryptosporidium, Giardia, Isospora, Entamoeba, Blastocystis	Low	ZNS, WMT, FECT	Cross sectional	87/200	0	[59]
Adamu et al., 2014	Ethiopia	HIV	Cryptosporidium	Low	PCR	Cross sectional	139/520	3	[187]
Addebbous et al., 2012	Morocco	HIV	Toxoplasma	Lower middle	ELISA, FACS, Western blot	Cross sectional	59/95	2	[188]
Adesij et al. 2007	Nigeria	HIV	Cryptosporidium, Entamoeba	Lower middle	ZNS, ELISA, Western blot	Cross sectional	85/100	2	[61]
Adu-Gyasi et al., 2013	Ghana	HIV	Plasmodium	Lower middle	RDT, Giemsa staining	Cross sectional	15/933	2	[189]
Aghaee et al., 2017	Iran	HIV	Toxoplasma	Lower middle	ELISA, PCR, FACS	Cross sectional	11/49	2	[190]
Agholi et al., 2013	Iran	HIV	Giardia, Cyclospora, Isospora, Entamoeba, Cryptosporidium, Blastocystis	Lower middle	DM, PCR	Cross sectional	81/356	3	[62]
Ahmed and Abhay, 2015	India	HIV	Entamoeba, Cryptosporidium, Isospora, Cyclospora, Giardia	Lower middle	ZNS, AFS, FACS	Cohort	43/142	0	[63]
Ajjampur et al., 2007	India	HIV	Cryptosporidium	Lower middle	AFS, PCR, FACS	Cross sectional	48/534	2	[191]
Akinbo et al., 2016	Nigeria	HIV	Plasmodium	Lower middle	FACS, RDT, Giemsa Staining	Case control	32/411	1	[192]
Akinbo et al., 2010	Nigeria	HIV	Cryptosporidium	Lower middle	FACS, PCR	Cross sectional	36/400	2	[193]
Akinbo et al., 2011	Nigeria	HIV	Entamoeba, Isospora	Lower middle	FECT, Ryan's modified trichrome staining	Retrospective	21/463	1	[64]
Akinbo et al., 2012	Nigeria	HIV	Plasmodium, Giardia, Entamoeba, Isospora, Cryptosporidium	Lower middle	DM, FACS	Cross sectional	190/2000	1	[194]
Ako-Nai et al., 2013	Nigeria	HIV	Plasmodium	Lower middle	RDT, ELISA, FACS, Giemsa staining	Retrospective	34/44	1	[195]
Alavi et al., 2013	Iran	HIV	Toxoplasma	Lower middle	ELISA, Western blot	Case control	31/42	2	[196]
Alberto et al., 2011	Colombia	HIV	Cryptosporidium	Upper middle	ELISA, Western blot	Cross sectional	38/131	1	[197]
Almeida et al., 2012	Brazil	HIV	Leishmania	Upper middle	RDT, PCR, DAT, IFA, Western blot	Case series	5/10	1	[198]
Almeida et al., 2010	Brazil	HIV	Trypanosoma	Upper middle	IFA, ELISA	Cross sectional	9/716	2	[199]

Table S3: Included studies of protozoan coinfections in virus-infected people.

Amoo et al., 2018	Nigeria	HIV	Cryptosporidium	Lower middle	RDT, Weber's Chromotrope staining, Kinyoun staining, FACS	Cross sectional	52/231	1	[68]
Amuta et al., 2012	Nigeria	HIV	Toxoplasma, Plasmodium	Lower middle	ELISA, FACS	Cross sectional	39/360	2	[200]
Amuta et al., 2012	Nigeria	HIV	Toxoplasma, Plasmodium	Lower middle	DM, FACS	Cross sectional	343/1080	2	[201]
Angal et al., 2016	Malaysia	HIV	Toxoplasma	Upper middle	ELISA	Case control	84/133	1	[202]
Anteneh et al., 2014	Ethiopia	HIV	Toxoplasma	Low	ELISA	Cross sectional	178/190	2	[203]
Archana et al., 2017	India	HIV	Isospora	Lower middle	FECT, ZNS	Cross sectional	17/104	0	[204]
Arefkhah et al., 2018	Iran	HIV	Toxoplasma	Lower middle	ELISA, PCR	Cross sectional	42/251	3	[205]
Arundhati and Niharika, 2016	India	Dengue virus	Plasmodium	Lower middle	ELISA	Cross sectional	16/101	2	[206]
Asma et al., 2011	Malaysia	HIV	Giardia, Isospora, Entamoeba, Cryptosporidium, Cyclospora	Upper middle	ZNS, AFS	Cross sectional	105/346	2	[69]
Assefa et al., 2009	Ethiopia	HIV	Cryptosporidium, Entamoeba, Giardia, Isospora, Entamoeba, Trichomonas	Low	DM, AFS, FACS	Cross sectional	152/214	2	[70]
Assefa et al., 2013	Ethiopia	HIV	Cryptosporidium, Entamoeba, Isospora, Giardia	Low	ZNS, DM, FECT	Cross sectional	43/272	2	[71]
Assir et al., 2014	Pakistan	Dengue virus	Plasmodium	Lower middle	PCR, ELISA, DM	Cross sectional	22/85	2	[207]
Assob et al., 2011	Cameroon	HIV	Toxoplasma	Lower middle	ELISA	Cross sectional	93/133	1	[208]
Ayi et al., 2016	Ghana	HIV	Toxoplasma	Lower middle	ELISA	Cross sectional	72/125	1	[209]
Babatunde et al., 2010	Nigeria	HIV	Isospora, Giardia, Entamoeba, Cryptosporidium, Cyclospora, Iodamoeba	Lower middle	ELISA, ZNS, FECT	Cross sectional	109/90	2	[72]
Bamba et al., 2014	Burkina Faso	HIV	Toxoplasma	Low	RDT, HSDA, ELFA	Cross sectional	11/16	2	[210]
Barcelos et al., 2018	Brazil	HIV	Entamoeba, Endolimax, Cryptosporidium, Entamoeba, Giardia, Iodamoeba, Blastocystis, Cystoisospora, Entamoeba	Upper middle	Rugai's method, Lutz technique, ZNS, Kinyoun staining, Safarian method, Ritchie method	Cross sectional	25/90	0	[73]
Bate et al., 2016	Cameroon	HIV	Plasmodium	Lower middle	DM, FACS, HB Estimation	Cross sectional	58/234	2	[211]
Beena et al., 2015	India	HIV	Toxoplasma	Lower middle	ELSIA	Cross sectional	141/661	2	[212]
Berenji et al., 2010	Iran	HIV	Chilomastix, Giardia, Entamoeba, Blastocystis, Enteromonas	Lower middle	DM, FECT, ASF	Cross sectional	21/31	1	[213]
Bessong and Mathomu 2010	South Africa	HIV	Toxoplasma	Upper middle	ELSIA	Cross sectional	29/160	1	[214]
Bharti et al., 2012	India	HIV	Plasmodium	Lower middle	PCR, RDT, FACS	Cross sectional	45/460	3	[215]

Table S3: Included studies of protozoan coinfections in virus-infected people.

Cardoso et al., 2011	Brazil	HIV	Isospora, Giardia, Cryptosporidium, Entamoeba, Endolimax	Upper middle	ZNS, ELISA, Western blot	Cross sectional	19/500	3	[74]
Carranza et al., 2009	Brazil	HIV	Leishmania	Upper middle	RDT, PCR, ELISA, IFAT	Cross sectional	26/163	2	[216]
Cavalcanti et al., 2012	Brazil	HIV	Leishmania	Upper middle	RDT, IIFT, Western blot, PCR	Case series	5/10	1	[217]
Chalwe et al., 2009	Zambia	HIV	Plasmodium	Low	DM, RDT, ICT	Case control	42/55	1	[41]
Chemoh et al., 2015	Thailand	HIV	Toxoplasma	Upper middle	ELISA, Western blot	Cross sectional	109/300	3	[218]
Chukwuanukwu et al., 2016	Nigeria	HIV	Plasmodium	Lower middle	CI, DM	Case control	110/239	1	[219]
Cohen et al., 2005	South Africa	HIV	Plasmodium	Upper middle	FACS,Giemsa staining, ELISA	Cohort	18/110	3	[220]
Colomba et al., 2009	Itlay	HIV	Leishmania	High	IFAT, ELISA, PCR	Cross sectional	24/145	1	[221]
Cota et al., 2013	Brazil	HIV	Leishmania	Upper middle	PCR, RDT, IFAT	Cross sectional	41/113	2	[222]
da Silva et al., 2005	Brazil	HIV	Cryptosporidium, Isospora, Giardia	Upper middle	ELISA, MEIA, Baermann method, Lutz Method, Ritchie method, Safranin/Methylene blue method, Weber's Chromotrope	Cross sectional	12/100	0	[77]
Dabo et al., 2015	Nigeria	HBV	Plasmodium	Lower middle	DM, ELISA, RDT	Cross sectional	9/13	1	[223]
Daryani et al., 2009	Iran	HIV	Cryptosporidium, Entamoeba, Giardia, Chilomastix, Entamoeba	Lower middle	DM, ZNS, FECT, ELISA, Western blot	Cross sectional	11/64	2	[224]
Daryani et al., 2011	Iran	HIV	Toxoplasma	Lower middle	FACS, ELISA	Cross sectional	54/62	1	[225]
Dash et al., 2013	India	HIV	Giardia, Isospora, Cyclospora, Cryptosporidium, Entamoeba	Lower middle	RDT, WMT, ZNS, FECT	Cross sectional	44/115	2	[78]
Davarpanah et al., 2007	Iran	HIV	Toxoplasma	Lower middle	IIFT, MRI	Cross sectional	38/208	2	[226]
Davis et al., 2016	United state	HIV	Trichomonas	High	RDT, PCR	Case control	16/44	2	[227]
De Lemos and Garcia-Zapata, 2010	Brazil	HIV	Trichomonas	Upper middle	DM	Case control	23/125	1	[228]
Dechassa et al., 2016	Ethiopia	HIV	Toxoplasma	Low	Toxo-latex test	Cross sectional	109/135	0	[229]
Desta et al., 2005	Ethiopia	HIV	Plasmodium	Low	RDT, DM, ELISA, FACS	Cross sectional	8/49	3	[230]
Djieyep et al., 2014	Nigeria	HIV	Cryptosporidium, Isospora	Lower middle	AFS, ZNS, PCV, FACS	Cross sectional	222/305	2	[231]
Domingos et al., 2013	Mozambique	HIV	Toxoplasma	Low	RDT, FACS	Cross sectional	92/200	1	[232]
Ebrahim et al., 2018	Iran	HIV	Toxoplasma	Lower middle	ELISA, Western blot	Cross sectional	51/246	3	[233]
Echoru et al., 2015	Uganda	HIV	Cryptosporidium, Isospora	Low	ZNS, DM	Cross sectional	6/111	1	[234]
El Nahas et al., 2014	Egypt	HCV	Toxoplasma	Lower middle	ELISA, PCR	Case control	105/120	2	[235]

Table S3: Included studies of protozoan coinfections in virus-infected people.

Epelboin et al., 2012	France	Dengue virus	Plasmodium	High	PCR	Cohort	104/208	3	[236]
Erhabor et al., 2011	Nigeria	HIV	Cryptosporidium	Lower middle	ZNS	Cross sectional	3/105	0	[237]
Eshetu et al., 2017	Nigeria	HIV	Cyclospora, Cryptosporidium, Entamoeba, Giardia	Lower middle	DM, FECT, AFS, FACS	Cross sectional	36/223	2	[80]
Feyisayo et al., 2014	Nigeria	HIV	Entamoeba, Blastocystis	Lower middle	DM, ZNS, FACS	Cross sectional	8/105	0	[82]
Fisseha et al., 2013	Ethiopia	HIV	Toxoplasma	Low	ELISA	Cross sectional	90/103	2	[238]
Franke et al., 2010	Tanzania	HIV	Plasmodium	Lower middle	DM	Cross sectional	199/1066	2	[239]
Frosch et al., 2017	Kenya	HIV	Plasmodium	Lower middle	Kato Katz, FACS	Cross sectional	11/138	1	[240]
Gabriela et al., 2005	Venezuela	HIV	Cryptosporidium	Upper middle	Kato-Katz	Cross sectional	59/397	2	[241]
Gallagher et al., 2005	Kenya	HIV	Plasmodium	Lower middle	ELISA	Cohort	34/83	2	[242]
Ganiem et al., 2013	Indonesia	HIV	Toxoplasma	Lower middle	PCR, FACS, ECLIA	Cohort	21/64	3	[243]
Garcia et al., 2006	Spain	HIV	Leishmania	High	PCR, ELISA, Western blot	Cross sectional	28/92	2	[244]
Gaurav and Atul, 2015	India	HIV	Isospora, Entamoeba, Giardia, Cryptosporidium	Lower middle	RDT, ZNS	Case control	14/40	0	[83]
Gebretsadik et al., 2018	Ethiopia	HIV	Isospora, Entamoeba, Giardia, Cryptosporidium	Low	ZNS, FECT	Cross sectional	23/223	2	[85]
Getaneh et al., 2018	Ethiopia	HIV	Cyclospora, Entamoeba, Cryptosporidium, Giardia, Isospora	Low	ZNS, DM, FECT, FACS	Cross sectional	47/220	2	[84]
Ghafari et al., 2018	Iran	HIV	Cryptosporidium	Lower middle	ZNS, FECT, PCR	Cross sectional	27/250	2	[245]
Gholami et al., 2016	Iran	HIV	Cryptosporidium	Lower middle	AFS, FECT, FACS	Cross sectional	4/53	1	[246]
Girma et al., 2014	Ethiopia	HIV	Cryptosporidium, Isospora	Low	ZNS, FECT	Cross sectional	94/268	2	[247]
Goselle et al., 2007	Nigeria	HIV	Plasmodium	Lower middle	DM	Cross sectional	64/200	0	[248]
Guk et al., 2005	Korea	HIV	Isospora, Giardia, Cryptosporidium	High	DM, FECT	Cross sectional	13/105	0	[86]
Gupta et al., 2013	India	HIV	Giardia, Isospora, Cryptosporidium, Entamoeba, Endolimax, Entamoeba, Trichomonas, Iodamoeba, Chilomastix, Blastocystis	Lower middle	FECT, AFS, DM, FACS	Case control	40/100	1	[87]
Haileeyesus et al., 2013	Ethiopia	HIV	Cryptosporidium, Blastocystis, Isospora, Giardia, Entamoeba	Low	DM, ZNS, FECT, FACS	Cross sectional	93/378	2	[88]
Hari et al., 2007	South Africa	HIV	Toxoplasma	Upper middle	Radiology, ELISA	Cross sectional	25/307	1	[249]
Hasang et al., 2014	Australia	HIV	Plasmodium	High	RDT, DM, FACS	Case control	100/216	1	[250]
Huibers et al., 2018	Malawi	HIV	Giardia, Cryptosporidium, Dientamoeba	Low	RT-PCR, RDT	Cross sectional	19/35	1	[251]
Hung et al., 2005	China	HIV	Toxoplasma	Upper middle	RDT, MRI, CT scan	Prospective	56/550	1	[252]
Hung et al., 2008	China	HIV	Entamoeba	Upper middle	PCR, IHA, AFS	Cross sectional	66/991	3	[253]
Hung et al., 2007	China	HIV	Cryptosporidium	Upper middle	PCR, ZNS,	Cross sectional	5/1376	3	[254]
Hussain et al., 2016	India	HIV	Cryptosporidium	Lower middle	RDT, DM, ZNS	Cross sectional	25/127	0	[255]
Idindili et al., 2011	Tanzania	HIV	Plasmodium	Lower middle	ELISA, ZNS, FECT, Kato-Katz	Cross sectional	105/421	2	[90]

Table S3: Included studies of protozoan coinfections in virus-infected people.

Igbeneghu et al., 2012	Nigeria	HIV	Plasmodium	Lower middle	RDT, ELISA, Western blot	Cross sectional	18/38	1	[256]
Iqbal et al., 2015	Malaysia	HIV	Cryptosporidium	Upper middle	DM, ZNS, AFS, PCR	Cross sectional	43/346	2	[257]
Iroezindu et al., 2012	Nigeria	HIV	Plasmodium	Lower middle	DM, FACS	Cross sectional	24/97	1	[258]
Iroezindu et al., 2012	Nigeria	HIV	Plasmodium	Lower middle	RDT, Western blot	Cross sectional	21/63	2	[259]
Izuka et al., 2017	Nigeria	HIV	Plasmodium	Lower middle	DM	Cohort	70/102	2	[260]
Jaiswal et al., 2014	Nepal	HIV	Entamoeba, Cryptosporidium, Isospora, Cyclospora, Giardia	Lower middle	DM, WMT	Cross sectional	26/103	1	[91]
Jayalakshmi et al., 2008	India	HIV	Isospora, Cryptosporidium, Giardia, Entamoeba	Lower middle	ELISA, AFS, FECT	Cross sectional	22/89	1	[261]
Javid et al., 2005	India	HIV	Cryptosporidium, Giardia, Entamoeba	Lower middle	ELISA, FACT, FECT	Retrospective	164/200	2	[262]
Jegede et al., 2014	Nigeria	HIV	Entamoeba, Blastocystis	Lower middle	FACS, ZNS	Cross sectional	8/105	1	[92]
Johnbull et al., 2014	Nigeria	HIV	Plasmodium	Lower middle	DM, RDT, Western blot	Cross sectional	149/301	3	[263]
Júnior et al., 2015	Brazil	HIV	Leishmania	Upper middle	RDT, ICT, DAT, PCR	Cross sectional	7/15	1	[264]
Kammili et al., 2008	India	HIV	Cryptosporidium, Cyclospora, Isospora	Lower middle	FECT, ZNS, WMT, PCR	Cross sectional	42/175	2	[265]
Kapito et al., 2011	Malawi	HIV	Plasmodium	Low	DM, PCR	Cross sectional	113/1142	3	[266]
Kawai et al., 2009	Tanzania	HIV	Plasmodium	Lower middle	HPLC, FACS, PCR	Cross sectional	177/971	3	[95]
Kfutwah et al., 2009	Cameroon	HIV	Plasmodium	Lower middle	ELISA, PCR	Retrospective	14/80	1	[267]
Kinimi et al., 2018	Tanzania	Chikungunya virus	Plasmodium	Lower middle	RDT, ELISA	Cross sectional	8/97	2	[268]
Kipyegen et al., 2012	Kenya	HIV	Entamoeba, Giardia, Entamoeba	Lower middle	WMT, ZNS, FECT	Cross sectional	122/285	2	[96]
Kiros et al., 2015	Ethiopia	HIV	Entamoeba, Cryptosporidium, Isospora, Giardia	Low	DM, FECT, ZNS, FACS	Cross sectional	122/399	1	[269]
Kissinger et al., 2008	United state	HIV	Trichomonas	High	WMT	Randomized	11/60	1	[270]
Kiying et al., 2010	Uganda	HIV	Plasmodium	Low	RDT, DM	Prospective	120/160	1	[271]
Kodym et al., 2015	Czech Republic	HIV	Toxoplasma	High	CFT, ELISA, PCR	Cohort	502/1235	2	[272]
Kolawole et al., 2017	Nigeria	Dengue virus	Plasmodium	Lower middle	ELISA, PCR, RDT	Cross sectional	5/81	2	[273]
Kucerova et al., 2011	Russia	HIV	Cryptosporidium	Upper middle	ELISA	Cross sectional	19/46	0	[274]
Kulkarni et al., 2009	India	HIV	Cyclospora, Entamoeba, Isospora, Cryptosporidium	Lower middle	ELISA, FECT	Cross sectional	37/137	1	[93]
Kurniawan et al., 2013	Indonesia	HIV	Cryptosporidium	Lower middle	AFS, PCR	Cross sectional	30/36	1	[275]
Kurniawan et al., 2009	Indonesia	HIV	Giardia, Cryptosporidium, Cyclospora, Entamoeba, Blastocystis, Entamoeba, Isospora	Lower middle	AFS, FACS	Cross sectional	266/318	2	[98]
Lago et al., 2009	Brazil	HIV	Toxoplasma	Upper middle	ELFA, ELISA, Western blot	Cross sectional	121/168	3	[276]

Table S3: Included studies of protozoan coinfections in virus-infected people.

Langoni and Souza, 2007	France	HIV	Cryptosporidium	High	FACS, ZNS, FECT	Cross sectional	11/105	0	[277]
Lar et al., 2016	Nigeria	HIV	Entamoeba, Cryptosporidium, Entamoeba	Lower middle	DM, ZNS	Cross sectional	50/205	2	[99]
Lehman et al., 2013	Cameroon	HIV	Cryptosporidium, Entamoeba, Isospora, Entamoeba, Cyclospora	Lower middle	DM, FECT, Kinyoun staining, AFS, FACS, Safranin method, FACS	Cross sectional	21/201	2	[100]
Lemos et al., 2009	Brazil	HIV	Trichomonas	Upper middle	WMT, DM	Case control	23/125	1	[278]
Li et al., 2010	China	HIV	Toxoplasma	Upper middle	ELISA, ICT	Case control	58/183	1	[279]
Llenas et al., 2012	Spain	HIV	Trypanosoma	High	ICT, ELISA, Western blot, IFAT, PCR	Cross sectional	4/155	2	[280]
Machala et al., 2009	Czech Republic	HIV	Toxoplasma	High	CFT, ELISA, CT scan, MRI	Cross sectional	268/626	2	[281]
Magalhaes et al., 2014	Brazil	Dengue virus	Plasmodium	Upper middle	PCR, ELISA, DM	Cross sectional	44/628	3	[282]
Mahmud et al., 2014	Ethiopia	HIV	Entamoeba, Giardia	Low	ZNS, Kato-Katz, FECT	Cross sectional	194/372	2	[102]
Maikai et al., 2012	Nigeria	HIV	Cryptosporidium	Lower middle	PCR	Cross sectional	1/61	1	[283]
Malla et al., 2005	India	HIV	Toxoplasma	Lower middle	ELISA, FACS	Cross sectional	14/100	1	[284]
Manyanga et al., 2014	Tanzania	HIV	Plasmodium	Lower middle	RDT, FACS, HB estimation	Cross sectional	19/420	2	[285]
Marete et al., 2014	Kenya	HIV	Plasmodium	Lower middle	DM, FACS	Cross sectional	201/245	2	[286]
Mariam et al., 2008	Ethiopia	HIV	Cryptosporidium, Isospora, Entamoeba, Giardia, Blastocystis	Low	RDT, ELISA, ZNS	Cross sectional	44/109	2	[104]
Masarat et al., 2012	India	HIV	Cryptosporidium	Lower middle	FECT, DM, ELISA, AFS	Cross sectional	45/45	1	[287]
Masese et al., 2011	Kenya	HIV	Trichomonas	Lower middle	WMT, RDT, ELISA	Cohort	31/147	2	[288]
Mathur et al., 2013	India	HIV	Cryptosporidium, Isospora, Cyclospora, Entamoeba	Lower middle	ZNS, WMT	Cross sectional	238/544	1	[106]
Meamar et al., 2007	Iran	HIV	Blastocystis, Giardia, Cryptosporidium, Isospora	Lower middle	ZNS, WMT, FECT	Cross sectional	90/781	1	[108]
Mehta et al., 2013	India	HIV	Isospora, Giardia, Cryptosporidium	Lower middle	AFS, FECT, FACS	Cross sectional	25/100	0	[109]
Mekonnen et al., 2014	Ethiopia	HIV	Cryptosporidium, Isospora	Low	FECT, ZNS	Cross sectional	96/268	2	[289]
Mengistu et al., 2014	Ethiopia	HIV	Toxoplasma	Low	RDT, RLAT	Cross sectional	38/43	2	[290]
Minti et al., 2017	India	HIV	Entamoeba	Lower middle	ZNS, FECT, DM, FACS	Cross sectional	1/27	1	[110]
Missaye et al., 2013	Ethiopia	HIV	Entamoeba, Giardia, Isospora, Cryptosporidium	Low	ZNS, FECT, WMT	Cross sectional	66/272	2	[111]
Mitra et al., 2016	India	HIV	Cryptosporidium, Cyclospora, Entamoeba, Giardia, Isospora	Lower middle	ZNS, RDT, ELISA	Case control	120/194	3	[291]
Moges et al., 2006	Ethiopia	HIV	Giardia, Entamoeba	Low	ELISA, FECT, DM	Cross sectional	3/28	3	[115]

Table S3: Included studies of protozoan coinfections in virus-infected people.

Mohan et al., 2014	India	HIV	Cryptosporidium, Cyclospora, Blastocystis, Isospora	Lower middle	WMT, FECT, AFS, Modified Safranin method, FACS	Cross sectional	14/150	0	[292]
Mohapatra et al., 2017	India	HIV	Plasmodium	Lower middle	RDT, DM, PCR, FACS	Cohort	23/333	3	[293]
Mohraz et al., 2011	Iran	HIV	Toxoplasma	Lower middle	ELISA, FACS	Cross sectional	100/201	2	[294]
Moran et al., 2009	Mexico	HIV	Entamoeba	Upper middle	DM, RDT, PCR, FACS	Case control	31/100	2	[295]
Moran et al., 2005	Mexico	HIV	Isospora, Blastocystis, Entamoeba, Giardia, Entamoeba, Endolimax, Iodamoeba	Upper middle	PCR, DM	Case control	115/203	2	[296]
Morawski et al., 2017	Uganda	HIV	Entamoeba, Cryptosporidium, Giardia	Low	PCR, FACS	Cohort	16/202	2	[116]
Morona et al., 2018	Tanzania	HIV	Plasmodium	Lower middle	RDT	Cross sectional	56/250	2	[297]
Mtapuri et al., 2014	Zimbabwe	HIV	Giardia, Entamoeba, Cryptosporidium, Blastocystis, Iodamoeba, Entamoeba, Cyclospora, Endolimax, Trichomonas	Lower middle	RDT, ZNS	Cross sectional	21/29	0	[117]
Nangammbi et al., 2013	South Africa	HIV	Trichomonas	Upper middle	PCR	Cross sectional	32/155	1	[298]
Navarro et al., 2006	Colombia	HIV	Giardia, Entamoeba, Isospora	Upper middle	PCR, FACS	Cross sectional	16/103	1	[118]
Nazari et al., 2018	Iran	HIV	Toxoplasma	Lower middle	ELISA, FACS	Cross sectional	167/385	2	[299]
Ngobeni and Samie, 2017	South Africa	HIV	Toxoplasma	Upper middle	ELISA	Cross sectional	38/100	0	[300]
Ngum et al., 2015	Cameroon	HIV	Cryptosporidium, Entamoeba, Iodamoeba, Entamoeba, Cystoisospora	Lower middle	WMT, FECT, ZNS, RDT	Cross sectional	138/320	2	[301]
Nissapatorn et al., 2007	Malaysia	HIV	Toxoplasma	Upper middle	CT scan, ELISA	Cross sectional	329/693	2	[302]
Njunda et al., 2016	Cameroon	HIV	Plasmodium	Lower middle	FACS, DM	Cross sectional	26/355	2	[303]
Nkenfou et al., 2013	Cameroon	HIV	Cryptosporidium, Entamoeba, Giardia	Lower middle	RDT, PCR, ZNS, FECT	Cross sectional	25/42	3	[304]
Nkenfou et al., 2017	Cameroon	HIV	Cryptosporidium, Entamoeba, Blastocystis, Entamoeba, Iodamoeba, Giardia, Cyclospora	Lower middle	RDT, ZNS, FECT	Cross sectional	62/68	2	[305]
Nkhoma et al., 2012	Malawi	HIV	Plasmodium	Low	RDT, DM, ZNS	Cohort	70/185	2	[306]
Nsagha et al., 2016	Cameroon	HIV	Isospora, Cryptosporidium, Blastocystis, Entamoeba, Giardia, Cyclospora	Lower middle	RDT, ZNS, Kato-Katz, FECT, AFS, FACS	Cross sectional	223/300	2	[121]
Nuhu et al., 2014	Nigeria	HIV	Cryptosporidium, Giardia, Entamoeba	Lower middle	RDT, ZNS, FECT	Case control	34/205	1	[122]
Nwadioha et al., 2012	Nigeria	HIV	Trichomonas	Lower middle	DM	Cross sectional	84/350	1	[307]
Nweze and Mouneke, 2011	Nigeria	HIV	Trichomonas	Lower middle	DM, FACS	Cross sectional	355/970	1	[308]
Ogoina et al., 2013	Nigeria	HIV	Toxoplasma	Lower middle	ELISA, FACS	Cross sectional	43/111	2	[309]

Table S3: Included studies of protozoan coinfections in virus-infected people.

Oguntibeju 2006	Lesotho	HIV	Giardia, Entamoeba, Isospora, Cryptosporidium, Entamoeba, Blastocystis	Lower middle	DM, ZNS, FECT, AFS	Cross sectional	25/60	0	[123]
Ojuromi et al., 2016	Nigeria	HIV	Cryptosporidium	Lower middle	PCR, ZNS	Cross sectional	4/90	1	[310]
Ojurongbe et al., 2011	Nigeria	HIV	Entamoeba	Lower middle	RDT, ZNS, FECT	Cross sectional	56/96	1	[124]
Okogun et al., 2010	Nigeria	HIV	Plasmodium	Lower middle	DM, RDT, EIA, Romanowsky stains	Cross sectional	5/16	0	[311]
Okonkwo et al., 2018	Nigeria	HIV	Plasmodium	Lower middle	PCR, RDT, DM, FACS	Cross sectional	61/179	3	[312]
Olaniran et al., 2012	Nigeria	HIV	Plasmodium	Lower middle	DM, FACS,	Cross sectional	83/93	0	[313]
Oliveira et al., 2011	Brazil	HIV	Leishmania	Upper middle	PCR, IFA	Cross sectional	13/169	1	[314]
Olopade et al., 2017	Nigeria	HIV	Cryptosporidium	Lower middle	ZNS, FECT	Cross sectional	10/226	2	[315]
Omalu et al., 2012	Nigeria	HBV	Plasmodium	Lower middle	ELISA	Cross sectional	27/30	1	
Omoti et al., 2013	Nigeria	HIV	Plasmodium	Lower middle	RDT, FACS, DM	Cross sectional	254/342	1	[316]
Onyenekwe et al., 2007	Nigeria	HIV	Plasmodium	Lower middle	ICT, RIT	Prospective	28/149	0	[317]
Oshinaike et al., 2010	Nigeria	HIV	Toxoplasma	Lower middle	ELISA, CT Scan	Case control	71/83	1	[318]
Osuji et al., 2012	Nigeria	HIV	Plasmodium	Lower middle	ICT, DM	Cross sectional	74/134	0	[319]
Osunkalu et al., 2011	Nigeria	HIV	Toxoplasma	Lower middle	FACS, ELISA	Cross sectional	206/380	1	[320]
Ouermi et al., 2009	Burkina Faso	HIV	Toxoplasma	Low	ELISA, FACS,	Cross sectional	44/138	1	[321]
Oyedeji et al., 2015	Nigeria	HIV	Cryptosporidium	Lower middle	ELISA, Western blot, PCR	Cohort	10/52	2	[126]
Paboriboune et al., 2014	Lao People's Democratic Republic	HIV	Entamoeba, Cystoisospora, Cyclospora, Giardia, Cryptosporidium, Entamoeba, Blastocystis	Lower middle	RDT, FACS, ZNS, Kato-Katz, ELISA, FECT, AFS	Cross sectional	89/137	2	[127]
Pandey et al., 2018	Thailand	HIV	Leishmania	Upper middle	DAT, PCR	Cross sectional	176/305	2	[322]
Pappoe et al., 2017	Ghana	HIV	Toxoplasma	Lower middle	ELISA, PCR, FECT	Cross sectional	293/394	3	[323]
Patel et al., 2011	India	HIV	Entamoeba, Isospora, Giardia, Cryptosporidium, Iodamoeba	Lower middle	ELISA, ZNS, FECT	Cross sectional	28/100	1	[324]
Pavie et al., 2012	France	HIV	Cryptosporidium, Giardia, Isospora, Entamoeba	High	PCR	Cohort	21/143	2	[128]
Pennap et al., 2015	Nigeria	HIV	Cyclospora, Entamoeba, Giardia, Cryptosporidium, Isospora, Blastocystis	Lower middle	WMT, FECT	Cross sectional	74/200	1	[129]
Pinlaor et al., 2005	Thailand	HIV	Cryptosporidium, Cyclospora, Isospora, Blastocystis, Giardia	Upper middle	Kato-Katz, RDT	Case control	13/78	1	[130]
Price et al., 2018	South Africa	HIV	Trichomonas	Upper middle	PCR	Cross sectional	76/359	2	[325]

Table S3: Included studies of protozoan coinfections in virus-infected people.

Quigley et al., 2005	Uganda	HIV	Plasmodium	Low	DM, Western blot	Cohort	154/222	3	[326]
Rahimi et al., 2015	Iran	HIV	Toxoplasma	Lower middle	ELISA	Cross sectional	79/82	1	[327]
Ramakrishnan et al., 2007	India	HIV	Entamoeba, Cryptosporidium, Entamoeba, Giardia, Isospora	Lower middle	ZNS, RDT	Case control	57/80	0	[132]
Ramana et al., 2010	India	HIV	Cryptosporidium, Cyclospora, Isospora, Entamoeba, Blastocystis, Entamoeba, Giardia	Lower middle	ELISA, FECT, WMT, AFS	Cross sectional	364/452	2	[133]
Rao et al., 2016	India	Dengue virus	Plasmodium	Lower middle	RDT, ELISA, PCR	Cross sectional	22/745	3	[49]
Rashmi and Ravi, 2013	India	HIV	Cryptosporidium	Lower middle	ZNS, ELISA	Cohort	15/90	2	[328]
Raytekar et al., 2012	India	HIV	Cryptosporidium, Entamoeba, Giardia	Lower middle	ELISA, RDT, ZNS	Cross sectional	25/127	1	[134]
Reema and Dharmendra, 2017	India	HIV	Toxoplasma	Lower middle	ELISA	Cross sectional	52/100	1	[329]
Rezaei et al., 2018	Iran	HIV	Leishmania	Lower middle	PCR, ELISA	Cross sectional	19/251	2	[330]
Rezanezhad et al., 2017	Iran	HIV	Toxoplasma	Lower middle	PCR, ELISA	Cross sectional	19/90	1	[331]
Ringo et al., 2015	Tanzania	HIV	Entamoeba, Giardia	Lower middle	WMT, FACT	Cross sectional	12/83	1	[332]
Rodrigues et al., 2011	Spain	HIV	Trypanosoma	High	ELISA, IFAT, PCR	Cross sectional	2/19	1	[333]
Rodrigues et al., 2008	Brazil	HIV	Cryptosporidium, Isospora, Entamoeba, Endolimax, Iodamoeba, Giardia, Chilomastix	Upper middle	ZNS, FACS, Lutz Method	Cohort	287/582	2	[136]
Rodrigues et al., 2005	United state	HIV	Trypanosoma	High	FACT, DM, PCR	Cross sectional	18/28	1	[334]
Roka et al., 2012	Guinea	HIV	Iodamoeba, Giardia, Cryptosporidium, Endolimax, Chilomastix, Entamoeba, Blastocystis, Entamoeba, Pentatrichomonas	Low	RDT, ZNS, DM	Cross sectional	274/260	2	[137]
Roka et al., 2013	Guinea	HIV	Entamoeba, Giardia, Cryptosporidium, Endolimax, Entamoeba, Chilomastix, Iodamoeba, Blastocystis, Pentatrichomonas	Low	RDT, ZNS, DM	Cross sectional	257/273	2	[138]
Rostami et al., 2014	Iran	HIV	Toxoplasma	Lower middle	PCR, ELISA	Cross sectional	19/94	2	[335]
Saksirisampant et al., 2009	Thailand	HIV	Cryptosporidium, Blastocystis, Cyclospora, Isospora	Upper middle	ZNS, PCR	Cross sectional	35/90	2	[139]
Samie et al., 2014	South Africa	HIV	Cryptosporidium, Entamoeba, Giardia, Blastocystis	Upper middle	DM, ZNS, PCR	Cross sectional	101/151	1	[140]
Sanyaolu et al., 2013	Nigeria	HIV	Plasmodium	Lower middle	DM, RDT	Cross sectional	31/65	2	[336]
Sanyaolu et al., 2011	Nigeria	HIV	Iodamoeba, Entamoeba, Giardia, Cryptosporidium, Isospora	Lower middle	RDT, DM, FECT, ZNS	Cross sectional	26/65	2	[141]
Saracino et al., 2012	Mozambique	HIV	Plasmodium	Low	DM, RDT	Cross sectional	29/220	1	[337]
Sarfati et al., 2006	Cameroon	HIV	Isospora, Cryptosporidium, Giardia, Entamoeba	Lower middle	Baermann method, ZNS, PCR	Cross sectional	17/154	2	[338]
Scotto and Fazio, 2018	Sub-Saharan Africa**	HBV	Plasmodium	Upper middle	DM, PCR	Cross sectional	15/41	1	[339]

Table S3: Included studies of protozoan coinfections in virus-infected people.

Shafieenia et al., 2018	Iran	HIV	Toxoplasma	Lower middle	ELISA, PCR	Cross sectional	142/379	2	[340]
Shafiei et al., 2014	Iran	HIV	Leishmania	Lower middle	DAT, Elisa, Western blot	Cross sectional	9/49	1	[341]
Shafiei et al., 2011	Iran	HIV	Toxoplasma	Lower middle	IFA, ELISA, Western blot	Cross sectional	49/121	3	[342]
Shen et al., 2016	China	HIV	Toxoplasma	Upper middle	ELISA	Cross sectional	25/259	1	[343]
Sherchan et al., 2012	Nepal	HIV	Entamoeba, Giardia, Isospora, Cryptosporidium, Cyclospora, Blastocystis	Lower middle	ELISA, ZNS	Cross sectional	44/146	1	[344]
Sherpa et al., 2010	India	HIV	Cryptosporidium, Cyclospora, Giardia	Lower middle	ZNS, RDT	Cross sectional	15/106	0	[142]
Shimelis and Tadesse, 2014	Ethiopia	HIV	Cryptosporidium	Low	DM, ZNS, RDT	Cross sectional	32/250	2	[345]
Shimelis et al., 2016	Ethiopia	HIV	Entamoeba, Giardia, Cryptosporidium, Isospora, Cyclospora	Low	RDT, ZNS, FECT	Cross sectional	138/491	2	[143]
Silva et al., 2005	Brazil	HIV	Cryptosporidium, Isospora, Giardia	Upper middle	ELISA, MEIA, Baermann method, Lutz, Ritchie method	Cross sectional	12/100	1	[144]
Simpore et al., 2006	Burkina Faso	HIV	Toxoplasma	Low	ELISA, RDT	Cross sectional	59/207	1	[346]
Simpore et al., 2006	Burkina Faso	HBV	Toxoplasma	Low	ELISA, RDT	Cross sectional	12/33	1	[346]
Simpore et al., 2006	Burkina Faso	HCV	Toxoplasma	Low	ELISA, RDT	Cross sectional	4/18	1	[346]
Sintayehu et al., 2013	Ethiopia	HIV	Giardia, Cryptosporidium, Isospora, Entamoeba	Low	FACS, FECT, DM	Cross sectional	78/343	2	[81]
Sitoe et al., 2010	Mozambique	HIV	Toxoplasma	Low	ELISA	Cross sectional	18/58	0	[347]
Sonali et al., 2014	India	HIV	Giardia, Cryptosporidium, Isospora, Entamoeba	Lower middle	FACS, ZNS	Cross sectional	68/250	1	[146]
Srisuphanunt et al., 2008	Thailand	HIV	Cryptosporidium	Upper middle	ZNS	Cross sectional	23/143	0	[348]
Stark et al., 2007	Australia	HIV	Enteromonas, Entamoeba, Giardia, Cryptosporidium, Endolimax, Chilomastix, Trichomonas, Retortamonas, Dientamoeba, Blastocystis, Iodamoeba	High	ELISA, PCR, FECT	Cohort	288/628	2	[349]
Stensvold et al., 2011	Denmark	HIV	Isospora, Giardia, Cryptosporidium, Entamoeba, Dientamoeba, Blastocystis	High	FECT, PCR	Cross sectional	32/96	1	[350]
Sucilathangam et al., 2012	India	HIV	Cryptosporidium, Cyclospora	Lower middle	AFS, DM	Case control	52/103	0	[351]
Suryawanshi et al., 2012	India	HIV	Entamoeba, Giardia, Cryptosporidium, Isospora	Lower middle	ELISA, AFS	Cross sectional	43/100	1	[147]
Tagoe and Boachie, 2012	Ghana	HIV	Plasmodium	Lower middle	RDT, FACS	Cross sectional	34/220	1	[352]

Table S3: Included studies of protozoan coinfections in virus-infected people.

Taherkhani et al., 2007	Iran	HIV	Cryptosporidium	Lower middle	WMT, FECT	Cross sectional	20/75	1	[353]
Tay et al., 2015	Ghana	HIV	Plasmodium	Lower middle	DM, FACS	Cross sectional	47/400	2	[354]
Tamirat et al., 2017	Ethiopia	HIV	Entamoeba, Endolimax, Giardia, Blastocystis	Low	RDT, Baermann method, DM, FECT	Cross sectional	46/226	2	[150]
Tegegne et al., 2017	Ethiopia	HIV	Entamoeba, Cryptosporidium, Cyclospora, Giardia	Low	FACS, AFS, WMT, FECT	Cross sectional	36/223	2	[80]
Tegenaw et al., 2013	Ethiopia	HIV	Plasmodium	Low	DM, RDT	Cross sectional	2/23	2	[355]
Tellevik et al., 2015	Tanzania	HIV	Cryptosporidium	Lower middle	PCR	Case control	8/33	3	[356]
Thompson et al., 2012	Kenya	Influenza virus	Plasmodium	Lower middle	PCR	Cross sectional	149/2408	3	[357]
Tian et al., 2012	China	HIV	Entamoeba, Cryptosporidium, Giardia, Blastocystis	Upper middle	ELISA, Kato-Katz	Case control	70/302	2	[151]
Tian et al., 2013	China	HIV	Entamoeba, Giardia, Cryptosporidium, Blastocystis	Upper middle	FACS, ELISA, Kato-Katz	Case control	18/79	2	[152]
Tigist et al., 2015	Ethiopia	HIV	Entamoeba, Giardia	Low	FECT, RDT	Cross sectional	38/209	2	[153]
Tiwari et al. 2013	Nepal	HIV	Cryptosporidium, Cyclospora, Giardia, Entamoeba, Blastocystis	Lower middle	WMT, AFS, FECT, FACS	Cross sectional	84/745	2	[154]
Tsegaye et al., 2014	Ethiopia	HIV	Toxoplasma	Low	ELISA	Cross sectional	150/170	1	[358]
Tuli et al., 2008	India	HIV	Cryptosporidium, Cyclospora, Giardia, Entamoeba, Isospora	Lower middle	ELISA, FACS, AFS	Case control	284/366	2	[155]
Tumwine et al., 2005	Uganda	HIV	Cryptosporidium	Low	PCR	Cross sectional	67/91	3	[359]
Uneke et al., 2005	Nigeria	HIV	Plasmodium	Lower middle	DM, RDT	Cross sectional	103/490	1	[360]
Uysal et al., 2017	Turkey	HIV	Cyclospora, Cryptosporidium	Upper middle	ZNS, AFS, PCR	Cross sectional	6/115	1	[361]
Vouking et al., 2014	Cameroon	HIV	Entamoeba, Cryptosporidium, Isospora	Lower middle	Kato-Katz, FECT, ZNS	Cross sectional	73/207	2	[156]
Vyas et al., 2013	India	HIV	Entamoeba, Cryptosporidium, Isospora, Giardia	Lower middle	FACS, WMT, FECT	Cross sectional	27/75	0	[157]
Wang et al., 2013	China	HIV	Cryptosporidium	Upper middle	PCR, ZNS	Case control	11/683	3	[362]
Wanyiri et al., 2013	Kenya	HIV	Cyclospora, Entamoeba, Cryptosporidium, Isospora, Giardia	Lower middle	FACS, ZNS, DM, AFS	Cross sectional	68/164	1	[158]
Wanyiri et al., 2014	Kenya	HIV	Cryptosporidium	Lower middle	PCR, ELISA	Cross sectional	56/164	1	[363]
Wumba et al., 2010	Congo	HIV	Cryptosporidium, Entamoeba, Isospora, Giardia, Sarcocystis	Lower middle	ZNS, PCR	Cross sectional	33/175	2	[159]
Wumba et al., 2012	Congo	HIV	Iodamoeba, Giardia, Entamoeba, Endolimax, Entamoeba, Cryptosporidium	Lower middle	ZNS, PCR	Cross sectional	43/242	2	[160]
Wumba et al., 2015	Congo	HIV	Plasmodium	Lower middle	DM, PCR, RDT	Cross sectional	19/25	3	[364]
Xavier et al., 2013	Brazil	HIV	Toxoplasma	Upper middle	IFAT	Cross sectional	200/250	2	[365]
Yosefi et al., 2012	Iran	HIV	Blastocystis, Dientamoeba, Endolimax, Cryptosporidium, Entamoeba, Giardia	Lower middle	MIF, ZNS, ELISA	Cross sectional	27/60	1	[366]

Table S3: Included studies of protozoan coinfections in virus-infected people.

Yu et al. 2011	China	HBV	Cryptosporidium	Upper middle	AFS, PCR, APS	Cross sectional	14/340	2	[367]
Zeleke and Melsew, 2017	Ethiopia	HIV	Toxoplasma	Low	ELISA	Cross sectional	255/270	2	[368]
Zeynudin et al., 2013	Ethiopia	HIV	Entamoeba, Cryptosporidium, Giardia, Isospora	Low	ZNS	Cross sectional	31/91	1	[369]
Zheng et al., 2017	Guinea	HIV	Plasmodium	Low	DM, ELISA, Western blot	Cross sectional	14/101	3	[370]
Anabire et al., 2019	Ghana	HBV	Plasmodium	Lower middle	RDT, PCR	Cross sectional	36/191	3	[371]
Bavand et al., 2019	Iran	HIV	Toxoplasma	Lower middle	ELISA, qRT-PCR	Cross sectional	66/149	2	[372]
Chukwuocha et al., 2019	Nigeria	HIV	Plasmodium	Lower middle	DM, RDT	Cross sectional	398/398	2	[373]
Gebrecherkos et al., 2019	Ethiopia	HIV	Cystoisospora, Isospora, Entamoeba, Giardia, Cryptosporidium, Giardia	Low	DM, ZNF, FECT, FACS	Cross sectional	59/150	1	[162]
Masoumi et al., 2019	Iran	HIV	Cyclospora, Giardia, Blastocystis	Lower middle	FECT, PCR	Cross sectional	4/102	2	[374]
Sannella et al., 2019	Thailand	HIV	Cryptosporidium, Isospora, Giardia, Entamoeba	Upper middle	DM, PCR	Cross sectional	113/155	2	[164]
Abange et al., 2020	Cameroon	HIV	Cryptosporidium, Giardia, Blastocystis, Trichomonas, Endolimax, Iodamoeba, Entamoeba	Lower middle	DM, Kato–Katz, ZNS, FECT, FACS	Cross sectional	24/119	2	[51]
Abdullahi et al., 2020	Nigeria	HIV	Leishmania	Lower middle	ELISA, RDT	Case control	28/58	1	[375]
Alemayehu et al., 2020	Ethiopia	HIV	Entamoeba, Giardia	Low	FECT, FACS, DM	Cross sectional	15/383	2	[165]
Amodu et al., 2020	Nigeria	HIV	Plasmodium	Lower middle	ELISA, PCR	Cross sectional	100/140	3	[376]
Anyanwu et al., 2020	Nigeria	HIV	Plasmodium	Lower middle	EIA, DM, RDT	Cross sectional	142/420	3	[377]
Ayele et al., 2020	Ethiopia	HIV	Giardia, Cryptosporidium	Low	DM, FECT,	Cross sectional	31/180	1	[378]
Barbosa et al., 2020	Brazil	HIV	Leishmania	Upper middle	PCR, RDT, ELISA, FACS	Retrospective	65/113	1	[379]
Barbosa et al., 2020	Brazil	HIV	Leishmania	Upper middle	PCR, RDT	Cross sectional	110/309	2	[380]
Belay et al., 2020	Ethiopia	HIV	Entamoeba, Giardia, Cryptosporidium, Cyclospora	Low	DM, ZNS, FECT	Cross sectional	22/112	2	[166]
Bokharaei et al., 2020	Iran	HIV	Toxoplasma	Lower middle	ELISA, PCR	Cross sectional	95/108	1	[381]
Charoensakulchai et al., 2020	Thailand	HIV	Leishmania	Upper middle	PCR	Cross sectional	110/526	3	[382]
Chhangte et al., 2020	India	HIV	Entamoeba, Giardia, Blastocystis, Cryptosporidium, Cystoisospora, Cyclospora	Lower middle	FECT, AFS, DM	Cross sectional	61/250	2	[167]
Cunha et al., 2020	Brazil	HIV	Leishmania	Upper middle	PCR, IFAT, DAT, ELISA, RDT	Cross sectional	36/240	2	[383]
Dutta et al., 2020	India	HIV	Toxoplasma	Lower middle	ELISA	Cross sectional	292/400	1	[384]
Hoshina et al., 2020	Japan	HIV	Toxoplasma	High	ELISA, SFDT, PCR	Cross sectional	33/399	3	[385]

Table S3: Included studies of protozoan coinfections in virus-infected people.

Jegede et al., 2020	Nigeria	HIV	Plasmodium	Lower middle	DM, RDT	Cross sectional	386/1521	2	[386]
Lengongo et al., 2020	Gabon	HIV	Blastocystis, Entamoeba, Giardia, Entamoeba, Iodamoeba, Chilomastix, Cryptosporidium, Isospora	Upper middle	ZNS, MIFc, DM	Cross sectional	54/115	2	[168]
Medina et al., 2020	South Africa	HIV	Trichomonas	Upper middle	RDT	Retrospective	86/427	1	[387]
Moro et al., 2020	Brazil	HIV	Toxoplasma	Upper middle	ELISA	Cross sectional	81/332	1	[388]
Namaji et al., 2020	India	HIV	Cryptosporidium, Giardia, Entamoeba, Cystoisospora, Cyclospora	Lower middle	DM	Cross sectional	129/361	2	[170]
Onosakponome et al., 2020	Nigeria	SARS-CoV-2	Plasmodium	Lower middle	DM	Cross sectional	300/300	2	[389]
Rabiu et al., 2020	Nigeria	HIV	Plasmodium	Lower middle	DM, Kato–Katz	Cross sectional	49/197	1	[171]
Zakari et al., 2020	Nigeria	HIV	Toxoplasma	Lower middle	ELISA, Flow cytometry	Cross sectional	54/160	2	[390]
Barreto et al., 2022	Brazil	HTLV-1	Endolimax, Entamoeba, Giardia, Iodamoeba, Chilomastix, Entamoeba	Upper middle	ELISA, WB, DM, Baermann–Moraes method	Cross sectional	38/178	2	[180]
Purbey., 2021	India	HIV	Entamoeba, Cryptosporidium, Giardia	Lower middle	DM, FECT	Case control	7/75	2	[182]
Sandie et al., 2021	Cameroon	HIV	Plasmodium	Lower middle	DM, ZNS, FECT, RDT, FACT count	Cohort	31/190	3	[391]
Sepahvand et al., 2022	Iran	HIV	Cryptosporidium	Lower middle	ELISA, DM, FECT	Cross sectional	7/26	3	[392]
Stiffler et al., 2021	Kenya	HIV	Plasmodium	Lower middle	qPCR, RDT	Cross sectional	44/129	3	[393]
Steinberg et al., 2021	Bolivia	HIV	Toxoplasma	Lower middle	WB, qPCR,	Cross sectional	173/215	2	[394]
Uzairue et al., 2021	Nigeria	HIV	Cryptosporidium	Lower middle	FACS, ZNS, FECT	Cross sectional	25/170	1	[183]
Yulfi et al., 2021	Indonesia	HIV	Cryptosporidium, Blastocystis	Lower middle	ELISA, AFS, ZNS	Cross sectional	18/54	2	[395]
Wolday et al., 2021	Ethiopia	SARS-CoV-2	Entamoeba, Giardia	Low	RT-PCR	Cohort	152/751	3	[181]
Weinreich et al., 2021	Ghana	HIV	Cryptosporidium	Lower middle	RT-PCR	Cross sectional	86/967	2	[396]
Ajayi et al., 2021	Nigeria	HIV	Entamoeba, Cryptosporidium, Isospora, Cyclospora, Balantidium	Lower middle	FEST, ZNS	Cross sectional	94/375	2	[178]
Alonso et al., 2021	Mozambique	HIV	Plasmodium	Low	qPCR	Retrospective	26/144	2	[397]
Bayleyegn et al., 2021	Ethiopia	HIV	Entamoeba, Giardia, Cryptosporidium	Low	Giemsa staining, FECT	Cross sectional	30/255	1	[173]
Betancourth et al., 2021	Honduras	HIV	Cryptosporidium, Cystospore, Chilomastix, Endolimax, Entamoeba, Giardia, Iodamoeba, Blastocystis, Trichomonas	Lower middle	PCR, DM, Kinyoun staining, RDT	Cross sectional	31/102	2	[174]
Botero et al., 2021	Colombia	HIV	Entamoeba, Blastocystis, Cystoisospora, Cryptosporidium	Upper middle	WMT, ZNS	Cross sectional	50/192	2	[172]
Chandi et al., 2021	India	HIV	Entamoeba, Cryptosporidium	Lower middle	ZNS	Cross sectional	10/80	1	[177]

Table S3: Included studies of protozoan coinfections in virus-infected people.

Dankwa et al., 2021	Ghana	HIV	Cryptosporidium	Lower middle	ELISA	Cross sectional	26/418	2	[398]
Fang et al., 2021	Cameroon	HIV	Toxoplasma	Lower middle	FACS, RDT	Cross sectional	164/325	2	[399]
Feleke et al., 2022	Ethiopia	HIV	Entamoeba, Giardia, Cryptosporidium, Isospora	Low	ZNS, FECT	Cross sectional	22/223	2	[175]
Jemikalajah et al., 2021	Nigeria	HIV	Plasmodium	Lower middle	Giemsa staining, RDT	Cross sectional	228/300	1	[400]
Miressa et al., 2021	Ethiopia	HIV	Giardia, Entamoeba	Low	FACS, DM	Cross sectional	73/230	1	[176]
McAtee et al., 2021	Bolivia	HIV	Trypanosoma	Lower middle	ELISA, qPCR, DM	Cross sectional	32/116	2	[401]
Sajedi et al., 2021	Iran	HIV	Toxoplasma	Lower middle	ELISA	Cross sectional	73/121	1	[402]

** Definitive country is not available.

Table S4: The prevalence rates of parasitic coinfections at genus level in HIV-infected people by meta-analysis

Parasite	Genus	OR [95% CI]
Helminths	*Ancylostoma*	2·79% (1·32-4·27)
	Ascaris	7·82% (6·15-9·49)
	Clonorchis	0·42% (0·00-1·05)
	Dicrocoelium	0·13% (0·00-0·38)
	Diphyllobothrium	9·01% (4·18-13·83)
	Echinosoma	5·13% (0·23-10·02)
	Enterobius	0·83% (0·55-1·10)
	Fasciolopsis	2·16% (0·29-4·04)
	Gymnophalloides	0·95% (0·00-2·81)
	Hymenolepis	0·51% (0·37- 0·64)
	Necator	14·16% (0·00-39·42)
	Opisthorchis	17·03% (1·22-32·84)
	Rhabditis	0·13% (0·00-0·38)
	Schistosoma	12·46% (5·82-19·10)
	Strongyloides	5·43% (4·11-6·74)
	Taenia	2·19% (1·52-2·85)
	Trichuris	4·82% (2·48-7·17)
	Wuchereria	30·44% (3·71-57·17)
Protozoa	*Balantidium*	1·07% (0·03-2·11)
	Blastocystis	10·61% (6·26-14·97)
	Chilomastix	1·24% (0·45-2·03)
	Cryptosporidium	14·27% (11·49-17·06)
	Cyclospora	3·08% (1·89-4·28)
	Cystoisospora	2·21% (0·60-3·82)
	Dientamoeba	2·76% (0·00-5·95)
	Endolimax	6·06% (3·03-9·09)
	Entamoeba	12·33% (10·09-14·57)
	Enteromonas	3·23% (0·00-9·45)
	Giardia	4·99% (4·18-5·81)
	Iodamoeba	2·00% (1·08-2·92)
	Isospora	4·58% (3·37-5·80)
	Leishmania	30·50% (21·65-39·35)
	Pentatrichomonas	3·18% (1·69-4·67)
	Plasmodium	34·96% (28·11-41·82)
	Retortamonas	0·16% (0·00-0·47)
	Sarcocystis	0·57% (0·00-1·69)
	Toxoplasma	48·85% (42·01-55·69)
	Trichomonas	14·75% (8·75-20·75)
	Trypanosoma	20·32% (0·00-42·37)

Text S1: Search strategy and rules for study selection

Search strategy

The reviewers searched four databases (Web of Science, Pub Med, Scopus and Jstor) for studies published from January 1, 2005 to April 30, 2022 by using terms “Parasite virus co-infection”, “Parasite Virus”, “Parasite Co-infection”, “helminth co-infection”, “helminth virus co-infection” or “helminth virus”.

Database	Key words					
	Parasite virus co-infection	Parasite virus	Parasite co-infection	Helminth co-infection	Helminth virus co-infection	Helminth virus
PubMed	("parasitation"[All Fields] OR "parasite s"[All Fields] OR "parasited"[All Fields] OR "parasites"[MeSH Terms] OR "parasites"[All Fields] OR "parasite"[All Fields] OR "parasitical"[All Fields] OR "parasitically"[All Fields] OR "parasitics"[All Fields] OR "parasiting"[	("parasitation"[All Fields] OR "parasite s"[All Fields] OR "parasited"[All Fields] OR "parasites"[MeSH Terms] OR "parasites"[All Fields] OR "parasite"[All Fields] OR "parasitical"[All Fields] OR "parasitically"[All Fields] OR "parasitics"[All Fields] OR "parasiting"[All Fields] OR "parasitism"[All Fields] OR "parasitisms"[All Fields] OR "parasitization"[All Fields] OR "parasitize"[All	("parasitation"[All Fields] OR "parasite s"[All Fields] OR "parasited"[All Fields] OR "parasites"[MeSH Terms] OR "parasites"[All Fields] OR "parasite"[All Fields] OR "parasitical"[All Fields] OR "parasitically"[All Fields] OR "parasitics"[All Fields] OR "parasiting"[All Fields] OR "parasitism"[All Fields] OR "parasitisms"[All Fields] OR "parasitization"[All Fields] OR "parasitize"[All	("helminthes"[All Fields] OR "helminthic"[All Fields] OR "helminths"[MeSH Terms] OR "helminths"[All Fields] OR "helminth"[All Fields]) AND ("coinfection"[MeSH Terms] OR "coinfection"[All Fields] OR ("co"[All Fields] AND "infection"[All Fields]) OR "co infection"[All Fields])	("helminthes"[All Fields] OR "helminthic"[All Fields] OR "helminths"[MeSH Terms] OR "helminths"[All Fields] OR "helminth"[All Fields]) AND ("virology"[MeSH Subheading] OR "virology"[All Fields] OR "viruses"[All Fields] OR "viruses"[MeSH Terms] OR "virus s"[All Fields] OR "viruse"[All Fields] OR "virus"[All Fields]) AND ("coinfection"[MeSH Terms]	("helminthes"[All Fields] OR "helminthic"[All Fields] OR "helminths"[MeSH Terms] OR "helminths"[All Fields] OR "helminth"[All Fields]) AND ("virology"[MeSH Subheading] OR "virology"[All Fields] OR "viruses"[All Fields] OR "viruses"[MeSH Terms] OR "virus s"[All Fields] OR "viruse"[All Fields] OR "virus"[All Fields])

	All Fields] OR "parasitism"[All Fields] OR "parasitisms"[All Fields] OR "parasitization"[All Fields] OR "parasitize"[All Fields] OR "parasitized"[All Fields] OR "parasitizes"[All Fields] OR "parasitizing"[All Fields] OR "parasitology"[MeSH Terms] OR "parasitology"[All Fields] OR "parasitic"[All Fields] OR "parasitology"[MeSH Subheading]) AND ("virology"[MeSH Subheading] OR "virology"[All Fields] OR	Fields] OR "parasitized"[All Fields] OR "parasitizes"[All Fields] OR "parasitizing"[All Fields] OR "parasitology"[MeSH Terms] OR "parasitology"[All Fields] OR "parasitic"[All Fields] OR "parasitology"[MeSH Subheading]) AND ("virology"[MeSH Subheading] OR "virology"[All Fields] OR "viruses"[All Fields] OR "viruses"[MeSH Terms] OR "virus s"[All Fields] OR "viruse"[All Fields] OR "virus"[All Fields])	Fields] OR "parasitized"[All Fields] OR "parasitizes"[All Fields] OR "parasitizing"[All Fields] OR "parasitology"[MeSH Terms] OR "parasitology"[All Fields] OR "parasitic"[All Fields] OR "parasitology"[MeSH Subheading]) AND ("coinfection"[MeSH Terms] OR "coinfection"[All Fields] OR ("co"[All Fields] AND "infection"[All Fields]) OR "co infection"[All Fields])		OR "coinfection"[All Fields] OR ("co"[All Fields] AND "infection"[All Fields]) OR "co infection"[All Fields])	

	"viruses"[All Fields] OR "viruses"[MeSH Terms] OR "viruses"[All Fields] OR "viruse"[All Fields] OR "virus"[All Fields]) AND ("coinfection"[MeSH Terms] OR "coinfection"[All Fields] OR ("co"[All Fields] AND "infection"[All Fields]) OR "co infection"[All Fields])					
Scopus	TITLE-ABS-KEY (parasite AND virus AND co-infection) AND PUBYEAR > 2004 AND PUBYEAR < 2023	**TITLE-ABS-KEY (parasite AND virus) AND PUBYEAR > 2004 AND PUBYEAR < 2023**	**TITLE-ABS-KEY (parasite AND co-infection) AND PUBYEAR > 2004 AND PUBYEAR < 2023**	TITLE-ABS-KEY (helminth AND co-infection) AND PUBYEAR > 2004 AND PUBYEAR < 2023	TITLE-ABS-KEY (helminth AND virus AND co-infection) AND PUBYEAR > 2004 AND PUBYEAR < 2023	TITLE-ABS-KEY (helminth AND virus) AND PUBYEAR > 2004 AND PUBYEAR < 2023
Web of Science	(Parasite virus co-infection)	(Parasite virus)<br><br>Timespan: 2005 -	(Parasite co-infection)	(helminth co-infection)	(helminth virus co-infection)	(helminth virus)<br><br>Timespan: 2005 -

	Timespan: 2005-2022. Databases: WOS, CABI, CSCD, DIIDW, KJD, RSCI, SCIELO.<br>Search language=Auto	2022. Databases: WOS, CABI, CSCD, DIIDW, KJD, RSCI, SCIELO.<br>Search language=Auto	Timespan: 2005-2022. Databases: WOS, CABI, CSCD, DIIDW, KJD, RSCI, SCIELO.<br>Search language=Auto	Timespan: 2005-2022. Databases: WOS, CABI, CSCD, DIIDW, KJD, RSCI, SCIELO.<br>Search language=Auto	Timespan: 2005-2022. Databases: WOS, CABI, CSCD, DIIDW, KJD, RSCI, SCIELO.<br>Search language=Auto	2022. Databases: WOS, CABI, CSCD, DIIDW, KJD, RSCI, SCIELO.<br>Search language=Auto

Rules for Study Selection

We included studies (original research articles, observational studies, experimental studies or surveys) with the prevalence data of parasitic co-infection in people living with viruses. There was no limitation of sample size for inclusion. Language was restricted to English only. Studies were excluded if they were review articles, book chapters, patents, unpublished data, abstracts, conference paper, animal or plant studies, or studies without prevalence rates. In some studies, there were other pathogens like fungi and bacteria. These pathogens, however, were not included in our study because our focus was on co-infections with parasites (helminths and protozoa) and viruses.

The included studies were divided into helminth-virus and protozoan-virus co-infection groups for further process. If multiple parasitic co-infections were present in an article, we categorized the parasites into helminthic-virus and protozoan-virus groups.

Text S2: Quality assessment of included studies

Factor	Criteria for Ranking	Ranking	Score of Ranking	No. of helminthic coinfection studies (Total 135)	No. of protozoan coinfection studies (Total 323)
**Sample size**	≤200	Low	0	62	137
	>200	High	1	73	186
**Diagnostic technique**	Non-Confirmatory Test	Low	0	91	176
	Confirmatory Test	High	1	44	147
**Study design**	Experimental study	Low	0	2	1
	Observational study	High	1	133	322
**Quality**		high	3	20	73
		moderate	2	77	184
		low	0 or 1	38	66

Diagnostic Technique

Non-Confirmatory test (Low rank): This included Acid fast staining (AFS), Scotch-tape technique, Ryan's modified trichrome staining, Kinyoun staining, Rugai's method, Lutz technique, Micro particle enzyme immunoassay (MEIA), Baermann method, Enzyme immunoassay (EIA), immunofluorescence assay (IFA), High performance liquid chromatography (HPLC), Biochemical analysis, immunofluorescence antibody test (IFAT), Indirect Coombs Test (ICT), chromatographic immunoassay (CI), Safranin/Methylene blue method, Ritchie method, Weber's Chromotrope staining, Toxo-latex test, Complement fixation test (CFT), Merthiolate-iodine-formaldehyde Technique (MIF), Ziehl-Neelsen staining (ZNS), Direct microscopy (DM), Fluorescence activating cell sorting (FACS), Enzyme-linked immunosorbent assay (ELISA), Rapid diagnostic test (RDT), Giemsa staining, Wet mount technique (WMT), Formal-ether concentration technique (FECT) and Kato-Katz.

Confirmatory test (High rank): This included Western blot (WB), Polymerase chain reaction (PCR), Quantitative real time-PCR (qRT-PCR). When ELISA was conducted together with any fecal technique, it was included in the confirmatory test.

Study Design

Experimental (Low rank): This included randomized and non-randomized studies.

Observational (High rank): This included cross-sectional, retrospective, prospective, cohort, case control studies and case series.

Quality

The quality of included studies was assessed through factors of sample size, detection method and study design. Each factor was given 1 point if a study belonged to high rank, and 0 point if a study belonged to low rank. The overall rating of a study's quality was calculated by adding the ranking score of each factor (sample size, detection method and study design). The studies were divided into 3 quality categories i.e., high, moderate and low quality. The records with score 3 were placed in high-quality group, score-2 records in moderate-quality group, and score-0 and 1 records in low-quality group.

Text S 3: Egger's linear regression test and Begg-Mazumdar rank correlation test on the pooled prevalence of helminth in virus-infected people

```
> print(egger_res)Helminth
Linear regression test of funnel plot asymmetry

Test result: t = 6.77, df = 121, p-value < 0.0001

Sample estimates:
   bias se.bias intercept se.intercept
 9.1036  1.3449   -0.0217       0.0185

Details:
- multiplicative residual heterogeneity variance (tau^2 = 92.3968)
- predictor: standard error
- weight:    inverse variance
- reference: Egger et al. (1997), BMJ
> print(begg_res)
Rank correlation test of funnel plot asymmetry

Test result: z = 7.12, p-value < 0.0001

Sample estimates:
        ks     se.ks
 3257.0000 457.4301

- reference: Begg & Mazumdar (1993), Biometrics
```

Text S4: Egger's linear regression test and Begg-Mazumdar rank correlation test on the pooled prevalence of protozoan in virus-infected people

```
> print(egger_res)Protozoa
Linear regression test of funnel plot asymmetry

Test result: t = -2.54, df = 306, p-value = 0.0116

Sample estimates:
    bias se.bias intercept se.intercept
 -5.8025  2.2849    0.4445       0.0315

Details:
- multiplicative residual heterogeneity variance (tau^2 = 1000.0970)
- predictor: standard error
- weight:    inverse variance
- reference: Egger et al. (1997), BMJ
> print(begg_res)
Rank correlation test of funnel plot asymmetry

Test result: z = 10.58, p-value < 0.0001

Sample estimates:
        ks     se.ks
 19107.0000 1806.1478

- reference: Begg & Mazumdar (1993), Biometrics
```
